# Cellular Interactions in the Tumor Microenvironment: The Role of Secretome

**DOI:** 10.7150/jca.21780

**Published:** 2019-08-07

**Authors:** Bianca Rodrigues da Cunha, Célia Domingos, Ana Carolina Buzzo Stefanini, Tiago Henrique, Giovana Mussi Polachini, Pedro Castelo-Branco, Eloiza Helena Tajara

**Affiliations:** 1 Department of Molecular Biology, School of Medicine of São José do Rio Preto/FAMERP, São José do Rio Preto, SP, Brazil.; 2 Department of Genetics and Evolutionary Biology, Institute of Biosciences, University of São Paulo, SP, Brazil.; 3 Department of Biomedical Sciences and Medicine, University of Algarve, Portugal.; 4 Centre for Biomedical Research (CBMR), University of Algarve, Faro, Portugal.; 5 Algarve Biomedical Center, Gambelas, Faro, Portugal.

**Keywords:** Cancer, Microenvironment, Secretome.

## Abstract

Over the past years, it has become evident that cancer initiation and progression depends on several components of the tumor microenvironment, including inflammatory and immune cells, fibroblasts, endothelial cells, adipocytes, and extracellular matrix. These components of the tumor microenvironment and the neoplastic cells interact with each other providing pro and antitumor signals. The tumor-stroma communication occurs directly between cells or via a variety of molecules secreted, such as growth factors, cytokines, chemokines and microRNAs. This secretome, which derives not only from tumor cells but also from cancer-associated stromal cells, is an important source of key regulators of the tumorigenic process. Their screening and characterization could provide useful biomarkers to improve cancer diagnosis, prognosis, and monitoring of treatment responses.

## Introduction

The secretome consists of a subset of proteins and metabolites released by a cell, tissue or an organism, which plays an important role in the regulation of cell-to-cell interactions essential to their normal physiological functions. Secretome alterations are often associated with atypical cellular phenotypes that are indicative of diseases such as cancer.

Secretome analysis has identified biomarkers for different pathological conditions including chronic inflammation, neurodegenerative disorders, cancer, cardiovascular disease, and diabetes. Moreover, secretome studies have contributed to better understand the molecular mechanisms involved in the pathogenesis of several diseases and to the development of novel treatment strategies 1, 2. Specifically, mutations that underlie cancer progression can lead to altered protein expression patterns, which can be detected in serum and other body fluids allowing for early diagnosis and prediction to treatment response 3.

## The Secretome

The term secretome was first used in a study by Tjalsma et al. in the year of 2000 4 to designate the whole secretory processes in the bacteria *Bacillus subtilis*. The definition of secretome was later expanded to denote the global group of proteins secreted, released or detached into the extracellular space by a cell, tissue, organ or organism at any given time 5. Such proteins comprise a large variety of bioactive molecules that play important roles in regulating cell-cell and cell-extracellular matrix interactions. They may act in an autocrine or paracrine fashion, positively or negatively influencing the ability of cells to survive, proliferate and differentiate 6.

Proteins secreted via the classical pathway, are synthesized in the endoplasmic reticulum (ER) presenting a signal peptide at their N-terminus 7. They are transported to the Golgi apparatus in a coat protein complex II (COPII)-coated vesicles and then, within secretory vesicles, to the plasma membrane and cell exterior 8, 9. During their passage through the Golgi apparatus, proteins are modified by different processes such as glycosylation 10, phosphorylation 11 and palmitoylation 12, 13.

Proteins can also be exported by the non-classical pathway via Golgi-independent mechanisms. This pathway corresponds to the translocation of proteins from the cytoplasm directly across the plasma membrane into the extracellular compartment. Examples include the fibroblast growth factor 2 (FGF2) and cytokines involved in inflammation and angiogenesis 14. The non-classical pathway uses vesicular and non-vesicular transport mechanisms. The non-vesicular mechanisms encompass self-sustained protein translocation through plasma membranes and ABC transporter-mediated secretion. The vesicular mechanism depends on intermediate carriers such as endosomes, multivesicular bodies (MVBs), autophagosomes and secretory lysosomes that fuse to the plasma membrane to release their contents directly to the cell exterior or in a vesicular/exosome-dependent manner 15, 16.

Secreted proteins are involved in a variety of physiological processes like immune response, blood coagulation, digestion, cell signaling and cell communication, and include hormones, proteases and digestive enzymes, immunoregulatory cytokines/chemokines and growth factors 17. Non-protein compounds, such as DNAs, RNAs, and lipids, as well as proteins, can be exported by exosomes 1, 18, 19 and mediate important intercellular signaling pathways 20, which may influence local and even distant microenvironments 21.

In addition to participating in many normal physiological processes, secretome components are involved in the pathogenesis of several diseases, such as cancer, and may promote invasion of the surrounding tissues, evasion of immune defenses and distant organ colonization 6. Therefore, a better understanding of the mechanisms regulating the secretome can lead to the development of novel targeted therapies.

## Secretome in Cancer

For many years, cancer was considered a stand-alone cell process. The concept was focused on genetically transformed cells and their progression to a malignant condition 22. However, it is becoming clear that cancer cells do not act isolated during their progression to malignancy. The primary tumor cells recruit and activate non-transformed stromal cells, including fibroblasts, endothelial, inflammatory and immune system cells 22, 23, in order to promote a microenvironment favorable to disease progression 1, 22. Besides stromal cells, the tumor microenvironment contains the extracellular matrix (ECM), which is rich in collagens, proteoglycans, hyaluronic acid, laminins and fibronectin 24, and provides the support structure that facilitates tumor proliferation and dissemination. This non-cellular macromolecular structure functions therefore as a scaffold for tumor tissue organization and contributes to the network of cell interactions mediated by surface receptors and embedded signaling molecules 23.

The tumor-stroma communication occurs directly between cells and, as cited above, via a variety of molecules secreted, such as growth factors, cytokines, chemokines and microRNAs 25, 26. Biochemical events may also arise from the mechanical properties and physical signals of the microenvironment, including contractile forces and matrix stiffness 27. The heterotypic secretome, which derives not only from tumor cells but also from cancer-associated stromal cells, is an important source of markers and key regulators of the tumorigenic process 28, 29. The tumor microenvironment is therefore much broader than the central unit formed by the neoplasm. Its complexity is due to the resident or recruited elements and multidirectional and dynamic interactions between them, which end up generating a loss of the normal tissues architecture, sites of inflammation, hypoxia and consequently neoangiogenesis to contemplate the high needs for oxygen and nutrients 1, 29.

Alterations of secretome composition during tumor development and progression depend on genetic mutations and nonmutational changes that affect gene expression. For example, high expression of the transcriptional activator c-MYC can result in altered levels of several secreted factors associated with cell proliferation, such as transforming growth factor beta-2 (TGF-beta-2). Target genes activated by a mutant tumor suppressor protein p53 also code secreted factors that may increase invasion of cancer cells. Likewise, loss of another tumor suppressor protein, the phosphotidyl-inositol phosphatase (PTEN), induces a more aggressive secretome that has been linked to tumor invasion and metastasis (for references, see 1). Hawkins and collaborators 30 recently reported that activation of Wnt signaling in Ewing sarcoma cells results in an enrichment of secreted proteins involved in ECM composition, organization and degradation, potentially affecting the crosstalk between cancer cells and their microenvironment and therefore impacting tumor progression.

The data above paint a picture where neoplastic cells acquire mutations and secrete factors aiming to create, in a figurative sense, a scene with actors and scenery working to benefit the tumor, which means survival, proliferation, invasion and metastasis. The outcome, if positive or negative for the host, will be driven by classical natural selection influenced by the initial conditions, both constitutive and micro-/macro-environmental, and their interactions. Strategies to direct or even control this Darwinian character of cancer focusing on neoplastic cells and their habitat may overcome current therapeutic limitations, as previously discussed by Greaves and Maley 31.

## The crosstalk between neoplastic and stromal cells

An early step in cancer progression is the epithelial-mesenchymal transition (EMT), a cellular process characterized by changing from epithelial to a mesenchymal phenotype, which increases the capacity of migration, invasion, and apoptosis resistance. This process also occurs during embryogenesis and in tissue regeneration after injury, the latter usually associated with inflammation 32.

During the neoplastic process, remodeling of the extracellular matrix follows epithelial-mesenchymal transition. Analysis of the cell secretome at this stage has shown increased expression of proteases, ECM components, factors that promote migration and reduced expression of adhesion proteins 33, 34. The secretome of non-transformed cells with sustained expression of c-Myc also modulate the composition/function of ECM and basement membrane and show high ability to induce proliferation, overcome senescence, and to attract fibroblasts. Alterations of matrix components can be seen as an event that is able to interrupt normal tissue organization. Moreover, the ability to attract fibroblasts suggests that the secretome can change the crosstalk between epithelial and stromal cells in early stages of tumorigenesis 6.

Fibroblasts represent the most abundant cells of the tumor stroma and play a critical role in tumor growth, survival, proliferation, migration, invasion, and metastasis 35, 36. Under normal conditions, the fibroblasts are quiescent and are activated during tissue regeneration after injury, originating myofibroblasts. In the tumor microenvironment, fibroblast activation is induced by stimuli derived from neoplastic cells or immune system cells, particularly growth factors and interleukins, which lead to changes in its morphology and function 37. It also seems to be self-induced by mutations leading to loss of the members of the tissue inhibitor of metalloproteinases (*TIMP*) gene family, which are associated with the control of the extracellular matrix through direct inhibition of metalloproteinases 38. The activated fibroblasts are often called cancer-associated fibroblasts (CAFs) and comprise a heterogeneous cell population identified by a number of markers, including the alpha-smooth muscle actin (alpha-SMA) protein 39. This protein is also present in resident fibroblasts and myofibroblasts 40, 41.

CAFs may originate from different resident precursors such as fibroblasts chronically exposed to oxidative stress 42 or from senescent cells with profiles similar to the senescence-associated secretory phenotype 43. Bone marrow-derived mesenchymal stem cells (BMSCs) can also differentiate in CAFs. This conclusion is based on the observation that colon cancer cells stimulate differentiation of BMSCs to a CAFs-like phenotype with increased expression of alpha-SMA and vimentin. Importantly, it was also observed that Notch signaling mediates transformation of BMSCs to CAFs through the downstream TGF-beta/Smad signaling pathway 44. The cytokine TGF-beta is secreted in a latent form 45 that upon activation becomes a potent inducer of epithelial-mesenchymal transition 46. Mesenchymal cells with chromosomal abnormalities similar to those found in adjacent epithelial cancer cells express alpha-SMA and vimentin and do not express keratin 47. These findings suggest that CAFs can also be directly derived from neoplastic cells that have undergone epithelial-mesenchymal transition by TGF-beta induction. Other cytokines participate in fibroblast activation including the interleukin 6 (IL-6) that reduces the expression of the tumor suppressor proteins p16^INK4A^, p21, and p53 in fibroblasts. IL-6 acts on the Jak2/STAT3 pathway through negative control of AUF1 (hnRNP D0) and promotes the expression of myofibroblast markers such as alpha-SMA 48.

**Figure 1:**
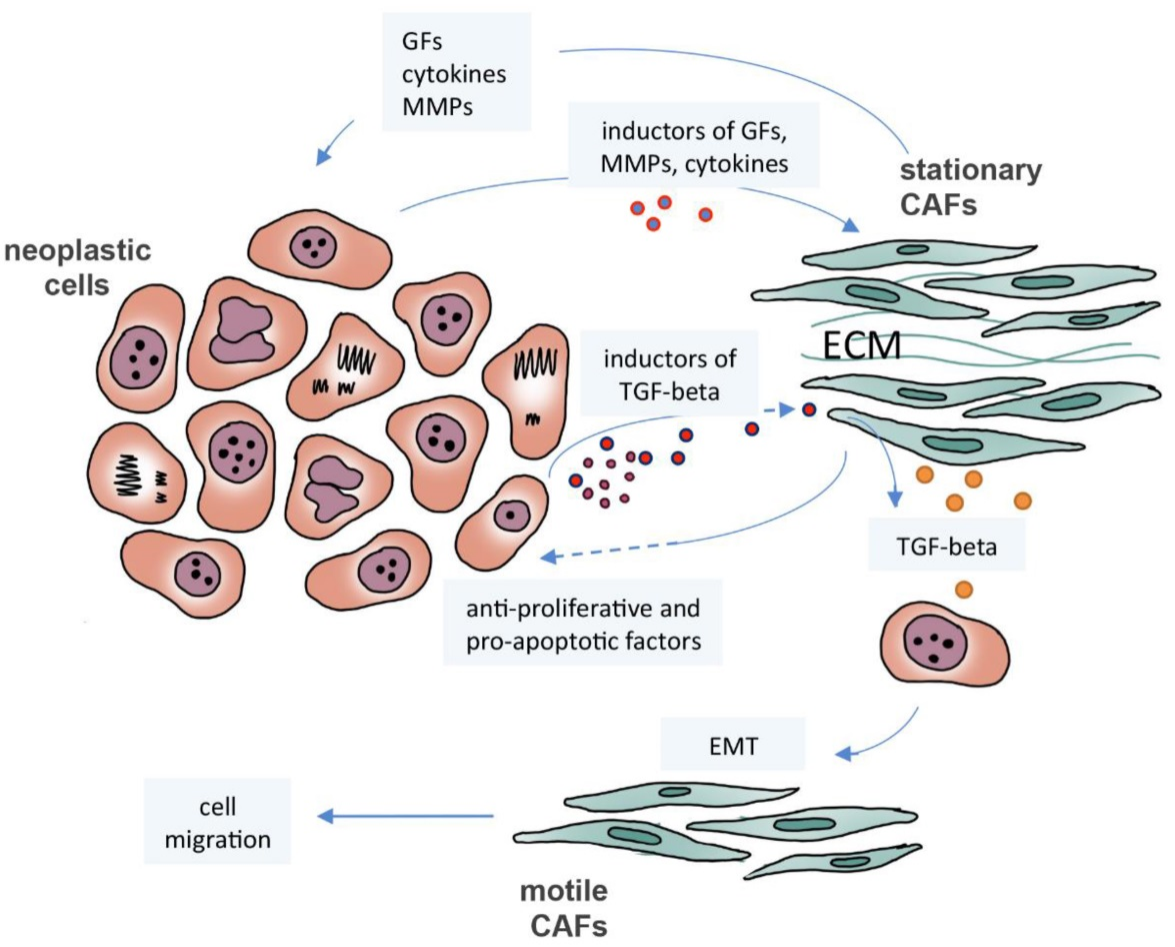
** The crosstalk between neoplastic cells and cancer-associated fibroblasts (CAFs).** Neoplastic cells induce stationary CAFs to synthesize cytokines, matrix metalloproteinases (MMPs), and growth factors (GFs), such as TGF-beta, leading to proliferation, migration, invasion and metastasis. Motile CAFs can be derived from neoplastic cells that have undergone epithelial-mesenchymal transition (EMT) by TGF-beta induction. CAFs may exhibit tumor suppressive activities inducing apoptosis and preventing proliferation.

CAFs constitute a heterogeneous population with a divergent transcriptome and secretome. In oral carcinomas, Costea and collaborators identified two distinct CAF subtypes: one with transcriptome and secretome similar to normal fibroblasts, high number of motile cells and cells responsive to TGF-beta; and another subtype with a dissimilar transcriptome, few motile cells and a high secretion of TGF-beta 49. This heterogeneity suggests the existence of a subpopulation of stationary/low migratory fibroblasts responsible for the synthesis of various growth factors, cytokines and matrix metalloproteinases (MMPs) 49, which may be activated by signals emanating from neoplastic cells 50, 51. Indeed, several studies on cancer-derived myofibroblast secretome observed an increase in the production and activation of MMP-1, -2, and -3, which may contribute to the remodeling of the cancer cell microenvironment and therefore to cell migration 52, as well as proteins associated with invasion and metastasis, or that act in the protection of cells against injuries caused by reactive oxygen species 38, 53. Contrary to their role in neoplastic progression, the signals derived from normal fibroblasts and CAFs can inhibit proliferation of colon cancer cells, as reported by Chen and collaborators 54. Similarly, the Tanaka group showed that CAFs induce apoptosis in gastric carcinoma cells, preventing proliferation-dependent invasion and suggesting a protective effect against cancer. However, the authors also observed that extracellular vesicles released by apoptotic cells activate fibroblasts and promote cancer invasion led by CAFs and tumor dissemination 55. Therefore, fibroblasts may exhibit tumor suppressive or pro-tumorigenic activities and, unfortunately, no specific marker is known to be effective in stratifying their subsets of phenotypes 56. From the data presented above, it is apparent that CAFs are an important stromal element that works in favor of but also against tumor development and progression through many factors released by these cells (**Figure 1**). Other stromal cells exhibit similar responses, such as macrophages and neutrophils, as will be discussed in the next sections.

Growing evidence from the literature has suggested that cancer stem cells (CSCs) or tumor-initiating cells show resistance to chemotherapeutic agents 57, 58. Su and collaborators recently demonstrated that this feature is supported by a subset of CAFs with high expression of the cell-surface molecules CD10 and GPR77. CD10^+^GPR77^+^ CAFs are insensitive to chemotherapy and associated with shorter survival in different cancer types. Moreover, they can promote the formation of a supporting niche for CSCs and induce chemoresistance in CSC by secreting pro-inflammatory interleukins IL-6 and IL-8, a function dependent of a persistent activation of the nuclear transcription factor kappa B (NF-kB) 59. These findings are of particular importance for the development of new approaches for diagnosis, prognosis, and monitoring treatment response in tumors driven by CSCs.

The role microenvironment as a key player in the development of chemoresistance has recently been analyzed by the Dzobo group 60, with special attention to mesenchymal stem cells, fibroblasts, macrophages, cancer stem cells, and EMC components. The authors highlighted the fact that an abnormal ECM composition rich in collagen activates signaling cascades such as the MEK-ERK pathway, which induces proliferation, survival, or even apoptosis. High expression of collagen also increases ECM stiffness, providing a physical barrier that reduces blood flow and drug delivery. In addition, the acidic character of this extracellular environment due to lactate accumulation generated by the high glycolytic rate of tumor cells facilitates drug inactivation - another step of the chemoresistance process 60.

Molecules in the tumor microenvironment derived from cancer or stromal cells, therefore, modulate biological processes that drive malignant progression, tumor response to drugs and the development of chemoresistance. In other words, metabolic pathways of cancer cells depend on their microenvironment components and also on the level of these components. Muir and Vander Heiden reported a number of examples showing the differences of drug response between tumor cells *in vivo* and cells in culture, which are due to differences in nutrients levels between tissues and standard culture medium 61. Understanding how the nutrients change metabolism is crucial for the development of new therapies and to identify efficient approaches for subsets of cancer patients carrying specific constitutive or somatic mutations.

## Senescence-Associated Secretome and Cancer

**Figure 2:**
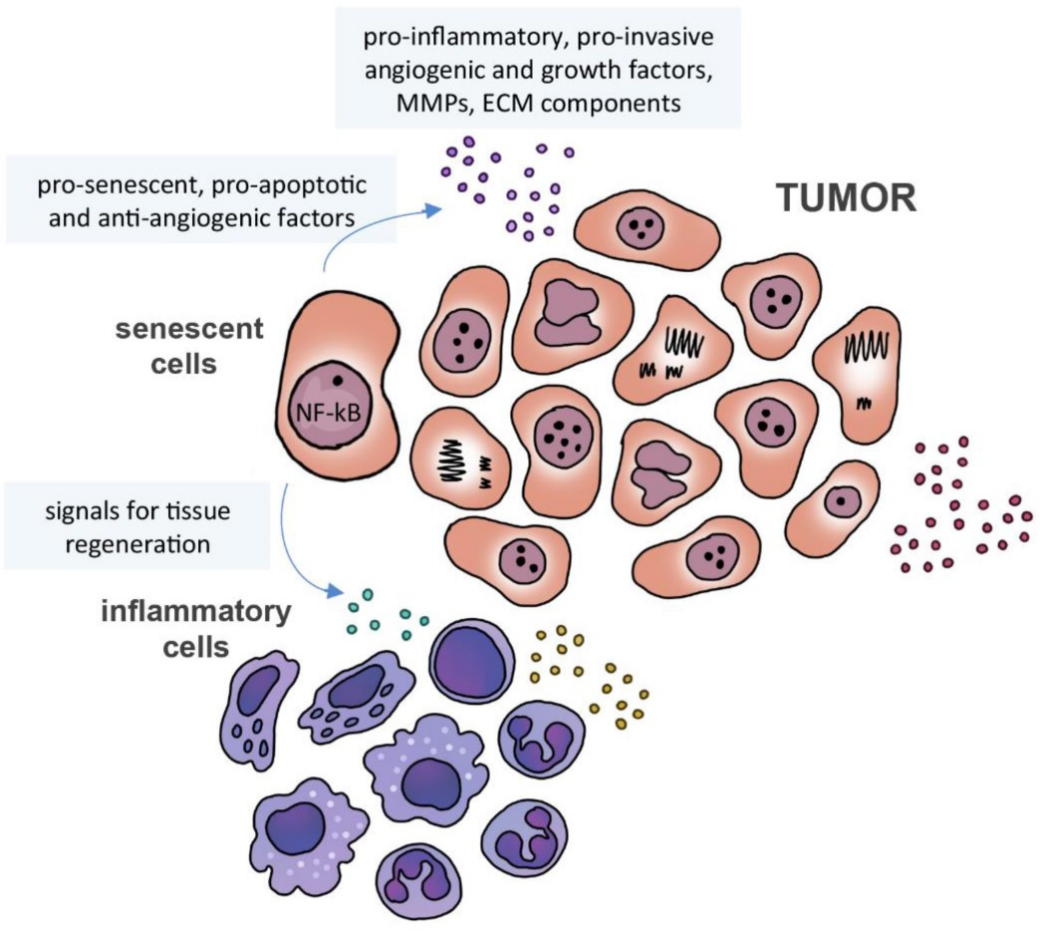
** Senescence-associated secretome.** Senescence-associated microenvironment is rich in matrix metalloproteinases (MMPs), cytokines, growth and angiogenic factors. Senescent cells may secrete pro-senescent, pro-apoptotic and anti-angiogenic factors, as well signals to induce immune cells to modulate tissue regeneration and senescent cell removal.

Senescence is a defense mechanism that has a great impact on the ability of cells to communicate, regardless of the cell type and source of the signal. This mechanism is a response to stress, which blocks cell proliferation and is often associated with expression of the tumor suppressor protein p16^INK4a^ and accompanied by a secretory phenotype (senescence-associated secretory phenotype - SASP) with stress-type-dependent profile 62-64. Cells in a senescent state and driven by NF-kB secrete 40-80 bioactive molecules, including pro-inflammatory interleukins and chemokines, growth and angiogenic factors, metalloproteinases and components of ECM, which act in a paracrine manner and can have both beneficial and detrimental effects 65-68. In fact, senescence may be seen as a cancer suppressor mechanism, with the inflammatory SASP phenotype inducing immune cells to modulate tissue regeneration and senescent cell removal, and secreting pro-senescent, pro-apoptotic and anti-angiogenic factors (reviewed by 69). However, the secretome of these cells also include factors that induce proliferation of premalignant cells *in vitro* and *in vivo* 70, 71 and stimulate migration and invasion of pre-neoplastic and endothelial cells (**Figure 2**). In addition, these factors 66, 72 may promote the formation of tumors *in vivo* and confer metastatic properties 73, 74.

As suggested by Muñoz-Espín D et al 75 and Storer M et al 76, senescence is a normal programmed mechanism, which operates similarly to apoptosis to remove cells at risk of abnormal cell growth. It is found in embryonic tissue remodeling and was probably adapted during evolution to act in tissue regeneration in the adult organism. A downside of this response is that the clearance of senescent cells can also remove non-senescent cells. Moreover, the number of senescent cells increases with age and the immune system may not be able to remove them completely, which can lead to a rich senescence-associated microenvironment with cytokines, growth and angiogenic factors, increasing the risk of malignant transformation 77. The physiological relevance and the consequences of senescence were evaluated by Baker et al 78, who observed that induction of apoptosis in p16^INK4A^-positive senescent cells extends lifespan, has a tumor-protective effect, and attenuates age-related deterioration of several organs. Differently, Takasugi and collaborators reported evidences of the relationship between senescence-associated secretome and tumorigenesis. The authors observed that exosome-like small extracellular vesicles (sEVs) secreted by senescent cells are enriched in receptor tyrosine kinase EphA, an expression event regulated by reactive oxygen species (ROS) levels. After the ephrin ligand binding, EphA can activate Erk pathway and mediate proliferative effects 79.

The literature has shown that oxidative stress and chemotherapeutic drugs promote senescence in sensitive neoplastic cells, and the formation of a proinflammatory and protumoral senescence-associated secretome driven by a poly(ADP-ribose) polymerase-1 (PARP-1) and NF-kB signaling. These data make plausible the hypothesis that a senescent phenotype induced by ROS and DNA damage may stimulate neighbor resistant cells to proliferate and invade surrounding tissues, ensuring tumor survival 74. This idea again evokes natural selection forces and the need to combine different drugs to target different cells within the neoplastic population.

## The Inflammatory Secretome and Cancer

It is estimated that 25% of cancers are inflammation-related cancers 80. The first observations on this association were carried out by the German physician Rudolf Virchow in the 19th century, when he described that leukocyte infiltration in tumors is linked to immune responses 81. Today it is widely recognized that inflammation promotes carcinogenesis. The hallmarks of cancer proposed by Hanahan and Weinberg in 2011 82 currently include two features that take into account inflammation: inflammatory environment and evasion of immune attack.

There are several examples of chronic inflammation that increase the risk of developing cancer. These include the predisposition of patients with Crohn's disease to colorectal cancer 83, *Helicobacter pylori* infection to gastric cancer 84, and human papillomavirus (HPV) infection to cervical cancer 85 and head and neck carcinoma 86, 87. Similarly, non-steroidal anti-inflammatory drugs decrease the risk of cancer death in various tumor types 88, 89.

The inflammatory processes may contribute to multiple stages of tumor development through growth factors, survival factors, proangiogenic factors, signals that activate EMT, enzymes that modify the extracellular matrix and facilitate invasion and metastasis, as well as reactive oxygen species that are actively mutagenic and promote neoplastic progression 82.

**Figure 3:**
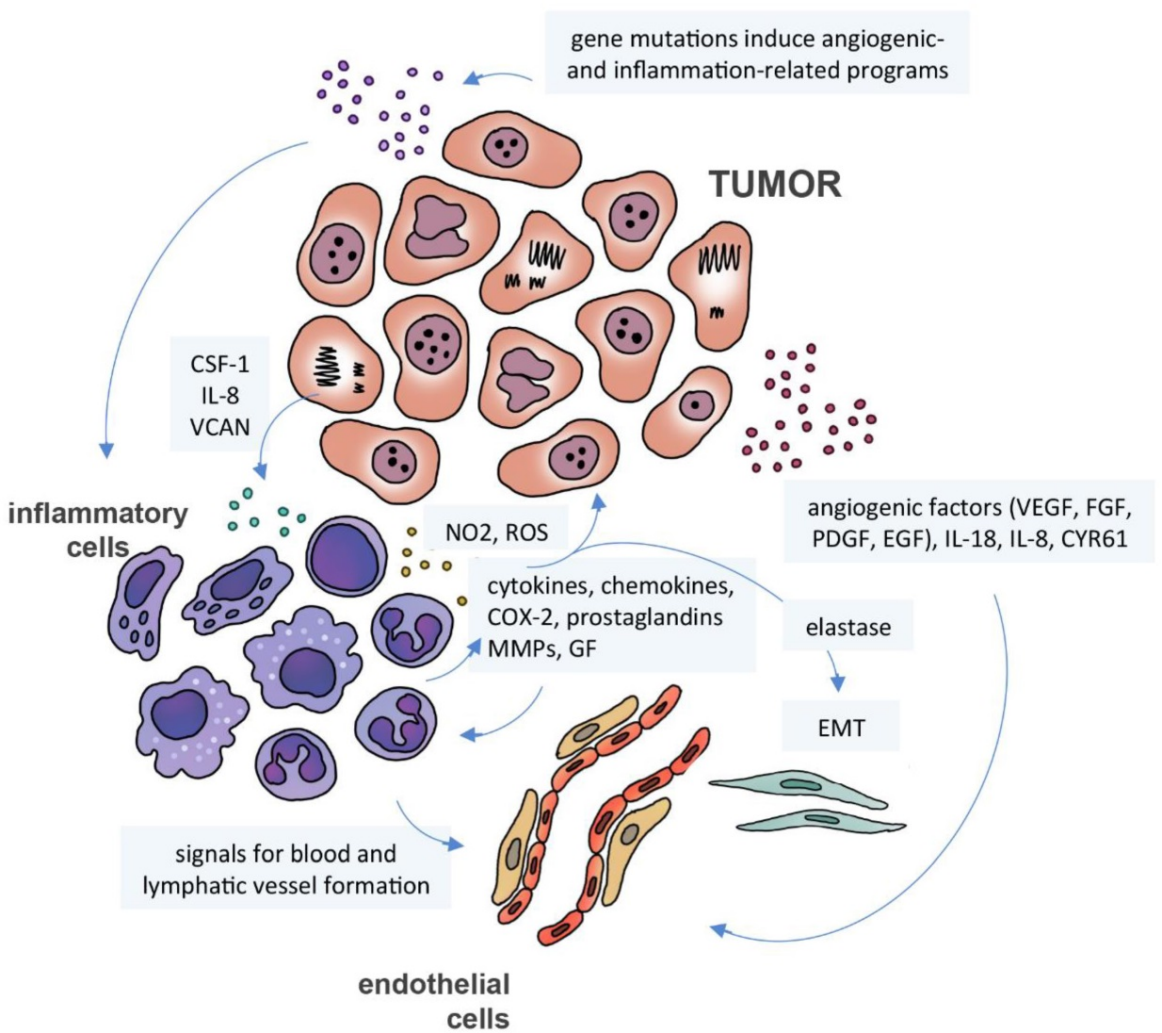
** Cancer secretome, inflammation and angiogenesis.** Gene mutations induce the expression of inflammation-related programs in neoplastic cells with activation of several transcription factors. The resulting signaling events induce immune cells to produce inflammatory mediators, including cytokines, chemokines, cyclooxygenase-2 (COX-2) and prostaglandins, which will trigger a cascade of signals in inflammatory, stromal and tumor cells. Neutrophils can release matrix metalloproteinases (MMPs), nitric oxide (NO_2_), reactive oxygen species (ROS) and elastase, promoting cell proliferation and epithelial-mesenchymal transition (EMT). Neoplastic and immune cells can also secrete pro-angiogenic factors, including growth factors (VEGF, FGF, PDGF, EGF, HDGF), interleukins, cytokines and chemokines (IL-8, IL-18) and cysteine-rich angiogenic inducer CYR61.

According to Mantovani et al 80, the inflammation-cancer relationship may be interpreted as having two pathways: the extrinsic pathway, initiated by inflammation caused by external agents, and the intrinsic pathway initiated by mutations or epigenetic changes in oncogenes and tumor suppressors, which induce the expression of inflammation-related programs. These two pathways activate transcription factors, such as NF-kB, signal transducer and activator of transcription 3 (STAT3) and hypoxia-inducible factor 1-alpha (HIF1-alpha). The resulting signaling events activate leukocytes to produce inflammatory mediators, including cytokines, chemokines, cyclooxygenase-2 (COX-2) and prostaglandins, which will trigger a cascade of similar signals in another inflammatory, stromal and tumor cells. The result is a broad inflammatory signature in the tumor microenvironment with consequent cell proliferation, transition of epithelial to mesenchymal cells, increase in blood and lymphatic vessel formation and inhibition of immune response 90 (**Figure 3**).

Tumor-associated macrophages (TAMs), mast cells and T cells are important components of the inflamed tumor microenvironment. Macrophages are differentiated from monocytes recruited by tumor-derived chemokines and, depending on the activating signals, they exhibit pro- or anti-inflammatory effects, promoting or suppressing tumor activity. Macrophage referred to as M1 has a cytotoxic characteristic and can induce responses of helper T cells to pathogens. M2 macrophages secrete growth and angiogenic factors, release metalloproteases for wound healing and decrease inflammatory responses. 90. Through the course of tumor initiation, macrophages have a central role in the immune response caused by chronic infection or irritation, producing cytokines that recruit other cells of the immune system. After this phase, the macrophages display a M2 phenotype that stimulates the neoplastic progression 91. There are evidences that this second type of action occurs in response to macrophage colony-stimulating factor 1 (CSF-1) and interleukins present in the tumor microenvironment 92, with consequent production of growth factors and metalloproteases by macrophages, invasion and metastasis 93.

The spread of metastases to specific anatomical sites also appears to be partly mediated by macrophages. Primary tumors secrete exosomes or soluble factors that attract macrophages to pre-metastatic niche and induce their reprogramming, for example by transferring oncoproteins through exosomes. The acquisition of a pro-vasculogenic phenotype boosts the development of a suitable microenvironment for tumor progression 94, 95.

The discrepancy in the signals sent by tumor cells for macrophage activation could explain the contradictory prognosis observed in different tumors. For example, the presence of macrophages has been associated with reduced survival in breast carcinoma 96, while it is associated with favorable prognosis in colorectal cancer 97. Analysis of proteins secreted by carcinoma cells that could influence macrophage phenotype identified the proteoglycan versican (VCAN) in colon carcinoma cell lines. Although the presence of stromal VCAN has not been associated with increased survival in colorectal cancer, its expression by epithelial cells in the periphery of the tumor was correlated with an improved prognosis suggesting that this factor is secreted by cancer cells and can participate in monocyte/macrophage differentiation 93.

In addition to macrophages, other immune cells that modulate inflammatory responses play an important role in tumor progression. Among them are the neutrophils, which similarly to the macrophages may have both pro-tumoral and anti-tumoral effects 98. Neutrophils are attracted in response to interleukin (IL-8) released by tumor cells and secrete nitric oxide, reactive oxygen species and matrix metalloproteinases 99. Depending on the context, neutrophils can release elastase promoting cell proliferation 100 and epithelial-mesenchymal transition 101, or modulate tumor cell lysis via T-lymphocytes 102.

Dendritic cells (DC) also play an important role in the regulation of inflammation, mainly due to their ability to secrete different cytokines and chemokines. DCs are able to initiate, expand and regulate the immune response. Although DCs may contribute to inhibit tumor development, neoplastic cells, in turn, can exploit DC to evade immunity 103. The DC secretome profile is less rich than the cytoplasmic proteome, but has been showed to be more specific and sensitive to functional changes, indicating that it could be an interesting source of potential biomarkers involved in the regulation of inflammatory processes 104.

Recently, Seehawer and collaborators 105 showed that necroptosis, a pro-inflammatory type of cell death 106, is associated with a specific cytokine expression profile that apparently induce epigenetic alterations and affect the behavior of neighbor cells, similarly to what was observed by Ohanna et al (2011) 74 in senescent cells. The important observation of Seehawer et al. was that necropsis and apoptosis influence the fate of cells and the resultant cancer subtype. Therefore, inflammatory processes, no matter if triggered by microorganism infection or a type of cell death taking place in the microenvironment, are important contributors to tumorigenesis and even to cancer subtype specification.

## Secretome Components and Angiogenesis

Angiogenesis is defined as the formation of new vessels from a pre-existing vascular network. The process is driven by the demand for oxygen, nutrients, and requires interactions between different cell types, the extracellular matrix and several cytokines and growth factors. Although fundamental in development, tissue maintenance and survival, angiogenesis plays a central role in tumor progression, invasion and metastasis 107.

Several conditions influence angiogenesis, including metabolic stress (hypoxia, acidosis, hypoglycemia), mechanical stress (compression generated by high cell proliferation), inflammatory responses (tumor-infiltrating immune cells) and genetic mutations (oncogene activation or inhibition of tumor suppressor genes) 108. The major effectors of this process are regulators of growth factors, such as HIF1-alpha that drives vascular endothelial growth factor (VEGF) expression, activators of proliferation and migration of endothelial cells, such as growth factors that bind to the tyrosine kinase receptors (VEGF, FGF, PDGF, EGF), and lysophosphatidic acid (LPA) that interacts with G protein-coupled transmembrane receptors 109, 110. Among the inhibitors are thrombospondin-1 and statins (angiostatin, endostatin, canstatin and tunstatina) 109. These pro- and anti-angiogenic factors may be released by tumor cells or derived from the tumor microenvironment 109.

Zhong and collaborators 111 studied the secretome of stromal cells during angiogenesis in a co-culture with lung cancer cells. They observed that the co-culturing promoted a secretome that increased migration of stromal cell, triggered endothelial tube formation, and induced cell proliferation. Secreted proteins included growth factors, interleukins, cytokines and chemokines, such as VEGF, IL-18, tumor necrosis factor (TNF-alpha), cytokine receptor common subunit beta (GM-CSF) and C-C motif chemokine 2 (MCP-1). Other studies have shown the involvement of additional secreted proteins in angiogenesis, such as IL-8 released by glioblastoma cells 112, cysteine-rich angiogenic inducer CYR61 113, 114 and hepatoma-derived growth factor (HDGF) 115 released by bone marrow stem cells and glioblastoma cells, respectively (**Figure 3**).

As mentioned before, tumor cells secrete not only soluble factors, but also extracellular vesicles that promote migration, invasion, tube formation, and neovascularization *in vivo* 116. Choi and collaborators 117 performed a comparative proteomic analysis of EVs and secretome in primary and metastatic colorectal cancer and detected many differences. The authors observed that EVs are enriched in plasma membrane, cytoskeletal and endosomal proteins, whereas secretome has proteins from extracellular matrix and extracellular region. The antigen CD276 (or B7-H3), which is an immunoregulator and cell surface tumor endothelial marker, showed a higher expression in EVs from metastatic cells. CD276 was already reported overexpressed during pathological but not physiological angiogenesis, as well as in tumor endothelial cells and in neoplastic cells 118, and positively correlated with microvessel density 119. Other studies showed similar results, including exosomes released from glioma cells in hypoxia, a condition that is a potent inducer of angiogenesis *in vivo* and *ex vivo* 120. Therefore, CD276 appear to be a promising target for antiangiogenic therapies.

Another potential antiangiogenic target is RalA-binding protein 1 (or RalBP1), a multifunctional protein that contains binding sites for signaling effectors and is expressed in many human tissues and overexpressed in tumors. RalBP1 modulates endothelial cell proliferation and migration by regulating the secretion of the angiogenic factor VEGF and the transcriptional activity of HIF-1 by the tumor 121, 122.

Angiogenic inhibitors targeting VEGF have demonstrated therapeutic efficacy in multiple human cancers. However, the benefits of these therapies are, at best, temporary and are followed by resistance in most patients 123. One explanation for this outcome is the involvement of alternate proangiogenic mediators 124, such as fibroblast growth factor 2 (FGF-2), IL-8, placenta growth factor (PIGF) and pleiotrophin 125.

As previously mentioned, nonsteroidal anti-inflammatory drugs have been found to be effective in preventing cancer. Their main mechanisms of action are pro-apoptotic and anti-angiogenic inhibiting malignant transformation and suppressing proliferation 126. One example is Sulindac, which is used to prevent colorectal cancer and reduces the secreted modulators of apoptosis and angiogenesis such as IL-8 127.

## Approaches for Cancer Secretome Studies

Secretome provides important information relevant to understanding the biology and behavior of cancer. Proteins of the cancer secretome have key roles in many biological processes and can be used in earlier diagnosis, disease monitoring as well as in therapy efficacy evaluation 128. The approaches for secretome studies are dependent on the target and the sample type, and can vary widely, each of them with advantages and limitations. The two main sources of cancer secretome are biological fluids and cancer cell line supernatants. Biofluids, which directly reflect the conditions *in vivo*, and conditioned medium obtained from cell culture, allow to investigate nucleic acids, proteins, metabolites, lipids and carbohydrates by different methods of detection.

### Biofluids

Serum and plasma are the most widely used biological fluids for clinical diagnosis, mainly due to their non-invasive characteristic of sample collection and because they represent a reservoir of molecules released by the tissues. Lin et al. 129 summarized the data from secretome studies using patient serum, plasma or tissue samples in breast, colorectal, gastric, liver, lung and prostate cancers, to provide a clinical validation of potential biomarkers and to determine their functional and diagnostic values. The authors emphasized that the characterization of serum/plasma biomarkers is not simple due to variations in concentration at relatively low levels, pre-analytical sequestration/degradation and clinical factors. In addition, blood contains multiple molecules secreted by different organs, as well as highly abundant proteins such as albumin and immunoglobulins that limits the detection of low abundance proteins 129.

Several biofluids other than serum and plasma have also been analyzed, such as saliva 130, the interstitial fluid of tissues (IF) 131, nipple aspirate fluid 132, pleural effusion 133, bile, pancreatic and digestive juices^134^, and ascites fluid^135^,. They are promising sources of biomarkers in a less complex matrix when compared to plasma or serum. Haslene-Hox and collaborators 136 reviewed several techniques for the isolation of interstitial fluids, and observed a great variation in their composition, which seems to be dependent on the method of isolation. The authors also found that tumor interstitial fluid is hypoxic and acidic compared with subcutaneous fluid and plasma. In addition, concentrations gradients may indicate whether fluid components are locally synthesized or derived from plasma. However, an important question is the origin of many proteins, from extracellular space or from cellular leakage.

Deciphering the overall biofluid composition and dynamics remains a major challenge. Despite the difficulties 2, 137, some methods have been applied for analyzing the secretome* in vivo*, such as microdialysis and ultrafiltration. The microdialysis method relies on the passive diffusion of substances across a semi-permeable membrane driven by a concentration gradient 18, 137. However, peptide and protein recovery are still challenging because the dialysis efficiency is very low (~1%) as a consequence, for example, of the number of pores within the membrane. Therefore, the dialysate data may not correspond to the molecular size distribution and concentration of the biofluid 136. The ultrafiltration technique also uses semi-permeable membranes to separate substances, but has some advantages when compared to microdialysis. The ultrafiltration uses negative pressure to collect a fluid and facilitates long-term and dynamic sampling collection, without changing the original concentration of the biofluid 18, 137.

### Conditioned Medium

Analyses of cell-conditioned medium (CM) have been widely performed to identify secreted proteins and cancer biomarkers, although cultures of neoplastic cells do not reconstitute tumor tissue and its microenvironment 3, 17. Tumor tissues may also exhibit dissimilar levels or even absence/presence of metabolites and proteins compared with standard culture medium 61. Nevertheless, the secretome released in the culture medium is less complex than biofluids and facilitates the identification of low abundance proteins. It also comprises a good option to assess the concentration of released molecules and provides information about biological processes 2, 18, 129.

The discovery of biomarkers in CM also has its technological challenges similar to what is observed in biofluids. For example, secreted proteins in CM are often masked by proteins present in supplements added to the culture medium. To circumvent this limitation, cells are usually incubated in serum-free medium experiments to reduce interference from serum components. Nevertheless, the serum deprivation may result in altered metabolism, decreased proliferation, increased cell death and protein release by autolysis, as well as in abnormal protein synthesis and secretion 129. To minimize the effect of in vitro conditions, isotope-labeled amino acids can be introduced into the culture medium enabling the differentiation between serum proteins and secreted proteins 129, 138, 139. Furthermore, the optimization of incubation time and cellular confluence appears to reduce contamination with intracellular proteins 140.

Using a dataset from five cell lines, Méndez and Villanueva 141 showed that many proteins in CM were both extra and intracellular according to Gene Ontology classification. However, several of them were solely intracellular, meaning that they are indeed derived from cell death and apoptosis under serum starvation or may be secreted by non-classical pathways, such as exosomes.

To validate Méndez and Villanueva results, we assembled data retrieved from the Human Cancer Secretome Database (HCSD) 142 (http://www.cancersecretome.org) and a subset of proteins identified after a literature search in PubMed database (**Table S1** and **Table S2**, respectively). Genes encoding for these proteins were included in the dataset as follows: (a) 1,835 non-redundant genes (log2 fold change values below 1 or above 1) from 9 high-throughput studies available in HCSD on seven cancer types (colorectal, esophageal, gastric, glioblastoma, head and neck, lung, pancreatic cancers) using 35 human neoplastic and 3 human non-neoplastic cell lines, and (b) 37 non-redundant genes from the literature search (references in **Table S1** and **Table S2**, respectively). Following manual curation and re-evaluation of redundancy, a final list of 1,776 genes was obtained. A gene ontology and pathway search using DAVID tools 143 showed a total of 1,767 DAVID identifiers. The most overrepresented cellular component category was extracellular exosome (Bonferroni corrected p-values=1.35^-292^), followed by cytosol, focal adhesion, extracellular matrix and space, cell-cell adherens junction, membrane and cytoplasm categories (**Table S3**, sheet 1 - cellular component), suggesting that many proteins may be located in both extra and intracellular compartments, or are derived from cell death/apoptosis processes, as shown by Méndez and Villanueva 141. DAVID analysis also identified significantly enriched biological processes and pathways related to cell-cell adhesion, extracellular matrix organization, ECM-receptor interaction, and focal adhesion, although processes and pathways associated with intracellular proteins have also been observed (**Table S3**, sheet 2 - biological processes, sheet 3 - pathways). In addition, more than 290 annotation clusters were obtained, 13 of them with enrichment scores>5.0 (**Table S4).** The results showed overrepresented clusters related to cell-cell adhesion, translation, protein metabolism, and secretion, again showing processes relevant to crosstalk between cells and to intracellular pathways.

The results address a question as to *why many intracellular proteins are present in the secretome.* Are they only a consequence of cell culture conditions or may be important in modulating cell communication and local or distant biological processes? As commented before, senescence, necropsis and apoptosis may not be an end in itself, but generate signals that stimulate cells in the neighborhood or in different tissues and organs. More studies are therefore necessary to sort the wheat from the chaff and answer these questions.

### Complementary Methodologies

Several complementary methodologies have been applied in secretome studies of both biofluids and CM, such as microarrays and large-scale cDNA sequencing to analyze transcripts present in exosomes 144, liquid chromatography-mass spectrometry and antibodies for targeted capture and detection of secreted proteins 17, and mass spectrometry and nuclear magnetic resonance to identify metabolites 145. Using these methodologies, many data on the cancer secretome have been generated and validated in a high number of samples and/or patients. For example, the study performed by Aleckovic and collaborators, using a quantitative mass spectrometry-based proteomics approach, identified hundreds of breast cancer- and melanoma-derived proteins secreted from lung metastatic cells. The authors observed that Nidogen-1 (NID-1), a basement membrane glycoprotein with binding sites for other ECM molecules, promote lung metastasis of breast cancer and melanoma and induces prometastatic characteristics, and its expression is correlated with poor prognosis 146. Likewise, Pierredon et al. 147 while investigating the secretome of ovarian cancer by mass spectrometry identified gelsolin, a protein involved in cell motility, phagocytosis, apoptosis, platelet formation, and activation, with significantly lower expression levels in cancer cells and in the sera of ovarian cancer patients.

Data on cancer secretome have also been organized in database formats. For example, The Human Cancer Secretome Database 142, mentioned above, contains 7000 nonredundant human proteins collected from up to 35 high-throughput studies on 17 cancer types. The repository of cancer-associated peptidomes in human biofluids (CancerPDF) 148 (http://crdd.osdd.net/raghava/cancerpdf/) covers nearly 30 types of human cancers and contains 14,367 experimentally validated peptides. The 2004 release of the web-based Secreted Protein Database (SPD) 149 (https://www.hsls.pitt.edu/obrc/index.php?page=URL1104935692) contains a total of 18152 secreted proteins in Human, Mouse and Rat proteomes. The Plasma Proteome Database 150 (http://www.plasmaproteomedatabase.org/) hosts qualitative and quantitative information on proteins from plasma and serum and the ones reported in extracellular vesicle isolated from plasma. The PeptideAtlas 151 (http://www.peptideatlas.org/) provides data on human plasma and urine and The Human Metabolome Database 152 (http://www.hmdb.ca/) contains information about metabolites in different biospecimens. Unfortunately, not all databases are regularly updated.

Although with restrictions related to false positive and false negative assignments 144, bioinformatic tools have contributed *significantly* not only to organize information but also to characterize the secretome. For example, the SignalP software 153 (http://www.cbs.dtu.dk/services/SignalP/) predicts the presence of signal peptide cleavage sites located at the N-terminus of proteins secreted by the classical pathway. Proteins secreted by the non-classical pathway are devoid of a signal peptide and can be predicted by the SecretomeP software 154 (http://www.cbs.dtu.dk/services/SecretomeP/) through the sequence features, such as posttranslational modifications, size, and charge. Clustering of expression profiles and functional analyses of secretome may also reveal signatures of cancer cells 155. In this respect, ExoCarta 156 (www.exocarta.org) and Gene Ontology consortium 157, 158 (www.geneontology.org) databases facilitate the access to information about content of exosomes, and cell location, molecular function and biological process of proteins, and help to distinguish between the true secreted proteins and intracellular contaminants.

## Conclusion

The secretome is a rich reservoir for cancer biomarkers. Decoding the secretome in the tumor microenvironment and understanding how it enables the exchange of signals between tumor cells and stromal cells could help to elucidate the biochemical pathways involved in tumorigenesis, from early to advanced stages, and to identify potential biomarkers for the development of new diagnostic and prognostic tools, therapies and prediction of therapeutic responses.

## Supplementary Material

Table S1.

Table S2.

Table S3.

Table S4.
