# Supplementary material for: Cellular Interactions in the Tumor Microenvironment: The Role of Secretome
Source: J Cancer. 2019 Aug 7;10(19):4574–87. doi: 10.7150/jca.21780 (PMC6746126; doi:10.7150/jca.21780)
Supplement: Supplementary file 1 — Table S1. [file jcav10p4574s1.pdf]

Table S1. Genes coding for proteins retrieved from the Human Cancer Secretome Database (HCSD) (www.cancersecretome.org). Studies available in HCSD (see reference PMID) were conducted using label-based methods in seven cancer types (colorectal, esophageal, gastric, glioblastoma, head and neck cancer, lung, pancreatic cancers) and 35 neoplastic and 3 non-neoplastic cell lines. Genes were selected when Log2 Fold-Change were > 1 or < -1.

Gene symbol	Focus of study	Cell line	Log2 Fold-Change	Method	Reference PMID
ACTA1	pancreatic cancer	neoplastic (HPDE, Panc1)	2	SILAC	16215274
ACTB	pancreatic cancer	neoplastic (HPDE, Panc1)	1.26	SILAC	16215274
ACTN4	pancreatic cancer	neoplastic (HPDE, Panc1)	1.96	SILAC	16215274
AKAP6	pancreatic cancer	neoplastic (HPDE, Panc1)	-4.32	SILAC	16215274
ALB	pancreatic cancer	neoplastic (HPDE, Panc1)	-4.32	SILAC	16215274
ANPEP	pancreatic cancer	neoplastic (HPDE, Panc1)	4.32	SILAC	16215274
ANXA2	pancreatic cancer	neoplastic (HPDE, Panc1)	-1.05	SILAC	16215274
ANXA3	pancreatic cancer	neoplastic (HPDE, Panc1)	-2.55	SILAC	16215274
ANXA5	pancreatic cancer	neoplastic (HPDE, Panc1)	-2.55	SILAC	16215274
APLP2	pancreatic cancer	neoplastic (HPDE, Panc1)	1.26	SILAC	16215274
APOE	pancreatic cancer	neoplastic (HPDE, Panc1)	4.32	SILAC	16215274
APP	pancreatic cancer	neoplastic (HPDE, Panc1)	1.58	SILAC	16215274
ATP5B	pancreatic cancer	neoplastic (HPDE, Panc1)	-2.18	SILAC	16215274
AZGP1	pancreatic cancer	neoplastic (HPDE, Panc1)	-4.32	SILAC	16215274
BTD	pancreatic cancer	neoplastic (HPDE, Panc1)	2.16	SILAC	16215274
C17orf25	pancreatic cancer	neoplastic (HPDE, Panc1)	1	SILAC	16215274
C6orf82	pancreatic cancer	neoplastic (HPDE, Panc1)	1.32	SILAC	16215274
C7orf24	pancreatic cancer	neoplastic (HPDE, Panc1)	-2.55	SILAC	16215274
Cab45	pancreatic cancer	neoplastic (HPDE, Panc1)	4.32	SILAC	16215274
CASP14	pancreatic cancer	neoplastic (HPDE, Panc1)	-4.32	SILAC	16215274
CCL2	pancreatic cancer	neoplastic (HPDE, Panc1)	4.32	SILAC	16215274
CD109	pancreatic cancer	neoplastic (HPDE, Panc1)	1.07	SILAC	16215274
CD9	pancreatic cancer	neoplastic (HPDE, Panc1)	3	SILAC	16215274
CLIC1	pancreatic cancer	neoplastic (HPDE, Panc1)	2.26	SILAC	16215274
CPA4	pancreatic cancer	neoplastic (HPDE, Panc1)	-4.32	SILAC	16215274
CRABP2	pancreatic cancer	neoplastic (HPDE, Panc1)	2.45	SILAC	16215274
CSF1	pancreatic cancer	neoplastic (HPDE, Panc1)	1.20	SILAC	16215274
CST3	pancreatic cancer	neoplastic (HPDE, Panc1)	-2.05	SILAC	16215274
CST6	pancreatic cancer	neoplastic (HPDE, Panc1)	-4.32	SILAC	16215274
CSTA	pancreatic cancer	neoplastic (HPDE, Panc1)	-4.32	SILAC	16215274
CTBS	pancreatic cancer	neoplastic (HPDE, Panc1)	2.23	SILAC	16215274
CTGF	pancreatic cancer	neoplastic (HPDE, Panc1)	-4.32	SILAC	16215274
CTSB	pancreatic cancer	neoplastic (HPDE, Panc1)	1.43	SILAC	16215274
CTSD	pancreatic cancer	neoplastic (HPDE, Panc1)	2.67	SILAC	16215274
CTSL	pancreatic cancer	neoplastic (HPDE, Panc1)	-4.32	SILAC	16215274
CYR61	pancreatic cancer	neoplastic (HPDE, Panc1)	-1	SILAC	16215274
DCD	pancreatic cancer	neoplastic (HPDE, Panc1)	-4.32	SILAC	16215274

DDAH2	pancreatic cancer	neoplastic (HPDE, Panc1)	4.32	SILAC	16215274
DNASE2	pancreatic cancer	neoplastic (HPDE, Panc1)	2.23	SILAC	16215274
DSC1	pancreatic cancer	neoplastic (HPDE, Panc1)	-4.32	SILAC	16215274
ENO1	pancreatic cancer	neoplastic (HPDE, Panc1)	-2.25	SILAC	16215274
FAM3C	pancreatic cancer	neoplastic (HPDE, Panc1)	2.07	SILAC	16215274
FN1	pancreatic cancer	neoplastic (HPDE, Panc1)	-4.32	SILAC	16215274
FUCA1	pancreatic cancer	neoplastic (HPDE, Panc1)	-2.18	SILAC	16215274
GAPD	pancreatic cancer	neoplastic (HPDE, Panc1)	-2.05	SILAC	16215274
GDI1	pancreatic cancer	neoplastic (HPDE, Panc1)	1.13	SILAC	16215274
GNAI2	pancreatic cancer	neoplastic (HPDE, Panc1)	2.43	SILAC	16215274
GOT1	pancreatic cancer	neoplastic (HPDE, Panc1)	1.13	SILAC	16215274
GOT2	pancreatic cancer	neoplastic (HPDE, Panc1)	-1.25	SILAC	16215274
H2AFJ	pancreatic cancer	neoplastic (HPDE, Panc1)	3	SILAC	16215274
HAPLN3	pancreatic cancer	neoplastic (HPDE, Panc1)	4.32	SILAC	16215274
HIST1H2BG	pancreatic cancer	neoplastic (HPDE, Panc1)	4.32	SILAC	16215274
HIST1H4A	pancreatic cancer	neoplastic (HPDE, Panc1)	2.72	SILAC	16215274
HSPG2	pancreatic cancer	neoplastic (HPDE, Panc1)	2.45	SILAC	16215274
IGFBP7	pancreatic cancer	neoplastic (HPDE, Panc1)	-4.32	SILAC	16215274
IL1F9	pancreatic cancer	neoplastic (HPDE, Panc1)	-4.32	SILAC	16215274
INS	pancreatic cancer	neoplastic (HPDE, Panc1)	-4.32	SILAC	16215274
ITGB1	pancreatic cancer	neoplastic (HPDE, Panc1)	2.16	SILAC	16215274
K-ALPHA-1	pancreatic cancer	neoplastic (HPDE, Panc1)	1.37	SILAC	16215274
KIAA1627	pancreatic cancer	neoplastic (HPDE, Panc1)	2.16	SILAC	16215274
KRT1	pancreatic cancer	neoplastic (HPDE, Panc1)	-4.32	SILAC	16215274
KRT10	pancreatic cancer	neoplastic (HPDE, Panc1)	-4.32	SILAC	16215274
KRT14	pancreatic cancer	neoplastic (HPDE, Panc1)	-4.32	SILAC	16215274
KRT19	pancreatic cancer	neoplastic (HPDE, Panc1)	-4.32	SILAC	16215274
KRT8	pancreatic cancer	neoplastic (HPDE, Panc1)	4.32	SILAC	16215274
KRT9	pancreatic cancer	neoplastic (HPDE, Panc1)	-4.32	SILAC	16215274
KRTHA1	pancreatic cancer	neoplastic (HPDE, Panc1)	-4.32	SILAC	16215274
KRTHA5	pancreatic cancer	neoplastic (HPDE, Panc1)	-4.32	SILAC	16215274
KRTHB2	pancreatic cancer	neoplastic (HPDE, Panc1)	-4.32	SILAC	16215274
KRTHB3	pancreatic cancer	neoplastic (HPDE, Panc1)	-4.32	SILAC	16215274
KRTHB5	pancreatic cancer	neoplastic (HPDE, Panc1)	-4.32	SILAC	16215274
L1CAM	pancreatic cancer	neoplastic (HPDE, Panc1)	4.32	SILAC	16215274
LCN1	pancreatic cancer	neoplastic (HPDE, Panc1)	-4.32	SILAC	16215274
LGALS3BP	pancreatic cancer	neoplastic (HPDE, Panc1)	2.32	SILAC	16215274
LGMN	pancreatic cancer	neoplastic (HPDE, Panc1)	-4.32	SILAC	16215274
LOC343531	pancreatic cancer	neoplastic (HPDE, Panc1)	4.32	SILAC	16215274
LOC347376	pancreatic cancer	neoplastic (HPDE, Panc1)	2.88	SILAC	16215274
LOXL2	pancreatic cancer	neoplastic (HPDE, Panc1)	4.32	SILAC	16215274
LTF	pancreatic cancer	neoplastic (HPDE, Panc1)	-4.32	SILAC	16215274
LYZ	pancreatic cancer	neoplastic (HPDE, Panc1)	-4.32	SILAC	16215274

MMP9	pancreatic cancer	neoplastic (HPDE, Panc1)	-4.32	SILAC	16215274
MYO18B	pancreatic cancer	neoplastic (HPDE, Panc1)	-4.32	SILAC	16215274
NIPSNAP1	pancreatic cancer	neoplastic (HPDE, Panc1)	-4.32	SILAC	16215274
NPC2	pancreatic cancer	neoplastic (HPDE, Panc1)	-1.32	SILAC	16215274
NUCB1	pancreatic cancer	neoplastic (HPDE, Panc1)	4.32	SILAC	16215274
P4HB	pancreatic cancer	neoplastic (HPDE, Panc1)	-1.05	SILAC	16215274
PFN1	pancreatic cancer	neoplastic (HPDE, Panc1)	-1.73	SILAC	16215274
PIP	pancreatic cancer	neoplastic (HPDE, Panc1)	-4.32	SILAC	16215274
PNPO	pancreatic cancer	neoplastic (HPDE, Panc1)	4.32	SILAC	16215274
PPIA	pancreatic cancer	neoplastic (HPDE, Panc1)	1.43	SILAC	16215274
PPIC	pancreatic cancer	neoplastic (HPDE, Panc1)	-1.47	SILAC	16215274
PRDX1	pancreatic cancer	neoplastic (HPDE, Panc1)	-1	SILAC	16215274
PSAP	pancreatic cancer	neoplastic (HPDE, Panc1)	3.32	SILAC	16215274
PSAT1	pancreatic cancer	neoplastic (HPDE, Panc1)	1.58	SILAC	16215274
PTK7	pancreatic cancer	neoplastic (HPDE, Panc1)	2.51	SILAC	16215274
RAC2	pancreatic cancer	neoplastic (HPDE, Panc1)	1.80	SILAC	16215274
RPS28	pancreatic cancer	neoplastic (HPDE, Panc1)	2.53	SILAC	16215274
S100A2	pancreatic cancer	neoplastic (HPDE, Panc1)	-4.32	SILAC	16215274
S100A7	pancreatic cancer	neoplastic (HPDE, Panc1)	-4.32	SILAC	16215274
S100A8	pancreatic cancer	neoplastic (HPDE, Panc1)	-4.32	SILAC	16215274
SCGB1D2	pancreatic cancer	neoplastic (HPDE, Panc1)	-4.32	SILAC	16215274
SDC1	pancreatic cancer	neoplastic (HPDE, Panc1)	-1.21	SILAC	16215274
SDC4	pancreatic cancer	neoplastic (HPDE, Panc1)	4.32	SILAC	16215274
SEMA4B	pancreatic cancer	neoplastic (HPDE, Panc1)	1.37	SILAC	16215274
SERPINB13	pancreatic cancer	neoplastic (HPDE, Panc1)	-4.32	SILAC	16215274
SERPINB2	pancreatic cancer	neoplastic (HPDE, Panc1)	-4.32	SILAC	16215274
SERPINB7	pancreatic cancer	neoplastic (HPDE, Panc1)	-2	SILAC	16215274
SLPI	pancreatic cancer	neoplastic (HPDE, Panc1)	-4.32	SILAC	16215274
SYT13	pancreatic cancer	neoplastic (HPDE, Panc1)	-2.83	SILAC	16215274
TF	pancreatic cancer	neoplastic (HPDE, Panc1)	-4.32	SILAC	16215274
TGFBI	pancreatic cancer	neoplastic (HPDE, Panc1)	-4.32	SILAC	16215274
TGM3	pancreatic cancer	neoplastic (HPDE, Panc1)	-4.32	SILAC	16215274
THBS1	pancreatic cancer	neoplastic (HPDE, Panc1)	-4.05	SILAC	16215274
TKT	pancreatic cancer	neoplastic (HPDE, Panc1)	2.07	SILAC	16215274
TPI1	pancreatic cancer	neoplastic (HPDE, Panc1)	1.26	SILAC	16215274
TPM3	pancreatic cancer	neoplastic (HPDE, Panc1)	-1.15	SILAC	16215274
TXNRD1	pancreatic cancer	neoplastic (HPDE, Panc1)	1	SILAC	16215274
VCL	pancreatic cancer	neoplastic (HPDE, Panc1)	2.16	SILAC	16215274
VCP	pancreatic cancer	neoplastic (HPDE, Panc1)	1	SILAC	16215274
VIM	pancreatic cancer	neoplastic (HPDE, Panc1)	-4.32	SILAC	16215274
YWHAG	pancreatic cancer	neoplastic (HPDE, Panc1)	1.26	SILAC	16215274
YWHAQ	pancreatic cancer	neoplastic (HPDE, Panc1)	2.56	SILAC	16215274
YWHAZ	pancreatic cancer	neoplastic (HPDE, Panc1)	1.80	SILAC	16215274

AASDHPPT	esophageal cancer	neoplastic (TE1, TE2, TE5, TE8, TE10, TE11, TE15) and non-neoplastic (Het-1A)	-1.12	SILAC	20686364
ACIN1	esophageal cancer	neoplastic (TE1, TE2, TE5, TE8, TE10, TE11, TE15) and non-neoplastic (Het-1A)	1.07	SILAC	20686364
ADH5	esophageal cancer	neoplastic (TE1, TE2, TE5, TE8, TE10, TE11, TE15) and non-neoplastic (Het-1A)	-1.78	SILAC	20686364
AIM1	esophageal cancer	neoplastic (TE1, TE2, TE5, TE8, TE10, TE11, TE15) and non-neoplastic (Het-1A)	-1.68	SILAC	20686364
AK3	esophageal cancer	neoplastic (TE1, TE2, TE5, TE8, TE10, TE11, TE15) and non-neoplastic (Het-1A)	-1	SILAC	20686364
AKAP13	esophageal cancer	neoplastic (TE1, TE2, TE5, TE8, TE10, TE11, TE15) and non-neoplastic (Het-1A)	-1.05	SILAC	20686364
ALDH1A1	esophageal cancer	neoplastic (TE1, TE2, TE5, TE8, TE10, TE11, TE15) and non-neoplastic (Het-1A)	2.59	SILAC	20686364
ALDOC	esophageal cancer	neoplastic (TE1, TE2, TE5, TE8, TE10, TE11, TE15) and non-neoplastic (Het-1A)	3.57	SILAC	20686364
AMDHD2	esophageal cancer	neoplastic (TE1, TE2, TE5, TE8, TE10, TE11, TE15) and non-neoplastic (Het-1A)	-1.28	SILAC	20686364
ANP32A	esophageal cancer	neoplastic (TE1, TE2, TE5, TE8, TE10, TE11, TE15) and non-neoplastic (Het-1A)	2.49	SILAC	20686364
API5	esophageal cancer	neoplastic (TE1, TE2, TE5, TE8, TE10, TE11, TE15) and non-neoplastic (Het-1A)	2.33	SILAC	20686364
APOD	esophageal cancer	neoplastic (TE1, TE2, TE5, TE8, TE10, TE11, TE15) and non-neoplastic (Het-1A)	-1.28	SILAC	20686364
ARPC4	esophageal cancer	neoplastic (TE1, TE2, TE5, TE8, TE10, TE11, TE15) and non-neoplastic (Het-1A)	2.96	SILAC	20686364
B2M	esophageal cancer	neoplastic (TE1, TE2, TE5, TE8, TE10, TE11, TE15) and non-neoplastic (Het-1A)	3.56	SILAC	20686364
BCAP31	esophageal cancer	neoplastic (TE1, TE2, TE5, TE8, TE10, TE11, TE15) and non-neoplastic (Het-1A)	-1.73	SILAC	20686364
C19orf53	esophageal cancer	neoplastic (TE1, TE2, TE5, TE8, TE10, TE11, TE15) and non-neoplastic (Het-1A)	-1.35	SILAC	20686364
C3	esophageal cancer	neoplastic (TE1, TE2, TE5, TE8, TE10, TE11, TE15) and non-neoplastic (Het-1A)	1.95	SILAC	20686364
C4orf44	esophageal cancer	neoplastic (TE1, TE2, TE5, TE8, TE10, TE11, TE15) and non-neoplastic (Het-1A)	-3.32	SILAC	20686364
CAV1	esophageal cancer	neoplastic (TE1, TE2, TE5, TE8, TE10, TE11, TE15) and non-neoplastic (Het-1A)	2.14	SILAC	20686364
CBR3	esophageal cancer	neoplastic (TE1, TE2, TE5, TE8, TE10, TE11, TE15) and non-neoplastic (Het-1A)	-1.25	SILAC	20686364
CDC42	esophageal cancer	neoplastic (TE1, TE2, TE5, TE8, TE10, TE11, TE15) and non-neoplastic (Het-1A)	-1.28	SILAC	20686364
CDH13	esophageal cancer	neoplastic (TE1, TE2, TE5, TE8, TE10, TE11, TE15) and non-neoplastic (Het-1A)	-1.88	SILAC	20686364
CHGA	esophageal cancer	neoplastic (TE1, TE2, TE5, TE8, TE10, TE11, TE15) and non-neoplastic (Het-1A)	-1.94	SILAC	20686364
CHMP4B	esophageal cancer	neoplastic (TE1, TE2, TE5, TE8, TE10, TE11, TE15) and non-neoplastic (Het-1A)	1.35	SILAC	20686364
CKAP5	esophageal cancer	neoplastic (TE1, TE2, TE5, TE8, TE10, TE11, TE15) and non-neoplastic (Het-1A)	1.05	SILAC	20686364
CLSTN1	esophageal cancer	neoplastic (TE1, TE2, TE5, TE8, TE10, TE11, TE15) and non-neoplastic (Het-1A)	4.05	SILAC	20686364
CNPY2	esophageal cancer	neoplastic (TE1, TE2, TE5, TE8, TE10, TE11, TE15) and non-neoplastic (Het-1A)	1.58	SILAC	20686364
CORO1A	esophageal cancer	neoplastic (TE1, TE2, TE5, TE8, TE10, TE11, TE15) and non-neoplastic (Het-1A)	-1.47	SILAC	20686364
CRKL	esophageal cancer	neoplastic (TE1, TE2, TE5, TE8, TE10, TE11, TE15) and non-neoplastic (Het-1A)	-1.25	SILAC	20686364
CST6	esophageal cancer	neoplastic (TE1, TE2, TE5, TE8, TE10, TE11, TE15) and non-neoplastic (Het-1A)	1.87	SILAC	20686364
CTSH	esophageal cancer	neoplastic (TE1, TE2, TE5, TE8, TE10, TE11, TE15) and non-neoplastic (Het-1A)	2.08	SILAC	20686364
CYCS	esophageal cancer	neoplastic (TE1, TE2, TE5, TE8, TE10, TE11, TE15) and non-neoplastic (Het-1A)	-1.28	SILAC	20686364
DDX39B	esophageal cancer	neoplastic (TE1, TE2, TE5, TE8, TE10, TE11, TE15) and non-neoplastic (Het-1A)	1.91	SILAC	20686364
DDX47	esophageal cancer	neoplastic (TE1, TE2, TE5, TE8, TE10, TE11, TE15) and non-neoplastic (Het-1A)	1.56	SILAC	20686364
DKC1	esophageal cancer	neoplastic (TE1, TE2, TE5, TE8, TE10, TE11, TE15) and non-neoplastic (Het-1A)	2.19	SILAC	20686364
DPYSL3	esophageal cancer	neoplastic (TE1, TE2, TE5, TE8, TE10, TE11, TE15) and non-neoplastic (Het-1A)	-1.43	SILAC	20686364
EEF1A2	esophageal cancer	neoplastic (TE1, TE2, TE5, TE8, TE10, TE11, TE15) and non-neoplastic (Het-1A)	1.63	SILAC	20686364
EEF2	esophageal cancer	neoplastic (TE1, TE2, TE5, TE8, TE10, TE11, TE15) and non-neoplastic (Het-1A)	1	SILAC	20686364
EIF2S3	esophageal cancer	neoplastic (TE1, TE2, TE5, TE8, TE10, TE11, TE15) and non-neoplastic (Het-1A)	1.40	SILAC	20686364
EIF3F	esophageal cancer	neoplastic (TE1, TE2, TE5, TE8, TE10, TE11, TE15) and non-neoplastic (Het-1A)	1.28	SILAC	20686364
EPG5	esophageal cancer	neoplastic (TE1, TE2, TE5, TE8, TE10, TE11, TE15) and non-neoplastic (Het-1A)	-3.32	SILAC	20686364
FAH	esophageal cancer	neoplastic (TE1, TE2, TE5, TE8, TE10, TE11, TE15) and non-neoplastic (Het-1A)	-1.32	SILAC	20686364
FAM83H	esophageal cancer	neoplastic (TE1, TE2, TE5, TE8, TE10, TE11, TE15) and non-neoplastic (Het-1A)	-1.51	SILAC	20686364

FBLIM1	esophageal cancer	neoplastic (TE1, TE2, TE5, TE8, TE10, TE11, TE15) and non-neoplastic (Het-1A)	-1.68	SILAC	20686364
FLAD1	esophageal cancer	neoplastic (TE1, TE2, TE5, TE8, TE10, TE11, TE15) and non-neoplastic (Het-1A)	-1.21	SILAC	20686364
FXR2	esophageal cancer	neoplastic (TE1, TE2, TE5, TE8, TE10, TE11, TE15) and non-neoplastic (Het-1A)	-1.05	SILAC	20686364
G6PD	esophageal cancer	neoplastic (TE1, TE2, TE5, TE8, TE10, TE11, TE15) and non-neoplastic (Het-1A)	-1.05	SILAC	20686364
GABRB2	esophageal cancer	neoplastic (TE1, TE2, TE5, TE8, TE10, TE11, TE15) and non-neoplastic (Het-1A)	-3.32	SILAC	20686364
GBE1	esophageal cancer	neoplastic (TE1, TE2, TE5, TE8, TE10, TE11, TE15) and non-neoplastic (Het-1A)	-1.35	SILAC	20686364
GLOD4	esophageal cancer	neoplastic (TE1, TE2, TE5, TE8, TE10, TE11, TE15) and non-neoplastic (Het-1A)	-1.73	SILAC	20686364
GM2A	esophageal cancer	neoplastic (TE1, TE2, TE5, TE8, TE10, TE11, TE15) and non-neoplastic (Het-1A)	-2.12	SILAC	20686364
GMPS	esophageal cancer	neoplastic (TE1, TE2, TE5, TE8, TE10, TE11, TE15) and non-neoplastic (Het-1A)	1.24	SILAC	20686364
GOT1	esophageal cancer	neoplastic (TE1, TE2, TE5, TE8, TE10, TE11, TE15) and non-neoplastic (Het-1A)	-1.25	SILAC	20686364
GPI	esophageal cancer	neoplastic (TE1, TE2, TE5, TE8, TE10, TE11, TE15) and non-neoplastic (Het-1A)	-1.08	SILAC	20686364
GPX1	esophageal cancer	neoplastic (TE1, TE2, TE5, TE8, TE10, TE11, TE15) and non-neoplastic (Het-1A)	-1.55	SILAC	20686364
GRPEL1	esophageal cancer	neoplastic (TE1, TE2, TE5, TE8, TE10, TE11, TE15) and non-neoplastic (Het-1A)	-1.32	SILAC	20686364
GSTM2	esophageal cancer	neoplastic (TE1, TE2, TE5, TE8, TE10, TE11, TE15) and non-neoplastic (Het-1A)	-2.05	SILAC	20686364
GSTM3	esophageal cancer	neoplastic (TE1, TE2, TE5, TE8, TE10, TE11, TE15) and non-neoplastic (Het-1A)	-1.25	SILAC	20686364
GSTO1	esophageal cancer	neoplastic (TE1, TE2, TE5, TE8, TE10, TE11, TE15) and non-neoplastic (Het-1A)	-1.25	SILAC	20686364
GSTP1	esophageal cancer	neoplastic (TE1, TE2, TE5, TE8, TE10, TE11, TE15) and non-neoplastic (Het-1A)	-1.21	SILAC	20686364
GTF2F1	esophageal cancer	neoplastic (TE1, TE2, TE5, TE8, TE10, TE11, TE15) and non-neoplastic (Het-1A)	1.32	SILAC	20686364
GUK1	esophageal cancer	neoplastic (TE1, TE2, TE5, TE8, TE10, TE11, TE15) and non-neoplastic (Het-1A)	-1.39	SILAC	20686364
HDGFRP3	esophageal cancer	neoplastic (TE1, TE2, TE5, TE8, TE10, TE11, TE15) and non-neoplastic (Het-1A)	1.58	SILAC	20686364
HEBP1	esophageal cancer	neoplastic (TE1, TE2, TE5, TE8, TE10, TE11, TE15) and non-neoplastic (Het-1A)	-1.32	SILAC	20686364
HIST1H2AJ	esophageal cancer	neoplastic (TE1, TE2, TE5, TE8, TE10, TE11, TE15) and non-neoplastic (Het-1A)	1.43	SILAC	20686364
HK1	esophageal cancer	neoplastic (TE1, TE2, TE5, TE8, TE10, TE11, TE15) and non-neoplastic (Het-1A)	4.75	SILAC	20686364
HMGA1	esophageal cancer	neoplastic (TE1, TE2, TE5, TE8, TE10, TE11, TE15) and non-neoplastic (Het-1A)	1.70	SILAC	20686364
HMGB1	esophageal cancer	neoplastic (TE1, TE2, TE5, TE8, TE10, TE11, TE15) and non-neoplastic (Het-1A)	2.01	SILAC	20686364
HMGB3	esophageal cancer	neoplastic (TE1, TE2, TE5, TE8, TE10, TE11, TE15) and non-neoplastic (Het-1A)	1.73	SILAC	20686364
HSP90B1	esophageal cancer	neoplastic (TE1, TE2, TE5, TE8, TE10, TE11, TE15) and non-neoplastic (Het-1A)	2.20	SILAC	20686364
HSPC159	esophageal cancer	neoplastic (TE1, TE2, TE5, TE8, TE10, TE11, TE15) and non-neoplastic (Het-1A)	-1.02	SILAC	20686364
ILF2	esophageal cancer	neoplastic (TE1, TE2, TE5, TE8, TE10, TE11, TE15) and non-neoplastic (Het-1A)	1.53	SILAC	20686364
IPO5	esophageal cancer	neoplastic (TE1, TE2, TE5, TE8, TE10, TE11, TE15) and non-neoplastic (Het-1A)	1.79	SILAC	20686364
ITSN1	esophageal cancer	neoplastic (TE1, TE2, TE5, TE8, TE10, TE11, TE15) and non-neoplastic (Het-1A)	-1.02	SILAC	20686364
JAG2	esophageal cancer	neoplastic (TE1, TE2, TE5, TE8, TE10, TE11, TE15) and non-neoplastic (Het-1A)	1.61	SILAC	20686364
KIAA0825	esophageal cancer	neoplastic (TE1, TE2, TE5, TE8, TE10, TE11, TE15) and non-neoplastic (Het-1A)	-2.55	SILAC	20686364
KLK6	esophageal cancer	neoplastic (TE1, TE2, TE5, TE8, TE10, TE11, TE15) and non-neoplastic (Het-1A)	2.57	SILAC	20686364
KRT17	esophageal cancer	neoplastic (TE1, TE2, TE5, TE8, TE10, TE11, TE15) and non-neoplastic (Het-1A)	1.19	SILAC	20686364
LOC400499	esophageal cancer	neoplastic (TE1, TE2, TE5, TE8, TE10, TE11, TE15) and non-neoplastic (Het-1A)	-1.12	SILAC	20686364
LPXN	esophageal cancer	neoplastic (TE1, TE2, TE5, TE8, TE10, TE11, TE15) and non-neoplastic (Het-1A)	-2.25	SILAC	20686364
MATN2	esophageal cancer	neoplastic (TE1, TE2, TE5, TE8, TE10, TE11, TE15) and non-neoplastic (Het-1A)	1.57	SILAC	20686364
MCM6	esophageal cancer	neoplastic (TE1, TE2, TE5, TE8, TE10, TE11, TE15) and non-neoplastic (Het-1A)	1.74	SILAC	20686364
MRTO4	esophageal cancer	neoplastic (TE1, TE2, TE5, TE8, TE10, TE11, TE15) and non-neoplastic (Het-1A)	1.50	SILAC	20686364
MSLN	esophageal cancer	neoplastic (TE1, TE2, TE5, TE8, TE10, TE11, TE15) and non-neoplastic (Het-1A)	-2.32	SILAC	20686364
MTPN	esophageal cancer	neoplastic (TE1, TE2, TE5, TE8, TE10, TE11, TE15) and non-neoplastic (Het-1A)	-1.73	SILAC	20686364
MXRA5	esophageal cancer	neoplastic (TE1, TE2, TE5, TE8, TE10, TE11, TE15) and non-neoplastic (Het-1A)	4.97	SILAC	20686364

MYL6B	esophageal cancer	neoplastic (TE1, TE2, TE5, TE8, TE10, TE11, TE15) and non-neoplastic (Het-1A)	-1	SILAC	20686364
NEFL	esophageal cancer	neoplastic (TE1, TE2, TE5, TE8, TE10, TE11, TE15) and non-neoplastic (Het-1A)	-1.32	SILAC	20686364
NENF	esophageal cancer	neoplastic (TE1, TE2, TE5, TE8, TE10, TE11, TE15) and non-neoplastic (Het-1A)	-1.02	SILAC	20686364
NGFR	esophageal cancer	neoplastic (TE1, TE2, TE5, TE8, TE10, TE11, TE15) and non-neoplastic (Het-1A)	-1.39	SILAC	20686364
NSUN4	esophageal cancer	neoplastic (TE1, TE2, TE5, TE8, TE10, TE11, TE15) and non-neoplastic (Het-1A)	9.08	SILAC	20686364
P4HB	esophageal cancer	neoplastic (TE1, TE2, TE5, TE8, TE10, TE11, TE15) and non-neoplastic (Het-1A)	2.09	SILAC	20686364
PAG1	esophageal cancer	neoplastic (TE1, TE2, TE5, TE8, TE10, TE11, TE15) and non-neoplastic (Het-1A)	-1.94	SILAC	20686364
PARD3	esophageal cancer	neoplastic (TE1, TE2, TE5, TE8, TE10, TE11, TE15) and non-neoplastic (Het-1A)	1.36	SILAC	20686364
PCDHGB7	esophageal cancer	neoplastic (TE1, TE2, TE5, TE8, TE10, TE11, TE15) and non-neoplastic (Het-1A)	-1	SILAC	20686364
PCNA	esophageal cancer	neoplastic (TE1, TE2, TE5, TE8, TE10, TE11, TE15) and non-neoplastic (Het-1A)	1.54	SILAC	20686364
PDDC1	esophageal cancer	neoplastic (TE1, TE2, TE5, TE8, TE10, TE11, TE15) and non-neoplastic (Het-1A)	-2.12	SILAC	20686364
PDXP	esophageal cancer	neoplastic (TE1, TE2, TE5, TE8, TE10, TE11, TE15) and non-neoplastic (Het-1A)	-1.05	SILAC	20686364
PEBP1	esophageal cancer	neoplastic (TE1, TE2, TE5, TE8, TE10, TE11, TE15) and non-neoplastic (Het-1A)	-1	SILAC	20686364
PIK3IP1	esophageal cancer	neoplastic (TE1, TE2, TE5, TE8, TE10, TE11, TE15) and non-neoplastic (Het-1A)	-2	SILAC	20686364
PLXNA1	esophageal cancer	neoplastic (TE1, TE2, TE5, TE8, TE10, TE11, TE15) and non-neoplastic (Het-1A)	-1.73	SILAC	20686364
PMP2	esophageal cancer	neoplastic (TE1, TE2, TE5, TE8, TE10, TE11, TE15) and non-neoplastic (Het-1A)	-3.18	SILAC	20686364
PPCS	esophageal cancer	neoplastic (TE1, TE2, TE5, TE8, TE10, TE11, TE15) and non-neoplastic (Het-1A)	-1.21	SILAC	20686364
PREP	esophageal cancer	neoplastic (TE1, TE2, TE5, TE8, TE10, TE11, TE15) and non-neoplastic (Het-1A)	-1.39	SILAC	20686364
PRKCSH	esophageal cancer	neoplastic (TE1, TE2, TE5, TE8, TE10, TE11, TE15) and non-neoplastic (Het-1A)	2.07	SILAC	20686364
PSMA7	esophageal cancer	neoplastic (TE1, TE2, TE5, TE8, TE10, TE11, TE15) and non-neoplastic (Het-1A)	-1	SILAC	20686364
PSMA7	esophageal cancer	neoplastic (TE1, TE2, TE5, TE8, TE10, TE11, TE15) and non-neoplastic (Het-1A)	1.83	SILAC	20686364
PSMB1	esophageal cancer	neoplastic (TE1, TE2, TE5, TE8, TE10, TE11, TE15) and non-neoplastic (Het-1A)	1.55	SILAC	20686364
PSMB6	esophageal cancer	neoplastic (TE1, TE2, TE5, TE8, TE10, TE11, TE15) and non-neoplastic (Het-1A)	1.12	SILAC	20686364
PSMD1	esophageal cancer	neoplastic (TE1, TE2, TE5, TE8, TE10, TE11, TE15) and non-neoplastic (Het-1A)	1.34	SILAC	20686364
PSMD12	esophageal cancer	neoplastic (TE1, TE2, TE5, TE8, TE10, TE11, TE15) and non-neoplastic (Het-1A)	2.19	SILAC	20686364
PTMS	esophageal cancer	neoplastic (TE1, TE2, TE5, TE8, TE10, TE11, TE15) and non-neoplastic (Het-1A)	2.24	SILAC	20686364
RPS18	esophageal cancer	neoplastic (TE1, TE2, TE5, TE8, TE10, TE11, TE15) and non-neoplastic (Het-1A)	1.72	SILAC	20686364
RTN4	esophageal cancer	neoplastic (TE1, TE2, TE5, TE8, TE10, TE11, TE15) and non-neoplastic (Het-1A)	1.57	SILAC	20686364
S100A2	esophageal cancer	neoplastic (TE1, TE2, TE5, TE8, TE10, TE11, TE15) and non-neoplastic (Het-1A)	-2.47	SILAC	20686364
S100A6	esophageal cancer	neoplastic (TE1, TE2, TE5, TE8, TE10, TE11, TE15) and non-neoplastic (Het-1A)	-1.88	SILAC	20686364
SAA1	esophageal cancer	neoplastic (TE1, TE2, TE5, TE8, TE10, TE11, TE15) and non-neoplastic (Het-1A)	-1.28	SILAC	20686364
SCLY	esophageal cancer	neoplastic (TE1, TE2, TE5, TE8, TE10, TE11, TE15) and non-neoplastic (Het-1A)	-1.35	SILAC	20686364
SDHA	esophageal cancer	neoplastic (TE1, TE2, TE5, TE8, TE10, TE11, TE15) and non-neoplastic (Het-1A)	-1.83	SILAC	20686364
SEC14L2	esophageal cancer	neoplastic (TE1, TE2, TE5, TE8, TE10, TE11, TE15) and non-neoplastic (Het-1A)	-1.78	SILAC	20686364
SERPINB5	esophageal cancer	neoplastic (TE1, TE2, TE5, TE8, TE10, TE11, TE15) and non-neoplastic (Het-1A)	-2.12	SILAC	20686364
SEZ6L2	esophageal cancer	neoplastic (TE1, TE2, TE5, TE8, TE10, TE11, TE15) and non-neoplastic (Het-1A)	-1.39	SILAC	20686364
SH3BGRL3	esophageal cancer	neoplastic (TE1, TE2, TE5, TE8, TE10, TE11, TE15) and non-neoplastic (Het-1A)	-1.88	SILAC	20686364
SMARCC2	esophageal cancer	neoplastic (TE1, TE2, TE5, TE8, TE10, TE11, TE15) and non-neoplastic (Het-1A)	1.33	SILAC	20686364
SNRPD3	esophageal cancer	neoplastic (TE1, TE2, TE5, TE8, TE10, TE11, TE15) and non-neoplastic (Het-1A)	-1.39	SILAC	20686364
SOD1	esophageal cancer	neoplastic (TE1, TE2, TE5, TE8, TE10, TE11, TE15) and non-neoplastic (Het-1A)	-1.08	SILAC	20686364
SORBS3	esophageal cancer	neoplastic (TE1, TE2, TE5, TE8, TE10, TE11, TE15) and non-neoplastic (Het-1A)	-1.05	SILAC	20686364
SPRYD4	esophageal cancer	neoplastic (TE1, TE2, TE5, TE8, TE10, TE11, TE15) and non-neoplastic (Het-1A)	-1.02	SILAC	20686364
STIL	esophageal cancer	neoplastic (TE1, TE2, TE5, TE8, TE10, TE11, TE15) and non-neoplastic (Het-1A)	6.82	SILAC	20686364

STX12	esophageal cancer	neoplastic (TE1, TE2, TE5, TE8, TE10, TE11, TE15) and non-neoplastic (Het-1A)	-1.18	SILAC	20686364
SUB1	esophageal cancer	neoplastic (TE1, TE2, TE5, TE8, TE10, TE11, TE15) and non-neoplastic (Het-1A)	1.53	SILAC	20686364
TARDBP	esophageal cancer	neoplastic (TE1, TE2, TE5, TE8, TE10, TE11, TE15) and non-neoplastic (Het-1A)	1.90	SILAC	20686364
TBCA	esophageal cancer	neoplastic (TE1, TE2, TE5, TE8, TE10, TE11, TE15) and non-neoplastic (Het-1A)	-1.05	SILAC	20686364
TCEB1	esophageal cancer	neoplastic (TE1, TE2, TE5, TE8, TE10, TE11, TE15) and non-neoplastic (Het-1A)	-1.47	SILAC	20686364
TIPRL	esophageal cancer	neoplastic (TE1, TE2, TE5, TE8, TE10, TE11, TE15) and non-neoplastic (Het-1A)	-1.12	SILAC	20686364
TNPO1	esophageal cancer	neoplastic (TE1, TE2, TE5, TE8, TE10, TE11, TE15) and non-neoplastic (Het-1A)	2.13	SILAC	20686364
TPM4	esophageal cancer	neoplastic (TE1, TE2, TE5, TE8, TE10, TE11, TE15) and non-neoplastic (Het-1A)	1.25	SILAC	20686364
TRIP6	esophageal cancer	neoplastic (TE1, TE2, TE5, TE8, TE10, TE11, TE15) and non-neoplastic (Het-1A)	-1.18	SILAC	20686364
TXNL4A	esophageal cancer	neoplastic (TE1, TE2, TE5, TE8, TE10, TE11, TE15) and non-neoplastic (Het-1A)	-1.47	SILAC	20686364
TYW1B	esophageal cancer	neoplastic (TE1, TE2, TE5, TE8, TE10, TE11, TE15) and non-neoplastic (Het-1A)	-3.18	SILAC	20686364
UBE2L6	esophageal cancer	neoplastic (TE1, TE2, TE5, TE8, TE10, TE11, TE15) and non-neoplastic (Het-1A)	-1.05	SILAC	20686364
ALDH1A1	lung cancer metastasis	neoplastic (CL1-0, CL1-5)	-1.64	iTRAQ	21186846
CCDC68	lung cancer metastasis	neoplastic (CL1-0, CL1-5)	1.07	iTRAQ	21186846
COL6A1	lung cancer metastasis	neoplastic (CL1-0, CL1-5)	1.51	iTRAQ	21186846
CTGF	lung cancer metastasis	neoplastic (CL1-0, CL1-5)	-2.64	iTRAQ	21186846
FABP5L3	lung cancer metastasis	neoplastic (CL1-0, CL1-5)	1.00	iTRAQ	21186846
FN1	lung cancer metastasis	neoplastic (CL1-0, CL1-5)	1.48	iTRAQ	21186846
GBE1	lung cancer metastasis	neoplastic (CL1-0, CL1-5)	-1.21	iTRAQ	21186846
HIST1H2BB	lung cancer metastasis	neoplastic (CL1-0, CL1-5)	2.09	iTRAQ	21186846
HIST1H2BD	lung cancer metastasis	neoplastic (CL1-0, CL1-5)	2.09	iTRAQ	21186846
HIST1H4A	lung cancer metastasis	neoplastic (CL1-0, CL1-5)	1.65	iTRAQ	21186846
HIST3H2BB	lung cancer metastasis	neoplastic (CL1-0, CL1-5)	2.09	iTRAQ	21186846
LOXL2	lung cancer metastasis	neoplastic (CL1-0, CL1-5)	2.93	iTRAQ	21186846
LSM14B	lung cancer metastasis	neoplastic (CL1-0, CL1-5)	2.63	iTRAQ	21186846
MAGEA4	lung cancer metastasis	neoplastic (CL1-0, CL1-5)	-1.05	iTRAQ	21186846
NUDT1	lung cancer metastasis	neoplastic (CL1-0, CL1-5)	-1.28	iTRAQ	21186846
PADI1	lung cancer metastasis	neoplastic (CL1-0, CL1-5)	-1	iTRAQ	21186846
PDA1	lung cancer metastasis	neoplastic (CL1-0, CL1-5)	2.21	iTRAQ	21186846
PLAU	lung cancer metastasis	neoplastic (CL1-0, CL1-5)	2.48	iTRAQ	21186846
PPP4R4	lung cancer metastasis	neoplastic (CL1-0, CL1-5)	-1.18	iTRAQ	21186846
PRDX1	lung cancer metastasis	neoplastic (CL1-0, CL1-5)	-1	iTRAQ	21186846
PTBP1	lung cancer metastasis	neoplastic (CL1-0, CL1-5)	-1.47	iTRAQ	21186846
QSOX1	lung cancer metastasis	neoplastic (CL1-0, CL1-5)	1.90	iTRAQ	21186846
RGPD7	lung cancer metastasis	neoplastic (CL1-0, CL1-5)	1.11	iTRAQ	21186846
SERPINA1	lung cancer metastasis	neoplastic (CL1-0, CL1-5)	2.92	iTRAQ	21186846
SPAG6	lung cancer metastasis	neoplastic (CL1-0, CL1-5)	1.26	iTRAQ	21186846
TACSTD1	lung cancer metastasis	neoplastic (CL1-0, CL1-5)	1.69	iTRAQ	21186846
THBS1	lung cancer metastasis	neoplastic (CL1-0, CL1-5)	1.19	iTRAQ	21186846
TIMP1	lung cancer metastasis	neoplastic (CL1-0, CL1-5)	1.81	iTRAQ	21186846
1A01	invasive glioblastoma	neoplastic (LN18, T98, U118, U87)	-1.88	SILAC	21574646
4F2	invasive glioblastoma	neoplastic (LN18, T98, U118, U87)	1.62	SILAC	21574646
A2MG	invasive glioblastoma	neoplastic (LN18, T98, U118, U87)	-3.64	SILAC	21574646

A4	invasive glioblastoma	neoplastic (LN18, T98, U118, U87)	-2.39	SILAC	21574646
A4	invasive glioblastoma	neoplastic (LN18, T98, U118, U87)	3.63	SILAC	21574646
ADA10	invasive glioblastoma	neoplastic (LN18, T98, U118, U87)	1.44	SILAC	21574646
ADA10	invasive glioblastoma	neoplastic (LN18, T98, U118, U87)	2.46	SILAC	21574646
ADAM9	invasive glioblastoma	neoplastic (LN18, T98, U118, U87)	3.21	SILAC	21574646
ADML	invasive glioblastoma	neoplastic (LN18, T98, U118, U87)	2.46	SILAC	21574646
ANAG	invasive glioblastoma	neoplastic (LN18, T98, U118, U87)	2.46	SILAC	21574646
ANAG	invasive glioblastoma	neoplastic (LN18, T98, U118, U87)	2.69	SILAC	21574646
ANGT	invasive glioblastoma	neoplastic (LN18, T98, U118, U87)	-1.78	SILAC	21574646
APOE	invasive glioblastoma	neoplastic (LN18, T98, U118, U87)	-1.43	SILAC	21574646
APOE	invasive glioblastoma	neoplastic (LN18, T98, U118, U87)	-2.05	SILAC	21574646
ARMET	invasive glioblastoma	neoplastic (LN18, T98, U118, U87)	1.08	SILAC	21574646
ARMET	invasive glioblastoma	neoplastic (LN18, T98, U118, U87)	-1.51	SILAC	21574646
ARMET	invasive glioblastoma	neoplastic (LN18, T98, U118, U87)	-3.05	SILAC	21574646
ASAH1	invasive glioblastoma	neoplastic (LN18, T98, U118, U87)	1.02	SILAC	21574646
ASAH1	invasive glioblastoma	neoplastic (LN18, T98, U118, U87)	3.52	SILAC	21574646
B2MG	invasive glioblastoma	neoplastic (LN18, T98, U118, U87)	3.37	SILAC	21574646
B2MG	invasive glioblastoma	neoplastic (LN18, T98, U118, U87)	-3.47	SILAC	21574646
BGH3	invasive glioblastoma	neoplastic (LN18, T98, U118, U87)	-2.83	SILAC	21574646
BGH3	invasive glioblastoma	neoplastic (LN18, T98, U118, U87)	-4.05	SILAC	21574646
C1R	invasive glioblastoma	neoplastic (LN18, T98, U118, U87)	-1.39	SILAC	21574646
C1R	invasive glioblastoma	neoplastic (LN18, T98, U118, U87)	-2.32	SILAC	21574646
C1S	invasive glioblastoma	neoplastic (LN18, T98, U118, U87)	-1.39	SILAC	21574646
C1S	invasive glioblastoma	neoplastic (LN18, T98, U118, U87)	-2.73	SILAC	21574646
C1S	invasive glioblastoma	neoplastic (LN18, T98, U118, U87)	-4.32	SILAC	21574646
CADH6	invasive glioblastoma	neoplastic (LN18, T98, U118, U87)	-4.05	SILAC	21574646
CAH12	invasive glioblastoma	neoplastic (LN18, T98, U118, U87)	2.68	SILAC	21574646
CALR	invasive glioblastoma	neoplastic (LN18, T98, U118, U87)	1.67	SILAC	21574646
CALR	invasive glioblastoma	neoplastic (LN18, T98, U118, U87)	-2.55	SILAC	21574646
CALU	invasive glioblastoma	neoplastic (LN18, T98, U118, U87)	-1.28	SILAC	21574646
CALU	invasive glioblastoma	neoplastic (LN18, T98, U118, U87)	-1.83	SILAC	21574646
CALU	invasive glioblastoma	neoplastic (LN18, T98, U118, U87)	5.01	SILAC	21574646
CATB	invasive glioblastoma	neoplastic (LN18, T98, U118, U87)	-1.68	SILAC	21574646
CATB	invasive glioblastoma	neoplastic (LN18, T98, U118, U87)	2.91	SILAC	21574646
CATB	invasive glioblastoma	neoplastic (LN18, T98, U118, U87)	3.49	SILAC	21574646
CATB	invasive glioblastoma	neoplastic (LN18, T98, U118, U87)	3.59	SILAC	21574646
CATL1	invasive glioblastoma	neoplastic (LN18, T98, U118, U87)	2.72	SILAC	21574646
CATS	invasive glioblastoma	neoplastic (LN18, T98, U118, U87)	3.58	SILAC	21574646
CATZ	invasive glioblastoma	neoplastic (LN18, T98, U118, U87)	-5.05	SILAC	21574646
CD109	invasive glioblastoma	neoplastic (LN18, T98, U118, U87)	-1.32	SILAC	21574646
CD109	invasive glioblastoma	neoplastic (LN18, T98, U118, U87)	1.45	SILAC	21574646
CD109	invasive glioblastoma	neoplastic (LN18, T98, U118, U87)	2.81	SILAC	21574646
CD109	invasive glioblastoma	neoplastic (LN18, T98, U118, U87)	-3.05	SILAC	21574646

CD44	invasive glioblastoma	neoplastic (LN18, T98, U118, U87)	-1.83	SILAC	21574646
CD44	invasive glioblastoma	neoplastic (LN18, T98, U118, U87)	3.38	SILAC	21574646
CD9	invasive glioblastoma	neoplastic (LN18, T98, U118, U87)	3.19	SILAC	21574646
CFAB	invasive glioblastoma	neoplastic (LN18, T98, U118, U87)	4.39	SILAC	21574646
CH3L1	invasive glioblastoma	neoplastic (LN18, T98, U118, U87)	-1.39	SILAC	21574646
CH3L1	invasive glioblastoma	neoplastic (LN18, T98, U118, U87)	-3.83	SILAC	21574646
CH3L2	invasive glioblastoma	neoplastic (LN18, T98, U118, U87)	3.87	SILAC	21574646
CHID1	invasive glioblastoma	neoplastic (LN18, T98, U118, U87)	-3.47	SILAC	21574646
CLIC1	invasive glioblastoma	neoplastic (LN18, T98, U118, U87)	-2.05	SILAC	21574646
CLIC4	invasive glioblastoma	neoplastic (LN18, T98, U118, U87)	3.70	SILAC	21574646
CLUS	invasive glioblastoma	neoplastic (LN18, T98, U118, U87)	-1.05	SILAC	21574646
CLUS	invasive glioblastoma	neoplastic (LN18, T98, U118, U87)	1.66	SILAC	21574646
CLUS	invasive glioblastoma	neoplastic (LN18, T98, U118, U87)	-2.12	SILAC	21574646
CLUS	invasive glioblastoma	neoplastic (LN18, T98, U118, U87)	2.46	SILAC	21574646
CLUS	invasive glioblastoma	neoplastic (LN18, T98, U118, U87)	2.80	SILAC	21574646
CO1A2	invasive glioblastoma	neoplastic (LN18, T98, U118, U87)	1.11	SILAC	21574646
CO1A2	invasive glioblastoma	neoplastic (LN18, T98, U118, U87)	-2.39	SILAC	21574646
CO1A2	invasive glioblastoma	neoplastic (LN18, T98, U118, U87)	-2.73	SILAC	21574646
CO1A2	invasive glioblastoma	neoplastic (LN18, T98, U118, U87)	2.92	SILAC	21574646
CO1A2	invasive glioblastoma	neoplastic (LN18, T98, U118, U87)	3.56	SILAC	21574646
CO1A2	invasive glioblastoma	neoplastic (LN18, T98, U118, U87)	3.75	SILAC	21574646
CO1A2	invasive glioblastoma	neoplastic (LN18, T98, U118, U87)	4.49	SILAC	21574646
CO3	invasive glioblastoma	neoplastic (LN18, T98, U118, U87)	1.55	SILAC	21574646
CO4A	invasive glioblastoma	neoplastic (LN18, T98, U118, U87)	-2.05	SILAC	21574646
CO6A1	invasive glioblastoma	neoplastic (LN18, T98, U118, U87)	-1.83	SILAC	21574646
CO6A1	invasive glioblastoma	neoplastic (LN18, T98, U118, U87)	2.04	SILAC	21574646
CO6A1	invasive glioblastoma	neoplastic (LN18, T98, U118, U87)	-3.47	SILAC	21574646
CO6A1	invasive glioblastoma	neoplastic (LN18, T98, U118, U87)	3.76	SILAC	21574646
CO6A2	invasive glioblastoma	neoplastic (LN18, T98, U118, U87)	3.32	SILAC	21574646
CO6A2	invasive glioblastoma	neoplastic (LN18, T98, U118, U87)	-3.32	SILAC	21574646
CO6A2	invasive glioblastoma	neoplastic (LN18, T98, U118, U87)	3.43	SILAC	21574646
CO6A3	invasive glioblastoma	neoplastic (LN18, T98, U118, U87)	-1.35	SILAC	21574646
CO6A3	invasive glioblastoma	neoplastic (LN18, T98, U118, U87)	-3.83	SILAC	21574646
CO6A3	invasive glioblastoma	neoplastic (LN18, T98, U118, U87)	-3.83	SILAC	21574646
CO7A1	invasive glioblastoma	neoplastic (LN18, T98, U118, U87)	-1.47	SILAC	21574646
CO7A1	invasive glioblastoma	neoplastic (LN18, T98, U118, U87)	-3.47	SILAC	21574646
COCA1	invasive glioblastoma	neoplastic (LN18, T98, U118, U87)	-2.47	SILAC	21574646
COCA1	invasive glioblastoma	neoplastic (LN18, T98, U118, U87)	-2.55	SILAC	21574646
COEA1	invasive glioblastoma	neoplastic (LN18, T98, U118, U87)	4.74	SILAC	21574646
COIA1	invasive glioblastoma	neoplastic (LN18, T98, U118, U87)	-3.32	SILAC	21574646
COPA	invasive glioblastoma	neoplastic (LN18, T98, U118, U87)	-2.25	SILAC	21574646
CS010	invasive glioblastoma	neoplastic (LN18, T98, U118, U87)	1.02	SILAC	21574646
CS010	invasive glioblastoma	neoplastic (LN18, T98, U118, U87)	-2.55	SILAC	21574646

CS010	invasive glioblastoma	neoplastic (LN18, T98, U118, U87)	4.59	SILAC	21574646
CSF1	invasive glioblastoma	neoplastic (LN18, T98, U118, U87)	-2.55	SILAC	21574646
CSF1	invasive glioblastoma	neoplastic (LN18, T98, U118, U87)	3.05	SILAC	21574646
CSTN1	invasive glioblastoma	neoplastic (LN18, T98, U118, U87)	1.22	SILAC	21574646
CSTN1	invasive glioblastoma	neoplastic (LN18, T98, U118, U87)	2.02	SILAC	21574646
CSTN2	invasive glioblastoma	neoplastic (LN18, T98, U118, U87)	-2.73	SILAC	21574646
CYTB	invasive glioblastoma	neoplastic (LN18, T98, U118, U87)	-1	SILAC	21574646
CYTB	invasive glioblastoma	neoplastic (LN18, T98, U118, U87)	3.47	SILAC	21574646
CYTC	invasive glioblastoma	neoplastic (LN18, T98, U118, U87)	-2.73	SILAC	21574646
DKK3	invasive glioblastoma	neoplastic (LN18, T98, U118, U87)	-1.55	SILAC	21574646
DKK3	invasive glioblastoma	neoplastic (LN18, T98, U118, U87)	-2.83	SILAC	21574646
DMP4	invasive glioblastoma	neoplastic (LN18, T98, U118, U87)	1.86	SILAC	21574646
DSG2	invasive glioblastoma	neoplastic (LN18, T98, U118, U87)	2.57	SILAC	21574646
EDIL3	invasive glioblastoma	neoplastic (LN18, T98, U118, U87)	2.71	SILAC	21574646
EPDR1	invasive glioblastoma	neoplastic (LN18, T98, U118, U87)	3.62	SILAC	21574646
ERP29	invasive glioblastoma	neoplastic (LN18, T98, U118, U87)	2.54	SILAC	21574646
FAM3C	invasive glioblastoma	neoplastic (LN18, T98, U118, U87)	3.83	SILAC	21574646
FBN1	invasive glioblastoma	neoplastic (LN18, T98, U118, U87)	2.14	SILAC	21574646
FBN1	invasive glioblastoma	neoplastic (LN18, T98, U118, U87)	3.06	SILAC	21574646
FBN1	invasive glioblastoma	neoplastic (LN18, T98, U118, U87)	3.46	SILAC	21574646
FINC	invasive glioblastoma	neoplastic (LN18, T98, U118, U87)	1.96	SILAC	21574646
FINC	invasive glioblastoma	neoplastic (LN18, T98, U118, U87)	-4.64	SILAC	21574646
FSTL1	invasive glioblastoma	neoplastic (LN18, T98, U118, U87)	2.51	SILAC	21574646
FSTL1	invasive glioblastoma	neoplastic (LN18, T98, U118, U87)	2.74	SILAC	21574646
FUCO2	invasive glioblastoma	neoplastic (LN18, T98, U118, U87)	2.33	SILAC	21574646
GALNS	invasive glioblastoma	neoplastic (LN18, T98, U118, U87)	1.83	SILAC	21574646
GALT2	invasive glioblastoma	neoplastic (LN18, T98, U118, U87)	1.96	SILAC	21574646
GDN	invasive glioblastoma	neoplastic (LN18, T98, U118, U87)	-1.83	SILAC	21574646
GDN	invasive glioblastoma	neoplastic (LN18, T98, U118, U87)	1.97	SILAC	21574646
GDN	invasive glioblastoma	neoplastic (LN18, T98, U118, U87)	-2.32	SILAC	21574646
GDN	invasive glioblastoma	neoplastic (LN18, T98, U118, U87)	3.37	SILAC	21574646
GELS	invasive glioblastoma	neoplastic (LN18, T98, U118, U87)	2.84	SILAC	21574646
GGH	invasive glioblastoma	neoplastic (LN18, T98, U118, U87)	4.76	SILAC	21574646
GLCM	invasive glioblastoma	neoplastic (LN18, T98, U118, U87)	-2.94	SILAC	21574646
GNS	invasive glioblastoma	neoplastic (LN18, T98, U118, U87)	-1.15	SILAC	21574646
GNS	invasive glioblastoma	neoplastic (LN18, T98, U118, U87)	1.98	SILAC	21574646
GPC1	invasive glioblastoma	neoplastic (LN18, T98, U118, U87)	1.08	SILAC	21574646
GPC1	invasive glioblastoma	neoplastic (LN18, T98, U118, U87)	-1.78	SILAC	21574646
GRN	invasive glioblastoma	neoplastic (LN18, T98, U118, U87)	1.19	SILAC	21574646
GRN	invasive glioblastoma	neoplastic (LN18, T98, U118, U87)	-1.21	SILAC	21574646
GRN	invasive glioblastoma	neoplastic (LN18, T98, U118, U87)	3.28	SILAC	21574646
GRP78	invasive glioblastoma	neoplastic (LN18, T98, U118, U87)	-1.39	SILAC	21574646
GRP78	invasive glioblastoma	neoplastic (LN18, T98, U118, U87)	2.25	SILAC	21574646

HEXB	invasive glioblastoma	neoplastic (LN18, T98, U118, U87)	2.71	SILAC	21574646
HTRA1	invasive glioblastoma	neoplastic (LN18, T98, U118, U87)	2.36	SILAC	21574646
HTRA1	invasive glioblastoma	neoplastic (LN18, T98, U118, U87)	3.07	SILAC	21574646
IBP2	invasive glioblastoma	neoplastic (LN18, T98, U118, U87)	-6.64	SILAC	21574646
IBP3	invasive glioblastoma	neoplastic (LN18, T98, U118, U87)	-3.18	SILAC	21574646
IBP3	invasive glioblastoma	neoplastic (LN18, T98, U118, U87)	-3.64	SILAC	21574646
IBP5	invasive glioblastoma	neoplastic (LN18, T98, U118, U87)	-1.73	SILAC	21574646
IBP5	invasive glioblastoma	neoplastic (LN18, T98, U118, U87)	-1.94	SILAC	21574646
IBP5	invasive glioblastoma	neoplastic (LN18, T98, U118, U87)	-4.05	SILAC	21574646
IBP6	invasive glioblastoma	neoplastic (LN18, T98, U118, U87)	2.87	SILAC	21574646
IBP6	invasive glioblastoma	neoplastic (LN18, T98, U118, U87)	-3.05	SILAC	21574646
ITA3	invasive glioblastoma	neoplastic (LN18, T98, U118, U87)	2.88	SILAC	21574646
ITAV	invasive glioblastoma	neoplastic (LN18, T98, U118, U87)	2.06	SILAC	21574646
LAMB1	invasive glioblastoma	neoplastic (LN18, T98, U118, U87)	-1.28	SILAC	21574646
LAMB1	invasive glioblastoma	neoplastic (LN18, T98, U118, U87)	-1.78	SILAC	21574646
LEG1	invasive glioblastoma	neoplastic (LN18, T98, U118, U87)	1.42	SILAC	21574646
LEG1	invasive glioblastoma	neoplastic (LN18, T98, U118, U87)	-2.12	SILAC	21574646
LG3BP	invasive glioblastoma	neoplastic (LN18, T98, U118, U87)	-1.73	SILAC	21574646
LG3BP	invasive glioblastoma	neoplastic (LN18, T98, U118, U87)	-1.94	SILAC	21574646
LICH	invasive glioblastoma	neoplastic (LN18, T98, U118, U87)	2.11	SILAC	21574646
LOXL1	invasive glioblastoma	neoplastic (LN18, T98, U118, U87)	3.50	SILAC	21574646
LOXL2	invasive glioblastoma	neoplastic (LN18, T98, U118, U87)	-2.83	SILAC	21574646
LYOX	invasive glioblastoma	neoplastic (LN18, T98, U118, U87)	3.38	SILAC	21574646
MANBA	invasive glioblastoma	neoplastic (LN18, T98, U118, U87)	-2.05	SILAC	21574646
MATN2	invasive glioblastoma	neoplastic (LN18, T98, U118, U87)	4.45	SILAC	21574646
MFAP2	invasive glioblastoma	neoplastic (LN18, T98, U118, U87)	4.14	SILAC	21574646
MMP1	invasive glioblastoma	neoplastic (LN18, T98, U118, U87)	2.51	SILAC	21574646
MMP1	invasive glioblastoma	neoplastic (LN18, T98, U118, U87)	-3.83	SILAC	21574646
MMP14	invasive glioblastoma	neoplastic (LN18, T98, U118, U87)	3.59	SILAC	21574646
MMP2	invasive glioblastoma	neoplastic (LN18, T98, U118, U87)	1.59	SILAC	21574646
MMP2	invasive glioblastoma	neoplastic (LN18, T98, U118, U87)	1.69	SILAC	21574646
MMP3	invasive glioblastoma	neoplastic (LN18, T98, U118, U87)	3.40	SILAC	21574646
MYOF	invasive glioblastoma	neoplastic (LN18, T98, U118, U87)	2.19	SILAC	21574646
NPTX1	invasive glioblastoma	neoplastic (LN18, T98, U118, U87)	2.05	SILAC	21574646
NPTX1	invasive glioblastoma	neoplastic (LN18, T98, U118, U87)	-6.64	SILAC	21574646
NPTXR	invasive glioblastoma	neoplastic (LN18, T98, U118, U87)	1.68	SILAC	21574646
NUCB1	invasive glioblastoma	neoplastic (LN18, T98, U118, U87)	1.04	SILAC	21574646
NUCB1	invasive glioblastoma	neoplastic (LN18, T98, U118, U87)	2.77	SILAC	21574646
NUCB1	invasive glioblastoma	neoplastic (LN18, T98, U118, U87)	-2.83	SILAC	21574646
NUCB1	invasive glioblastoma	neoplastic (LN18, T98, U118, U87)	-3.32	SILAC	21574646
NUCB1	invasive glioblastoma	neoplastic (LN18, T98, U118, U87)	-4.05	SILAC	21574646
NUCB2	invasive glioblastoma	neoplastic (LN18, T98, U118, U87)	2.69	SILAC	21574646
PAI1	invasive glioblastoma	neoplastic (LN18, T98, U118, U87)	5.39	SILAC	21574646

PCDGK	invasive glioblastoma	neoplastic (LN18, T98, U118, U87)	2.26	SILAC	21574646
PCOC1	invasive glioblastoma	neoplastic (LN18, T98, U118, U87)	1.04	SILAC	21574646
PCOC1	invasive glioblastoma	neoplastic (LN18, T98, U118, U87)	1.07	SILAC	21574646
PCOC1	invasive glioblastoma	neoplastic (LN18, T98, U118, U87)	1.09	SILAC	21574646
PCOC1	invasive glioblastoma	neoplastic (LN18, T98, U118, U87)	2.39	SILAC	21574646
PCOC1	invasive glioblastoma	neoplastic (LN18, T98, U118, U87)	-3.05	SILAC	21574646
PGK1	invasive glioblastoma	neoplastic (LN18, T98, U118, U87)	-1.18	SILAC	21574646
PGK1	invasive glioblastoma	neoplastic (LN18, T98, U118, U87)	-3.47	SILAC	21574646
PGRC2	invasive glioblastoma	neoplastic (LN18, T98, U118, U87)	1.88	SILAC	21574646
PLBL2	invasive glioblastoma	neoplastic (LN18, T98, U118, U87)	2.03	SILAC	21574646
PLIN3	invasive glioblastoma	neoplastic (LN18, T98, U118, U87)	1.68	SILAC	21574646
PLTP	invasive glioblastoma	neoplastic (LN18, T98, U118, U87)	2.93	SILAC	21574646
POSTN	invasive glioblastoma	neoplastic (LN18, T98, U118, U87)	3.73	SILAC	21574646
PPGB	invasive glioblastoma	neoplastic (LN18, T98, U118, U87)	-2.64	SILAC	21574646
PPT1	invasive glioblastoma	neoplastic (LN18, T98, U118, U87)	1.84	SILAC	21574646
PROS	invasive glioblastoma	neoplastic (LN18, T98, U118, U87)	2.40	SILAC	21574646
PTX3	invasive glioblastoma	neoplastic (LN18, T98, U118, U87)	1.48	SILAC	21574646
PTX3	invasive glioblastoma	neoplastic (LN18, T98, U118, U87)	2.66	SILAC	21574646
PTX3	invasive glioblastoma	neoplastic (LN18, T98, U118, U87)	4.45	SILAC	21574646
PVR	invasive glioblastoma	neoplastic (LN18, T98, U118, U87)	3.36	SILAC	21574646
PXDN	invasive glioblastoma	neoplastic (LN18, T98, U118, U87)	1.56	SILAC	21574646
RAB5C	invasive glioblastoma	neoplastic (LN18, T98, U118, U87)	1.85	SILAC	21574646
RAB7A	invasive glioblastoma	neoplastic (LN18, T98, U118, U87)	2.25	SILAC	21574646
RCN1	invasive glioblastoma	neoplastic (LN18, T98, U118, U87)	-1.35	SILAC	21574646
RCN1	invasive glioblastoma	neoplastic (LN18, T98, U118, U87)	-1.83	SILAC	21574646
RCN1	invasive glioblastoma	neoplastic (LN18, T98, U118, U87)	2.38	SILAC	21574646
RENR	invasive glioblastoma	neoplastic (LN18, T98, U118, U87)	-2.05	SILAC	21574646
RNT2	invasive glioblastoma	neoplastic (LN18, T98, U118, U87)	3.33	SILAC	21574646
S10A7	invasive glioblastoma	neoplastic (LN18, T98, U118, U87)	2.28	SILAC	21574646
SAP	invasive glioblastoma	neoplastic (LN18, T98, U118, U87)	1.13	SILAC	21574646
SAP	invasive glioblastoma	neoplastic (LN18, T98, U118, U87)	-1.78	SILAC	21574646
SAP	invasive glioblastoma	neoplastic (LN18, T98, U118, U87)	1.86	SILAC	21574646
SAP	invasive glioblastoma	neoplastic (LN18, T98, U118, U87)	-2.05	SILAC	21574646
SAP	invasive glioblastoma	neoplastic (LN18, T98, U118, U87)	2.58	SILAC	21574646
SAP	invasive glioblastoma	neoplastic (LN18, T98, U118, U87)	2.95	SILAC	21574646
SAP	invasive glioblastoma	neoplastic (LN18, T98, U118, U87)	3.83	SILAC	21574646
SERA	invasive glioblastoma	neoplastic (LN18, T98, U118, U87)	-1.88	SILAC	21574646
SERA	invasive glioblastoma	neoplastic (LN18, T98, U118, U87)	-2.18	SILAC	21574646
SPRC	invasive glioblastoma	neoplastic (LN18, T98, U118, U87)	-5.05	SILAC	21574646
SRPX	invasive glioblastoma	neoplastic (LN18, T98, U118, U87)	2.81	SILAC	21574646
STC1	invasive glioblastoma	neoplastic (LN18, T98, U118, U87)	1.10	SILAC	21574646
TFR1	invasive glioblastoma	neoplastic (LN18, T98, U118, U87)	2.09	SILAC	21574646
TICN1	invasive glioblastoma	neoplastic (LN18, T98, U118, U87)	4.63	SILAC	21574646

TIMP1	invasive glioblastoma	neoplastic (LN18, T98, U118, U87)	2.89	SILAC	21574646
TIMP2	invasive glioblastoma	neoplastic (LN18, T98, U118, U87)	-2.39	SILAC	21574646
TPP1	invasive glioblastoma	neoplastic (LN18, T98, U118, U87)	1.76	SILAC	21574646
TPP1	invasive glioblastoma	neoplastic (LN18, T98, U118, U87)	-1.78	SILAC	21574646
TRXR1	invasive glioblastoma	neoplastic (LN18, T98, U118, U87)	-1.94	SILAC	21574646
TSP1	invasive glioblastoma	neoplastic (LN18, T98, U118, U87)	-1.51	SILAC	21574646
TSP1	invasive glioblastoma	neoplastic (LN18, T98, U118, U87)	-1.94	SILAC	21574646
TSP1	invasive glioblastoma	neoplastic (LN18, T98, U118, U87)	-4.32	SILAC	21574646
TSP1	invasive glioblastoma	neoplastic (LN18, T98, U118, U87)	-4.64	SILAC	21574646
UFO	invasive glioblastoma	neoplastic (LN18, T98, U118, U87)	1.23	SILAC	21574646
UFO	invasive glioblastoma	neoplastic (LN18, T98, U118, U87)	3.42	SILAC	21574646
UFO	invasive glioblastoma	neoplastic (LN18, T98, U118, U87)	3.78	SILAC	21574646
UFO	invasive glioblastoma	neoplastic (LN18, T98, U118, U87)	3.98	SILAC	21574646
VAMP2	invasive glioblastoma	neoplastic (LN18, T98, U118, U87)	2.13	SILAC	21574646
VASN	invasive glioblastoma	neoplastic (LN18, T98, U118, U87)	2.10	SILAC	21574646
VIME	invasive glioblastoma	neoplastic (LN18, T98, U118, U87)	1.07	SILAC	21574646
VIME	invasive glioblastoma	neoplastic (LN18, T98, U118, U87)	1.15	SILAC	21574646
VIME	invasive glioblastoma	neoplastic (LN18, T98, U118, U87)	-1.39	SILAC	21574646
VIME	invasive glioblastoma	neoplastic (LN18, T98, U118, U87)	1.76	SILAC	21574646
VIME	invasive glioblastoma	neoplastic (LN18, T98, U118, U87)	-1.78	SILAC	21574646
VIME	invasive glioblastoma	neoplastic (LN18, T98, U118, U87)	1.85	SILAC	21574646
VIME	invasive glioblastoma	neoplastic (LN18, T98, U118, U87)	2.08	SILAC	21574646
VIME	invasive glioblastoma	neoplastic (LN18, T98, U118, U87)	-2.18	SILAC	21574646
VIME	invasive glioblastoma	neoplastic (LN18, T98, U118, U87)	2.29	SILAC	21574646
VIME	invasive glioblastoma	neoplastic (LN18, T98, U118, U87)	2.33	SILAC	21574646
VIME	invasive glioblastoma	neoplastic (LN18, T98, U118, U87)	-2.39	SILAC	21574646
VIME	invasive glioblastoma	neoplastic (LN18, T98, U118, U87)	2.50	SILAC	21574646
VIME	invasive glioblastoma	neoplastic (LN18, T98, U118, U87)	3.42	SILAC	21574646
VIME	invasive glioblastoma	neoplastic (LN18, T98, U118, U87)	3.47	SILAC	21574646
VPS35	invasive glioblastoma	neoplastic (LN18, T98, U118, U87)	-2.55	SILAC	21574646
ADAM10	head and neck cancer	neoplastic (FaDu, UTSCC8, UTSCC42a) and non-neoplastic (NOE)	1.43	SILAC	22918226
ADAM10	head and neck cancer	neoplastic (FaDu, UTSCC8, UTSCC42a) and non-neoplastic (NOE)	2.09	SILAC	22918226
ADAM10	head and neck cancer	neoplastic (FaDu, UTSCC8, UTSCC42a) and non-neoplastic (NOE)	2.38	SILAC	22918226
ADAM10	head and neck cancer	neoplastic (FaDu, UTSCC8, UTSCC42a) and non-neoplastic (NOE)	5.64	SILAC	22918226
ADAM10	head and neck cancer	neoplastic (FaDu, UTSCC8, UTSCC42a) and non-neoplastic (NOE)	6.07	SILAC	22918226
ADAM10	head and neck cancer	neoplastic (FaDu, UTSCC8, UTSCC42a) and non-neoplastic (NOE)	6.09	SILAC	22918226
AGRN	head and neck cancer	neoplastic (FaDu, UTSCC8, UTSCC42a) and non-neoplastic (NOE)	2.03	SILAC	22918226
AGRN	head and neck cancer	neoplastic (FaDu, UTSCC8, UTSCC42a) and non-neoplastic (NOE)	2.35	SILAC	22918226
AGRN	head and neck cancer	neoplastic (FaDu, UTSCC8, UTSCC42a) and non-neoplastic (NOE)	4.47	SILAC	22918226
AGRN	head and neck cancer	neoplastic (FaDu, UTSCC8, UTSCC42a) and non-neoplastic (NOE)	7.21	SILAC	22918226
AGRN	head and neck cancer	neoplastic (FaDu, UTSCC8, UTSCC42a) and non-neoplastic (NOE)	7.37	SILAC	22918226
AKR1C1	head and neck cancer	neoplastic (FaDu, UTSCC8, UTSCC42a) and non-neoplastic (NOE)	5.95	SILAC	22918226
AKR1C1	head and neck cancer	neoplastic (FaDu, UTSCC8, UTSCC42a) and non-neoplastic (NOE)	6.18	SILAC	22918226

AKR1C1	head and neck cancer	neoplastic (FaDu, UTSCC8, UTSCC42a) and non-neoplastic (NOE)	6.71	SILAC	22918226
AKR1C1	head and neck cancer	neoplastic (FaDu, UTSCC8, UTSCC42a) and non-neoplastic (NOE)	6.90	SILAC	22918226
AKR1C1	head and neck cancer	neoplastic (FaDu, UTSCC8, UTSCC42a) and non-neoplastic (NOE)	7.98	SILAC	22918226
ALDOA	head and neck cancer	neoplastic (FaDu, UTSCC8, UTSCC42a) and non-neoplastic (NOE)	2.31	SILAC	22918226
ALDOA	head and neck cancer	neoplastic (FaDu, UTSCC8, UTSCC42a) and non-neoplastic (NOE)	2.79	SILAC	22918226
ALDOA	head and neck cancer	neoplastic (FaDu, UTSCC8, UTSCC42a) and non-neoplastic (NOE)	3.04	SILAC	22918226
ALDOA	head and neck cancer	neoplastic (FaDu, UTSCC8, UTSCC42a) and non-neoplastic (NOE)	3.21	SILAC	22918226
ALDOA	head and neck cancer	neoplastic (FaDu, UTSCC8, UTSCC42a) and non-neoplastic (NOE)	3.22	SILAC	22918226
ALDOC	head and neck cancer	neoplastic (FaDu, UTSCC8, UTSCC42a) and non-neoplastic (NOE)	1.07	SILAC	22918226
ALDOC	head and neck cancer	neoplastic (FaDu, UTSCC8, UTSCC42a) and non-neoplastic (NOE)	1.13	SILAC	22918226
ALDOC	head and neck cancer	neoplastic (FaDu, UTSCC8, UTSCC42a) and non-neoplastic (NOE)	2.13	SILAC	22918226
ALDOC	head and neck cancer	neoplastic (FaDu, UTSCC8, UTSCC42a) and non-neoplastic (NOE)	3.11	SILAC	22918226
ALDOC	head and neck cancer	neoplastic (FaDu, UTSCC8, UTSCC42a) and non-neoplastic (NOE)	3.28	SILAC	22918226
ALDOC	head and neck cancer	neoplastic (FaDu, UTSCC8, UTSCC42a) and non-neoplastic (NOE)	7.10	SILAC	22918226
ARHGDIB	head and neck cancer	neoplastic (FaDu, UTSCC8, UTSCC42a) and non-neoplastic (NOE)	10.10	SILAC	22918226
ARHGDIB	head and neck cancer	neoplastic (FaDu, UTSCC8, UTSCC42a) and non-neoplastic (NOE)	2.62	SILAC	22918226
ARHGDIB	head and neck cancer	neoplastic (FaDu, UTSCC8, UTSCC42a) and non-neoplastic (NOE)	5.17	SILAC	22918226
ARHGDIB	head and neck cancer	neoplastic (FaDu, UTSCC8, UTSCC42a) and non-neoplastic (NOE)	6.45	SILAC	22918226
ARHGDIB	head and neck cancer	neoplastic (FaDu, UTSCC8, UTSCC42a) and non-neoplastic (NOE)	9.64	SILAC	22918226
ARHGDIB	head and neck cancer	neoplastic (FaDu, UTSCC8, UTSCC42a) and non-neoplastic (NOE)	9.68	SILAC	22918226
AXL	head and neck cancer	neoplastic (FaDu, UTSCC8, UTSCC42a) and non-neoplastic (NOE)	1.53	SILAC	22918226
AXL	head and neck cancer	neoplastic (FaDu, UTSCC8, UTSCC42a) and non-neoplastic (NOE)	6.06	SILAC	22918226
AXL	head and neck cancer	neoplastic (FaDu, UTSCC8, UTSCC42a) and non-neoplastic (NOE)	6.09	SILAC	22918226
AXL	head and neck cancer	neoplastic (FaDu, UTSCC8, UTSCC42a) and non-neoplastic (NOE)	7.46	SILAC	22918226
AXL	head and neck cancer	neoplastic (FaDu, UTSCC8, UTSCC42a) and non-neoplastic (NOE)	7.92	SILAC	22918226
AXL	head and neck cancer	neoplastic (FaDu, UTSCC8, UTSCC42a) and non-neoplastic (NOE)	8.67	SILAC	22918226
BANF1	head and neck cancer	neoplastic (FaDu, UTSCC8, UTSCC42a) and non-neoplastic (NOE)	1.15	SILAC	22918226
BANF1	head and neck cancer	neoplastic (FaDu, UTSCC8, UTSCC42a) and non-neoplastic (NOE)	1.50	SILAC	22918226
BANF1	head and neck cancer	neoplastic (FaDu, UTSCC8, UTSCC42a) and non-neoplastic (NOE)	2.89	SILAC	22918226
BANF1	head and neck cancer	neoplastic (FaDu, UTSCC8, UTSCC42a) and non-neoplastic (NOE)	6.29	SILAC	22918226
BANF1	head and neck cancer	neoplastic (FaDu, UTSCC8, UTSCC42a) and non-neoplastic (NOE)	6.33	SILAC	22918226
BASP1	head and neck cancer	neoplastic (FaDu, UTSCC8, UTSCC42a) and non-neoplastic (NOE)	1.46	SILAC	22918226
BASP1	head and neck cancer	neoplastic (FaDu, UTSCC8, UTSCC42a) and non-neoplastic (NOE)	2.08	SILAC	22918226
BASP1	head and neck cancer	neoplastic (FaDu, UTSCC8, UTSCC42a) and non-neoplastic (NOE)	2.44	SILAC	22918226
BASP1	head and neck cancer	neoplastic (FaDu, UTSCC8, UTSCC42a) and non-neoplastic (NOE)	3.13	SILAC	22918226
BASP1	head and neck cancer	neoplastic (FaDu, UTSCC8, UTSCC42a) and non-neoplastic (NOE)	6.31	SILAC	22918226
BPNT1	head and neck cancer	neoplastic (FaDu, UTSCC8, UTSCC42a) and non-neoplastic (NOE)	1.08	SILAC	22918226
BPNT1	head and neck cancer	neoplastic (FaDu, UTSCC8, UTSCC42a) and non-neoplastic (NOE)	2.32	SILAC	22918226
BPNT1	head and neck cancer	neoplastic (FaDu, UTSCC8, UTSCC42a) and non-neoplastic (NOE)	3.42	SILAC	22918226
BPNT1	head and neck cancer	neoplastic (FaDu, UTSCC8, UTSCC42a) and non-neoplastic (NOE)	-4.64	SILAC	22918226
BPNT1	head and neck cancer	neoplastic (FaDu, UTSCC8, UTSCC42a) and non-neoplastic (NOE)	6.16	SILAC	22918226
BPNT1	head and neck cancer	neoplastic (FaDu, UTSCC8, UTSCC42a) and non-neoplastic (NOE)	6.33	SILAC	22918226
C3	head and neck cancer	neoplastic (FaDu, UTSCC8, UTSCC42a) and non-neoplastic (NOE)	2.38	SILAC	22918226

C3	head and neck cancer	neoplastic (FaDu, UTSCC8, UTSCC42a) and non-neoplastic (NOE)	2.80	SILAC	22918226
C3	head and neck cancer	neoplastic (FaDu, UTSCC8, UTSCC42a) and non-neoplastic (NOE)	6.85	SILAC	22918226
C3	head and neck cancer	neoplastic (FaDu, UTSCC8, UTSCC42a) and non-neoplastic (NOE)	7.00	SILAC	22918226
C3	head and neck cancer	neoplastic (FaDu, UTSCC8, UTSCC42a) and non-neoplastic (NOE)	8.03	SILAC	22918226
C3	head and neck cancer	neoplastic (FaDu, UTSCC8, UTSCC42a) and non-neoplastic (NOE)	8.58	SILAC	22918226
CAPG	head and neck cancer	neoplastic (FaDu, UTSCC8, UTSCC42a) and non-neoplastic (NOE)	2.29	SILAC	22918226
CAPG	head and neck cancer	neoplastic (FaDu, UTSCC8, UTSCC42a) and non-neoplastic (NOE)	3.06	SILAC	22918226
CAPG	head and neck cancer	neoplastic (FaDu, UTSCC8, UTSCC42a) and non-neoplastic (NOE)	3.20	SILAC	22918226
CAPG	head and neck cancer	neoplastic (FaDu, UTSCC8, UTSCC42a) and non-neoplastic (NOE)	4.14	SILAC	22918226
CAPG	head and neck cancer	neoplastic (FaDu, UTSCC8, UTSCC42a) and non-neoplastic (NOE)	4.20	SILAC	22918226
CBR1	head and neck cancer	neoplastic (FaDu, UTSCC8, UTSCC42a) and non-neoplastic (NOE)	1.06	SILAC	22918226
CBR1	head and neck cancer	neoplastic (FaDu, UTSCC8, UTSCC42a) and non-neoplastic (NOE)	1.08	SILAC	22918226
CBR1	head and neck cancer	neoplastic (FaDu, UTSCC8, UTSCC42a) and non-neoplastic (NOE)	2.45	SILAC	22918226
CBR1	head and neck cancer	neoplastic (FaDu, UTSCC8, UTSCC42a) and non-neoplastic (NOE)	3.51	SILAC	22918226
CBR1	head and neck cancer	neoplastic (FaDu, UTSCC8, UTSCC42a) and non-neoplastic (NOE)	6.31	SILAC	22918226
CD109	head and neck cancer	neoplastic (FaDu, UTSCC8, UTSCC42a) and non-neoplastic (NOE)	1.76	SILAC	22918226
CD109	head and neck cancer	neoplastic (FaDu, UTSCC8, UTSCC42a) and non-neoplastic (NOE)	2.18	SILAC	22918226
CD109	head and neck cancer	neoplastic (FaDu, UTSCC8, UTSCC42a) and non-neoplastic (NOE)	2.55	SILAC	22918226
CD109	head and neck cancer	neoplastic (FaDu, UTSCC8, UTSCC42a) and non-neoplastic (NOE)	5.89	SILAC	22918226
CD109	head and neck cancer	neoplastic (FaDu, UTSCC8, UTSCC42a) and non-neoplastic (NOE)	5.90	SILAC	22918226
CDH3	head and neck cancer	neoplastic (FaDu, UTSCC8, UTSCC42a) and non-neoplastic (NOE)	2.37	SILAC	22918226
CDH3	head and neck cancer	neoplastic (FaDu, UTSCC8, UTSCC42a) and non-neoplastic (NOE)	5.75	SILAC	22918226
CDH3	head and neck cancer	neoplastic (FaDu, UTSCC8, UTSCC42a) and non-neoplastic (NOE)	5.96	SILAC	22918226
CDH3	head and neck cancer	neoplastic (FaDu, UTSCC8, UTSCC42a) and non-neoplastic (NOE)	6.59	SILAC	22918226
CDH3	head and neck cancer	neoplastic (FaDu, UTSCC8, UTSCC42a) and non-neoplastic (NOE)	6.74	SILAC	22918226
CFB	head and neck cancer	neoplastic (FaDu, UTSCC8, UTSCC42a) and non-neoplastic (NOE)	1.53	SILAC	22918226
CFB	head and neck cancer	neoplastic (FaDu, UTSCC8, UTSCC42a) and non-neoplastic (NOE)	2.69	SILAC	22918226
CFB	head and neck cancer	neoplastic (FaDu, UTSCC8, UTSCC42a) and non-neoplastic (NOE)	6.17	SILAC	22918226
CFB	head and neck cancer	neoplastic (FaDu, UTSCC8, UTSCC42a) and non-neoplastic (NOE)	6.31	SILAC	22918226
CFB	head and neck cancer	neoplastic (FaDu, UTSCC8, UTSCC42a) and non-neoplastic (NOE)	6.35	SILAC	22918226
CFB	head and neck cancer	neoplastic (FaDu, UTSCC8, UTSCC42a) and non-neoplastic (NOE)	6.49	SILAC	22918226
COL1A2	head and neck cancer	neoplastic (FaDu, UTSCC8, UTSCC42a) and non-neoplastic (NOE)	1.98	SILAC	22918226
COL1A2	head and neck cancer	neoplastic (FaDu, UTSCC8, UTSCC42a) and non-neoplastic (NOE)	3.01	SILAC	22918226
COL1A2	head and neck cancer	neoplastic (FaDu, UTSCC8, UTSCC42a) and non-neoplastic (NOE)	4.73	SILAC	22918226
COL1A2	head and neck cancer	neoplastic (FaDu, UTSCC8, UTSCC42a) and non-neoplastic (NOE)	5.89	SILAC	22918226
COL1A2	head and neck cancer	neoplastic (FaDu, UTSCC8, UTSCC42a) and non-neoplastic (NOE)	6.12	SILAC	22918226
CST6	head and neck cancer	neoplastic (FaDu, UTSCC8, UTSCC42a) and non-neoplastic (NOE)	1.53	SILAC	22918226
CST6	head and neck cancer	neoplastic (FaDu, UTSCC8, UTSCC42a) and non-neoplastic (NOE)	11.39	SILAC	22918226
CST6	head and neck cancer	neoplastic (FaDu, UTSCC8, UTSCC42a) and non-neoplastic (NOE)	11.80	SILAC	22918226
CST6	head and neck cancer	neoplastic (FaDu, UTSCC8, UTSCC42a) and non-neoplastic (NOE)	12.82	SILAC	22918226
CST6	head and neck cancer	neoplastic (FaDu, UTSCC8, UTSCC42a) and non-neoplastic (NOE)	6.15	SILAC	22918226
CST6	head and neck cancer	neoplastic (FaDu, UTSCC8, UTSCC42a) and non-neoplastic (NOE)	6.16	SILAC	22918226
CTSZ	head and neck cancer	neoplastic (FaDu, UTSCC8, UTSCC42a) and non-neoplastic (NOE)	2.35	SILAC	22918226

CTSZ	head and neck cancer	neoplastic (FaDu, UTSCC8, UTSCC42a) and non-neoplastic (NOE)	3.30	SILAC	22918226
CTSZ	head and neck cancer	neoplastic (FaDu, UTSCC8, UTSCC42a) and non-neoplastic (NOE)	3.55	SILAC	22918226
CTSZ	head and neck cancer	neoplastic (FaDu, UTSCC8, UTSCC42a) and non-neoplastic (NOE)	4.42	SILAC	22918226
CTSZ	head and neck cancer	neoplastic (FaDu, UTSCC8, UTSCC42a) and non-neoplastic (NOE)	6.59	SILAC	22918226
CTSZ	head and neck cancer	neoplastic (FaDu, UTSCC8, UTSCC42a) and non-neoplastic (NOE)	6.89	SILAC	22918226
DAG1	head and neck cancer	neoplastic (FaDu, UTSCC8, UTSCC42a) and non-neoplastic (NOE)	-1.12	SILAC	22918226
DAG1	head and neck cancer	neoplastic (FaDu, UTSCC8, UTSCC42a) and non-neoplastic (NOE)	1.61	SILAC	22918226
DAG1	head and neck cancer	neoplastic (FaDu, UTSCC8, UTSCC42a) and non-neoplastic (NOE)	2.13	SILAC	22918226
DAG1	head and neck cancer	neoplastic (FaDu, UTSCC8, UTSCC42a) and non-neoplastic (NOE)	6.29	SILAC	22918226
DAG1	head and neck cancer	neoplastic (FaDu, UTSCC8, UTSCC42a) and non-neoplastic (NOE)	6.72	SILAC	22918226
DAG1	head and neck cancer	neoplastic (FaDu, UTSCC8, UTSCC42a) and non-neoplastic (NOE)	6.86	SILAC	22918226
DKK1	head and neck cancer	neoplastic (FaDu, UTSCC8, UTSCC42a) and non-neoplastic (NOE)	1.53	SILAC	22918226
DKK1	head and neck cancer	neoplastic (FaDu, UTSCC8, UTSCC42a) and non-neoplastic (NOE)	5.68	SILAC	22918226
DKK1	head and neck cancer	neoplastic (FaDu, UTSCC8, UTSCC42a) and non-neoplastic (NOE)	6.09	SILAC	22918226
DKK1	head and neck cancer	neoplastic (FaDu, UTSCC8, UTSCC42a) and non-neoplastic (NOE)	6.57	SILAC	22918226
DKK1	head and neck cancer	neoplastic (FaDu, UTSCC8, UTSCC42a) and non-neoplastic (NOE)	6.57	SILAC	22918226
DKK1	head and neck cancer	neoplastic (FaDu, UTSCC8, UTSCC42a) and non-neoplastic (NOE)	7.33	SILAC	22918226
EXT1	head and neck cancer	neoplastic (FaDu, UTSCC8, UTSCC42a) and non-neoplastic (NOE)	1.24	SILAC	22918226
EXT1	head and neck cancer	neoplastic (FaDu, UTSCC8, UTSCC42a) and non-neoplastic (NOE)	2.35	SILAC	22918226
EXT1	head and neck cancer	neoplastic (FaDu, UTSCC8, UTSCC42a) and non-neoplastic (NOE)	2.80	SILAC	22918226
EXT1	head and neck cancer	neoplastic (FaDu, UTSCC8, UTSCC42a) and non-neoplastic (NOE)	5.97	SILAC	22918226
EXT1	head and neck cancer	neoplastic (FaDu, UTSCC8, UTSCC42a) and non-neoplastic (NOE)	6.16	SILAC	22918226
EXT1	head and neck cancer	neoplastic (FaDu, UTSCC8, UTSCC42a) and non-neoplastic (NOE)	6.18	SILAC	22918226
EZR	head and neck cancer	neoplastic (FaDu, UTSCC8, UTSCC42a) and non-neoplastic (NOE)	1.54	SILAC	22918226
EZR	head and neck cancer	neoplastic (FaDu, UTSCC8, UTSCC42a) and non-neoplastic (NOE)	1.60	SILAC	22918226
EZR	head and neck cancer	neoplastic (FaDu, UTSCC8, UTSCC42a) and non-neoplastic (NOE)	1.91	SILAC	22918226
EZR	head and neck cancer	neoplastic (FaDu, UTSCC8, UTSCC42a) and non-neoplastic (NOE)	2.23	SILAC	22918226
EZR	head and neck cancer	neoplastic (FaDu, UTSCC8, UTSCC42a) and non-neoplastic (NOE)	2.39	SILAC	22918226
EZR	head and neck cancer	neoplastic (FaDu, UTSCC8, UTSCC42a) and non-neoplastic (NOE)	2.74	SILAC	22918226
F5	head and neck cancer	neoplastic (FaDu, UTSCC8, UTSCC42a) and non-neoplastic (NOE)	1.84	SILAC	22918226
F5	head and neck cancer	neoplastic (FaDu, UTSCC8, UTSCC42a) and non-neoplastic (NOE)	1.93	SILAC	22918226
F5	head and neck cancer	neoplastic (FaDu, UTSCC8, UTSCC42a) and non-neoplastic (NOE)	2.19	SILAC	22918226
F5	head and neck cancer	neoplastic (FaDu, UTSCC8, UTSCC42a) and non-neoplastic (NOE)	5.79	SILAC	22918226
F5	head and neck cancer	neoplastic (FaDu, UTSCC8, UTSCC42a) and non-neoplastic (NOE)	5.87	SILAC	22918226
FABP5	head and neck cancer	neoplastic (FaDu, UTSCC8, UTSCC42a) and non-neoplastic (NOE)	2.11	SILAC	22918226
FABP5	head and neck cancer	neoplastic (FaDu, UTSCC8, UTSCC42a) and non-neoplastic (NOE)	3.19	SILAC	22918226
FABP5	head and neck cancer	neoplastic (FaDu, UTSCC8, UTSCC42a) and non-neoplastic (NOE)	3.36	SILAC	22918226
FABP5	head and neck cancer	neoplastic (FaDu, UTSCC8, UTSCC42a) and non-neoplastic (NOE)	3.53	SILAC	22918226
FABP5	head and neck cancer	neoplastic (FaDu, UTSCC8, UTSCC42a) and non-neoplastic (NOE)	3.64	SILAC	22918226
FABP5	head and neck cancer	neoplastic (FaDu, UTSCC8, UTSCC42a) and non-neoplastic (NOE)	4.13	SILAC	22918226
FAM129B	head and neck cancer	neoplastic (FaDu, UTSCC8, UTSCC42a) and non-neoplastic (NOE)	1.95	SILAC	22918226
FAM129B	head and neck cancer	neoplastic (FaDu, UTSCC8, UTSCC42a) and non-neoplastic (NOE)	3.39	SILAC	22918226
FAM129B	head and neck cancer	neoplastic (FaDu, UTSCC8, UTSCC42a) and non-neoplastic (NOE)	3.52	SILAC	22918226

FAM129B	head and neck cancer	neoplastic (FaDu, UTSCC8, UTSCC42a) and non-neoplastic (NOE)	4.22	SILAC	22918226
FAM129B	head and neck cancer	neoplastic (FaDu, UTSCC8, UTSCC42a) and non-neoplastic (NOE)	5.64	SILAC	22918226
FAM129B	head and neck cancer	neoplastic (FaDu, UTSCC8, UTSCC42a) and non-neoplastic (NOE)	6.35	SILAC	22918226
FAT2	head and neck cancer	neoplastic (FaDu, UTSCC8, UTSCC42a) and non-neoplastic (NOE)	5.02	SILAC	22918226
FAT2	head and neck cancer	neoplastic (FaDu, UTSCC8, UTSCC42a) and non-neoplastic (NOE)	6.29	SILAC	22918226
FAT2	head and neck cancer	neoplastic (FaDu, UTSCC8, UTSCC42a) and non-neoplastic (NOE)	6.38	SILAC	22918226
FAT2	head and neck cancer	neoplastic (FaDu, UTSCC8, UTSCC42a) and non-neoplastic (NOE)	6.70	SILAC	22918226
FAT2	head and neck cancer	neoplastic (FaDu, UTSCC8, UTSCC42a) and non-neoplastic (NOE)	7.04	SILAC	22918226
FAT2	head and neck cancer	neoplastic (FaDu, UTSCC8, UTSCC42a) and non-neoplastic (NOE)	7.05	SILAC	22918226
FGFBP1	head and neck cancer	neoplastic (FaDu, UTSCC8, UTSCC42a) and non-neoplastic (NOE)	6.50	SILAC	22918226
FGFBP1	head and neck cancer	neoplastic (FaDu, UTSCC8, UTSCC42a) and non-neoplastic (NOE)	6.63	SILAC	22918226
FGFBP1	head and neck cancer	neoplastic (FaDu, UTSCC8, UTSCC42a) and non-neoplastic (NOE)	7.15	SILAC	22918226
FGFBP1	head and neck cancer	neoplastic (FaDu, UTSCC8, UTSCC42a) and non-neoplastic (NOE)	7.85	SILAC	22918226
FGFBP1	head and neck cancer	neoplastic (FaDu, UTSCC8, UTSCC42a) and non-neoplastic (NOE)	8.84	SILAC	22918226
FN1	head and neck cancer	neoplastic (FaDu, UTSCC8, UTSCC42a) and non-neoplastic (NOE)	-2.47	SILAC	22918226
FN1	head and neck cancer	neoplastic (FaDu, UTSCC8, UTSCC42a) and non-neoplastic (NOE)	2.95	SILAC	22918226
FN1	head and neck cancer	neoplastic (FaDu, UTSCC8, UTSCC42a) and non-neoplastic (NOE)	4.45	SILAC	22918226
FN1	head and neck cancer	neoplastic (FaDu, UTSCC8, UTSCC42a) and non-neoplastic (NOE)	6.63	SILAC	22918226
FN1	head and neck cancer	neoplastic (FaDu, UTSCC8, UTSCC42a) and non-neoplastic (NOE)	6.72	SILAC	22918226
FN1	head and neck cancer	neoplastic (FaDu, UTSCC8, UTSCC42a) and non-neoplastic (NOE)	7.02	SILAC	22918226
GCLC	head and neck cancer	neoplastic (FaDu, UTSCC8, UTSCC42a) and non-neoplastic (NOE)	1.53	SILAC	22918226
GCLC	head and neck cancer	neoplastic (FaDu, UTSCC8, UTSCC42a) and non-neoplastic (NOE)	1.86	SILAC	22918226
GCLC	head and neck cancer	neoplastic (FaDu, UTSCC8, UTSCC42a) and non-neoplastic (NOE)	2.45	SILAC	22918226
GCLC	head and neck cancer	neoplastic (FaDu, UTSCC8, UTSCC42a) and non-neoplastic (NOE)	4.89	SILAC	22918226
GCLC	head and neck cancer	neoplastic (FaDu, UTSCC8, UTSCC42a) and non-neoplastic (NOE)	5.89	SILAC	22918226
GCLC	head and neck cancer	neoplastic (FaDu, UTSCC8, UTSCC42a) and non-neoplastic (NOE)	6.31	SILAC	22918226
GPC1	head and neck cancer	neoplastic (FaDu, UTSCC8, UTSCC42a) and non-neoplastic (NOE)	-1.94	SILAC	22918226
GPC1	head and neck cancer	neoplastic (FaDu, UTSCC8, UTSCC42a) and non-neoplastic (NOE)	2.42	SILAC	22918226
GPC1	head and neck cancer	neoplastic (FaDu, UTSCC8, UTSCC42a) and non-neoplastic (NOE)	2.65	SILAC	22918226
GPC1	head and neck cancer	neoplastic (FaDu, UTSCC8, UTSCC42a) and non-neoplastic (NOE)	3.87	SILAC	22918226
GPC1	head and neck cancer	neoplastic (FaDu, UTSCC8, UTSCC42a) and non-neoplastic (NOE)	3.95	SILAC	22918226
GPC1	head and neck cancer	neoplastic (FaDu, UTSCC8, UTSCC42a) and non-neoplastic (NOE)	6.77	SILAC	22918226
HDGF	head and neck cancer	neoplastic (FaDu, UTSCC8, UTSCC42a) and non-neoplastic (NOE)	1.32	SILAC	22918226
HDGF	head and neck cancer	neoplastic (FaDu, UTSCC8, UTSCC42a) and non-neoplastic (NOE)	1.37	SILAC	22918226
HDGF	head and neck cancer	neoplastic (FaDu, UTSCC8, UTSCC42a) and non-neoplastic (NOE)	2.43	SILAC	22918226
HDGF	head and neck cancer	neoplastic (FaDu, UTSCC8, UTSCC42a) and non-neoplastic (NOE)	3.00	SILAC	22918226
HDGF	head and neck cancer	neoplastic (FaDu, UTSCC8, UTSCC42a) and non-neoplastic (NOE)	3.80	SILAC	22918226
HMGA1	head and neck cancer	neoplastic (FaDu, UTSCC8, UTSCC42a) and non-neoplastic (NOE)	3.63	SILAC	22918226
HMGA1	head and neck cancer	neoplastic (FaDu, UTSCC8, UTSCC42a) and non-neoplastic (NOE)	3.80	SILAC	22918226
HMGA1	head and neck cancer	neoplastic (FaDu, UTSCC8, UTSCC42a) and non-neoplastic (NOE)	3.90	SILAC	22918226
HMGA1	head and neck cancer	neoplastic (FaDu, UTSCC8, UTSCC42a) and non-neoplastic (NOE)	6.35	SILAC	22918226
HMGA1	head and neck cancer	neoplastic (FaDu, UTSCC8, UTSCC42a) and non-neoplastic (NOE)	6.47	SILAC	22918226
HMGB1L1	head and neck cancer	neoplastic (FaDu, UTSCC8, UTSCC42a) and non-neoplastic (NOE)	1.02	SILAC	22918226

HMGB1L1	head and neck cancer	neoplastic (FaDu, UTSCC8, UTSCC42a) and non-neoplastic (NOE)	1.48	SILAC	22918226
HMGB1L1	head and neck cancer	neoplastic (FaDu, UTSCC8, UTSCC42a) and non-neoplastic (NOE)	2.32	SILAC	22918226
HMGB1L1	head and neck cancer	neoplastic (FaDu, UTSCC8, UTSCC42a) and non-neoplastic (NOE)	6.01	SILAC	22918226
HMGB1L1	head and neck cancer	neoplastic (FaDu, UTSCC8, UTSCC42a) and non-neoplastic (NOE)	6.38	SILAC	22918226
HN1	head and neck cancer	neoplastic (FaDu, UTSCC8, UTSCC42a) and non-neoplastic (NOE)	1.36	SILAC	22918226
HN1	head and neck cancer	neoplastic (FaDu, UTSCC8, UTSCC42a) and non-neoplastic (NOE)	1.49	SILAC	22918226
HN1	head and neck cancer	neoplastic (FaDu, UTSCC8, UTSCC42a) and non-neoplastic (NOE)	1.58	SILAC	22918226
HN1	head and neck cancer	neoplastic (FaDu, UTSCC8, UTSCC42a) and non-neoplastic (NOE)	6.28	SILAC	22918226
HN1	head and neck cancer	neoplastic (FaDu, UTSCC8, UTSCC42a) and non-neoplastic (NOE)	6.37	SILAC	22918226
HN1	head and neck cancer	neoplastic (FaDu, UTSCC8, UTSCC42a) and non-neoplastic (NOE)	6.46	SILAC	22918226
HN1L	head and neck cancer	neoplastic (FaDu, UTSCC8, UTSCC42a) and non-neoplastic (NOE)	1.05	SILAC	22918226
HN1L	head and neck cancer	neoplastic (FaDu, UTSCC8, UTSCC42a) and non-neoplastic (NOE)	1.10	SILAC	22918226
HN1L	head and neck cancer	neoplastic (FaDu, UTSCC8, UTSCC42a) and non-neoplastic (NOE)	1.73	SILAC	22918226
HN1L	head and neck cancer	neoplastic (FaDu, UTSCC8, UTSCC42a) and non-neoplastic (NOE)	2.86	SILAC	22918226
HN1L	head and neck cancer	neoplastic (FaDu, UTSCC8, UTSCC42a) and non-neoplastic (NOE)	6.40	SILAC	22918226
HN1L	head and neck cancer	neoplastic (FaDu, UTSCC8, UTSCC42a) and non-neoplastic (NOE)	6.42	SILAC	22918226
HSPA2	head and neck cancer	neoplastic (FaDu, UTSCC8, UTSCC42a) and non-neoplastic (NOE)	1.14	SILAC	22918226
HSPA2	head and neck cancer	neoplastic (FaDu, UTSCC8, UTSCC42a) and non-neoplastic (NOE)	1.66	SILAC	22918226
HSPA2	head and neck cancer	neoplastic (FaDu, UTSCC8, UTSCC42a) and non-neoplastic (NOE)	2.31	SILAC	22918226
HSPA2	head and neck cancer	neoplastic (FaDu, UTSCC8, UTSCC42a) and non-neoplastic (NOE)	7.25	SILAC	22918226
HSPA2	head and neck cancer	neoplastic (FaDu, UTSCC8, UTSCC42a) and non-neoplastic (NOE)	7.38	SILAC	22918226
HSPG2	head and neck cancer	neoplastic (FaDu, UTSCC8, UTSCC42a) and non-neoplastic (NOE)	1.77	SILAC	22918226
HSPG2	head and neck cancer	neoplastic (FaDu, UTSCC8, UTSCC42a) and non-neoplastic (NOE)	3.64	SILAC	22918226
HSPG2	head and neck cancer	neoplastic (FaDu, UTSCC8, UTSCC42a) and non-neoplastic (NOE)	4.20	SILAC	22918226
HSPG2	head and neck cancer	neoplastic (FaDu, UTSCC8, UTSCC42a) and non-neoplastic (NOE)	4.59	SILAC	22918226
HSPG2	head and neck cancer	neoplastic (FaDu, UTSCC8, UTSCC42a) and non-neoplastic (NOE)	6.37	SILAC	22918226
HSPG2	head and neck cancer	neoplastic (FaDu, UTSCC8, UTSCC42a) and non-neoplastic (NOE)	6.81	SILAC	22918226
IGFBP3	head and neck cancer	neoplastic (FaDu, UTSCC8, UTSCC42a) and non-neoplastic (NOE)	11.81	SILAC	22918226
IGFBP3	head and neck cancer	neoplastic (FaDu, UTSCC8, UTSCC42a) and non-neoplastic (NOE)	12.33	SILAC	22918226
IGFBP3	head and neck cancer	neoplastic (FaDu, UTSCC8, UTSCC42a) and non-neoplastic (NOE)	2.95	SILAC	22918226
IGFBP3	head and neck cancer	neoplastic (FaDu, UTSCC8, UTSCC42a) and non-neoplastic (NOE)	6.04	SILAC	22918226
IGFBP3	head and neck cancer	neoplastic (FaDu, UTSCC8, UTSCC42a) and non-neoplastic (NOE)	6.93	SILAC	22918226
IGFBP6	head and neck cancer	neoplastic (FaDu, UTSCC8, UTSCC42a) and non-neoplastic (NOE)	6.29	SILAC	22918226
IGFBP6	head and neck cancer	neoplastic (FaDu, UTSCC8, UTSCC42a) and non-neoplastic (NOE)	6.51	SILAC	22918226
IGFBP6	head and neck cancer	neoplastic (FaDu, UTSCC8, UTSCC42a) and non-neoplastic (NOE)	6.51	SILAC	22918226
IGFBP6	head and neck cancer	neoplastic (FaDu, UTSCC8, UTSCC42a) and non-neoplastic (NOE)	6.65	SILAC	22918226
IGFBP6	head and neck cancer	neoplastic (FaDu, UTSCC8, UTSCC42a) and non-neoplastic (NOE)	6.80	SILAC	22918226
IGFBP6	head and neck cancer	neoplastic (FaDu, UTSCC8, UTSCC42a) and non-neoplastic (NOE)	6.84	SILAC	22918226
IGFBP7	head and neck cancer	neoplastic (FaDu, UTSCC8, UTSCC42a) and non-neoplastic (NOE)	1.97	SILAC	22918226
IGFBP7	head and neck cancer	neoplastic (FaDu, UTSCC8, UTSCC42a) and non-neoplastic (NOE)	2.77	SILAC	22918226
IGFBP7	head and neck cancer	neoplastic (FaDu, UTSCC8, UTSCC42a) and non-neoplastic (NOE)	3.86	SILAC	22918226
IGFBP7	head and neck cancer	neoplastic (FaDu, UTSCC8, UTSCC42a) and non-neoplastic (NOE)	6.37	SILAC	22918226
IGFBP7	head and neck cancer	neoplastic (FaDu, UTSCC8, UTSCC42a) and non-neoplastic (NOE)	6.68	SILAC	22918226

IGFBP7	head and neck cancer	neoplastic (FaDu, UTSCC8, UTSCC42a) and non-neoplastic (NOE)	6.83	SILAC	22918226
ITIH2	head and neck cancer	neoplastic (FaDu, UTSCC8, UTSCC42a) and non-neoplastic (NOE)	2.02	SILAC	22918226
ITIH2	head and neck cancer	neoplastic (FaDu, UTSCC8, UTSCC42a) and non-neoplastic (NOE)	2.20	SILAC	22918226
ITIH2	head and neck cancer	neoplastic (FaDu, UTSCC8, UTSCC42a) and non-neoplastic (NOE)	7.14	SILAC	22918226
ITIH2	head and neck cancer	neoplastic (FaDu, UTSCC8, UTSCC42a) and non-neoplastic (NOE)	7.18	SILAC	22918226
ITIH2	head and neck cancer	neoplastic (FaDu, UTSCC8, UTSCC42a) and non-neoplastic (NOE)	7.38	SILAC	22918226
ITM2B	head and neck cancer	neoplastic (FaDu, UTSCC8, UTSCC42a) and non-neoplastic (NOE)	1.22	SILAC	22918226
ITM2B	head and neck cancer	neoplastic (FaDu, UTSCC8, UTSCC42a) and non-neoplastic (NOE)	1.53	SILAC	22918226
ITM2B	head and neck cancer	neoplastic (FaDu, UTSCC8, UTSCC42a) and non-neoplastic (NOE)	1.67	SILAC	22918226
ITM2B	head and neck cancer	neoplastic (FaDu, UTSCC8, UTSCC42a) and non-neoplastic (NOE)	2.49	SILAC	22918226
ITM2B	head and neck cancer	neoplastic (FaDu, UTSCC8, UTSCC42a) and non-neoplastic (NOE)	6.24	SILAC	22918226
ITM2B	head and neck cancer	neoplastic (FaDu, UTSCC8, UTSCC42a) and non-neoplastic (NOE)	6.60	SILAC	22918226
KLK10	head and neck cancer	neoplastic (FaDu, UTSCC8, UTSCC42a) and non-neoplastic (NOE)	6.32	SILAC	22918226
KLK10	head and neck cancer	neoplastic (FaDu, UTSCC8, UTSCC42a) and non-neoplastic (NOE)	6.45	SILAC	22918226
KLK10	head and neck cancer	neoplastic (FaDu, UTSCC8, UTSCC42a) and non-neoplastic (NOE)	6.69	SILAC	22918226
KLK10	head and neck cancer	neoplastic (FaDu, UTSCC8, UTSCC42a) and non-neoplastic (NOE)	7.44	SILAC	22918226
KLK10	head and neck cancer	neoplastic (FaDu, UTSCC8, UTSCC42a) and non-neoplastic (NOE)	7.93	SILAC	22918226
KLK10	head and neck cancer	neoplastic (FaDu, UTSCC8, UTSCC42a) and non-neoplastic (NOE)	8.52	SILAC	22918226
KLK6	head and neck cancer	neoplastic (FaDu, UTSCC8, UTSCC42a) and non-neoplastic (NOE)	4.59	SILAC	22918226
KLK6	head and neck cancer	neoplastic (FaDu, UTSCC8, UTSCC42a) and non-neoplastic (NOE)	4.75	SILAC	22918226
KLK6	head and neck cancer	neoplastic (FaDu, UTSCC8, UTSCC42a) and non-neoplastic (NOE)	6.97	SILAC	22918226
KLK6	head and neck cancer	neoplastic (FaDu, UTSCC8, UTSCC42a) and non-neoplastic (NOE)	7.21	SILAC	22918226
KLK6	head and neck cancer	neoplastic (FaDu, UTSCC8, UTSCC42a) and non-neoplastic (NOE)	7.74	SILAC	22918226
KRT2	head and neck cancer	neoplastic (FaDu, UTSCC8, UTSCC42a) and non-neoplastic (NOE)	1.18	SILAC	22918226
KRT2	head and neck cancer	neoplastic (FaDu, UTSCC8, UTSCC42a) and non-neoplastic (NOE)	1.97	SILAC	22918226
KRT2	head and neck cancer	neoplastic (FaDu, UTSCC8, UTSCC42a) and non-neoplastic (NOE)	2.43	SILAC	22918226
KRT2	head and neck cancer	neoplastic (FaDu, UTSCC8, UTSCC42a) and non-neoplastic (NOE)	6.93	SILAC	22918226
KRT2	head and neck cancer	neoplastic (FaDu, UTSCC8, UTSCC42a) and non-neoplastic (NOE)	6.94	SILAC	22918226
KRT7	head and neck cancer	neoplastic (FaDu, UTSCC8, UTSCC42a) and non-neoplastic (NOE)	1.53	SILAC	22918226
KRT7	head and neck cancer	neoplastic (FaDu, UTSCC8, UTSCC42a) and non-neoplastic (NOE)	3.39	SILAC	22918226
KRT7	head and neck cancer	neoplastic (FaDu, UTSCC8, UTSCC42a) and non-neoplastic (NOE)	4.41	SILAC	22918226
KRT7	head and neck cancer	neoplastic (FaDu, UTSCC8, UTSCC42a) and non-neoplastic (NOE)	4.77	SILAC	22918226
KRT7	head and neck cancer	neoplastic (FaDu, UTSCC8, UTSCC42a) and non-neoplastic (NOE)	6.65	SILAC	22918226
KRT7	head and neck cancer	neoplastic (FaDu, UTSCC8, UTSCC42a) and non-neoplastic (NOE)	7.07	SILAC	22918226
KRT9	head and neck cancer	neoplastic (FaDu, UTSCC8, UTSCC42a) and non-neoplastic (NOE)	2.25	SILAC	22918226
KRT9	head and neck cancer	neoplastic (FaDu, UTSCC8, UTSCC42a) and non-neoplastic (NOE)	3.00	SILAC	22918226
KRT9	head and neck cancer	neoplastic (FaDu, UTSCC8, UTSCC42a) and non-neoplastic (NOE)	3.90	SILAC	22918226
KRT9	head and neck cancer	neoplastic (FaDu, UTSCC8, UTSCC42a) and non-neoplastic (NOE)	6.03	SILAC	22918226
KRT9	head and neck cancer	neoplastic (FaDu, UTSCC8, UTSCC42a) and non-neoplastic (NOE)	6.61	SILAC	22918226
KRT9	head and neck cancer	neoplastic (FaDu, UTSCC8, UTSCC42a) and non-neoplastic (NOE)	6.87	SILAC	22918226
LAMA5	head and neck cancer	neoplastic (FaDu, UTSCC8, UTSCC42a) and non-neoplastic (NOE)	3.47	SILAC	22918226
LAMA5	head and neck cancer	neoplastic (FaDu, UTSCC8, UTSCC42a) and non-neoplastic (NOE)	3.55	SILAC	22918226
LAMA5	head and neck cancer	neoplastic (FaDu, UTSCC8, UTSCC42a) and non-neoplastic (NOE)	6.57	SILAC	22918226

LAMA5	head and neck cancer	neoplastic (FaDu, UTSCC8, UTSCC42a) and non-neoplastic (NOE)	6.57	SILAC	22918226
LAMA5	head and neck cancer	neoplastic (FaDu, UTSCC8, UTSCC42a) and non-neoplastic (NOE)	6.84	SILAC	22918226
LAMB3	head and neck cancer	neoplastic (FaDu, UTSCC8, UTSCC42a) and non-neoplastic (NOE)	3.52	SILAC	22918226
LAMB3	head and neck cancer	neoplastic (FaDu, UTSCC8, UTSCC42a) and non-neoplastic (NOE)	4.06	SILAC	22918226
LAMB3	head and neck cancer	neoplastic (FaDu, UTSCC8, UTSCC42a) and non-neoplastic (NOE)	4.14	SILAC	22918226
LAMB3	head and neck cancer	neoplastic (FaDu, UTSCC8, UTSCC42a) and non-neoplastic (NOE)	4.45	SILAC	22918226
LAMB3	head and neck cancer	neoplastic (FaDu, UTSCC8, UTSCC42a) and non-neoplastic (NOE)	6.28	SILAC	22918226
LAMC2	head and neck cancer	neoplastic (FaDu, UTSCC8, UTSCC42a) and non-neoplastic (NOE)	3.03	SILAC	22918226
LAMC2	head and neck cancer	neoplastic (FaDu, UTSCC8, UTSCC42a) and non-neoplastic (NOE)	4.85	SILAC	22918226
LAMC2	head and neck cancer	neoplastic (FaDu, UTSCC8, UTSCC42a) and non-neoplastic (NOE)	5.69	SILAC	22918226
LAMC2	head and neck cancer	neoplastic (FaDu, UTSCC8, UTSCC42a) and non-neoplastic (NOE)	6.43	SILAC	22918226
LAMC2	head and neck cancer	neoplastic (FaDu, UTSCC8, UTSCC42a) and non-neoplastic (NOE)	6.62	SILAC	22918226
LAMC2	head and neck cancer	neoplastic (FaDu, UTSCC8, UTSCC42a) and non-neoplastic (NOE)	7.07	SILAC	22918226
LASP1	head and neck cancer	neoplastic (FaDu, UTSCC8, UTSCC42a) and non-neoplastic (NOE)	2.26	SILAC	22918226
LASP1	head and neck cancer	neoplastic (FaDu, UTSCC8, UTSCC42a) and non-neoplastic (NOE)	2.32	SILAC	22918226
LASP1	head and neck cancer	neoplastic (FaDu, UTSCC8, UTSCC42a) and non-neoplastic (NOE)	2.61	SILAC	22918226
LASP1	head and neck cancer	neoplastic (FaDu, UTSCC8, UTSCC42a) and non-neoplastic (NOE)	3.44	SILAC	22918226
LASP1	head and neck cancer	neoplastic (FaDu, UTSCC8, UTSCC42a) and non-neoplastic (NOE)	3.87	SILAC	22918226
LASP1	head and neck cancer	neoplastic (FaDu, UTSCC8, UTSCC42a) and non-neoplastic (NOE)	4.20	SILAC	22918226
LMNA	head and neck cancer	neoplastic (FaDu, UTSCC8, UTSCC42a) and non-neoplastic (NOE)	1.39	SILAC	22918226
LMNA	head and neck cancer	neoplastic (FaDu, UTSCC8, UTSCC42a) and non-neoplastic (NOE)	1.63	SILAC	22918226
LMNA	head and neck cancer	neoplastic (FaDu, UTSCC8, UTSCC42a) and non-neoplastic (NOE)	1.78	SILAC	22918226
LMNA	head and neck cancer	neoplastic (FaDu, UTSCC8, UTSCC42a) and non-neoplastic (NOE)	2.01	SILAC	22918226
LMNA	head and neck cancer	neoplastic (FaDu, UTSCC8, UTSCC42a) and non-neoplastic (NOE)	2.69	SILAC	22918226
LMNA	head and neck cancer	neoplastic (FaDu, UTSCC8, UTSCC42a) and non-neoplastic (NOE)	2.84	SILAC	22918226
LSR	head and neck cancer	neoplastic (FaDu, UTSCC8, UTSCC42a) and non-neoplastic (NOE)	2.90	SILAC	22918226
LSR	head and neck cancer	neoplastic (FaDu, UTSCC8, UTSCC42a) and non-neoplastic (NOE)	3.70	SILAC	22918226
LSR	head and neck cancer	neoplastic (FaDu, UTSCC8, UTSCC42a) and non-neoplastic (NOE)	6.59	SILAC	22918226
LSR	head and neck cancer	neoplastic (FaDu, UTSCC8, UTSCC42a) and non-neoplastic (NOE)	6.61	SILAC	22918226
LSR	head and neck cancer	neoplastic (FaDu, UTSCC8, UTSCC42a) and non-neoplastic (NOE)	6.74	SILAC	22918226
LTA4H	head and neck cancer	neoplastic (FaDu, UTSCC8, UTSCC42a) and non-neoplastic (NOE)	1.06	SILAC	22918226
LTA4H	head and neck cancer	neoplastic (FaDu, UTSCC8, UTSCC42a) and non-neoplastic (NOE)	1.60	SILAC	22918226
LTA4H	head and neck cancer	neoplastic (FaDu, UTSCC8, UTSCC42a) and non-neoplastic (NOE)	2.06	SILAC	22918226
LTA4H	head and neck cancer	neoplastic (FaDu, UTSCC8, UTSCC42a) and non-neoplastic (NOE)	2.79	SILAC	22918226
LTA4H	head and neck cancer	neoplastic (FaDu, UTSCC8, UTSCC42a) and non-neoplastic (NOE)	3.51	SILAC	22918226
MAN2B1	head and neck cancer	neoplastic (FaDu, UTSCC8, UTSCC42a) and non-neoplastic (NOE)	1.55	SILAC	22918226
MAN2B1	head and neck cancer	neoplastic (FaDu, UTSCC8, UTSCC42a) and non-neoplastic (NOE)	1.85	SILAC	22918226
MAN2B1	head and neck cancer	neoplastic (FaDu, UTSCC8, UTSCC42a) and non-neoplastic (NOE)	5.65	SILAC	22918226
MAN2B1	head and neck cancer	neoplastic (FaDu, UTSCC8, UTSCC42a) and non-neoplastic (NOE)	6.53	SILAC	22918226
MAN2B1	head and neck cancer	neoplastic (FaDu, UTSCC8, UTSCC42a) and non-neoplastic (NOE)	6.70	SILAC	22918226
MATN2	head and neck cancer	neoplastic (FaDu, UTSCC8, UTSCC42a) and non-neoplastic (NOE)	3.62	SILAC	22918226
MATN2	head and neck cancer	neoplastic (FaDu, UTSCC8, UTSCC42a) and non-neoplastic (NOE)	4.15	SILAC	22918226
MATN2	head and neck cancer	neoplastic (FaDu, UTSCC8, UTSCC42a) and non-neoplastic (NOE)	5.39	SILAC	22918226

MATN2	head and neck cancer	neoplastic (FaDu, UTSCC8, UTSCC42a) and non-neoplastic (NOE)	6.27	SILAC	22918226
MATN2	head and neck cancer	neoplastic (FaDu, UTSCC8, UTSCC42a) and non-neoplastic (NOE)	6.48	SILAC	22918226
MATN2	head and neck cancer	neoplastic (FaDu, UTSCC8, UTSCC42a) and non-neoplastic (NOE)	6.76	SILAC	22918226
MDH1	head and neck cancer	neoplastic (FaDu, UTSCC8, UTSCC42a) and non-neoplastic (NOE)	1.15	SILAC	22918226
MDH1	head and neck cancer	neoplastic (FaDu, UTSCC8, UTSCC42a) and non-neoplastic (NOE)	1.38	SILAC	22918226
MDH1	head and neck cancer	neoplastic (FaDu, UTSCC8, UTSCC42a) and non-neoplastic (NOE)	1.73	SILAC	22918226
MDH1	head and neck cancer	neoplastic (FaDu, UTSCC8, UTSCC42a) and non-neoplastic (NOE)	2.55	SILAC	22918226
MDH1	head and neck cancer	neoplastic (FaDu, UTSCC8, UTSCC42a) and non-neoplastic (NOE)	2.70	SILAC	22918226
MDH1	head and neck cancer	neoplastic (FaDu, UTSCC8, UTSCC42a) and non-neoplastic (NOE)	3.12	SILAC	22918226
ME1	head and neck cancer	neoplastic (FaDu, UTSCC8, UTSCC42a) and non-neoplastic (NOE)	2.48	SILAC	22918226
ME1	head and neck cancer	neoplastic (FaDu, UTSCC8, UTSCC42a) and non-neoplastic (NOE)	3.13	SILAC	22918226
ME1	head and neck cancer	neoplastic (FaDu, UTSCC8, UTSCC42a) and non-neoplastic (NOE)	3.68	SILAC	22918226
ME1	head and neck cancer	neoplastic (FaDu, UTSCC8, UTSCC42a) and non-neoplastic (NOE)	5.16	SILAC	22918226
ME1	head and neck cancer	neoplastic (FaDu, UTSCC8, UTSCC42a) and non-neoplastic (NOE)	5.97	SILAC	22918226
ME1	head and neck cancer	neoplastic (FaDu, UTSCC8, UTSCC42a) and non-neoplastic (NOE)	6.35	SILAC	22918226
MMP13	head and neck cancer	neoplastic (FaDu, UTSCC8, UTSCC42a) and non-neoplastic (NOE)	4.66	SILAC	22918226
MMP13	head and neck cancer	neoplastic (FaDu, UTSCC8, UTSCC42a) and non-neoplastic (NOE)	5.10	SILAC	22918226
MMP13	head and neck cancer	neoplastic (FaDu, UTSCC8, UTSCC42a) and non-neoplastic (NOE)	6.48	SILAC	22918226
MMP13	head and neck cancer	neoplastic (FaDu, UTSCC8, UTSCC42a) and non-neoplastic (NOE)	6.48	SILAC	22918226
MMP13	head and neck cancer	neoplastic (FaDu, UTSCC8, UTSCC42a) and non-neoplastic (NOE)	9.24	SILAC	22918226
MMP14	head and neck cancer	neoplastic (FaDu, UTSCC8, UTSCC42a) and non-neoplastic (NOE)	2.70	SILAC	22918226
MMP14	head and neck cancer	neoplastic (FaDu, UTSCC8, UTSCC42a) and non-neoplastic (NOE)	3.94	SILAC	22918226
MMP14	head and neck cancer	neoplastic (FaDu, UTSCC8, UTSCC42a) and non-neoplastic (NOE)	5.39	SILAC	22918226
MMP14	head and neck cancer	neoplastic (FaDu, UTSCC8, UTSCC42a) and non-neoplastic (NOE)	6.06	SILAC	22918226
MMP14	head and neck cancer	neoplastic (FaDu, UTSCC8, UTSCC42a) and non-neoplastic (NOE)	6.21	SILAC	22918226
MMP14	head and neck cancer	neoplastic (FaDu, UTSCC8, UTSCC42a) and non-neoplastic (NOE)	6.52	SILAC	22918226
NUDT5	head and neck cancer	neoplastic (FaDu, UTSCC8, UTSCC42a) and non-neoplastic (NOE)	1.21	SILAC	22918226
NUDT5	head and neck cancer	neoplastic (FaDu, UTSCC8, UTSCC42a) and non-neoplastic (NOE)	1.67	SILAC	22918226
NUDT5	head and neck cancer	neoplastic (FaDu, UTSCC8, UTSCC42a) and non-neoplastic (NOE)	1.70	SILAC	22918226
NUDT5	head and neck cancer	neoplastic (FaDu, UTSCC8, UTSCC42a) and non-neoplastic (NOE)	6.18	SILAC	22918226
NUDT5	head and neck cancer	neoplastic (FaDu, UTSCC8, UTSCC42a) and non-neoplastic (NOE)	6.28	SILAC	22918226
PCSK9	head and neck cancer	neoplastic (FaDu, UTSCC8, UTSCC42a) and non-neoplastic (NOE)	10.96	SILAC	22918226
PCSK9	head and neck cancer	neoplastic (FaDu, UTSCC8, UTSCC42a) and non-neoplastic (NOE)	12.64	SILAC	22918226
PCSK9	head and neck cancer	neoplastic (FaDu, UTSCC8, UTSCC42a) and non-neoplastic (NOE)	13.36	SILAC	22918226
PCSK9	head and neck cancer	neoplastic (FaDu, UTSCC8, UTSCC42a) and non-neoplastic (NOE)	6.93	SILAC	22918226
PCSK9	head and neck cancer	neoplastic (FaDu, UTSCC8, UTSCC42a) and non-neoplastic (NOE)	7.05	SILAC	22918226
PCSK9	head and neck cancer	neoplastic (FaDu, UTSCC8, UTSCC42a) and non-neoplastic (NOE)	7.18	SILAC	22918226
PDLIM1	head and neck cancer	neoplastic (FaDu, UTSCC8, UTSCC42a) and non-neoplastic (NOE)	1.18	SILAC	22918226
PDLIM1	head and neck cancer	neoplastic (FaDu, UTSCC8, UTSCC42a) and non-neoplastic (NOE)	2.02	SILAC	22918226
PDLIM1	head and neck cancer	neoplastic (FaDu, UTSCC8, UTSCC42a) and non-neoplastic (NOE)	2.06	SILAC	22918226
PDLIM1	head and neck cancer	neoplastic (FaDu, UTSCC8, UTSCC42a) and non-neoplastic (NOE)	2.48	SILAC	22918226
PDLIM1	head and neck cancer	neoplastic (FaDu, UTSCC8, UTSCC42a) and non-neoplastic (NOE)	3.88	SILAC	22918226
PDLIM1	head and neck cancer	neoplastic (FaDu, UTSCC8, UTSCC42a) and non-neoplastic (NOE)	4.34	SILAC	22918226

PLAU	head and neck cancer	neoplastic (FaDu, UTSCC8, UTSCC42a) and non-neoplastic (NOE)	6.06	SILAC	22918226
PLAU	head and neck cancer	neoplastic (FaDu, UTSCC8, UTSCC42a) and non-neoplastic (NOE)	6.18	SILAC	22918226
PLAU	head and neck cancer	neoplastic (FaDu, UTSCC8, UTSCC42a) and non-neoplastic (NOE)	6.58	SILAC	22918226
PLAU	head and neck cancer	neoplastic (FaDu, UTSCC8, UTSCC42a) and non-neoplastic (NOE)	6.59	SILAC	22918226
PLAU	head and neck cancer	neoplastic (FaDu, UTSCC8, UTSCC42a) and non-neoplastic (NOE)	6.83	SILAC	22918226
PLAU	head and neck cancer	neoplastic (FaDu, UTSCC8, UTSCC42a) and non-neoplastic (NOE)	6.89	SILAC	22918226
PPT1	head and neck cancer	neoplastic (FaDu, UTSCC8, UTSCC42a) and non-neoplastic (NOE)	-1.39	SILAC	22918226
PPT1	head and neck cancer	neoplastic (FaDu, UTSCC8, UTSCC42a) and non-neoplastic (NOE)	2.01	SILAC	22918226
PPT1	head and neck cancer	neoplastic (FaDu, UTSCC8, UTSCC42a) and non-neoplastic (NOE)	2.10	SILAC	22918226
PPT1	head and neck cancer	neoplastic (FaDu, UTSCC8, UTSCC42a) and non-neoplastic (NOE)	2.16	SILAC	22918226
PPT1	head and neck cancer	neoplastic (FaDu, UTSCC8, UTSCC42a) and non-neoplastic (NOE)	2.33	SILAC	22918226
PPT1	head and neck cancer	neoplastic (FaDu, UTSCC8, UTSCC42a) and non-neoplastic (NOE)	3.37	SILAC	22918226
PRDX1	head and neck cancer	neoplastic (FaDu, UTSCC8, UTSCC42a) and non-neoplastic (NOE)	1.23	SILAC	22918226
PRDX1	head and neck cancer	neoplastic (FaDu, UTSCC8, UTSCC42a) and non-neoplastic (NOE)	1.46	SILAC	22918226
PRDX1	head and neck cancer	neoplastic (FaDu, UTSCC8, UTSCC42a) and non-neoplastic (NOE)	1.81	SILAC	22918226
PRDX1	head and neck cancer	neoplastic (FaDu, UTSCC8, UTSCC42a) and non-neoplastic (NOE)	2.41	SILAC	22918226
PRDX1	head and neck cancer	neoplastic (FaDu, UTSCC8, UTSCC42a) and non-neoplastic (NOE)	2.90	SILAC	22918226
PRDX1	head and neck cancer	neoplastic (FaDu, UTSCC8, UTSCC42a) and non-neoplastic (NOE)	4.16	SILAC	22918226
PSMA1	head and neck cancer	neoplastic (FaDu, UTSCC8, UTSCC42a) and non-neoplastic (NOE)	1.32	SILAC	22918226
PSMA1	head and neck cancer	neoplastic (FaDu, UTSCC8, UTSCC42a) and non-neoplastic (NOE)	1.45	SILAC	22918226
PSMA1	head and neck cancer	neoplastic (FaDu, UTSCC8, UTSCC42a) and non-neoplastic (NOE)	1.77	SILAC	22918226
PSMA1	head and neck cancer	neoplastic (FaDu, UTSCC8, UTSCC42a) and non-neoplastic (NOE)	2.05	SILAC	22918226
PSMA1	head and neck cancer	neoplastic (FaDu, UTSCC8, UTSCC42a) and non-neoplastic (NOE)	2.12	SILAC	22918226
PSMA1	head and neck cancer	neoplastic (FaDu, UTSCC8, UTSCC42a) and non-neoplastic (NOE)	2.79	SILAC	22918226
PSMA7	head and neck cancer	neoplastic (FaDu, UTSCC8, UTSCC42a) and non-neoplastic (NOE)	1.32	SILAC	22918226
PSMA7	head and neck cancer	neoplastic (FaDu, UTSCC8, UTSCC42a) and non-neoplastic (NOE)	1.55	SILAC	22918226
PSMA7	head and neck cancer	neoplastic (FaDu, UTSCC8, UTSCC42a) and non-neoplastic (NOE)	1.70	SILAC	22918226
PSMA7	head and neck cancer	neoplastic (FaDu, UTSCC8, UTSCC42a) and non-neoplastic (NOE)	2.20	SILAC	22918226
PSMA7	head and neck cancer	neoplastic (FaDu, UTSCC8, UTSCC42a) and non-neoplastic (NOE)	2.28	SILAC	22918226
PSMA7	head and neck cancer	neoplastic (FaDu, UTSCC8, UTSCC42a) and non-neoplastic (NOE)	2.45	SILAC	22918226
PSMB6	head and neck cancer	neoplastic (FaDu, UTSCC8, UTSCC42a) and non-neoplastic (NOE)	1.04	SILAC	22918226
PSMB6	head and neck cancer	neoplastic (FaDu, UTSCC8, UTSCC42a) and non-neoplastic (NOE)	1.35	SILAC	22918226
PSMB6	head and neck cancer	neoplastic (FaDu, UTSCC8, UTSCC42a) and non-neoplastic (NOE)	2.37	SILAC	22918226
PSMB6	head and neck cancer	neoplastic (FaDu, UTSCC8, UTSCC42a) and non-neoplastic (NOE)	3.57	SILAC	22918226
PSMB6	head and neck cancer	neoplastic (FaDu, UTSCC8, UTSCC42a) and non-neoplastic (NOE)	6.40	SILAC	22918226
PSMB6	head and neck cancer	neoplastic (FaDu, UTSCC8, UTSCC42a) and non-neoplastic (NOE)	6.43	SILAC	22918226
PSMB9	head and neck cancer	neoplastic (FaDu, UTSCC8, UTSCC42a) and non-neoplastic (NOE)	5.08	SILAC	22918226
PSMB9	head and neck cancer	neoplastic (FaDu, UTSCC8, UTSCC42a) and non-neoplastic (NOE)	6.01	SILAC	22918226
PSMB9	head and neck cancer	neoplastic (FaDu, UTSCC8, UTSCC42a) and non-neoplastic (NOE)	6.05	SILAC	22918226
PSMB9	head and neck cancer	neoplastic (FaDu, UTSCC8, UTSCC42a) and non-neoplastic (NOE)	6.06	SILAC	22918226
PSMB9	head and neck cancer	neoplastic (FaDu, UTSCC8, UTSCC42a) and non-neoplastic (NOE)	6.56	SILAC	22918226
PSME1	head and neck cancer	neoplastic (FaDu, UTSCC8, UTSCC42a) and non-neoplastic (NOE)	1.03	SILAC	22918226
PSME1	head and neck cancer	neoplastic (FaDu, UTSCC8, UTSCC42a) and non-neoplastic (NOE)	2.04	SILAC	22918226

PSME1	head and neck cancer	neoplastic (FaDu, UTSCC8, UTSCC42a) and non-neoplastic (NOE)	2.06	SILAC	22918226
PSME1	head and neck cancer	neoplastic (FaDu, UTSCC8, UTSCC42a) and non-neoplastic (NOE)	2.55	SILAC	22918226
PSME1	head and neck cancer	neoplastic (FaDu, UTSCC8, UTSCC42a) and non-neoplastic (NOE)	2.62	SILAC	22918226
PSME1	head and neck cancer	neoplastic (FaDu, UTSCC8, UTSCC42a) and non-neoplastic (NOE)	2.86	SILAC	22918226
PTPRF	head and neck cancer	neoplastic (FaDu, UTSCC8, UTSCC42a) and non-neoplastic (NOE)	1.20	SILAC	22918226
PTPRF	head and neck cancer	neoplastic (FaDu, UTSCC8, UTSCC42a) and non-neoplastic (NOE)	1.49	SILAC	22918226
PTPRF	head and neck cancer	neoplastic (FaDu, UTSCC8, UTSCC42a) and non-neoplastic (NOE)	4.23	SILAC	22918226
PTPRF	head and neck cancer	neoplastic (FaDu, UTSCC8, UTSCC42a) and non-neoplastic (NOE)	6.06	SILAC	22918226
PTPRF	head and neck cancer	neoplastic (FaDu, UTSCC8, UTSCC42a) and non-neoplastic (NOE)	6.27	SILAC	22918226
PTPRK	head and neck cancer	neoplastic (FaDu, UTSCC8, UTSCC42a) and non-neoplastic (NOE)	1.53	SILAC	22918226
PTPRK	head and neck cancer	neoplastic (FaDu, UTSCC8, UTSCC42a) and non-neoplastic (NOE)	2.41	SILAC	22918226
PTPRK	head and neck cancer	neoplastic (FaDu, UTSCC8, UTSCC42a) and non-neoplastic (NOE)	3.00	SILAC	22918226
PTPRK	head and neck cancer	neoplastic (FaDu, UTSCC8, UTSCC42a) and non-neoplastic (NOE)	3.61	SILAC	22918226
PTPRK	head and neck cancer	neoplastic (FaDu, UTSCC8, UTSCC42a) and non-neoplastic (NOE)	6.16	SILAC	22918226
PTPRK	head and neck cancer	neoplastic (FaDu, UTSCC8, UTSCC42a) and non-neoplastic (NOE)	6.23	SILAC	22918226
QSOX1	head and neck cancer	neoplastic (FaDu, UTSCC8, UTSCC42a) and non-neoplastic (NOE)	1.15	SILAC	22918226
QSOX1	head and neck cancer	neoplastic (FaDu, UTSCC8, UTSCC42a) and non-neoplastic (NOE)	1.93	SILAC	22918226
QSOX1	head and neck cancer	neoplastic (FaDu, UTSCC8, UTSCC42a) and non-neoplastic (NOE)	6.87	SILAC	22918226
QSOX1	head and neck cancer	neoplastic (FaDu, UTSCC8, UTSCC42a) and non-neoplastic (NOE)	7.01	SILAC	22918226
QSOX1	head and neck cancer	neoplastic (FaDu, UTSCC8, UTSCC42a) and non-neoplastic (NOE)	7.21	SILAC	22918226
RNASET2	head and neck cancer	neoplastic (FaDu, UTSCC8, UTSCC42a) and non-neoplastic (NOE)	1.46	SILAC	22918226
RNASET2	head and neck cancer	neoplastic (FaDu, UTSCC8, UTSCC42a) and non-neoplastic (NOE)	1.62	SILAC	22918226
RNASET2	head and neck cancer	neoplastic (FaDu, UTSCC8, UTSCC42a) and non-neoplastic (NOE)	2.07	SILAC	22918226
RNASET2	head and neck cancer	neoplastic (FaDu, UTSCC8, UTSCC42a) and non-neoplastic (NOE)	6.07	SILAC	22918226
RNASET2	head and neck cancer	neoplastic (FaDu, UTSCC8, UTSCC42a) and non-neoplastic (NOE)	6.24	SILAC	22918226
RPS21	head and neck cancer	neoplastic (FaDu, UTSCC8, UTSCC42a) and non-neoplastic (NOE)	1.20	SILAC	22918226
RPS21	head and neck cancer	neoplastic (FaDu, UTSCC8, UTSCC42a) and non-neoplastic (NOE)	1.43	SILAC	22918226
RPS21	head and neck cancer	neoplastic (FaDu, UTSCC8, UTSCC42a) and non-neoplastic (NOE)	1.53	SILAC	22918226
RPS21	head and neck cancer	neoplastic (FaDu, UTSCC8, UTSCC42a) and non-neoplastic (NOE)	2.18	SILAC	22918226
RPS21	head and neck cancer	neoplastic (FaDu, UTSCC8, UTSCC42a) and non-neoplastic (NOE)	6.37	SILAC	22918226
RPS21	head and neck cancer	neoplastic (FaDu, UTSCC8, UTSCC42a) and non-neoplastic (NOE)	6.55	SILAC	22918226
SEPHS1	head and neck cancer	neoplastic (FaDu, UTSCC8, UTSCC42a) and non-neoplastic (NOE)	1.38	SILAC	22918226
SEPHS1	head and neck cancer	neoplastic (FaDu, UTSCC8, UTSCC42a) and non-neoplastic (NOE)	1.85	SILAC	22918226
SEPHS1	head and neck cancer	neoplastic (FaDu, UTSCC8, UTSCC42a) and non-neoplastic (NOE)	1.98	SILAC	22918226
SEPHS1	head and neck cancer	neoplastic (FaDu, UTSCC8, UTSCC42a) and non-neoplastic (NOE)	2.73	SILAC	22918226
SEPHS1	head and neck cancer	neoplastic (FaDu, UTSCC8, UTSCC42a) and non-neoplastic (NOE)	6.05	SILAC	22918226
SEPHS1	head and neck cancer	neoplastic (FaDu, UTSCC8, UTSCC42a) and non-neoplastic (NOE)	6.41	SILAC	22918226
SEPT7	head and neck cancer	neoplastic (FaDu, UTSCC8, UTSCC42a) and non-neoplastic (NOE)	1.30	SILAC	22918226
SEPT7	head and neck cancer	neoplastic (FaDu, UTSCC8, UTSCC42a) and non-neoplastic (NOE)	1.67	SILAC	22918226
SEPT7	head and neck cancer	neoplastic (FaDu, UTSCC8, UTSCC42a) and non-neoplastic (NOE)	1.71	SILAC	22918226
SEPT7	head and neck cancer	neoplastic (FaDu, UTSCC8, UTSCC42a) and non-neoplastic (NOE)	2.96	SILAC	22918226
SEPT7	head and neck cancer	neoplastic (FaDu, UTSCC8, UTSCC42a) and non-neoplastic (NOE)	5.92	SILAC	22918226
SEPT7	head and neck cancer	neoplastic (FaDu, UTSCC8, UTSCC42a) and non-neoplastic (NOE)	5.97	SILAC	22918226

SERPINB1	head and neck cancer	neoplastic (FaDu, UTSCC8, UTSCC42a) and non-neoplastic (NOE)	1.15	SILAC	22918226
SERPINB1	head and neck cancer	neoplastic (FaDu, UTSCC8, UTSCC42a) and non-neoplastic (NOE)	1.70	SILAC	22918226
SERPINB1	head and neck cancer	neoplastic (FaDu, UTSCC8, UTSCC42a) and non-neoplastic (NOE)	3.21	SILAC	22918226
SERPINB1	head and neck cancer	neoplastic (FaDu, UTSCC8, UTSCC42a) and non-neoplastic (NOE)	6.06	SILAC	22918226
SERPINB1	head and neck cancer	neoplastic (FaDu, UTSCC8, UTSCC42a) and non-neoplastic (NOE)	6.06	SILAC	22918226
SERPINB1	head and neck cancer	neoplastic (FaDu, UTSCC8, UTSCC42a) and non-neoplastic (NOE)	6.14	SILAC	22918226
SH3BGRL3	head and neck cancer	neoplastic (FaDu, UTSCC8, UTSCC42a) and non-neoplastic (NOE)	1.05	SILAC	22918226
SH3BGRL3	head and neck cancer	neoplastic (FaDu, UTSCC8, UTSCC42a) and non-neoplastic (NOE)	1.53	SILAC	22918226
SH3BGRL3	head and neck cancer	neoplastic (FaDu, UTSCC8, UTSCC42a) and non-neoplastic (NOE)	1.61	SILAC	22918226
SH3BGRL3	head and neck cancer	neoplastic (FaDu, UTSCC8, UTSCC42a) and non-neoplastic (NOE)	1.93	SILAC	22918226
SH3BGRL3	head and neck cancer	neoplastic (FaDu, UTSCC8, UTSCC42a) and non-neoplastic (NOE)	6.24	SILAC	22918226
SH3BGRL3	head and neck cancer	neoplastic (FaDu, UTSCC8, UTSCC42a) and non-neoplastic (NOE)	6.45	SILAC	22918226
TCEA1	head and neck cancer	neoplastic (FaDu, UTSCC8, UTSCC42a) and non-neoplastic (NOE)	1.09	SILAC	22918226
TCEA1	head and neck cancer	neoplastic (FaDu, UTSCC8, UTSCC42a) and non-neoplastic (NOE)	2.35	SILAC	22918226
TCEA1	head and neck cancer	neoplastic (FaDu, UTSCC8, UTSCC42a) and non-neoplastic (NOE)	3.26	SILAC	22918226
TCEA1	head and neck cancer	neoplastic (FaDu, UTSCC8, UTSCC42a) and non-neoplastic (NOE)	5.89	SILAC	22918226
TCEA1	head and neck cancer	neoplastic (FaDu, UTSCC8, UTSCC42a) and non-neoplastic (NOE)	6.24	SILAC	22918226
TGFBI	head and neck cancer	neoplastic (FaDu, UTSCC8, UTSCC42a) and non-neoplastic (NOE)	6.23	SILAC	22918226
TGFBI	head and neck cancer	neoplastic (FaDu, UTSCC8, UTSCC42a) and non-neoplastic (NOE)	6.32	SILAC	22918226
TGFBI	head and neck cancer	neoplastic (FaDu, UTSCC8, UTSCC42a) and non-neoplastic (NOE)	6.95	SILAC	22918226
TGFBI	head and neck cancer	neoplastic (FaDu, UTSCC8, UTSCC42a) and non-neoplastic (NOE)	7.15	SILAC	22918226
TGFBI	head and neck cancer	neoplastic (FaDu, UTSCC8, UTSCC42a) and non-neoplastic (NOE)	7.57	SILAC	22918226
TGFBI	head and neck cancer	neoplastic (FaDu, UTSCC8, UTSCC42a) and non-neoplastic (NOE)	8.10	SILAC	22918226
THBS1	head and neck cancer	neoplastic (FaDu, UTSCC8, UTSCC42a) and non-neoplastic (NOE)	4.56	SILAC	22918226
THBS1	head and neck cancer	neoplastic (FaDu, UTSCC8, UTSCC42a) and non-neoplastic (NOE)	5.30	SILAC	22918226
THBS1	head and neck cancer	neoplastic (FaDu, UTSCC8, UTSCC42a) and non-neoplastic (NOE)	5.92	SILAC	22918226
THBS1	head and neck cancer	neoplastic (FaDu, UTSCC8, UTSCC42a) and non-neoplastic (NOE)	6.50	SILAC	22918226
THBS1	head and neck cancer	neoplastic (FaDu, UTSCC8, UTSCC42a) and non-neoplastic (NOE)	7.07	SILAC	22918226
THBS1	head and neck cancer	neoplastic (FaDu, UTSCC8, UTSCC42a) and non-neoplastic (NOE)	7.88	SILAC	22918226
TINAGL1	head and neck cancer	neoplastic (FaDu, UTSCC8, UTSCC42a) and non-neoplastic (NOE)	3.20	SILAC	22918226
TINAGL1	head and neck cancer	neoplastic (FaDu, UTSCC8, UTSCC42a) and non-neoplastic (NOE)	4.07	SILAC	22918226
TINAGL1	head and neck cancer	neoplastic (FaDu, UTSCC8, UTSCC42a) and non-neoplastic (NOE)	4.22	SILAC	22918226
TINAGL1	head and neck cancer	neoplastic (FaDu, UTSCC8, UTSCC42a) and non-neoplastic (NOE)	6.14	SILAC	22918226
TINAGL1	head and neck cancer	neoplastic (FaDu, UTSCC8, UTSCC42a) and non-neoplastic (NOE)	6.84	SILAC	22918226
TINAGL1	head and neck cancer	neoplastic (FaDu, UTSCC8, UTSCC42a) and non-neoplastic (NOE)	7.07	SILAC	22918226
TPD52	head and neck cancer	neoplastic (FaDu, UTSCC8, UTSCC42a) and non-neoplastic (NOE)	2	SILAC	22918226
TPD52	head and neck cancer	neoplastic (FaDu, UTSCC8, UTSCC42a) and non-neoplastic (NOE)	1.69	SILAC	22918226
TPD52	head and neck cancer	neoplastic (FaDu, UTSCC8, UTSCC42a) and non-neoplastic (NOE)	2.31	SILAC	22918226
TPD52	head and neck cancer	neoplastic (FaDu, UTSCC8, UTSCC42a) and non-neoplastic (NOE)	6.25	SILAC	22918226
TPD52	head and neck cancer	neoplastic (FaDu, UTSCC8, UTSCC42a) and non-neoplastic (NOE)	6.54	SILAC	22918226
TPD52	head and neck cancer	neoplastic (FaDu, UTSCC8, UTSCC42a) and non-neoplastic (NOE)	6.66	SILAC	22918226
TPM3	head and neck cancer	neoplastic (FaDu, UTSCC8, UTSCC42a) and non-neoplastic (NOE)	1.91	SILAC	22918226
TPM3	head and neck cancer	neoplastic (FaDu, UTSCC8, UTSCC42a) and non-neoplastic (NOE)	2.32	SILAC	22918226

TPM3	head and neck cancer	neoplastic (FaDu, UTSCC8, UTSCC42a) and non-neoplastic (NOE)	2.57	SILAC	22918226
TPM3	head and neck cancer	neoplastic (FaDu, UTSCC8, UTSCC42a) and non-neoplastic (NOE)	2.69	SILAC	22918226
TPM3	head and neck cancer	neoplastic (FaDu, UTSCC8, UTSCC42a) and non-neoplastic (NOE)	2.71	SILAC	22918226
TPM3	head and neck cancer	neoplastic (FaDu, UTSCC8, UTSCC42a) and non-neoplastic (NOE)	2.93	SILAC	22918226
TXNDC17	head and neck cancer	neoplastic (FaDu, UTSCC8, UTSCC42a) and non-neoplastic (NOE)	1.45	SILAC	22918226
TXNDC17	head and neck cancer	neoplastic (FaDu, UTSCC8, UTSCC42a) and non-neoplastic (NOE)	2.12	SILAC	22918226
TXNDC17	head and neck cancer	neoplastic (FaDu, UTSCC8, UTSCC42a) and non-neoplastic (NOE)	2.26	SILAC	22918226
TXNDC17	head and neck cancer	neoplastic (FaDu, UTSCC8, UTSCC42a) and non-neoplastic (NOE)	6.18	SILAC	22918226
TXNDC17	head and neck cancer	neoplastic (FaDu, UTSCC8, UTSCC42a) and non-neoplastic (NOE)	6.18	SILAC	22918226
TXNDC17	head and neck cancer	neoplastic (FaDu, UTSCC8, UTSCC42a) and non-neoplastic (NOE)	6.30	SILAC	22918226
UBE2V2	head and neck cancer	neoplastic (FaDu, UTSCC8, UTSCC42a) and non-neoplastic (NOE)	-1.05	SILAC	22918226
UBQLN1	head and neck cancer	neoplastic (FaDu, UTSCC8, UTSCC42a) and non-neoplastic (NOE)	-1.08	SILAC	22918226
UBR7	head and neck cancer	neoplastic (FaDu, UTSCC8, UTSCC42a) and non-neoplastic (NOE)	1.15	SILAC	22918226
UCHL1	head and neck cancer	neoplastic (FaDu, UTSCC8, UTSCC42a) and non-neoplastic (NOE)	1.60	SILAC	22918226
UCHL3	head and neck cancer	neoplastic (FaDu, UTSCC8, UTSCC42a) and non-neoplastic (NOE)	1.49	SILAC	22918226
UCHL3	head and neck cancer	neoplastic (FaDu, UTSCC8, UTSCC42a) and non-neoplastic (NOE)	2.24	SILAC	22918226
UCHL3	head and neck cancer	neoplastic (FaDu, UTSCC8, UTSCC42a) and non-neoplastic (NOE)	6.15	SILAC	22918226
UCHL3	head and neck cancer	neoplastic (FaDu, UTSCC8, UTSCC42a) and non-neoplastic (NOE)	6.53	SILAC	22918226
UCHL3	head and neck cancer	neoplastic (FaDu, UTSCC8, UTSCC42a) and non-neoplastic (NOE)	6.69	SILAC	22918226
UFM1	head and neck cancer	neoplastic (FaDu, UTSCC8, UTSCC42a) and non-neoplastic (NOE)	-1.25	SILAC	22918226
UGDH	head and neck cancer	neoplastic (FaDu, UTSCC8, UTSCC42a) and non-neoplastic (NOE)	1.58	SILAC	22918226
UGP2	head and neck cancer	neoplastic (FaDu, UTSCC8, UTSCC42a) and non-neoplastic (NOE)	-1.18	SILAC	22918226
URM1	head and neck cancer	neoplastic (FaDu, UTSCC8, UTSCC42a) and non-neoplastic (NOE)	-1.68	SILAC	22918226
USP7	head and neck cancer	neoplastic (FaDu, UTSCC8, UTSCC42a) and non-neoplastic (NOE)	1.89	SILAC	22918226
USP9X	head and neck cancer	neoplastic (FaDu, UTSCC8, UTSCC42a) and non-neoplastic (NOE)	1.01	SILAC	22918226
VAPA	head and neck cancer	neoplastic (FaDu, UTSCC8, UTSCC42a) and non-neoplastic (NOE)	-1.15	SILAC	22918226
VAPB	head and neck cancer	neoplastic (FaDu, UTSCC8, UTSCC42a) and non-neoplastic (NOE)	1.01	SILAC	22918226
VASN	head and neck cancer	neoplastic (FaDu, UTSCC8, UTSCC42a) and non-neoplastic (NOE)	-1.15	SILAC	22918226
VAT1	head and neck cancer	neoplastic (FaDu, UTSCC8, UTSCC42a) and non-neoplastic (NOE)	-1.12	SILAC	22918226
VBP1	head and neck cancer	neoplastic (FaDu, UTSCC8, UTSCC42a) and non-neoplastic (NOE)	1.04	SILAC	22918226
VCAN	head and neck cancer	neoplastic (FaDu, UTSCC8, UTSCC42a) and non-neoplastic (NOE)	-1.47	SILAC	22918226
VCL	head and neck cancer	neoplastic (FaDu, UTSCC8, UTSCC42a) and non-neoplastic (NOE)	1.17	SILAC	22918226
VCL	head and neck cancer	neoplastic (FaDu, UTSCC8, UTSCC42a) and non-neoplastic (NOE)	1.58	SILAC	22918226
VCL	head and neck cancer	neoplastic (FaDu, UTSCC8, UTSCC42a) and non-neoplastic (NOE)	2.03	SILAC	22918226
VCL	head and neck cancer	neoplastic (FaDu, UTSCC8, UTSCC42a) and non-neoplastic (NOE)	2.62	SILAC	22918226
VCL	head and neck cancer	neoplastic (FaDu, UTSCC8, UTSCC42a) and non-neoplastic (NOE)	2.98	SILAC	22918226
VCL	head and neck cancer	neoplastic (FaDu, UTSCC8, UTSCC42a) and non-neoplastic (NOE)	3.42	SILAC	22918226
VCP	head and neck cancer	neoplastic (FaDu, UTSCC8, UTSCC42a) and non-neoplastic (NOE)	1.83	SILAC	22918226
VCP	head and neck cancer	neoplastic (FaDu, UTSCC8, UTSCC42a) and non-neoplastic (NOE)	2.74	SILAC	22918226
VIM	head and neck cancer	neoplastic (FaDu, UTSCC8, UTSCC42a) and non-neoplastic (NOE)	-2.32	SILAC	22918226
VPS26A	head and neck cancer	neoplastic (FaDu, UTSCC8, UTSCC42a) and non-neoplastic (NOE)	1.33	SILAC	22918226
VPS35	head and neck cancer	neoplastic (FaDu, UTSCC8, UTSCC42a) and non-neoplastic (NOE)	2.04	SILAC	22918226
VSNL1	head and neck cancer	neoplastic (FaDu, UTSCC8, UTSCC42a) and non-neoplastic (NOE)	-1.94	SILAC	22918226

VTN	head and neck cancer	neoplastic (FaDu, UTSCC8, UTSCC42a) and non-neoplastic (NOE)	2.81	SILAC	22918226
WAPAL	head and neck cancer	neoplastic (FaDu, UTSCC8, UTSCC42a) and non-neoplastic (NOE)	-3.32	SILAC	22918226
WBP2	head and neck cancer	neoplastic (FaDu, UTSCC8, UTSCC42a) and non-neoplastic (NOE)	-1.05	SILAC	22918226
WDHD1	head and neck cancer	neoplastic (FaDu, UTSCC8, UTSCC42a) and non-neoplastic (NOE)	7.18	SILAC	22918226
WDR5	head and neck cancer	neoplastic (FaDu, UTSCC8, UTSCC42a) and non-neoplastic (NOE)	1.15	SILAC	22918226
WFS1	head and neck cancer	neoplastic (FaDu, UTSCC8, UTSCC42a) and non-neoplastic (NOE)	-1.18	SILAC	22918226
WIBG	head and neck cancer	neoplastic (FaDu, UTSCC8, UTSCC42a) and non-neoplastic (NOE)	-1.47	SILAC	22918226
XDH	head and neck cancer	neoplastic (FaDu, UTSCC8, UTSCC42a) and non-neoplastic (NOE)	-2.73	SILAC	22918226
XKR3	head and neck cancer	neoplastic (FaDu, UTSCC8, UTSCC42a) and non-neoplastic (NOE)	1.18	SILAC	22918226
XPNPEP1	head and neck cancer	neoplastic (FaDu, UTSCC8, UTSCC42a) and non-neoplastic (NOE)	-1.12	SILAC	22918226
XPNPEP3	head and neck cancer	neoplastic (FaDu, UTSCC8, UTSCC42a) and non-neoplastic (NOE)	2.43	SILAC	22918226
XRCC5	head and neck cancer	neoplastic (FaDu, UTSCC8, UTSCC42a) and non-neoplastic (NOE)	-1.73	SILAC	22918226
XRCC5	head and neck cancer	neoplastic (FaDu, UTSCC8, UTSCC42a) and non-neoplastic (NOE)	2.68	SILAC	22918226
XRCC6	head and neck cancer	neoplastic (FaDu, UTSCC8, UTSCC42a) and non-neoplastic (NOE)	2.18	SILAC	22918226
YBX1	head and neck cancer	neoplastic (FaDu, UTSCC8, UTSCC42a) and non-neoplastic (NOE)	1.19	SILAC	22918226
YBX1	head and neck cancer	neoplastic (FaDu, UTSCC8, UTSCC42a) and non-neoplastic (NOE)	-2.32	SILAC	22918226
YIPF3	head and neck cancer	neoplastic (FaDu, UTSCC8, UTSCC42a) and non-neoplastic (NOE)	1.11	SILAC	22918226
YWHAB	head and neck cancer	neoplastic (FaDu, UTSCC8, UTSCC42a) and non-neoplastic (NOE)	-1	SILAC	22918226
YWHAG	head and neck cancer	neoplastic (FaDu, UTSCC8, UTSCC42a) and non-neoplastic (NOE)	1.20	SILAC	22918226
YWHAZ	head and neck cancer	neoplastic (FaDu, UTSCC8, UTSCC42a) and non-neoplastic (NOE)	1.80	SILAC	22918226
ZC3H15	head and neck cancer	neoplastic (FaDu, UTSCC8, UTSCC42a) and non-neoplastic (NOE)	3.31	SILAC	22918226
ZC3HAV1	head and neck cancer	neoplastic (FaDu, UTSCC8, UTSCC42a) and non-neoplastic (NOE)	2.12	SILAC	22918226
ZDBF2	head and neck cancer	neoplastic (FaDu, UTSCC8, UTSCC42a) and non-neoplastic (NOE)	2.84	SILAC	22918226
ZFR	head and neck cancer	neoplastic (FaDu, UTSCC8, UTSCC42a) and non-neoplastic (NOE)	1.33	SILAC	22918226
ZNF185	head and neck cancer	neoplastic (FaDu, UTSCC8, UTSCC42a) and non-neoplastic (NOE)	-1.39	SILAC	22918226
ZNF207	head and neck cancer	neoplastic (FaDu, UTSCC8, UTSCC42a) and non-neoplastic (NOE)	1.54	SILAC	22918226
ZNF259	head and neck cancer	neoplastic (FaDu, UTSCC8, UTSCC42a) and non-neoplastic (NOE)	-1.02	SILAC	22918226
ZNF518B	head and neck cancer	neoplastic (FaDu, UTSCC8, UTSCC42a) and non-neoplastic (NOE)	2.16	SILAC	22918226
ZNF787	head and neck cancer	neoplastic (FaDu, UTSCC8, UTSCC42a) and non-neoplastic (NOE)	1.25	SILAC	22918226
ZYX	head and neck cancer	neoplastic (FaDu, UTSCC8, UTSCC42a) and non-neoplastic (NOE)	-1.21	SILAC	22918226
AARS	gastric cancer	neoplastic (AGS, KatoIII, NCI-N87, SNU-1, SNU-5, SNU-16) and non-neoplastic (HFE-145)	1.68	SILAC	23161554
ABCE1	gastric cancer	neoplastic (AGS, KatoIII, NCI-N87, SNU-1, SNU-5, SNU-16) and non-neoplastic (HFE-145)	2.78	SILAC	23161554
ABHD14B	gastric cancer	neoplastic (AGS, KatoIII, NCI-N87, SNU-1, SNU-5, SNU-16) and non-neoplastic (HFE-145)	2.09	SILAC	23161554
ACAT1	gastric cancer	neoplastic (AGS, KatoIII, NCI-N87, SNU-1, SNU-5, SNU-16) and non-neoplastic (HFE-145)	2.13	SILAC	23161554
ACIN1	gastric cancer	neoplastic (AGS, KatoIII, NCI-N87, SNU-1, SNU-5, SNU-16) and non-neoplastic (HFE-145)	1.63	SILAC	23161554
ACTA1	gastric cancer	neoplastic (AGS, KatoIII, NCI-N87, SNU-1, SNU-5, SNU-16) and non-neoplastic (HFE-145)	1.19	SILAC	23161554
ACTB	gastric cancer	neoplastic (AGS, KatoIII, NCI-N87, SNU-1, SNU-5, SNU-16) and non-neoplastic (HFE-145)	1.58	SILAC	23161554
ACTL6A	gastric cancer	neoplastic (AGS, KatoIII, NCI-N87, SNU-1, SNU-5, SNU-16) and non-neoplastic (HFE-145)	-2.55	SILAC	23161554
ACTN3	gastric cancer	neoplastic (AGS, KatoIII, NCI-N87, SNU-1, SNU-5, SNU-16) and non-neoplastic (HFE-145)	1.52	SILAC	23161554
ACTN4	gastric cancer	neoplastic (AGS, KatoIII, NCI-N87, SNU-1, SNU-5, SNU-16) and non-neoplastic (HFE-145)	2.07	SILAC	23161554
ADAM10	gastric cancer	neoplastic (AGS, KatoIII, NCI-N87, SNU-1, SNU-5, SNU-16) and non-neoplastic (HFE-145)	4.36	SILAC	23161554
ADH5	gastric cancer	neoplastic (AGS, KatoIII, NCI-N87, SNU-1, SNU-5, SNU-16) and non-neoplastic (HFE-145)	1.53	SILAC	23161554
AGR2	gastric cancer	neoplastic (AGS, KatoIII, NCI-N87, SNU-1, SNU-5, SNU-16) and non-neoplastic (HFE-145)	5.74	SILAC	23161554

AGRN	gastric cancer	neoplastic (AGS, KatoIII, NCI-N87, SNU-1, SNU-5, SNU-16) and non-neoplastic (HFE-145)	1.94	SILAC	23161554
AHCY	gastric cancer	neoplastic (AGS, KatoIII, NCI-N87, SNU-1, SNU-5, SNU-16) and non-neoplastic (HFE-145)	1.95	SILAC	23161554
AHNAK	gastric cancer	neoplastic (AGS, KatoIII, NCI-N87, SNU-1, SNU-5, SNU-16) and non-neoplastic (HFE-145)	2.92	SILAC	23161554
AIMP1	gastric cancer	neoplastic (AGS, KatoIII, NCI-N87, SNU-1, SNU-5, SNU-16) and non-neoplastic (HFE-145)	1.04	SILAC	23161554
AIMP2	gastric cancer	neoplastic (AGS, KatoIII, NCI-N87, SNU-1, SNU-5, SNU-16) and non-neoplastic (HFE-145)	2.19	SILAC	23161554
AK2	gastric cancer	neoplastic (AGS, KatoIII, NCI-N87, SNU-1, SNU-5, SNU-16) and non-neoplastic (HFE-145)	-1.21	SILAC	23161554
AK3	gastric cancer	neoplastic (AGS, KatoIII, NCI-N87, SNU-1, SNU-5, SNU-16) and non-neoplastic (HFE-145)	-1.78	SILAC	23161554
AKAP12	gastric cancer	neoplastic (AGS, KatoIII, NCI-N87, SNU-1, SNU-5, SNU-16) and non-neoplastic (HFE-145)	-2.83	SILAC	23161554
AKR1A1	gastric cancer	neoplastic (AGS, KatoIII, NCI-N87, SNU-1, SNU-5, SNU-16) and non-neoplastic (HFE-145)	1.18	SILAC	23161554
AKR1C2	gastric cancer	neoplastic (AGS, KatoIII, NCI-N87, SNU-1, SNU-5, SNU-16) and non-neoplastic (HFE-145)	4.46	SILAC	23161554
AKT2	gastric cancer	neoplastic (AGS, KatoIII, NCI-N87, SNU-1, SNU-5, SNU-16) and non-neoplastic (HFE-145)	1.93	SILAC	23161554
ALCAM	gastric cancer	neoplastic (AGS, KatoIII, NCI-N87, SNU-1, SNU-5, SNU-16) and non-neoplastic (HFE-145)	1	SILAC	23161554
ALDH16A1	gastric cancer	neoplastic (AGS, KatoIII, NCI-N87, SNU-1, SNU-5, SNU-16) and non-neoplastic (HFE-145)	1.08	SILAC	23161554
ALDH1A1	gastric cancer	neoplastic (AGS, KatoIII, NCI-N87, SNU-1, SNU-5, SNU-16) and non-neoplastic (HFE-145)	5.51	SILAC	23161554
ALDH1A3	gastric cancer	neoplastic (AGS, KatoIII, NCI-N87, SNU-1, SNU-5, SNU-16) and non-neoplastic (HFE-145)	3.74	SILAC	23161554
ALDH9A1	gastric cancer	neoplastic (AGS, KatoIII, NCI-N87, SNU-1, SNU-5, SNU-16) and non-neoplastic (HFE-145)	1.13	SILAC	23161554
ALDOC	gastric cancer	neoplastic (AGS, KatoIII, NCI-N87, SNU-1, SNU-5, SNU-16) and non-neoplastic (HFE-145)	1.00	SILAC	23161554
ANG	gastric cancer	neoplastic (AGS, KatoIII, NCI-N87, SNU-1, SNU-5, SNU-16) and non-neoplastic (HFE-145)	1.16	SILAC	23161554
ANP32A	gastric cancer	neoplastic (AGS, KatoIII, NCI-N87, SNU-1, SNU-5, SNU-16) and non-neoplastic (HFE-145)	1.85	SILAC	23161554
ANP32B	gastric cancer	neoplastic (AGS, KatoIII, NCI-N87, SNU-1, SNU-5, SNU-16) and non-neoplastic (HFE-145)	2.70	SILAC	23161554
ANP32E	gastric cancer	neoplastic (AGS, KatoIII, NCI-N87, SNU-1, SNU-5, SNU-16) and non-neoplastic (HFE-145)	2.16	SILAC	23161554
ANXA1	gastric cancer	neoplastic (AGS, KatoIII, NCI-N87, SNU-1, SNU-5, SNU-16) and non-neoplastic (HFE-145)	5.45	SILAC	23161554
ANXA10	gastric cancer	neoplastic (AGS, KatoIII, NCI-N87, SNU-1, SNU-5, SNU-16) and non-neoplastic (HFE-145)	4.76	SILAC	23161554
ANXA2	gastric cancer	neoplastic (AGS, KatoIII, NCI-N87, SNU-1, SNU-5, SNU-16) and non-neoplastic (HFE-145)	2.59	SILAC	23161554
ANXA3	gastric cancer	neoplastic (AGS, KatoIII, NCI-N87, SNU-1, SNU-5, SNU-16) and non-neoplastic (HFE-145)	2.54	SILAC	23161554
ANXA4	gastric cancer	neoplastic (AGS, KatoIII, NCI-N87, SNU-1, SNU-5, SNU-16) and non-neoplastic (HFE-145)	2.67	SILAC	23161554
ANXA5	gastric cancer	neoplastic (AGS, KatoIII, NCI-N87, SNU-1, SNU-5, SNU-16) and non-neoplastic (HFE-145)	1.36	SILAC	23161554
AP1B1	gastric cancer	neoplastic (AGS, KatoIII, NCI-N87, SNU-1, SNU-5, SNU-16) and non-neoplastic (HFE-145)	1.15	SILAC	23161554
AP1S1	gastric cancer	neoplastic (AGS, KatoIII, NCI-N87, SNU-1, SNU-5, SNU-16) and non-neoplastic (HFE-145)	1.10	SILAC	23161554
AP2A1	gastric cancer	neoplastic (AGS, KatoIII, NCI-N87, SNU-1, SNU-5, SNU-16) and non-neoplastic (HFE-145)	-1.55	SILAC	23161554
AP2A2	gastric cancer	neoplastic (AGS, KatoIII, NCI-N87, SNU-1, SNU-5, SNU-16) and non-neoplastic (HFE-145)	2.30	SILAC	23161554
AP2B1	gastric cancer	neoplastic (AGS, KatoIII, NCI-N87, SNU-1, SNU-5, SNU-16) and non-neoplastic (HFE-145)	1.37	SILAC	23161554
APEH	gastric cancer	neoplastic (AGS, KatoIII, NCI-N87, SNU-1, SNU-5, SNU-16) and non-neoplastic (HFE-145)	1.45	SILAC	23161554
APEX1	gastric cancer	neoplastic (AGS, KatoIII, NCI-N87, SNU-1, SNU-5, SNU-16) and non-neoplastic (HFE-145)	1.13	SILAC	23161554
APIP	gastric cancer	neoplastic (AGS, KatoIII, NCI-N87, SNU-1, SNU-5, SNU-16) and non-neoplastic (HFE-145)	2.61	SILAC	23161554
APOA1BP	gastric cancer	neoplastic (AGS, KatoIII, NCI-N87, SNU-1, SNU-5, SNU-16) and non-neoplastic (HFE-145)	1.24	SILAC	23161554
APOB	gastric cancer	neoplastic (AGS, KatoIII, NCI-N87, SNU-1, SNU-5, SNU-16) and non-neoplastic (HFE-145)	1.94	SILAC	23161554
APOBEC3C	gastric cancer	neoplastic (AGS, KatoIII, NCI-N87, SNU-1, SNU-5, SNU-16) and non-neoplastic (HFE-145)	1.43	SILAC	23161554
APP	gastric cancer	neoplastic (AGS, KatoIII, NCI-N87, SNU-1, SNU-5, SNU-16) and non-neoplastic (HFE-145)	2.28	SILAC	23161554
APRT	gastric cancer	neoplastic (AGS, KatoIII, NCI-N87, SNU-1, SNU-5, SNU-16) and non-neoplastic (HFE-145)	3.45	SILAC	23161554
AREG	gastric cancer	neoplastic (AGS, KatoIII, NCI-N87, SNU-1, SNU-5, SNU-16) and non-neoplastic (HFE-145)	2.25	SILAC	23161554
ARF1	gastric cancer	neoplastic (AGS, KatoIII, NCI-N87, SNU-1, SNU-5, SNU-16) and non-neoplastic (HFE-145)	1.11	SILAC	23161554
ARF4	gastric cancer	neoplastic (AGS, KatoIII, NCI-N87, SNU-1, SNU-5, SNU-16) and non-neoplastic (HFE-145)	1.46	SILAC	23161554

ARHGAP1	gastric cancer	neoplastic (AGS, KatoIII, NCI-N87, SNU-1, SNU-5, SNU-16) and non-neoplastic (HFE-145)	1.65	SILAC	23161554
ARHGAP18	gastric cancer	neoplastic (AGS, KatoIII, NCI-N87, SNU-1, SNU-5, SNU-16) and non-neoplastic (HFE-145)	1.93	SILAC	23161554
ARPC1B	gastric cancer	neoplastic (AGS, KatoIII, NCI-N87, SNU-1, SNU-5, SNU-16) and non-neoplastic (HFE-145)	1	SILAC	23161554
ASL	gastric cancer	neoplastic (AGS, KatoIII, NCI-N87, SNU-1, SNU-5, SNU-16) and non-neoplastic (HFE-145)	1.76	SILAC	23161554
ASNS	gastric cancer	neoplastic (AGS, KatoIII, NCI-N87, SNU-1, SNU-5, SNU-16) and non-neoplastic (HFE-145)	1.97	SILAC	23161554
ATIC	gastric cancer	neoplastic (AGS, KatoIII, NCI-N87, SNU-1, SNU-5, SNU-16) and non-neoplastic (HFE-145)	1.71	SILAC	23161554
ATP5B	gastric cancer	neoplastic (AGS, KatoIII, NCI-N87, SNU-1, SNU-5, SNU-16) and non-neoplastic (HFE-145)	1.49	SILAC	23161554
ATP6AP2	gastric cancer	neoplastic (AGS, KatoIII, NCI-N87, SNU-1, SNU-5, SNU-16) and non-neoplastic (HFE-145)	2.70	SILAC	23161554
ATP6V1E1	gastric cancer	neoplastic (AGS, KatoIII, NCI-N87, SNU-1, SNU-5, SNU-16) and non-neoplastic (HFE-145)	1.11	SILAC	23161554
ATXN1	gastric cancer	neoplastic (AGS, KatoIII, NCI-N87, SNU-1, SNU-5, SNU-16) and non-neoplastic (HFE-145)	2.39	SILAC	23161554
AXL	gastric cancer	neoplastic (AGS, KatoIII, NCI-N87, SNU-1, SNU-5, SNU-16) and non-neoplastic (HFE-145)	-3.64	SILAC	23161554
B2M	gastric cancer	neoplastic (AGS, KatoIII, NCI-N87, SNU-1, SNU-5, SNU-16) and non-neoplastic (HFE-145)	2.11	SILAC	23161554
B3GNT1	gastric cancer	neoplastic (AGS, KatoIII, NCI-N87, SNU-1, SNU-5, SNU-16) and non-neoplastic (HFE-145)	-2.12	SILAC	23161554
BAG2	gastric cancer	neoplastic (AGS, KatoIII, NCI-N87, SNU-1, SNU-5, SNU-16) and non-neoplastic (HFE-145)	1.52	SILAC	23161554
BAG5	gastric cancer	neoplastic (AGS, KatoIII, NCI-N87, SNU-1, SNU-5, SNU-16) and non-neoplastic (HFE-145)	2.62	SILAC	23161554
BAG6	gastric cancer	neoplastic (AGS, KatoIII, NCI-N87, SNU-1, SNU-5, SNU-16) and non-neoplastic (HFE-145)	1.44	SILAC	23161554
BANF1	gastric cancer	neoplastic (AGS, KatoIII, NCI-N87, SNU-1, SNU-5, SNU-16) and non-neoplastic (HFE-145)	1.55	SILAC	23161554
BCAM	gastric cancer	neoplastic (AGS, KatoIII, NCI-N87, SNU-1, SNU-5, SNU-16) and non-neoplastic (HFE-145)	3.94	SILAC	23161554
BLMH	gastric cancer	neoplastic (AGS, KatoIII, NCI-N87, SNU-1, SNU-5, SNU-16) and non-neoplastic (HFE-145)	2.08	SILAC	23161554
BLVRB	gastric cancer	neoplastic (AGS, KatoIII, NCI-N87, SNU-1, SNU-5, SNU-16) and non-neoplastic (HFE-145)	1.56	SILAC	23161554
BPNT1	gastric cancer	neoplastic (AGS, KatoIII, NCI-N87, SNU-1, SNU-5, SNU-16) and non-neoplastic (HFE-145)	1.45	SILAC	23161554
BSG	gastric cancer	neoplastic (AGS, KatoIII, NCI-N87, SNU-1, SNU-5, SNU-16) and non-neoplastic (HFE-145)	1.22	SILAC	23161554
BTF3	gastric cancer	neoplastic (AGS, KatoIII, NCI-N87, SNU-1, SNU-5, SNU-16) and non-neoplastic (HFE-145)	3.70	SILAC	23161554
BUB3	gastric cancer	neoplastic (AGS, KatoIII, NCI-N87, SNU-1, SNU-5, SNU-16) and non-neoplastic (HFE-145)	1.02	SILAC	23161554
BZW2	gastric cancer	neoplastic (AGS, KatoIII, NCI-N87, SNU-1, SNU-5, SNU-16) and non-neoplastic (HFE-145)	2.40	SILAC	23161554
C11orf2	gastric cancer	neoplastic (AGS, KatoIII, NCI-N87, SNU-1, SNU-5, SNU-16) and non-neoplastic (HFE-145)	1.70	SILAC	23161554
C11orf68	gastric cancer	neoplastic (AGS, KatoIII, NCI-N87, SNU-1, SNU-5, SNU-16) and non-neoplastic (HFE-145)	-2.32	SILAC	23161554
C14orf149	gastric cancer	neoplastic (AGS, KatoIII, NCI-N87, SNU-1, SNU-5, SNU-16) and non-neoplastic (HFE-145)	-2.94	SILAC	23161554
C14orf166	gastric cancer	neoplastic (AGS, KatoIII, NCI-N87, SNU-1, SNU-5, SNU-16) and non-neoplastic (HFE-145)	1.11	SILAC	23161554
C1R	gastric cancer	neoplastic (AGS, KatoIII, NCI-N87, SNU-1, SNU-5, SNU-16) and non-neoplastic (HFE-145)	-3.64	SILAC	23161554
C21orf33	gastric cancer	neoplastic (AGS, KatoIII, NCI-N87, SNU-1, SNU-5, SNU-16) and non-neoplastic (HFE-145)	-1.35	SILAC	23161554
C6orf108	gastric cancer	neoplastic (AGS, KatoIII, NCI-N87, SNU-1, SNU-5, SNU-16) and non-neoplastic (HFE-145)	1.29	SILAC	23161554
C7orf59	gastric cancer	neoplastic (AGS, KatoIII, NCI-N87, SNU-1, SNU-5, SNU-16) and non-neoplastic (HFE-145)	2.63	SILAC	23161554
CACYBP	gastric cancer	neoplastic (AGS, KatoIII, NCI-N87, SNU-1, SNU-5, SNU-16) and non-neoplastic (HFE-145)	1.22	SILAC	23161554
CAD	gastric cancer	neoplastic (AGS, KatoIII, NCI-N87, SNU-1, SNU-5, SNU-16) and non-neoplastic (HFE-145)	2.67	SILAC	23161554
CALR	gastric cancer	neoplastic (AGS, KatoIII, NCI-N87, SNU-1, SNU-5, SNU-16) and non-neoplastic (HFE-145)	1.62	SILAC	23161554
CANT1	gastric cancer	neoplastic (AGS, KatoIII, NCI-N87, SNU-1, SNU-5, SNU-16) and non-neoplastic (HFE-145)	1.18	SILAC	23161554
CAPG	gastric cancer	neoplastic (AGS, KatoIII, NCI-N87, SNU-1, SNU-5, SNU-16) and non-neoplastic (HFE-145)	1.87	SILAC	23161554
CAPN1	gastric cancer	neoplastic (AGS, KatoIII, NCI-N87, SNU-1, SNU-5, SNU-16) and non-neoplastic (HFE-145)	1.58	SILAC	23161554
CAPNS1	gastric cancer	neoplastic (AGS, KatoIII, NCI-N87, SNU-1, SNU-5, SNU-16) and non-neoplastic (HFE-145)	1.04	SILAC	23161554
CAPRIN1	gastric cancer	neoplastic (AGS, KatoIII, NCI-N87, SNU-1, SNU-5, SNU-16) and non-neoplastic (HFE-145)	1.20	SILAC	23161554
CAPZA1	gastric cancer	neoplastic (AGS, KatoIII, NCI-N87, SNU-1, SNU-5, SNU-16) and non-neoplastic (HFE-145)	1.02	SILAC	23161554
CARHSP1	gastric cancer	neoplastic (AGS, KatoIII, NCI-N87, SNU-1, SNU-5, SNU-16) and non-neoplastic (HFE-145)	1.23	SILAC	23161554

CARM1	gastric cancer	neoplastic (AGS, KatoIII, NCI-N87, SNU-1, SNU-5, SNU-16) and non-neoplastic (HFE-145)	1.40	SILAC	23161554
CASK	gastric cancer	neoplastic (AGS, KatoIII, NCI-N87, SNU-1, SNU-5, SNU-16) and non-neoplastic (HFE-145)	1.43	SILAC	23161554
CAST	gastric cancer	neoplastic (AGS, KatoIII, NCI-N87, SNU-1, SNU-5, SNU-16) and non-neoplastic (HFE-145)	1.29	SILAC	23161554
CBX3	gastric cancer	neoplastic (AGS, KatoIII, NCI-N87, SNU-1, SNU-5, SNU-16) and non-neoplastic (HFE-145)	1.63	SILAC	23161554
CD109	gastric cancer	neoplastic (AGS, KatoIII, NCI-N87, SNU-1, SNU-5, SNU-16) and non-neoplastic (HFE-145)	3.56	SILAC	23161554
CD2AP	gastric cancer	neoplastic (AGS, KatoIII, NCI-N87, SNU-1, SNU-5, SNU-16) and non-neoplastic (HFE-145)	2.15	SILAC	23161554
CD9	gastric cancer	neoplastic (AGS, KatoIII, NCI-N87, SNU-1, SNU-5, SNU-16) and non-neoplastic (HFE-145)	4.71	SILAC	23161554
CDC37	gastric cancer	neoplastic (AGS, KatoIII, NCI-N87, SNU-1, SNU-5, SNU-16) and non-neoplastic (HFE-145)	1.57	SILAC	23161554
CDC42	gastric cancer	neoplastic (AGS, KatoIII, NCI-N87, SNU-1, SNU-5, SNU-16) and non-neoplastic (HFE-145)	2.15	SILAC	23161554
CDH17	gastric cancer	neoplastic (AGS, KatoIII, NCI-N87, SNU-1, SNU-5, SNU-16) and non-neoplastic (HFE-145)	1.18	SILAC	23161554
CDH2	gastric cancer	neoplastic (AGS, KatoIII, NCI-N87, SNU-1, SNU-5, SNU-16) and non-neoplastic (HFE-145)	-2.47	SILAC	23161554
CDK2	gastric cancer	neoplastic (AGS, KatoIII, NCI-N87, SNU-1, SNU-5, SNU-16) and non-neoplastic (HFE-145)	-1.39	SILAC	23161554
CHI3L1	gastric cancer	neoplastic (AGS, KatoIII, NCI-N87, SNU-1, SNU-5, SNU-16) and non-neoplastic (HFE-145)	2.83	SILAC	23161554
CHMP4B	gastric cancer	neoplastic (AGS, KatoIII, NCI-N87, SNU-1, SNU-5, SNU-16) and non-neoplastic (HFE-145)	2.01	SILAC	23161554
CIAPIN1	gastric cancer	neoplastic (AGS, KatoIII, NCI-N87, SNU-1, SNU-5, SNU-16) and non-neoplastic (HFE-145)	-1.78	SILAC	23161554
CKAP5	gastric cancer	neoplastic (AGS, KatoIII, NCI-N87, SNU-1, SNU-5, SNU-16) and non-neoplastic (HFE-145)	-2.12	SILAC	23161554
CLASP1	gastric cancer	neoplastic (AGS, KatoIII, NCI-N87, SNU-1, SNU-5, SNU-16) and non-neoplastic (HFE-145)	-1.94	SILAC	23161554
CLEC3B	gastric cancer	neoplastic (AGS, KatoIII, NCI-N87, SNU-1, SNU-5, SNU-16) and non-neoplastic (HFE-145)	3.00	SILAC	23161554
CLIC1	gastric cancer	neoplastic (AGS, KatoIII, NCI-N87, SNU-1, SNU-5, SNU-16) and non-neoplastic (HFE-145)	1.16	SILAC	23161554
CLIC4	gastric cancer	neoplastic (AGS, KatoIII, NCI-N87, SNU-1, SNU-5, SNU-16) and non-neoplastic (HFE-145)	-2.32	SILAC	23161554
CLIP1	gastric cancer	neoplastic (AGS, KatoIII, NCI-N87, SNU-1, SNU-5, SNU-16) and non-neoplastic (HFE-145)	1.46	SILAC	23161554
CLSTN1	gastric cancer	neoplastic (AGS, KatoIII, NCI-N87, SNU-1, SNU-5, SNU-16) and non-neoplastic (HFE-145)	1.65	SILAC	23161554
CLSTN3	gastric cancer	neoplastic (AGS, KatoIII, NCI-N87, SNU-1, SNU-5, SNU-16) and non-neoplastic (HFE-145)	1.35	SILAC	23161554
CLTC	gastric cancer	neoplastic (AGS, KatoIII, NCI-N87, SNU-1, SNU-5, SNU-16) and non-neoplastic (HFE-145)	1.00	SILAC	23161554
CMBL	gastric cancer	neoplastic (AGS, KatoIII, NCI-N87, SNU-1, SNU-5, SNU-16) and non-neoplastic (HFE-145)	3.10	SILAC	23161554
CMPK1	gastric cancer	neoplastic (AGS, KatoIII, NCI-N87, SNU-1, SNU-5, SNU-16) and non-neoplastic (HFE-145)	1.05	SILAC	23161554
CNDP2	gastric cancer	neoplastic (AGS, KatoIII, NCI-N87, SNU-1, SNU-5, SNU-16) and non-neoplastic (HFE-145)	1.88	SILAC	23161554
CNPY2	gastric cancer	neoplastic (AGS, KatoIII, NCI-N87, SNU-1, SNU-5, SNU-16) and non-neoplastic (HFE-145)	1.50	SILAC	23161554
COL12A1	gastric cancer	neoplastic (AGS, KatoIII, NCI-N87, SNU-1, SNU-5, SNU-16) and non-neoplastic (HFE-145)	-4.32	SILAC	23161554
COL1A1	gastric cancer	neoplastic (AGS, KatoIII, NCI-N87, SNU-1, SNU-5, SNU-16) and non-neoplastic (HFE-145)	-2.47	SILAC	23161554
COL3A1	gastric cancer	neoplastic (AGS, KatoIII, NCI-N87, SNU-1, SNU-5, SNU-16) and non-neoplastic (HFE-145)	2.40	SILAC	23161554
COL6A1	gastric cancer	neoplastic (AGS, KatoIII, NCI-N87, SNU-1, SNU-5, SNU-16) and non-neoplastic (HFE-145)	-2.25	SILAC	23161554
COL6A2	gastric cancer	neoplastic (AGS, KatoIII, NCI-N87, SNU-1, SNU-5, SNU-16) and non-neoplastic (HFE-145)	-5.64	SILAC	23161554
COL6A3	gastric cancer	neoplastic (AGS, KatoIII, NCI-N87, SNU-1, SNU-5, SNU-16) and non-neoplastic (HFE-145)	-5.05	SILAC	23161554
COL7A1	gastric cancer	neoplastic (AGS, KatoIII, NCI-N87, SNU-1, SNU-5, SNU-16) and non-neoplastic (HFE-145)	-1.21	SILAC	23161554
COMMD3	gastric cancer	neoplastic (AGS, KatoIII, NCI-N87, SNU-1, SNU-5, SNU-16) and non-neoplastic (HFE-145)	1.74	SILAC	23161554
COPA	gastric cancer	neoplastic (AGS, KatoIII, NCI-N87, SNU-1, SNU-5, SNU-16) and non-neoplastic (HFE-145)	1.76	SILAC	23161554
COPB1	gastric cancer	neoplastic (AGS, KatoIII, NCI-N87, SNU-1, SNU-5, SNU-16) and non-neoplastic (HFE-145)	1.89	SILAC	23161554
COPG	gastric cancer	neoplastic (AGS, KatoIII, NCI-N87, SNU-1, SNU-5, SNU-16) and non-neoplastic (HFE-145)	2.04	SILAC	23161554
COPS6	gastric cancer	neoplastic (AGS, KatoIII, NCI-N87, SNU-1, SNU-5, SNU-16) and non-neoplastic (HFE-145)	-1.88	SILAC	23161554
COPZ1	gastric cancer	neoplastic (AGS, KatoIII, NCI-N87, SNU-1, SNU-5, SNU-16) and non-neoplastic (HFE-145)	1.64	SILAC	23161554
CORO1A	gastric cancer	neoplastic (AGS, KatoIII, NCI-N87, SNU-1, SNU-5, SNU-16) and non-neoplastic (HFE-145)	-1.94	SILAC	23161554
CORO7	gastric cancer	neoplastic (AGS, KatoIII, NCI-N87, SNU-1, SNU-5, SNU-16) and non-neoplastic (HFE-145)	2.20	SILAC	23161554

CPNE3	gastric cancer	neoplastic (AGS, KatoIII, NCI-N87, SNU-1, SNU-5, SNU-16) and non-neoplastic (HFE-145)	1.38	SILAC	23161554
CRYZ	gastric cancer	neoplastic (AGS, KatoIII, NCI-N87, SNU-1, SNU-5, SNU-16) and non-neoplastic (HFE-145)	1.67	SILAC	23161554
CS	gastric cancer	neoplastic (AGS, KatoIII, NCI-N87, SNU-1, SNU-5, SNU-16) and non-neoplastic (HFE-145)	-1.02	SILAC	23161554
CSF1	gastric cancer	neoplastic (AGS, KatoIII, NCI-N87, SNU-1, SNU-5, SNU-16) and non-neoplastic (HFE-145)	1.20	SILAC	23161554
CSPG4	gastric cancer	neoplastic (AGS, KatoIII, NCI-N87, SNU-1, SNU-5, SNU-16) and non-neoplastic (HFE-145)	-4.64	SILAC	23161554
CST3	gastric cancer	neoplastic (AGS, KatoIII, NCI-N87, SNU-1, SNU-5, SNU-16) and non-neoplastic (HFE-145)	2.58	SILAC	23161554
CSTB	gastric cancer	neoplastic (AGS, KatoIII, NCI-N87, SNU-1, SNU-5, SNU-16) and non-neoplastic (HFE-145)	2.06	SILAC	23161554
CTGF	gastric cancer	neoplastic (AGS, KatoIII, NCI-N87, SNU-1, SNU-5, SNU-16) and non-neoplastic (HFE-145)	-1.28	SILAC	23161554
CTNNBL1	gastric cancer	neoplastic (AGS, KatoIII, NCI-N87, SNU-1, SNU-5, SNU-16) and non-neoplastic (HFE-145)	-1	SILAC	23161554
CTSC	gastric cancer	neoplastic (AGS, KatoIII, NCI-N87, SNU-1, SNU-5, SNU-16) and non-neoplastic (HFE-145)	3.57	SILAC	23161554
CTSD	gastric cancer	neoplastic (AGS, KatoIII, NCI-N87, SNU-1, SNU-5, SNU-16) and non-neoplastic (HFE-145)	3.07	SILAC	23161554
CTSH	gastric cancer	neoplastic (AGS, KatoIII, NCI-N87, SNU-1, SNU-5, SNU-16) and non-neoplastic (HFE-145)	3.43	SILAC	23161554
CTSL2	gastric cancer	neoplastic (AGS, KatoIII, NCI-N87, SNU-1, SNU-5, SNU-16) and non-neoplastic (HFE-145)	2.86	SILAC	23161554
CTSZ	gastric cancer	neoplastic (AGS, KatoIII, NCI-N87, SNU-1, SNU-5, SNU-16) and non-neoplastic (HFE-145)	5.15	SILAC	23161554
CTTN	gastric cancer	neoplastic (AGS, KatoIII, NCI-N87, SNU-1, SNU-5, SNU-16) and non-neoplastic (HFE-145)	1.52	SILAC	23161554
CYFIP1	gastric cancer	neoplastic (AGS, KatoIII, NCI-N87, SNU-1, SNU-5, SNU-16) and non-neoplastic (HFE-145)	2.31	SILAC	23161554
CYR61	gastric cancer	neoplastic (AGS, KatoIII, NCI-N87, SNU-1, SNU-5, SNU-16) and non-neoplastic (HFE-145)	1.64	SILAC	23161554
DAG1	gastric cancer	neoplastic (AGS, KatoIII, NCI-N87, SNU-1, SNU-5, SNU-16) and non-neoplastic (HFE-145)	1.29	SILAC	23161554
DAK	gastric cancer	neoplastic (AGS, KatoIII, NCI-N87, SNU-1, SNU-5, SNU-16) and non-neoplastic (HFE-145)	2.18	SILAC	23161554
DARS	gastric cancer	neoplastic (AGS, KatoIII, NCI-N87, SNU-1, SNU-5, SNU-16) and non-neoplastic (HFE-145)	1.28	SILAC	23161554
DBI	gastric cancer	neoplastic (AGS, KatoIII, NCI-N87, SNU-1, SNU-5, SNU-16) and non-neoplastic (HFE-145)	1.98	SILAC	23161554
DBNL	gastric cancer	neoplastic (AGS, KatoIII, NCI-N87, SNU-1, SNU-5, SNU-16) and non-neoplastic (HFE-145)	1.97	SILAC	23161554
DCPS	gastric cancer	neoplastic (AGS, KatoIII, NCI-N87, SNU-1, SNU-5, SNU-16) and non-neoplastic (HFE-145)	2.12	SILAC	23161554
DCTD	gastric cancer	neoplastic (AGS, KatoIII, NCI-N87, SNU-1, SNU-5, SNU-16) and non-neoplastic (HFE-145)	2	SILAC	23161554
DCTPP1	gastric cancer	neoplastic (AGS, KatoIII, NCI-N87, SNU-1, SNU-5, SNU-16) and non-neoplastic (HFE-145)	1.60	SILAC	23161554
DDAH1	gastric cancer	neoplastic (AGS, KatoIII, NCI-N87, SNU-1, SNU-5, SNU-16) and non-neoplastic (HFE-145)	1.77	SILAC	23161554
DDB1	gastric cancer	neoplastic (AGS, KatoIII, NCI-N87, SNU-1, SNU-5, SNU-16) and non-neoplastic (HFE-145)	1.25	SILAC	23161554
DDT	gastric cancer	neoplastic (AGS, KatoIII, NCI-N87, SNU-1, SNU-5, SNU-16) and non-neoplastic (HFE-145)	1.65	SILAC	23161554
DDX21	gastric cancer	neoplastic (AGS, KatoIII, NCI-N87, SNU-1, SNU-5, SNU-16) and non-neoplastic (HFE-145)	1.95	SILAC	23161554
DDX3X	gastric cancer	neoplastic (AGS, KatoIII, NCI-N87, SNU-1, SNU-5, SNU-16) and non-neoplastic (HFE-145)	1.56	SILAC	23161554
DES	gastric cancer	neoplastic (AGS, KatoIII, NCI-N87, SNU-1, SNU-5, SNU-16) and non-neoplastic (HFE-145)	-3.32	SILAC	23161554
DHFR	gastric cancer	neoplastic (AGS, KatoIII, NCI-N87, SNU-1, SNU-5, SNU-16) and non-neoplastic (HFE-145)	3.31	SILAC	23161554
DHX15	gastric cancer	neoplastic (AGS, KatoIII, NCI-N87, SNU-1, SNU-5, SNU-16) and non-neoplastic (HFE-145)	1.02	SILAC	23161554
DKC1	gastric cancer	neoplastic (AGS, KatoIII, NCI-N87, SNU-1, SNU-5, SNU-16) and non-neoplastic (HFE-145)	1.22	SILAC	23161554
DNAJA1	gastric cancer	neoplastic (AGS, KatoIII, NCI-N87, SNU-1, SNU-5, SNU-16) and non-neoplastic (HFE-145)	1.86	SILAC	23161554
DNAJB1	gastric cancer	neoplastic (AGS, KatoIII, NCI-N87, SNU-1, SNU-5, SNU-16) and non-neoplastic (HFE-145)	1.58	SILAC	23161554
DNAJC9	gastric cancer	neoplastic (AGS, KatoIII, NCI-N87, SNU-1, SNU-5, SNU-16) and non-neoplastic (HFE-145)	1.08	SILAC	23161554
DNASE2	gastric cancer	neoplastic (AGS, KatoIII, NCI-N87, SNU-1, SNU-5, SNU-16) and non-neoplastic (HFE-145)	2.70	SILAC	23161554
DNM2	gastric cancer	neoplastic (AGS, KatoIII, NCI-N87, SNU-1, SNU-5, SNU-16) and non-neoplastic (HFE-145)	1.55	SILAC	23161554
DNMT1	gastric cancer	neoplastic (AGS, KatoIII, NCI-N87, SNU-1, SNU-5, SNU-16) and non-neoplastic (HFE-145)	1.59	SILAC	23161554
DNPEP	gastric cancer	neoplastic (AGS, KatoIII, NCI-N87, SNU-1, SNU-5, SNU-16) and non-neoplastic (HFE-145)	1.65	SILAC	23161554
DPYSL2	gastric cancer	neoplastic (AGS, KatoIII, NCI-N87, SNU-1, SNU-5, SNU-16) and non-neoplastic (HFE-145)	1.03	SILAC	23161554
DRG1	gastric cancer	neoplastic (AGS, KatoIII, NCI-N87, SNU-1, SNU-5, SNU-16) and non-neoplastic (HFE-145)	2.10	SILAC	23161554

DSG2	gastric cancer	neoplastic (AGS, KatoIII, NCI-N87, SNU-1, SNU-5, SNU-16) and non-neoplastic (HFE-145)	2.73	SILAC	23161554
DSTN	gastric cancer	neoplastic (AGS, KatoIII, NCI-N87, SNU-1, SNU-5, SNU-16) and non-neoplastic (HFE-145)	1.04	SILAC	23161554
DTYMK	gastric cancer	neoplastic (AGS, KatoIII, NCI-N87, SNU-1, SNU-5, SNU-16) and non-neoplastic (HFE-145)	-1.18	SILAC	23161554
DYNC1H1	gastric cancer	neoplastic (AGS, KatoIII, NCI-N87, SNU-1, SNU-5, SNU-16) and non-neoplastic (HFE-145)	1.81	SILAC	23161554
DYNC1I2	gastric cancer	neoplastic (AGS, KatoIII, NCI-N87, SNU-1, SNU-5, SNU-16) and non-neoplastic (HFE-145)	4.26	SILAC	23161554
DYNC1LI1	gastric cancer	neoplastic (AGS, KatoIII, NCI-N87, SNU-1, SNU-5, SNU-16) and non-neoplastic (HFE-145)	1.54	SILAC	23161554
DYNC1LI2	gastric cancer	neoplastic (AGS, KatoIII, NCI-N87, SNU-1, SNU-5, SNU-16) and non-neoplastic (HFE-145)	2.14	SILAC	23161554
DYNC2H1	gastric cancer	neoplastic (AGS, KatoIII, NCI-N87, SNU-1, SNU-5, SNU-16) and non-neoplastic (HFE-145)	1.08	SILAC	23161554
DYNLT1	gastric cancer	neoplastic (AGS, KatoIII, NCI-N87, SNU-1, SNU-5, SNU-16) and non-neoplastic (HFE-145)	1.46	SILAC	23161554
ECH1	gastric cancer	neoplastic (AGS, KatoIII, NCI-N87, SNU-1, SNU-5, SNU-16) and non-neoplastic (HFE-145)	1.99	SILAC	23161554
ECHS1	gastric cancer	neoplastic (AGS, KatoIII, NCI-N87, SNU-1, SNU-5, SNU-16) and non-neoplastic (HFE-145)	-1.08	SILAC	23161554
EEF1A1	gastric cancer	neoplastic (AGS, KatoIII, NCI-N87, SNU-1, SNU-5, SNU-16) and non-neoplastic (HFE-145)	1.48	SILAC	23161554
EEF1B2	gastric cancer	neoplastic (AGS, KatoIII, NCI-N87, SNU-1, SNU-5, SNU-16) and non-neoplastic (HFE-145)	1.68	SILAC	23161554
EEF1E1	gastric cancer	neoplastic (AGS, KatoIII, NCI-N87, SNU-1, SNU-5, SNU-16) and non-neoplastic (HFE-145)	1.17	SILAC	23161554
EGFR	gastric cancer	neoplastic (AGS, KatoIII, NCI-N87, SNU-1, SNU-5, SNU-16) and non-neoplastic (HFE-145)	2.93	SILAC	23161554
EHD1	gastric cancer	neoplastic (AGS, KatoIII, NCI-N87, SNU-1, SNU-5, SNU-16) and non-neoplastic (HFE-145)	1.66	SILAC	23161554
EIF1	gastric cancer	neoplastic (AGS, KatoIII, NCI-N87, SNU-1, SNU-5, SNU-16) and non-neoplastic (HFE-145)	2.37	SILAC	23161554
EIF1AY	gastric cancer	neoplastic (AGS, KatoIII, NCI-N87, SNU-1, SNU-5, SNU-16) and non-neoplastic (HFE-145)	1.65	SILAC	23161554
EIF2B4	gastric cancer	neoplastic (AGS, KatoIII, NCI-N87, SNU-1, SNU-5, SNU-16) and non-neoplastic (HFE-145)	1.66	SILAC	23161554
EIF2C2	gastric cancer	neoplastic (AGS, KatoIII, NCI-N87, SNU-1, SNU-5, SNU-16) and non-neoplastic (HFE-145)	1.76	SILAC	23161554
EIF2S1	gastric cancer	neoplastic (AGS, KatoIII, NCI-N87, SNU-1, SNU-5, SNU-16) and non-neoplastic (HFE-145)	1.15	SILAC	23161554
EIF2S2	gastric cancer	neoplastic (AGS, KatoIII, NCI-N87, SNU-1, SNU-5, SNU-16) and non-neoplastic (HFE-145)	1.57	SILAC	23161554
EIF2S3	gastric cancer	neoplastic (AGS, KatoIII, NCI-N87, SNU-1, SNU-5, SNU-16) and non-neoplastic (HFE-145)	1.31	SILAC	23161554
EIF3B	gastric cancer	neoplastic (AGS, KatoIII, NCI-N87, SNU-1, SNU-5, SNU-16) and non-neoplastic (HFE-145)	1.02	SILAC	23161554
EIF3J	gastric cancer	neoplastic (AGS, KatoIII, NCI-N87, SNU-1, SNU-5, SNU-16) and non-neoplastic (HFE-145)	2.16	SILAC	23161554
EIF4E	gastric cancer	neoplastic (AGS, KatoIII, NCI-N87, SNU-1, SNU-5, SNU-16) and non-neoplastic (HFE-145)	1.23	SILAC	23161554
EIF4H	gastric cancer	neoplastic (AGS, KatoIII, NCI-N87, SNU-1, SNU-5, SNU-16) and non-neoplastic (HFE-145)	2.81	SILAC	23161554
EIF5	gastric cancer	neoplastic (AGS, KatoIII, NCI-N87, SNU-1, SNU-5, SNU-16) and non-neoplastic (HFE-145)	2.38	SILAC	23161554
EIF5B	gastric cancer	neoplastic (AGS, KatoIII, NCI-N87, SNU-1, SNU-5, SNU-16) and non-neoplastic (HFE-145)	1.00	SILAC	23161554
EMG1	gastric cancer	neoplastic (AGS, KatoIII, NCI-N87, SNU-1, SNU-5, SNU-16) and non-neoplastic (HFE-145)	2.07	SILAC	23161554
EML4	gastric cancer	neoplastic (AGS, KatoIII, NCI-N87, SNU-1, SNU-5, SNU-16) and non-neoplastic (HFE-145)	2.33	SILAC	23161554
ENSA	gastric cancer	neoplastic (AGS, KatoIII, NCI-N87, SNU-1, SNU-5, SNU-16) and non-neoplastic (HFE-145)	1.22	SILAC	23161554
EPB41L2	gastric cancer	neoplastic (AGS, KatoIII, NCI-N87, SNU-1, SNU-5, SNU-16) and non-neoplastic (HFE-145)	2.07	SILAC	23161554
EPPK1	gastric cancer	neoplastic (AGS, KatoIII, NCI-N87, SNU-1, SNU-5, SNU-16) and non-neoplastic (HFE-145)	2.31	SILAC	23161554
EPRS	gastric cancer	neoplastic (AGS, KatoIII, NCI-N87, SNU-1, SNU-5, SNU-16) and non-neoplastic (HFE-145)	1.62	SILAC	23161554
ERAP1	gastric cancer	neoplastic (AGS, KatoIII, NCI-N87, SNU-1, SNU-5, SNU-16) and non-neoplastic (HFE-145)	1.81	SILAC	23161554
ERH	gastric cancer	neoplastic (AGS, KatoIII, NCI-N87, SNU-1, SNU-5, SNU-16) and non-neoplastic (HFE-145)	1.28	SILAC	23161554
ERP29	gastric cancer	neoplastic (AGS, KatoIII, NCI-N87, SNU-1, SNU-5, SNU-16) and non-neoplastic (HFE-145)	1.78	SILAC	23161554
ESD	gastric cancer	neoplastic (AGS, KatoIII, NCI-N87, SNU-1, SNU-5, SNU-16) and non-neoplastic (HFE-145)	1.38	SILAC	23161554
ETFA	gastric cancer	neoplastic (AGS, KatoIII, NCI-N87, SNU-1, SNU-5, SNU-16) and non-neoplastic (HFE-145)	1.13	SILAC	23161554
EZR	gastric cancer	neoplastic (AGS, KatoIII, NCI-N87, SNU-1, SNU-5, SNU-16) and non-neoplastic (HFE-145)	1.47	SILAC	23161554
FABP5	gastric cancer	neoplastic (AGS, KatoIII, NCI-N87, SNU-1, SNU-5, SNU-16) and non-neoplastic (HFE-145)	1.38	SILAC	23161554
FAM129B	gastric cancer	neoplastic (AGS, KatoIII, NCI-N87, SNU-1, SNU-5, SNU-16) and non-neoplastic (HFE-145)	1.28	SILAC	23161554

FAM3C	gastric cancer	neoplastic (AGS, KatoIII, NCI-N87, SNU-1, SNU-5, SNU-16) and non-neoplastic (HFE-145)	3.14	SILAC	23161554
FARSA	gastric cancer	neoplastic (AGS, KatoIII, NCI-N87, SNU-1, SNU-5, SNU-16) and non-neoplastic (HFE-145)	1.48	SILAC	23161554
FARSB	gastric cancer	neoplastic (AGS, KatoIII, NCI-N87, SNU-1, SNU-5, SNU-16) and non-neoplastic (HFE-145)	1.18	SILAC	23161554
FASN	gastric cancer	neoplastic (AGS, KatoIII, NCI-N87, SNU-1, SNU-5, SNU-16) and non-neoplastic (HFE-145)	3.03	SILAC	23161554
FAT1	gastric cancer	neoplastic (AGS, KatoIII, NCI-N87, SNU-1, SNU-5, SNU-16) and non-neoplastic (HFE-145)	2.99	SILAC	23161554
FBLN2	gastric cancer	neoplastic (AGS, KatoIII, NCI-N87, SNU-1, SNU-5, SNU-16) and non-neoplastic (HFE-145)	3.95	SILAC	23161554
FGFR2	gastric cancer	neoplastic (AGS, KatoIII, NCI-N87, SNU-1, SNU-5, SNU-16) and non-neoplastic (HFE-145)	5.62	SILAC	23161554
FKBP3	gastric cancer	neoplastic (AGS, KatoIII, NCI-N87, SNU-1, SNU-5, SNU-16) and non-neoplastic (HFE-145)	1	SILAC	23161554
FLNB	gastric cancer	neoplastic (AGS, KatoIII, NCI-N87, SNU-1, SNU-5, SNU-16) and non-neoplastic (HFE-145)	1.68	SILAC	23161554
FLNC	gastric cancer	neoplastic (AGS, KatoIII, NCI-N87, SNU-1, SNU-5, SNU-16) and non-neoplastic (HFE-145)	-3.64	SILAC	23161554
FN1	gastric cancer	neoplastic (AGS, KatoIII, NCI-N87, SNU-1, SNU-5, SNU-16) and non-neoplastic (HFE-145)	-2.73	SILAC	23161554
FSCN1	gastric cancer	neoplastic (AGS, KatoIII, NCI-N87, SNU-1, SNU-5, SNU-16) and non-neoplastic (HFE-145)	-1	SILAC	23161554
FSTL1	gastric cancer	neoplastic (AGS, KatoIII, NCI-N87, SNU-1, SNU-5, SNU-16) and non-neoplastic (HFE-145)	-4.64	SILAC	23161554
FTH1	gastric cancer	neoplastic (AGS, KatoIII, NCI-N87, SNU-1, SNU-5, SNU-16) and non-neoplastic (HFE-145)	1.25	SILAC	23161554
FUBP1	gastric cancer	neoplastic (AGS, KatoIII, NCI-N87, SNU-1, SNU-5, SNU-16) and non-neoplastic (HFE-145)	1.28	SILAC	23161554
FUCA1	gastric cancer	neoplastic (AGS, KatoIII, NCI-N87, SNU-1, SNU-5, SNU-16) and non-neoplastic (HFE-145)	2	SILAC	23161554
FUCA2	gastric cancer	neoplastic (AGS, KatoIII, NCI-N87, SNU-1, SNU-5, SNU-16) and non-neoplastic (HFE-145)	1.93	SILAC	23161554
G3BP2	gastric cancer	neoplastic (AGS, KatoIII, NCI-N87, SNU-1, SNU-5, SNU-16) and non-neoplastic (HFE-145)	1.58	SILAC	23161554
GALNS	gastric cancer	neoplastic (AGS, KatoIII, NCI-N87, SNU-1, SNU-5, SNU-16) and non-neoplastic (HFE-145)	2.93	SILAC	23161554
GARS	gastric cancer	neoplastic (AGS, KatoIII, NCI-N87, SNU-1, SNU-5, SNU-16) and non-neoplastic (HFE-145)	2.10	SILAC	23161554
GAS6	gastric cancer	neoplastic (AGS, KatoIII, NCI-N87, SNU-1, SNU-5, SNU-16) and non-neoplastic (HFE-145)	1.55	SILAC	23161554
GCN1L1	gastric cancer	neoplastic (AGS, KatoIII, NCI-N87, SNU-1, SNU-5, SNU-16) and non-neoplastic (HFE-145)	3.03	SILAC	23161554
GDI2	gastric cancer	neoplastic (AGS, KatoIII, NCI-N87, SNU-1, SNU-5, SNU-16) and non-neoplastic (HFE-145)	1.25	SILAC	23161554
GEMIN4	gastric cancer	neoplastic (AGS, KatoIII, NCI-N87, SNU-1, SNU-5, SNU-16) and non-neoplastic (HFE-145)	2.16	SILAC	23161554
GFPT1	gastric cancer	neoplastic (AGS, KatoIII, NCI-N87, SNU-1, SNU-5, SNU-16) and non-neoplastic (HFE-145)	3.10	SILAC	23161554
GGCT	gastric cancer	neoplastic (AGS, KatoIII, NCI-N87, SNU-1, SNU-5, SNU-16) and non-neoplastic (HFE-145)	1.95	SILAC	23161554
GGH	gastric cancer	neoplastic (AGS, KatoIII, NCI-N87, SNU-1, SNU-5, SNU-16) and non-neoplastic (HFE-145)	1.59	SILAC	23161554
GLA	gastric cancer	neoplastic (AGS, KatoIII, NCI-N87, SNU-1, SNU-5, SNU-16) and non-neoplastic (HFE-145)	1.87	SILAC	23161554
GLRX	gastric cancer	neoplastic (AGS, KatoIII, NCI-N87, SNU-1, SNU-5, SNU-16) and non-neoplastic (HFE-145)	2.28	SILAC	23161554
GLRX3	gastric cancer	neoplastic (AGS, KatoIII, NCI-N87, SNU-1, SNU-5, SNU-16) and non-neoplastic (HFE-145)	1.07	SILAC	23161554
GMDS	gastric cancer	neoplastic (AGS, KatoIII, NCI-N87, SNU-1, SNU-5, SNU-16) and non-neoplastic (HFE-145)	2.68	SILAC	23161554
GMFB	gastric cancer	neoplastic (AGS, KatoIII, NCI-N87, SNU-1, SNU-5, SNU-16) and non-neoplastic (HFE-145)	1.36	SILAC	23161554
GNPDA1	gastric cancer	neoplastic (AGS, KatoIII, NCI-N87, SNU-1, SNU-5, SNU-16) and non-neoplastic (HFE-145)	1.16	SILAC	23161554
GNPNAT1	gastric cancer	neoplastic (AGS, KatoIII, NCI-N87, SNU-1, SNU-5, SNU-16) and non-neoplastic (HFE-145)	1.67	SILAC	23161554
GNS	gastric cancer	neoplastic (AGS, KatoIII, NCI-N87, SNU-1, SNU-5, SNU-16) and non-neoplastic (HFE-145)	2.07	SILAC	23161554
GORASP2	gastric cancer	neoplastic (AGS, KatoIII, NCI-N87, SNU-1, SNU-5, SNU-16) and non-neoplastic (HFE-145)	1.72	SILAC	23161554
GOT2	gastric cancer	neoplastic (AGS, KatoIII, NCI-N87, SNU-1, SNU-5, SNU-16) and non-neoplastic (HFE-145)	-1.02	SILAC	23161554
GPC1	gastric cancer	neoplastic (AGS, KatoIII, NCI-N87, SNU-1, SNU-5, SNU-16) and non-neoplastic (HFE-145)	1.37	SILAC	23161554
GPD1L	gastric cancer	neoplastic (AGS, KatoIII, NCI-N87, SNU-1, SNU-5, SNU-16) and non-neoplastic (HFE-145)	2.35	SILAC	23161554
GPR126	gastric cancer	neoplastic (AGS, KatoIII, NCI-N87, SNU-1, SNU-5, SNU-16) and non-neoplastic (HFE-145)	2.82	SILAC	23161554
GPX4	gastric cancer	neoplastic (AGS, KatoIII, NCI-N87, SNU-1, SNU-5, SNU-16) and non-neoplastic (HFE-145)	1.61	SILAC	23161554
GSN	gastric cancer	neoplastic (AGS, KatoIII, NCI-N87, SNU-1, SNU-5, SNU-16) and non-neoplastic (HFE-145)	1.40	SILAC	23161554
GSPT1	gastric cancer	neoplastic (AGS, KatoIII, NCI-N87, SNU-1, SNU-5, SNU-16) and non-neoplastic (HFE-145)	1.02	SILAC	23161554

GSR	gastric cancer	neoplastic (AGS, KatoIII, NCI-N87, SNU-1, SNU-5, SNU-16) and non-neoplastic (HFE-145)	1.98	SILAC	23161554
GSTM1	gastric cancer	neoplastic (AGS, KatoIII, NCI-N87, SNU-1, SNU-5, SNU-16) and non-neoplastic (HFE-145)	1.67	SILAC	23161554
GSTM3	gastric cancer	neoplastic (AGS, KatoIII, NCI-N87, SNU-1, SNU-5, SNU-16) and non-neoplastic (HFE-145)	1.23	SILAC	23161554
GSTO1	gastric cancer	neoplastic (AGS, KatoIII, NCI-N87, SNU-1, SNU-5, SNU-16) and non-neoplastic (HFE-145)	1.64	SILAC	23161554
GSTP1	gastric cancer	neoplastic (AGS, KatoIII, NCI-N87, SNU-1, SNU-5, SNU-16) and non-neoplastic (HFE-145)	1.22	SILAC	23161554
GSTT1	gastric cancer	neoplastic (AGS, KatoIII, NCI-N87, SNU-1, SNU-5, SNU-16) and non-neoplastic (HFE-145)	-1.32	SILAC	23161554
GTF2I	gastric cancer	neoplastic (AGS, KatoIII, NCI-N87, SNU-1, SNU-5, SNU-16) and non-neoplastic (HFE-145)	2.02	SILAC	23161554
GYS1	gastric cancer	neoplastic (AGS, KatoIII, NCI-N87, SNU-1, SNU-5, SNU-16) and non-neoplastic (HFE-145)	1.70	SILAC	23161554
H2AFY	gastric cancer	neoplastic (AGS, KatoIII, NCI-N87, SNU-1, SNU-5, SNU-16) and non-neoplastic (HFE-145)	1.33	SILAC	23161554
H2AFZ	gastric cancer	neoplastic (AGS, KatoIII, NCI-N87, SNU-1, SNU-5, SNU-16) and non-neoplastic (HFE-145)	-1.47	SILAC	23161554
HDGF	gastric cancer	neoplastic (AGS, KatoIII, NCI-N87, SNU-1, SNU-5, SNU-16) and non-neoplastic (HFE-145)	1.55	SILAC	23161554
HDLBP	gastric cancer	neoplastic (AGS, KatoIII, NCI-N87, SNU-1, SNU-5, SNU-16) and non-neoplastic (HFE-145)	1.10	SILAC	23161554
HEBP2	gastric cancer	neoplastic (AGS, KatoIII, NCI-N87, SNU-1, SNU-5, SNU-16) and non-neoplastic (HFE-145)	2.26	SILAC	23161554
HEXA	gastric cancer	neoplastic (AGS, KatoIII, NCI-N87, SNU-1, SNU-5, SNU-16) and non-neoplastic (HFE-145)	1.41	SILAC	23161554
HEXB	gastric cancer	neoplastic (AGS, KatoIII, NCI-N87, SNU-1, SNU-5, SNU-16) and non-neoplastic (HFE-145)	1.07	SILAC	23161554
HIBADH	gastric cancer	neoplastic (AGS, KatoIII, NCI-N87, SNU-1, SNU-5, SNU-16) and non-neoplastic (HFE-145)	1.32	SILAC	23161554
HIST1H2AJ	gastric cancer	neoplastic (AGS, KatoIII, NCI-N87, SNU-1, SNU-5, SNU-16) and non-neoplastic (HFE-145)	-1.55	SILAC	23161554
HIST1H2BB	gastric cancer	neoplastic (AGS, KatoIII, NCI-N87, SNU-1, SNU-5, SNU-16) and non-neoplastic (HFE-145)	-1.51	SILAC	23161554
HIST1H2BD	gastric cancer	neoplastic (AGS, KatoIII, NCI-N87, SNU-1, SNU-5, SNU-16) and non-neoplastic (HFE-145)	-1.12	SILAC	23161554
HIST1H3F	gastric cancer	neoplastic (AGS, KatoIII, NCI-N87, SNU-1, SNU-5, SNU-16) and non-neoplastic (HFE-145)	-1.59	SILAC	23161554
HIST1H4I	gastric cancer	neoplastic (AGS, KatoIII, NCI-N87, SNU-1, SNU-5, SNU-16) and non-neoplastic (HFE-145)	-1.32	SILAC	23161554
HK1	gastric cancer	neoplastic (AGS, KatoIII, NCI-N87, SNU-1, SNU-5, SNU-16) and non-neoplastic (HFE-145)	1.66	SILAC	23161554
HK2	gastric cancer	neoplastic (AGS, KatoIII, NCI-N87, SNU-1, SNU-5, SNU-16) and non-neoplastic (HFE-145)	2.24	SILAC	23161554
HLA-A	gastric cancer	neoplastic (AGS, KatoIII, NCI-N87, SNU-1, SNU-5, SNU-16) and non-neoplastic (HFE-145)	3.09	SILAC	23161554
HMBS	gastric cancer	neoplastic (AGS, KatoIII, NCI-N87, SNU-1, SNU-5, SNU-16) and non-neoplastic (HFE-145)	1.26	SILAC	23161554
HMGB2	gastric cancer	neoplastic (AGS, KatoIII, NCI-N87, SNU-1, SNU-5, SNU-16) and non-neoplastic (HFE-145)	1.54	SILAC	23161554
HMGB3	gastric cancer	neoplastic (AGS, KatoIII, NCI-N87, SNU-1, SNU-5, SNU-16) and non-neoplastic (HFE-145)	1.18	SILAC	23161554
HMGCS1	gastric cancer	neoplastic (AGS, KatoIII, NCI-N87, SNU-1, SNU-5, SNU-16) and non-neoplastic (HFE-145)	4.42	SILAC	23161554
HMGN3	gastric cancer	neoplastic (AGS, KatoIII, NCI-N87, SNU-1, SNU-5, SNU-16) and non-neoplastic (HFE-145)	2.60	SILAC	23161554
HMGN4	gastric cancer	neoplastic (AGS, KatoIII, NCI-N87, SNU-1, SNU-5, SNU-16) and non-neoplastic (HFE-145)	2.83	SILAC	23161554
HN1	gastric cancer	neoplastic (AGS, KatoIII, NCI-N87, SNU-1, SNU-5, SNU-16) and non-neoplastic (HFE-145)	1.57	SILAC	23161554
HNRNPA3	gastric cancer	neoplastic (AGS, KatoIII, NCI-N87, SNU-1, SNU-5, SNU-16) and non-neoplastic (HFE-145)	1.23	SILAC	23161554
HNRNPC	gastric cancer	neoplastic (AGS, KatoIII, NCI-N87, SNU-1, SNU-5, SNU-16) and non-neoplastic (HFE-145)	1.66	SILAC	23161554
HNRNPF	gastric cancer	neoplastic (AGS, KatoIII, NCI-N87, SNU-1, SNU-5, SNU-16) and non-neoplastic (HFE-145)	1.80	SILAC	23161554
HNRNPH1	gastric cancer	neoplastic (AGS, KatoIII, NCI-N87, SNU-1, SNU-5, SNU-16) and non-neoplastic (HFE-145)	2.66	SILAC	23161554
HNRNPH2	gastric cancer	neoplastic (AGS, KatoIII, NCI-N87, SNU-1, SNU-5, SNU-16) and non-neoplastic (HFE-145)	2.92	SILAC	23161554
HNRNPM	gastric cancer	neoplastic (AGS, KatoIII, NCI-N87, SNU-1, SNU-5, SNU-16) and non-neoplastic (HFE-145)	1.35	SILAC	23161554
HNRNPUL1	gastric cancer	neoplastic (AGS, KatoIII, NCI-N87, SNU-1, SNU-5, SNU-16) and non-neoplastic (HFE-145)	2.92	SILAC	23161554
HRSP12	gastric cancer	neoplastic (AGS, KatoIII, NCI-N87, SNU-1, SNU-5, SNU-16) and non-neoplastic (HFE-145)	1.13	SILAC	23161554
HSD3B1	gastric cancer	neoplastic (AGS, KatoIII, NCI-N87, SNU-1, SNU-5, SNU-16) and non-neoplastic (HFE-145)	1.29	SILAC	23161554
HSP90AB1	gastric cancer	neoplastic (AGS, KatoIII, NCI-N87, SNU-1, SNU-5, SNU-16) and non-neoplastic (HFE-145)	1.06	SILAC	23161554
HSP90B1	gastric cancer	neoplastic (AGS, KatoIII, NCI-N87, SNU-1, SNU-5, SNU-16) and non-neoplastic (HFE-145)	1.54	SILAC	23161554
HSPA14	gastric cancer	neoplastic (AGS, KatoIII, NCI-N87, SNU-1, SNU-5, SNU-16) and non-neoplastic (HFE-145)	3.14	SILAC	23161554

HSPA1L	gastric cancer	neoplastic (AGS, KatoIII, NCI-N87, SNU-1, SNU-5, SNU-16) and non-neoplastic (HFE-145)	1.20	SILAC	23161554
HSPA4L	gastric cancer	neoplastic (AGS, KatoIII, NCI-N87, SNU-1, SNU-5, SNU-16) and non-neoplastic (HFE-145)	4.00	SILAC	23161554
HSPA5	gastric cancer	neoplastic (AGS, KatoIII, NCI-N87, SNU-1, SNU-5, SNU-16) and non-neoplastic (HFE-145)	2.27	SILAC	23161554
HSPA8	gastric cancer	neoplastic (AGS, KatoIII, NCI-N87, SNU-1, SNU-5, SNU-16) and non-neoplastic (HFE-145)	1.02	SILAC	23161554
HSPB1	gastric cancer	neoplastic (AGS, KatoIII, NCI-N87, SNU-1, SNU-5, SNU-16) and non-neoplastic (HFE-145)	3.19	SILAC	23161554
HSPD1	gastric cancer	neoplastic (AGS, KatoIII, NCI-N87, SNU-1, SNU-5, SNU-16) and non-neoplastic (HFE-145)	2.43	SILAC	23161554
HSPG2	gastric cancer	neoplastic (AGS, KatoIII, NCI-N87, SNU-1, SNU-5, SNU-16) and non-neoplastic (HFE-145)	3.07	SILAC	23161554
HSPH1	gastric cancer	neoplastic (AGS, KatoIII, NCI-N87, SNU-1, SNU-5, SNU-16) and non-neoplastic (HFE-145)	2.75	SILAC	23161554
HTRA1	gastric cancer	neoplastic (AGS, KatoIII, NCI-N87, SNU-1, SNU-5, SNU-16) and non-neoplastic (HFE-145)	-1.05	SILAC	23161554
HUWE1	gastric cancer	neoplastic (AGS, KatoIII, NCI-N87, SNU-1, SNU-5, SNU-16) and non-neoplastic (HFE-145)	1.41	SILAC	23161554
HYOU1	gastric cancer	neoplastic (AGS, KatoIII, NCI-N87, SNU-1, SNU-5, SNU-16) and non-neoplastic (HFE-145)	1.25	SILAC	23161554
IARS	gastric cancer	neoplastic (AGS, KatoIII, NCI-N87, SNU-1, SNU-5, SNU-16) and non-neoplastic (HFE-145)	1.41	SILAC	23161554
IDE	gastric cancer	neoplastic (AGS, KatoIII, NCI-N87, SNU-1, SNU-5, SNU-16) and non-neoplastic (HFE-145)	2	SILAC	23161554
IDH1	gastric cancer	neoplastic (AGS, KatoIII, NCI-N87, SNU-1, SNU-5, SNU-16) and non-neoplastic (HFE-145)	2.50	SILAC	23161554
IDI1	gastric cancer	neoplastic (AGS, KatoIII, NCI-N87, SNU-1, SNU-5, SNU-16) and non-neoplastic (HFE-145)	1.45	SILAC	23161554
IFITM3	gastric cancer	neoplastic (AGS, KatoIII, NCI-N87, SNU-1, SNU-5, SNU-16) and non-neoplastic (HFE-145)	2.23	SILAC	23161554
IGF2R	gastric cancer	neoplastic (AGS, KatoIII, NCI-N87, SNU-1, SNU-5, SNU-16) and non-neoplastic (HFE-145)	1.37	SILAC	23161554
IGFBP4	gastric cancer	neoplastic (AGS, KatoIII, NCI-N87, SNU-1, SNU-5, SNU-16) and non-neoplastic (HFE-145)	1.61	SILAC	23161554
IGFBP6	gastric cancer	neoplastic (AGS, KatoIII, NCI-N87, SNU-1, SNU-5, SNU-16) and non-neoplastic (HFE-145)	3.03	SILAC	23161554
IL8	gastric cancer	neoplastic (AGS, KatoIII, NCI-N87, SNU-1, SNU-5, SNU-16) and non-neoplastic (HFE-145)	-4.32	SILAC	23161554
IMPDH2	gastric cancer	neoplastic (AGS, KatoIII, NCI-N87, SNU-1, SNU-5, SNU-16) and non-neoplastic (HFE-145)	2.71	SILAC	23161554
ING5	gastric cancer	neoplastic (AGS, KatoIII, NCI-N87, SNU-1, SNU-5, SNU-16) and non-neoplastic (HFE-145)	1.43	SILAC	23161554
INPP1	gastric cancer	neoplastic (AGS, KatoIII, NCI-N87, SNU-1, SNU-5, SNU-16) and non-neoplastic (HFE-145)	2.65	SILAC	23161554
IPO4	gastric cancer	neoplastic (AGS, KatoIII, NCI-N87, SNU-1, SNU-5, SNU-16) and non-neoplastic (HFE-145)	1.64	SILAC	23161554
IRF2BP1	gastric cancer	neoplastic (AGS, KatoIII, NCI-N87, SNU-1, SNU-5, SNU-16) and non-neoplastic (HFE-145)	4.95	SILAC	23161554
ISOC1	gastric cancer	neoplastic (AGS, KatoIII, NCI-N87, SNU-1, SNU-5, SNU-16) and non-neoplastic (HFE-145)	1.24	SILAC	23161554
ITGA6	gastric cancer	neoplastic (AGS, KatoIII, NCI-N87, SNU-1, SNU-5, SNU-16) and non-neoplastic (HFE-145)	2.87	SILAC	23161554
KARS	gastric cancer	neoplastic (AGS, KatoIII, NCI-N87, SNU-1, SNU-5, SNU-16) and non-neoplastic (HFE-145)	2.51	SILAC	23161554
KHSRP	gastric cancer	neoplastic (AGS, KatoIII, NCI-N87, SNU-1, SNU-5, SNU-16) and non-neoplastic (HFE-145)	1	SILAC	23161554
KIAA0368	gastric cancer	neoplastic (AGS, KatoIII, NCI-N87, SNU-1, SNU-5, SNU-16) and non-neoplastic (HFE-145)	1.10	SILAC	23161554
KIAA0664	gastric cancer	neoplastic (AGS, KatoIII, NCI-N87, SNU-1, SNU-5, SNU-16) and non-neoplastic (HFE-145)	2.12	SILAC	23161554
KIAA1199	gastric cancer	neoplastic (AGS, KatoIII, NCI-N87, SNU-1, SNU-5, SNU-16) and non-neoplastic (HFE-145)	2.48	SILAC	23161554
KIF5B	gastric cancer	neoplastic (AGS, KatoIII, NCI-N87, SNU-1, SNU-5, SNU-16) and non-neoplastic (HFE-145)	1.00	SILAC	23161554
KLK10	gastric cancer	neoplastic (AGS, KatoIII, NCI-N87, SNU-1, SNU-5, SNU-16) and non-neoplastic (HFE-145)	2.04	SILAC	23161554
KLK11	gastric cancer	neoplastic (AGS, KatoIII, NCI-N87, SNU-1, SNU-5, SNU-16) and non-neoplastic (HFE-145)	4.18	SILAC	23161554
KLK6	gastric cancer	neoplastic (AGS, KatoIII, NCI-N87, SNU-1, SNU-5, SNU-16) and non-neoplastic (HFE-145)	5.37	SILAC	23161554
KRT10	gastric cancer	neoplastic (AGS, KatoIII, NCI-N87, SNU-1, SNU-5, SNU-16) and non-neoplastic (HFE-145)	1.71	SILAC	23161554
KRT8	gastric cancer	neoplastic (AGS, KatoIII, NCI-N87, SNU-1, SNU-5, SNU-16) and non-neoplastic (HFE-145)	3.94	SILAC	23161554
LACTB2	gastric cancer	neoplastic (AGS, KatoIII, NCI-N87, SNU-1, SNU-5, SNU-16) and non-neoplastic (HFE-145)	1.45	SILAC	23161554
LAGE3	gastric cancer	neoplastic (AGS, KatoIII, NCI-N87, SNU-1, SNU-5, SNU-16) and non-neoplastic (HFE-145)	1.54	SILAC	23161554
LAMA4	gastric cancer	neoplastic (AGS, KatoIII, NCI-N87, SNU-1, SNU-5, SNU-16) and non-neoplastic (HFE-145)	-3.18	SILAC	23161554
LAMA5	gastric cancer	neoplastic (AGS, KatoIII, NCI-N87, SNU-1, SNU-5, SNU-16) and non-neoplastic (HFE-145)	5.22	SILAC	23161554
LAMC1	gastric cancer	neoplastic (AGS, KatoIII, NCI-N87, SNU-1, SNU-5, SNU-16) and non-neoplastic (HFE-145)	1.06	SILAC	23161554

LAMP1	gastric cancer	neoplastic (AGS, KatoIII, NCI-N87, SNU-1, SNU-5, SNU-16) and non-neoplastic (HFE-145)	1.04	SILAC	23161554
LAMP2	gastric cancer	neoplastic (AGS, KatoIII, NCI-N87, SNU-1, SNU-5, SNU-16) and non-neoplastic (HFE-145)	1.20	SILAC	23161554
LAMTOR3	gastric cancer	neoplastic (AGS, KatoIII, NCI-N87, SNU-1, SNU-5, SNU-16) and non-neoplastic (HFE-145)	2.06	SILAC	23161554
LAP3	gastric cancer	neoplastic (AGS, KatoIII, NCI-N87, SNU-1, SNU-5, SNU-16) and non-neoplastic (HFE-145)	1.75	SILAC	23161554
LARS	gastric cancer	neoplastic (AGS, KatoIII, NCI-N87, SNU-1, SNU-5, SNU-16) and non-neoplastic (HFE-145)	2.09	SILAC	23161554
LCN2	gastric cancer	neoplastic (AGS, KatoIII, NCI-N87, SNU-1, SNU-5, SNU-16) and non-neoplastic (HFE-145)	3.54	SILAC	23161554
LDLR	gastric cancer	neoplastic (AGS, KatoIII, NCI-N87, SNU-1, SNU-5, SNU-16) and non-neoplastic (HFE-145)	2.55	SILAC	23161554
LGALS1	gastric cancer	neoplastic (AGS, KatoIII, NCI-N87, SNU-1, SNU-5, SNU-16) and non-neoplastic (HFE-145)	-1.88	SILAC	23161554
LGALS3	gastric cancer	neoplastic (AGS, KatoIII, NCI-N87, SNU-1, SNU-5, SNU-16) and non-neoplastic (HFE-145)	3.15	SILAC	23161554
LGALS3BP	gastric cancer	neoplastic (AGS, KatoIII, NCI-N87, SNU-1, SNU-5, SNU-16) and non-neoplastic (HFE-145)	2.83	SILAC	23161554
LGALS4	gastric cancer	neoplastic (AGS, KatoIII, NCI-N87, SNU-1, SNU-5, SNU-16) and non-neoplastic (HFE-145)	5.27	SILAC	23161554
LGMN	gastric cancer	neoplastic (AGS, KatoIII, NCI-N87, SNU-1, SNU-5, SNU-16) and non-neoplastic (HFE-145)	3.71	SILAC	23161554
LIN7C	gastric cancer	neoplastic (AGS, KatoIII, NCI-N87, SNU-1, SNU-5, SNU-16) and non-neoplastic (HFE-145)	1.10	SILAC	23161554
LIPA	gastric cancer	neoplastic (AGS, KatoIII, NCI-N87, SNU-1, SNU-5, SNU-16) and non-neoplastic (HFE-145)	1.14	SILAC	23161554
LMAN2	gastric cancer	neoplastic (AGS, KatoIII, NCI-N87, SNU-1, SNU-5, SNU-16) and non-neoplastic (HFE-145)	2.58	SILAC	23161554
LMNA	gastric cancer	neoplastic (AGS, KatoIII, NCI-N87, SNU-1, SNU-5, SNU-16) and non-neoplastic (HFE-145)	2.73	SILAC	23161554
LMNB1	gastric cancer	neoplastic (AGS, KatoIII, NCI-N87, SNU-1, SNU-5, SNU-16) and non-neoplastic (HFE-145)	-1.28	SILAC	23161554
LMNB2	gastric cancer	neoplastic (AGS, KatoIII, NCI-N87, SNU-1, SNU-5, SNU-16) and non-neoplastic (HFE-145)	-2.73	SILAC	23161554
LOC10050550	gastric cancer	neoplastic (AGS, KatoIII, NCI-N87, SNU-1, SNU-5, SNU-16) and non-neoplastic (HFE-145)	2.00	SILAC	23161554
LOC10050769	gastric cancer	neoplastic (AGS, KatoIII, NCI-N87, SNU-1, SNU-5, SNU-16) and non-neoplastic (HFE-145)	1.28	SILAC	23161554
LOXL2	gastric cancer	neoplastic (AGS, KatoIII, NCI-N87, SNU-1, SNU-5, SNU-16) and non-neoplastic (HFE-145)	-4.64	SILAC	23161554
LPP	gastric cancer	neoplastic (AGS, KatoIII, NCI-N87, SNU-1, SNU-5, SNU-16) and non-neoplastic (HFE-145)	1.18	SILAC	23161554
LRP1	gastric cancer	neoplastic (AGS, KatoIII, NCI-N87, SNU-1, SNU-5, SNU-16) and non-neoplastic (HFE-145)	1.32	SILAC	23161554
LRPPRC	gastric cancer	neoplastic (AGS, KatoIII, NCI-N87, SNU-1, SNU-5, SNU-16) and non-neoplastic (HFE-145)	-1.02	SILAC	23161554
LRRC59	gastric cancer	neoplastic (AGS, KatoIII, NCI-N87, SNU-1, SNU-5, SNU-16) and non-neoplastic (HFE-145)	1.40	SILAC	23161554
LRRFIP1	gastric cancer	neoplastic (AGS, KatoIII, NCI-N87, SNU-1, SNU-5, SNU-16) and non-neoplastic (HFE-145)	2.38	SILAC	23161554
LSM5	gastric cancer	neoplastic (AGS, KatoIII, NCI-N87, SNU-1, SNU-5, SNU-16) and non-neoplastic (HFE-145)	1.03	SILAC	23161554
LTA4H	gastric cancer	neoplastic (AGS, KatoIII, NCI-N87, SNU-1, SNU-5, SNU-16) and non-neoplastic (HFE-145)	1.42	SILAC	23161554
LUC7L2	gastric cancer	neoplastic (AGS, KatoIII, NCI-N87, SNU-1, SNU-5, SNU-16) and non-neoplastic (HFE-145)	1.84	SILAC	23161554
LXN	gastric cancer	neoplastic (AGS, KatoIII, NCI-N87, SNU-1, SNU-5, SNU-16) and non-neoplastic (HFE-145)	-1.73	SILAC	23161554
LYZ	gastric cancer	neoplastic (AGS, KatoIII, NCI-N87, SNU-1, SNU-5, SNU-16) and non-neoplastic (HFE-145)	4.00	SILAC	23161554
MANBA	gastric cancer	neoplastic (AGS, KatoIII, NCI-N87, SNU-1, SNU-5, SNU-16) and non-neoplastic (HFE-145)	3.41	SILAC	23161554
MAP2K1	gastric cancer	neoplastic (AGS, KatoIII, NCI-N87, SNU-1, SNU-5, SNU-16) and non-neoplastic (HFE-145)	1.67	SILAC	23161554
MARCKS	gastric cancer	neoplastic (AGS, KatoIII, NCI-N87, SNU-1, SNU-5, SNU-16) and non-neoplastic (HFE-145)	1.03	SILAC	23161554
MARS	gastric cancer	neoplastic (AGS, KatoIII, NCI-N87, SNU-1, SNU-5, SNU-16) and non-neoplastic (HFE-145)	1.05	SILAC	23161554
MCF2	gastric cancer	neoplastic (AGS, KatoIII, NCI-N87, SNU-1, SNU-5, SNU-16) and non-neoplastic (HFE-145)	2.14	SILAC	23161554
MCFD2	gastric cancer	neoplastic (AGS, KatoIII, NCI-N87, SNU-1, SNU-5, SNU-16) and non-neoplastic (HFE-145)	2.09	SILAC	23161554
MCM3	gastric cancer	neoplastic (AGS, KatoIII, NCI-N87, SNU-1, SNU-5, SNU-16) and non-neoplastic (HFE-145)	1.65	SILAC	23161554
MCM4	gastric cancer	neoplastic (AGS, KatoIII, NCI-N87, SNU-1, SNU-5, SNU-16) and non-neoplastic (HFE-145)	1.35	SILAC	23161554
MCM6	gastric cancer	neoplastic (AGS, KatoIII, NCI-N87, SNU-1, SNU-5, SNU-16) and non-neoplastic (HFE-145)	1.31	SILAC	23161554
MCM7	gastric cancer	neoplastic (AGS, KatoIII, NCI-N87, SNU-1, SNU-5, SNU-16) and non-neoplastic (HFE-145)	1.04	SILAC	23161554
MCTS1	gastric cancer	neoplastic (AGS, KatoIII, NCI-N87, SNU-1, SNU-5, SNU-16) and non-neoplastic (HFE-145)	1.24	SILAC	23161554
MEMO1	gastric cancer	neoplastic (AGS, KatoIII, NCI-N87, SNU-1, SNU-5, SNU-16) and non-neoplastic (HFE-145)	1.36	SILAC	23161554

MET	gastric cancer	neoplastic (AGS, KatoIII, NCI-N87, SNU-1, SNU-5, SNU-16) and non-neoplastic (HFE-145)	2.85	SILAC	23161554
METAP1	gastric cancer	neoplastic (AGS, KatoIII, NCI-N87, SNU-1, SNU-5, SNU-16) and non-neoplastic (HFE-145)	2.04	SILAC	23161554
METTL11A	gastric cancer	neoplastic (AGS, KatoIII, NCI-N87, SNU-1, SNU-5, SNU-16) and non-neoplastic (HFE-145)	2.67	SILAC	23161554
MGAT5	gastric cancer	neoplastic (AGS, KatoIII, NCI-N87, SNU-1, SNU-5, SNU-16) and non-neoplastic (HFE-145)	1.66	SILAC	23161554
MMP1	gastric cancer	neoplastic (AGS, KatoIII, NCI-N87, SNU-1, SNU-5, SNU-16) and non-neoplastic (HFE-145)	-5.05	SILAC	23161554
MMS19	gastric cancer	neoplastic (AGS, KatoIII, NCI-N87, SNU-1, SNU-5, SNU-16) and non-neoplastic (HFE-145)	1.11	SILAC	23161554
MRTO4	gastric cancer	neoplastic (AGS, KatoIII, NCI-N87, SNU-1, SNU-5, SNU-16) and non-neoplastic (HFE-145)	1.19	SILAC	23161554
MSN	gastric cancer	neoplastic (AGS, KatoIII, NCI-N87, SNU-1, SNU-5, SNU-16) and non-neoplastic (HFE-145)	-2.25	SILAC	23161554
MSRA	gastric cancer	neoplastic (AGS, KatoIII, NCI-N87, SNU-1, SNU-5, SNU-16) and non-neoplastic (HFE-145)	1.20	SILAC	23161554
MTHFD1	gastric cancer	neoplastic (AGS, KatoIII, NCI-N87, SNU-1, SNU-5, SNU-16) and non-neoplastic (HFE-145)	1.83	SILAC	23161554
MVD	gastric cancer	neoplastic (AGS, KatoIII, NCI-N87, SNU-1, SNU-5, SNU-16) and non-neoplastic (HFE-145)	3.01	SILAC	23161554
MVP	gastric cancer	neoplastic (AGS, KatoIII, NCI-N87, SNU-1, SNU-5, SNU-16) and non-neoplastic (HFE-145)	3.34	SILAC	23161554
MYADM	gastric cancer	neoplastic (AGS, KatoIII, NCI-N87, SNU-1, SNU-5, SNU-16) and non-neoplastic (HFE-145)	2.03	SILAC	23161554
MYH14	gastric cancer	neoplastic (AGS, KatoIII, NCI-N87, SNU-1, SNU-5, SNU-16) and non-neoplastic (HFE-145)	4.46	SILAC	23161554
MYH9	gastric cancer	neoplastic (AGS, KatoIII, NCI-N87, SNU-1, SNU-5, SNU-16) and non-neoplastic (HFE-145)	2.54	SILAC	23161554
MYL12A	gastric cancer	neoplastic (AGS, KatoIII, NCI-N87, SNU-1, SNU-5, SNU-16) and non-neoplastic (HFE-145)	2.06	SILAC	23161554
MYL6	gastric cancer	neoplastic (AGS, KatoIII, NCI-N87, SNU-1, SNU-5, SNU-16) and non-neoplastic (HFE-145)	1.69	SILAC	23161554
NAA10	gastric cancer	neoplastic (AGS, KatoIII, NCI-N87, SNU-1, SNU-5, SNU-16) and non-neoplastic (HFE-145)	1.31	SILAC	23161554
NAGLU	gastric cancer	neoplastic (AGS, KatoIII, NCI-N87, SNU-1, SNU-5, SNU-16) and non-neoplastic (HFE-145)	1.26	SILAC	23161554
NAMPT	gastric cancer	neoplastic (AGS, KatoIII, NCI-N87, SNU-1, SNU-5, SNU-16) and non-neoplastic (HFE-145)	1.68	SILAC	23161554
NBL1	gastric cancer	neoplastic (AGS, KatoIII, NCI-N87, SNU-1, SNU-5, SNU-16) and non-neoplastic (HFE-145)	1.60	SILAC	23161554
NCKAP1	gastric cancer	neoplastic (AGS, KatoIII, NCI-N87, SNU-1, SNU-5, SNU-16) and non-neoplastic (HFE-145)	2.48	SILAC	23161554
NCL	gastric cancer	neoplastic (AGS, KatoIII, NCI-N87, SNU-1, SNU-5, SNU-16) and non-neoplastic (HFE-145)	1.71	SILAC	23161554
NDRG1	gastric cancer	neoplastic (AGS, KatoIII, NCI-N87, SNU-1, SNU-5, SNU-16) and non-neoplastic (HFE-145)	2.32	SILAC	23161554
NEO1	gastric cancer	neoplastic (AGS, KatoIII, NCI-N87, SNU-1, SNU-5, SNU-16) and non-neoplastic (HFE-145)	2.40	SILAC	23161554
NID1	gastric cancer	neoplastic (AGS, KatoIII, NCI-N87, SNU-1, SNU-5, SNU-16) and non-neoplastic (HFE-145)	-2.39	SILAC	23161554
NIPSNAP3A	gastric cancer	neoplastic (AGS, KatoIII, NCI-N87, SNU-1, SNU-5, SNU-16) and non-neoplastic (HFE-145)	-1.43	SILAC	23161554
NLE1	gastric cancer	neoplastic (AGS, KatoIII, NCI-N87, SNU-1, SNU-5, SNU-16) and non-neoplastic (HFE-145)	1.19	SILAC	23161554
NME1-NME2	gastric cancer	neoplastic (AGS, KatoIII, NCI-N87, SNU-1, SNU-5, SNU-16) and non-neoplastic (HFE-145)	1.00	SILAC	23161554
NOMO2	gastric cancer	neoplastic (AGS, KatoIII, NCI-N87, SNU-1, SNU-5, SNU-16) and non-neoplastic (HFE-145)	2.32	SILAC	23161554
NOP56	gastric cancer	neoplastic (AGS, KatoIII, NCI-N87, SNU-1, SNU-5, SNU-16) and non-neoplastic (HFE-145)	2.27	SILAC	23161554
NPC2	gastric cancer	neoplastic (AGS, KatoIII, NCI-N87, SNU-1, SNU-5, SNU-16) and non-neoplastic (HFE-145)	2.78	SILAC	23161554
NPM1	gastric cancer	neoplastic (AGS, KatoIII, NCI-N87, SNU-1, SNU-5, SNU-16) and non-neoplastic (HFE-145)	1.40	SILAC	23161554
NPM3	gastric cancer	neoplastic (AGS, KatoIII, NCI-N87, SNU-1, SNU-5, SNU-16) and non-neoplastic (HFE-145)	1.00	SILAC	23161554
NQO1	gastric cancer	neoplastic (AGS, KatoIII, NCI-N87, SNU-1, SNU-5, SNU-16) and non-neoplastic (HFE-145)	2.81	SILAC	23161554
NSUN2	gastric cancer	neoplastic (AGS, KatoIII, NCI-N87, SNU-1, SNU-5, SNU-16) and non-neoplastic (HFE-145)	2.37	SILAC	23161554
NT5DC1	gastric cancer	neoplastic (AGS, KatoIII, NCI-N87, SNU-1, SNU-5, SNU-16) and non-neoplastic (HFE-145)	1.65	SILAC	23161554
NUCB2	gastric cancer	neoplastic (AGS, KatoIII, NCI-N87, SNU-1, SNU-5, SNU-16) and non-neoplastic (HFE-145)	1.57	SILAC	23161554
NUDT5	gastric cancer	neoplastic (AGS, KatoIII, NCI-N87, SNU-1, SNU-5, SNU-16) and non-neoplastic (HFE-145)	1.83	SILAC	23161554
NUMA1	gastric cancer	neoplastic (AGS, KatoIII, NCI-N87, SNU-1, SNU-5, SNU-16) and non-neoplastic (HFE-145)	-1.02	SILAC	23161554
NXN	gastric cancer	neoplastic (AGS, KatoIII, NCI-N87, SNU-1, SNU-5, SNU-16) and non-neoplastic (HFE-145)	1.33	SILAC	23161554
OAF	gastric cancer	neoplastic (AGS, KatoIII, NCI-N87, SNU-1, SNU-5, SNU-16) and non-neoplastic (HFE-145)	2.30	SILAC	23161554
OLA1	gastric cancer	neoplastic (AGS, KatoIII, NCI-N87, SNU-1, SNU-5, SNU-16) and non-neoplastic (HFE-145)	1.37	SILAC	23161554

OSBP	gastric cancer	neoplastic (AGS, KatoIII, NCI-N87, SNU-1, SNU-5, SNU-16) and non-neoplastic (HFE-145)	1.20	SILAC	23161554
OTOF	gastric cancer	neoplastic (AGS, KatoIII, NCI-N87, SNU-1, SNU-5, SNU-16) and non-neoplastic (HFE-145)	4.09	SILAC	23161554
OXSR1	gastric cancer	neoplastic (AGS, KatoIII, NCI-N87, SNU-1, SNU-5, SNU-16) and non-neoplastic (HFE-145)	1.76	SILAC	23161554
P4HB	gastric cancer	neoplastic (AGS, KatoIII, NCI-N87, SNU-1, SNU-5, SNU-16) and non-neoplastic (HFE-145)	1.70	SILAC	23161554
PABPC1	gastric cancer	neoplastic (AGS, KatoIII, NCI-N87, SNU-1, SNU-5, SNU-16) and non-neoplastic (HFE-145)	1.22	SILAC	23161554
PABPC4	gastric cancer	neoplastic (AGS, KatoIII, NCI-N87, SNU-1, SNU-5, SNU-16) and non-neoplastic (HFE-145)	2	SILAC	23161554
PAFAH1B2	gastric cancer	neoplastic (AGS, KatoIII, NCI-N87, SNU-1, SNU-5, SNU-16) and non-neoplastic (HFE-145)	1.29	SILAC	23161554
PAICS	gastric cancer	neoplastic (AGS, KatoIII, NCI-N87, SNU-1, SNU-5, SNU-16) and non-neoplastic (HFE-145)	1.13	SILAC	23161554
PAK2	gastric cancer	neoplastic (AGS, KatoIII, NCI-N87, SNU-1, SNU-5, SNU-16) and non-neoplastic (HFE-145)	2.30	SILAC	23161554
PARP4	gastric cancer	neoplastic (AGS, KatoIII, NCI-N87, SNU-1, SNU-5, SNU-16) and non-neoplastic (HFE-145)	2.55	SILAC	23161554
PARVA	gastric cancer	neoplastic (AGS, KatoIII, NCI-N87, SNU-1, SNU-5, SNU-16) and non-neoplastic (HFE-145)	-1.83	SILAC	23161554
PCBD1	gastric cancer	neoplastic (AGS, KatoIII, NCI-N87, SNU-1, SNU-5, SNU-16) and non-neoplastic (HFE-145)	3.35	SILAC	23161554
PCBP1	gastric cancer	neoplastic (AGS, KatoIII, NCI-N87, SNU-1, SNU-5, SNU-16) and non-neoplastic (HFE-145)	1.43	SILAC	23161554
PDAP1	gastric cancer	neoplastic (AGS, KatoIII, NCI-N87, SNU-1, SNU-5, SNU-16) and non-neoplastic (HFE-145)	2.37	SILAC	23161554
PDCD10	gastric cancer	neoplastic (AGS, KatoIII, NCI-N87, SNU-1, SNU-5, SNU-16) and non-neoplastic (HFE-145)	1.27	SILAC	23161554
PDCD2L	gastric cancer	neoplastic (AGS, KatoIII, NCI-N87, SNU-1, SNU-5, SNU-16) and non-neoplastic (HFE-145)	1.43	SILAC	23161554
PDCD6IP	gastric cancer	neoplastic (AGS, KatoIII, NCI-N87, SNU-1, SNU-5, SNU-16) and non-neoplastic (HFE-145)	1.05	SILAC	23161554
PDCL3	gastric cancer	neoplastic (AGS, KatoIII, NCI-N87, SNU-1, SNU-5, SNU-16) and non-neoplastic (HFE-145)	1.22	SILAC	23161554
PDGFC	gastric cancer	neoplastic (AGS, KatoIII, NCI-N87, SNU-1, SNU-5, SNU-16) and non-neoplastic (HFE-145)	2.61	SILAC	23161554
PDIA3	gastric cancer	neoplastic (AGS, KatoIII, NCI-N87, SNU-1, SNU-5, SNU-16) and non-neoplastic (HFE-145)	2.47	SILAC	23161554
PDIA4	gastric cancer	neoplastic (AGS, KatoIII, NCI-N87, SNU-1, SNU-5, SNU-16) and non-neoplastic (HFE-145)	2.17	SILAC	23161554
PDIA6	gastric cancer	neoplastic (AGS, KatoIII, NCI-N87, SNU-1, SNU-5, SNU-16) and non-neoplastic (HFE-145)	1.78	SILAC	23161554
PDLIM1	gastric cancer	neoplastic (AGS, KatoIII, NCI-N87, SNU-1, SNU-5, SNU-16) and non-neoplastic (HFE-145)	1.83	SILAC	23161554
PEBP1	gastric cancer	neoplastic (AGS, KatoIII, NCI-N87, SNU-1, SNU-5, SNU-16) and non-neoplastic (HFE-145)	1.05	SILAC	23161554
PFDN4	gastric cancer	neoplastic (AGS, KatoIII, NCI-N87, SNU-1, SNU-5, SNU-16) and non-neoplastic (HFE-145)	1.23	SILAC	23161554
PFKL	gastric cancer	neoplastic (AGS, KatoIII, NCI-N87, SNU-1, SNU-5, SNU-16) and non-neoplastic (HFE-145)	1.26	SILAC	23161554
PFKP	gastric cancer	neoplastic (AGS, KatoIII, NCI-N87, SNU-1, SNU-5, SNU-16) and non-neoplastic (HFE-145)	1.05	SILAC	23161554
PFN2	gastric cancer	neoplastic (AGS, KatoIII, NCI-N87, SNU-1, SNU-5, SNU-16) and non-neoplastic (HFE-145)	-1.05	SILAC	23161554
PHF5A	gastric cancer	neoplastic (AGS, KatoIII, NCI-N87, SNU-1, SNU-5, SNU-16) and non-neoplastic (HFE-145)	1.41	SILAC	23161554
PHGDH	gastric cancer	neoplastic (AGS, KatoIII, NCI-N87, SNU-1, SNU-5, SNU-16) and non-neoplastic (HFE-145)	1.33	SILAC	23161554
PKM2	gastric cancer	neoplastic (AGS, KatoIII, NCI-N87, SNU-1, SNU-5, SNU-16) and non-neoplastic (HFE-145)	1.69	SILAC	23161554
PLAT	gastric cancer	neoplastic (AGS, KatoIII, NCI-N87, SNU-1, SNU-5, SNU-16) and non-neoplastic (HFE-145)	-1.59	SILAC	23161554
PLCB3	gastric cancer	neoplastic (AGS, KatoIII, NCI-N87, SNU-1, SNU-5, SNU-16) and non-neoplastic (HFE-145)	1.87	SILAC	23161554
PLIN3	gastric cancer	neoplastic (AGS, KatoIII, NCI-N87, SNU-1, SNU-5, SNU-16) and non-neoplastic (HFE-145)	3.04	SILAC	23161554
PLOD2	gastric cancer	neoplastic (AGS, KatoIII, NCI-N87, SNU-1, SNU-5, SNU-16) and non-neoplastic (HFE-145)	-3.18	SILAC	23161554
PLOD3	gastric cancer	neoplastic (AGS, KatoIII, NCI-N87, SNU-1, SNU-5, SNU-16) and non-neoplastic (HFE-145)	3.43	SILAC	23161554
PLTP	gastric cancer	neoplastic (AGS, KatoIII, NCI-N87, SNU-1, SNU-5, SNU-16) and non-neoplastic (HFE-145)	1.38	SILAC	23161554
PMM2	gastric cancer	neoplastic (AGS, KatoIII, NCI-N87, SNU-1, SNU-5, SNU-16) and non-neoplastic (HFE-145)	1.56	SILAC	23161554
PMVK	gastric cancer	neoplastic (AGS, KatoIII, NCI-N87, SNU-1, SNU-5, SNU-16) and non-neoplastic (HFE-145)	1.58	SILAC	23161554
PNP	gastric cancer	neoplastic (AGS, KatoIII, NCI-N87, SNU-1, SNU-5, SNU-16) and non-neoplastic (HFE-145)	2.54	SILAC	23161554
POLDIP2	gastric cancer	neoplastic (AGS, KatoIII, NCI-N87, SNU-1, SNU-5, SNU-16) and non-neoplastic (HFE-145)	-1.32	SILAC	23161554
POLR2B	gastric cancer	neoplastic (AGS, KatoIII, NCI-N87, SNU-1, SNU-5, SNU-16) and non-neoplastic (HFE-145)	1.79	SILAC	23161554
POMP	gastric cancer	neoplastic (AGS, KatoIII, NCI-N87, SNU-1, SNU-5, SNU-16) and non-neoplastic (HFE-145)	2.26	SILAC	23161554

POSTN	gastric cancer	neoplastic (AGS, KatoIII, NCI-N87, SNU-1, SNU-5, SNU-16) and non-neoplastic (HFE-145)	1.20	SILAC	23161554
PPA1	gastric cancer	neoplastic (AGS, KatoIII, NCI-N87, SNU-1, SNU-5, SNU-16) and non-neoplastic (HFE-145)	2.14	SILAC	23161554
PPIA	gastric cancer	neoplastic (AGS, KatoIII, NCI-N87, SNU-1, SNU-5, SNU-16) and non-neoplastic (HFE-145)	1.43	SILAC	23161554
PPIB	gastric cancer	neoplastic (AGS, KatoIII, NCI-N87, SNU-1, SNU-5, SNU-16) and non-neoplastic (HFE-145)	2	SILAC	23161554
PPIF	gastric cancer	neoplastic (AGS, KatoIII, NCI-N87, SNU-1, SNU-5, SNU-16) and non-neoplastic (HFE-145)	-2.32	SILAC	23161554
PPIL3	gastric cancer	neoplastic (AGS, KatoIII, NCI-N87, SNU-1, SNU-5, SNU-16) and non-neoplastic (HFE-145)	1.00	SILAC	23161554
PPM1F	gastric cancer	neoplastic (AGS, KatoIII, NCI-N87, SNU-1, SNU-5, SNU-16) and non-neoplastic (HFE-145)	-1.18	SILAC	23161554
PPM1G	gastric cancer	neoplastic (AGS, KatoIII, NCI-N87, SNU-1, SNU-5, SNU-16) and non-neoplastic (HFE-145)	1.00	SILAC	23161554
PPP1CC	gastric cancer	neoplastic (AGS, KatoIII, NCI-N87, SNU-1, SNU-5, SNU-16) and non-neoplastic (HFE-145)	1.62	SILAC	23161554
PPP1R8	gastric cancer	neoplastic (AGS, KatoIII, NCI-N87, SNU-1, SNU-5, SNU-16) and non-neoplastic (HFE-145)	1.58	SILAC	23161554
PPP2R2A	gastric cancer	neoplastic (AGS, KatoIII, NCI-N87, SNU-1, SNU-5, SNU-16) and non-neoplastic (HFE-145)	2.12	SILAC	23161554
PPP2R5D	gastric cancer	neoplastic (AGS, KatoIII, NCI-N87, SNU-1, SNU-5, SNU-16) and non-neoplastic (HFE-145)	1.46	SILAC	23161554
PPP4C	gastric cancer	neoplastic (AGS, KatoIII, NCI-N87, SNU-1, SNU-5, SNU-16) and non-neoplastic (HFE-145)	1.62	SILAC	23161554
PPP5C	gastric cancer	neoplastic (AGS, KatoIII, NCI-N87, SNU-1, SNU-5, SNU-16) and non-neoplastic (HFE-145)	1.08	SILAC	23161554
PPT1	gastric cancer	neoplastic (AGS, KatoIII, NCI-N87, SNU-1, SNU-5, SNU-16) and non-neoplastic (HFE-145)	1.27	SILAC	23161554
PRDX1	gastric cancer	neoplastic (AGS, KatoIII, NCI-N87, SNU-1, SNU-5, SNU-16) and non-neoplastic (HFE-145)	1.57	SILAC	23161554
PRDX2	gastric cancer	neoplastic (AGS, KatoIII, NCI-N87, SNU-1, SNU-5, SNU-16) and non-neoplastic (HFE-145)	2.43	SILAC	23161554
PRDX3	gastric cancer	neoplastic (AGS, KatoIII, NCI-N87, SNU-1, SNU-5, SNU-16) and non-neoplastic (HFE-145)	-1.35	SILAC	23161554
PRDX4	gastric cancer	neoplastic (AGS, KatoIII, NCI-N87, SNU-1, SNU-5, SNU-16) and non-neoplastic (HFE-145)	1.85	SILAC	23161554
PRDX5	gastric cancer	neoplastic (AGS, KatoIII, NCI-N87, SNU-1, SNU-5, SNU-16) and non-neoplastic (HFE-145)	1.72	SILAC	23161554
PREP	gastric cancer	neoplastic (AGS, KatoIII, NCI-N87, SNU-1, SNU-5, SNU-16) and non-neoplastic (HFE-145)	1.64	SILAC	23161554
PRKDC	gastric cancer	neoplastic (AGS, KatoIII, NCI-N87, SNU-1, SNU-5, SNU-16) and non-neoplastic (HFE-145)	1.19	SILAC	23161554
PRMT1	gastric cancer	neoplastic (AGS, KatoIII, NCI-N87, SNU-1, SNU-5, SNU-16) and non-neoplastic (HFE-145)	1.06	SILAC	23161554
PROCR	gastric cancer	neoplastic (AGS, KatoIII, NCI-N87, SNU-1, SNU-5, SNU-16) and non-neoplastic (HFE-145)	1.15	SILAC	23161554
PRPF39	gastric cancer	neoplastic (AGS, KatoIII, NCI-N87, SNU-1, SNU-5, SNU-16) and non-neoplastic (HFE-145)	1.32	SILAC	23161554
PRPS1	gastric cancer	neoplastic (AGS, KatoIII, NCI-N87, SNU-1, SNU-5, SNU-16) and non-neoplastic (HFE-145)	1.12	SILAC	23161554
PRPS2	gastric cancer	neoplastic (AGS, KatoIII, NCI-N87, SNU-1, SNU-5, SNU-16) and non-neoplastic (HFE-145)	2.44	SILAC	23161554
PRPSAP1	gastric cancer	neoplastic (AGS, KatoIII, NCI-N87, SNU-1, SNU-5, SNU-16) and non-neoplastic (HFE-145)	-1.83	SILAC	23161554
PRPSAP2	gastric cancer	neoplastic (AGS, KatoIII, NCI-N87, SNU-1, SNU-5, SNU-16) and non-neoplastic (HFE-145)	-1.28	SILAC	23161554
PRSS23	gastric cancer	neoplastic (AGS, KatoIII, NCI-N87, SNU-1, SNU-5, SNU-16) and non-neoplastic (HFE-145)	1.36	SILAC	23161554
PRTFDC1	gastric cancer	neoplastic (AGS, KatoIII, NCI-N87, SNU-1, SNU-5, SNU-16) and non-neoplastic (HFE-145)	2.26	SILAC	23161554
PSAP	gastric cancer	neoplastic (AGS, KatoIII, NCI-N87, SNU-1, SNU-5, SNU-16) and non-neoplastic (HFE-145)	1.29	SILAC	23161554
PSAT1	gastric cancer	neoplastic (AGS, KatoIII, NCI-N87, SNU-1, SNU-5, SNU-16) and non-neoplastic (HFE-145)	1.50	SILAC	23161554
PSMA1	gastric cancer	neoplastic (AGS, KatoIII, NCI-N87, SNU-1, SNU-5, SNU-16) and non-neoplastic (HFE-145)	1.61	SILAC	23161554
PSMA2	gastric cancer	neoplastic (AGS, KatoIII, NCI-N87, SNU-1, SNU-5, SNU-16) and non-neoplastic (HFE-145)	1.70	SILAC	23161554
PSMA3	gastric cancer	neoplastic (AGS, KatoIII, NCI-N87, SNU-1, SNU-5, SNU-16) and non-neoplastic (HFE-145)	1.50	SILAC	23161554
PSMA4	gastric cancer	neoplastic (AGS, KatoIII, NCI-N87, SNU-1, SNU-5, SNU-16) and non-neoplastic (HFE-145)	1.57	SILAC	23161554
PSMA5	gastric cancer	neoplastic (AGS, KatoIII, NCI-N87, SNU-1, SNU-5, SNU-16) and non-neoplastic (HFE-145)	1.77	SILAC	23161554
PSMA6	gastric cancer	neoplastic (AGS, KatoIII, NCI-N87, SNU-1, SNU-5, SNU-16) and non-neoplastic (HFE-145)	1.51	SILAC	23161554
PSMA7	gastric cancer	neoplastic (AGS, KatoIII, NCI-N87, SNU-1, SNU-5, SNU-16) and non-neoplastic (HFE-145)	1.69	SILAC	23161554
PSMB1	gastric cancer	neoplastic (AGS, KatoIII, NCI-N87, SNU-1, SNU-5, SNU-16) and non-neoplastic (HFE-145)	1.45	SILAC	23161554
PSMB10	gastric cancer	neoplastic (AGS, KatoIII, NCI-N87, SNU-1, SNU-5, SNU-16) and non-neoplastic (HFE-145)	2.60	SILAC	23161554
PSMB2	gastric cancer	neoplastic (AGS, KatoIII, NCI-N87, SNU-1, SNU-5, SNU-16) and non-neoplastic (HFE-145)	1.62	SILAC	23161554

PSMB3	gastric cancer	neoplastic (AGS, KatoIII, NCI-N87, SNU-1, SNU-5, SNU-16) and non-neoplastic (HFE-145)	1.61	SILAC	23161554
PSMB4	gastric cancer	neoplastic (AGS, KatoIII, NCI-N87, SNU-1, SNU-5, SNU-16) and non-neoplastic (HFE-145)	1.48	SILAC	23161554
PSMB5	gastric cancer	neoplastic (AGS, KatoIII, NCI-N87, SNU-1, SNU-5, SNU-16) and non-neoplastic (HFE-145)	1.25	SILAC	23161554
PSMB6	gastric cancer	neoplastic (AGS, KatoIII, NCI-N87, SNU-1, SNU-5, SNU-16) and non-neoplastic (HFE-145)	1.32	SILAC	23161554
PSMB7	gastric cancer	neoplastic (AGS, KatoIII, NCI-N87, SNU-1, SNU-5, SNU-16) and non-neoplastic (HFE-145)	1.51	SILAC	23161554
PSMB8	gastric cancer	neoplastic (AGS, KatoIII, NCI-N87, SNU-1, SNU-5, SNU-16) and non-neoplastic (HFE-145)	2.25	SILAC	23161554
PSMC1	gastric cancer	neoplastic (AGS, KatoIII, NCI-N87, SNU-1, SNU-5, SNU-16) and non-neoplastic (HFE-145)	1.46	SILAC	23161554
PSMC2	gastric cancer	neoplastic (AGS, KatoIII, NCI-N87, SNU-1, SNU-5, SNU-16) and non-neoplastic (HFE-145)	1.45	SILAC	23161554
PSMC3	gastric cancer	neoplastic (AGS, KatoIII, NCI-N87, SNU-1, SNU-5, SNU-16) and non-neoplastic (HFE-145)	1.50	SILAC	23161554
PSMC4	gastric cancer	neoplastic (AGS, KatoIII, NCI-N87, SNU-1, SNU-5, SNU-16) and non-neoplastic (HFE-145)	1.70	SILAC	23161554
PSMC5	gastric cancer	neoplastic (AGS, KatoIII, NCI-N87, SNU-1, SNU-5, SNU-16) and non-neoplastic (HFE-145)	1.32	SILAC	23161554
PSMC6	gastric cancer	neoplastic (AGS, KatoIII, NCI-N87, SNU-1, SNU-5, SNU-16) and non-neoplastic (HFE-145)	1.61	SILAC	23161554
PSMD1	gastric cancer	neoplastic (AGS, KatoIII, NCI-N87, SNU-1, SNU-5, SNU-16) and non-neoplastic (HFE-145)	1.28	SILAC	23161554
PSMD11	gastric cancer	neoplastic (AGS, KatoIII, NCI-N87, SNU-1, SNU-5, SNU-16) and non-neoplastic (HFE-145)	1.60	SILAC	23161554
PSMD12	gastric cancer	neoplastic (AGS, KatoIII, NCI-N87, SNU-1, SNU-5, SNU-16) and non-neoplastic (HFE-145)	1.73	SILAC	23161554
PSMD13	gastric cancer	neoplastic (AGS, KatoIII, NCI-N87, SNU-1, SNU-5, SNU-16) and non-neoplastic (HFE-145)	1.46	SILAC	23161554
PSMD14	gastric cancer	neoplastic (AGS, KatoIII, NCI-N87, SNU-1, SNU-5, SNU-16) and non-neoplastic (HFE-145)	1.53	SILAC	23161554
PSMD2	gastric cancer	neoplastic (AGS, KatoIII, NCI-N87, SNU-1, SNU-5, SNU-16) and non-neoplastic (HFE-145)	1.25	SILAC	23161554
PSMD3	gastric cancer	neoplastic (AGS, KatoIII, NCI-N87, SNU-1, SNU-5, SNU-16) and non-neoplastic (HFE-145)	1.51	SILAC	23161554
PSMD6	gastric cancer	neoplastic (AGS, KatoIII, NCI-N87, SNU-1, SNU-5, SNU-16) and non-neoplastic (HFE-145)	1.32	SILAC	23161554
PSMD7	gastric cancer	neoplastic (AGS, KatoIII, NCI-N87, SNU-1, SNU-5, SNU-16) and non-neoplastic (HFE-145)	1.56	SILAC	23161554
PSMD8	gastric cancer	neoplastic (AGS, KatoIII, NCI-N87, SNU-1, SNU-5, SNU-16) and non-neoplastic (HFE-145)	1.22	SILAC	23161554
PSME3	gastric cancer	neoplastic (AGS, KatoIII, NCI-N87, SNU-1, SNU-5, SNU-16) and non-neoplastic (HFE-145)	1.24	SILAC	23161554
PSMG1	gastric cancer	neoplastic (AGS, KatoIII, NCI-N87, SNU-1, SNU-5, SNU-16) and non-neoplastic (HFE-145)	1.69	SILAC	23161554
PSMG3	gastric cancer	neoplastic (AGS, KatoIII, NCI-N87, SNU-1, SNU-5, SNU-16) and non-neoplastic (HFE-145)	1.59	SILAC	23161554
PSMG4	gastric cancer	neoplastic (AGS, KatoIII, NCI-N87, SNU-1, SNU-5, SNU-16) and non-neoplastic (HFE-145)	1.37	SILAC	23161554
PSPH	gastric cancer	neoplastic (AGS, KatoIII, NCI-N87, SNU-1, SNU-5, SNU-16) and non-neoplastic (HFE-145)	2.38	SILAC	23161554
PTGFRN	gastric cancer	neoplastic (AGS, KatoIII, NCI-N87, SNU-1, SNU-5, SNU-16) and non-neoplastic (HFE-145)	2.60	SILAC	23161554
PTK2	gastric cancer	neoplastic (AGS, KatoIII, NCI-N87, SNU-1, SNU-5, SNU-16) and non-neoplastic (HFE-145)	-1.05	SILAC	23161554
PTMA	gastric cancer	neoplastic (AGS, KatoIII, NCI-N87, SNU-1, SNU-5, SNU-16) and non-neoplastic (HFE-145)	2.41	SILAC	23161554
PTPN12	gastric cancer	neoplastic (AGS, KatoIII, NCI-N87, SNU-1, SNU-5, SNU-16) and non-neoplastic (HFE-145)	1.67	SILAC	23161554
PTPRK	gastric cancer	neoplastic (AGS, KatoIII, NCI-N87, SNU-1, SNU-5, SNU-16) and non-neoplastic (HFE-145)	2.87	SILAC	23161554
PUS1	gastric cancer	neoplastic (AGS, KatoIII, NCI-N87, SNU-1, SNU-5, SNU-16) and non-neoplastic (HFE-145)	1.01	SILAC	23161554
PUS7	gastric cancer	neoplastic (AGS, KatoIII, NCI-N87, SNU-1, SNU-5, SNU-16) and non-neoplastic (HFE-145)	2.29	SILAC	23161554
PVR	gastric cancer	neoplastic (AGS, KatoIII, NCI-N87, SNU-1, SNU-5, SNU-16) and non-neoplastic (HFE-145)	3.93	SILAC	23161554
PYCARD	gastric cancer	neoplastic (AGS, KatoIII, NCI-N87, SNU-1, SNU-5, SNU-16) and non-neoplastic (HFE-145)	2.67	SILAC	23161554
PYCRL	gastric cancer	neoplastic (AGS, KatoIII, NCI-N87, SNU-1, SNU-5, SNU-16) and non-neoplastic (HFE-145)	1.66	SILAC	23161554
PYGB	gastric cancer	neoplastic (AGS, KatoIII, NCI-N87, SNU-1, SNU-5, SNU-16) and non-neoplastic (HFE-145)	2.49	SILAC	23161554
PZP	gastric cancer	neoplastic (AGS, KatoIII, NCI-N87, SNU-1, SNU-5, SNU-16) and non-neoplastic (HFE-145)	3.03	SILAC	23161554
QPCT	gastric cancer	neoplastic (AGS, KatoIII, NCI-N87, SNU-1, SNU-5, SNU-16) and non-neoplastic (HFE-145)	4.32	SILAC	23161554
QSOX2	gastric cancer	neoplastic (AGS, KatoIII, NCI-N87, SNU-1, SNU-5, SNU-16) and non-neoplastic (HFE-145)	1.31	SILAC	23161554
RAB10	gastric cancer	neoplastic (AGS, KatoIII, NCI-N87, SNU-1, SNU-5, SNU-16) and non-neoplastic (HFE-145)	1.72	SILAC	23161554
RAB11B	gastric cancer	neoplastic (AGS, KatoIII, NCI-N87, SNU-1, SNU-5, SNU-16) and non-neoplastic (HFE-145)	1.49	SILAC	23161554

RAB14	gastric cancer	neoplastic (AGS, KatoIII, NCI-N87, SNU-1, SNU-5, SNU-16) and non-neoplastic (HFE-145)	1.59	SILAC	23161554
RAB18	gastric cancer	neoplastic (AGS, KatoIII, NCI-N87, SNU-1, SNU-5, SNU-16) and non-neoplastic (HFE-145)	1.67	SILAC	23161554
RAB19	gastric cancer	neoplastic (AGS, KatoIII, NCI-N87, SNU-1, SNU-5, SNU-16) and non-neoplastic (HFE-145)	1.23	SILAC	23161554
RAB1A	gastric cancer	neoplastic (AGS, KatoIII, NCI-N87, SNU-1, SNU-5, SNU-16) and non-neoplastic (HFE-145)	1.68	SILAC	23161554
RAB35	gastric cancer	neoplastic (AGS, KatoIII, NCI-N87, SNU-1, SNU-5, SNU-16) and non-neoplastic (HFE-145)	1.58	SILAC	23161554
RAB5C	gastric cancer	neoplastic (AGS, KatoIII, NCI-N87, SNU-1, SNU-5, SNU-16) and non-neoplastic (HFE-145)	1.40	SILAC	23161554
RAB7A	gastric cancer	neoplastic (AGS, KatoIII, NCI-N87, SNU-1, SNU-5, SNU-16) and non-neoplastic (HFE-145)	1.20	SILAC	23161554
RABGGTA	gastric cancer	neoplastic (AGS, KatoIII, NCI-N87, SNU-1, SNU-5, SNU-16) and non-neoplastic (HFE-145)	1.63	SILAC	23161554
RAD23B	gastric cancer	neoplastic (AGS, KatoIII, NCI-N87, SNU-1, SNU-5, SNU-16) and non-neoplastic (HFE-145)	2.74	SILAC	23161554
RAD50	gastric cancer	neoplastic (AGS, KatoIII, NCI-N87, SNU-1, SNU-5, SNU-16) and non-neoplastic (HFE-145)	1.38	SILAC	23161554
RALA	gastric cancer	neoplastic (AGS, KatoIII, NCI-N87, SNU-1, SNU-5, SNU-16) and non-neoplastic (HFE-145)	2.37	SILAC	23161554
RALY	gastric cancer	neoplastic (AGS, KatoIII, NCI-N87, SNU-1, SNU-5, SNU-16) and non-neoplastic (HFE-145)	2.17	SILAC	23161554
RAN	gastric cancer	neoplastic (AGS, KatoIII, NCI-N87, SNU-1, SNU-5, SNU-16) and non-neoplastic (HFE-145)	1.37	SILAC	23161554
RANBP1	gastric cancer	neoplastic (AGS, KatoIII, NCI-N87, SNU-1, SNU-5, SNU-16) and non-neoplastic (HFE-145)	1.21	SILAC	23161554
RAP1A	gastric cancer	neoplastic (AGS, KatoIII, NCI-N87, SNU-1, SNU-5, SNU-16) and non-neoplastic (HFE-145)	4.21	SILAC	23161554
RBX1	gastric cancer	neoplastic (AGS, KatoIII, NCI-N87, SNU-1, SNU-5, SNU-16) and non-neoplastic (HFE-145)	2.60	SILAC	23161554
RCN1	gastric cancer	neoplastic (AGS, KatoIII, NCI-N87, SNU-1, SNU-5, SNU-16) and non-neoplastic (HFE-145)	1.27	SILAC	23161554
RCN2	gastric cancer	neoplastic (AGS, KatoIII, NCI-N87, SNU-1, SNU-5, SNU-16) and non-neoplastic (HFE-145)	1.16	SILAC	23161554
RHOA	gastric cancer	neoplastic (AGS, KatoIII, NCI-N87, SNU-1, SNU-5, SNU-16) and non-neoplastic (HFE-145)	1.74	SILAC	23161554
RNASE4	gastric cancer	neoplastic (AGS, KatoIII, NCI-N87, SNU-1, SNU-5, SNU-16) and non-neoplastic (HFE-145)	2.19	SILAC	23161554
RNASET2	gastric cancer	neoplastic (AGS, KatoIII, NCI-N87, SNU-1, SNU-5, SNU-16) and non-neoplastic (HFE-145)	2.95	SILAC	23161554
RNPEP	gastric cancer	neoplastic (AGS, KatoIII, NCI-N87, SNU-1, SNU-5, SNU-16) and non-neoplastic (HFE-145)	1.75	SILAC	23161554
RPL10	gastric cancer	neoplastic (AGS, KatoIII, NCI-N87, SNU-1, SNU-5, SNU-16) and non-neoplastic (HFE-145)	2.62	SILAC	23161554
RPL10A	gastric cancer	neoplastic (AGS, KatoIII, NCI-N87, SNU-1, SNU-5, SNU-16) and non-neoplastic (HFE-145)	3.46	SILAC	23161554
RPL11	gastric cancer	neoplastic (AGS, KatoIII, NCI-N87, SNU-1, SNU-5, SNU-16) and non-neoplastic (HFE-145)	1.38	SILAC	23161554
RPL12	gastric cancer	neoplastic (AGS, KatoIII, NCI-N87, SNU-1, SNU-5, SNU-16) and non-neoplastic (HFE-145)	1.73	SILAC	23161554
RPL13	gastric cancer	neoplastic (AGS, KatoIII, NCI-N87, SNU-1, SNU-5, SNU-16) and non-neoplastic (HFE-145)	2.42	SILAC	23161554
RPL13A	gastric cancer	neoplastic (AGS, KatoIII, NCI-N87, SNU-1, SNU-5, SNU-16) and non-neoplastic (HFE-145)	2.00	SILAC	23161554
RPL14	gastric cancer	neoplastic (AGS, KatoIII, NCI-N87, SNU-1, SNU-5, SNU-16) and non-neoplastic (HFE-145)	1.82	SILAC	23161554
RPL15	gastric cancer	neoplastic (AGS, KatoIII, NCI-N87, SNU-1, SNU-5, SNU-16) and non-neoplastic (HFE-145)	2.03	SILAC	23161554
RPL17	gastric cancer	neoplastic (AGS, KatoIII, NCI-N87, SNU-1, SNU-5, SNU-16) and non-neoplastic (HFE-145)	1.59	SILAC	23161554
RPL18	gastric cancer	neoplastic (AGS, KatoIII, NCI-N87, SNU-1, SNU-5, SNU-16) and non-neoplastic (HFE-145)	1.87	SILAC	23161554
RPL18A	gastric cancer	neoplastic (AGS, KatoIII, NCI-N87, SNU-1, SNU-5, SNU-16) and non-neoplastic (HFE-145)	1.90	SILAC	23161554
RPL21	gastric cancer	neoplastic (AGS, KatoIII, NCI-N87, SNU-1, SNU-5, SNU-16) and non-neoplastic (HFE-145)	2.58	SILAC	23161554
RPL23A	gastric cancer	neoplastic (AGS, KatoIII, NCI-N87, SNU-1, SNU-5, SNU-16) and non-neoplastic (HFE-145)	1.96	SILAC	23161554
RPL24	gastric cancer	neoplastic (AGS, KatoIII, NCI-N87, SNU-1, SNU-5, SNU-16) and non-neoplastic (HFE-145)	2.02	SILAC	23161554
RPL26	gastric cancer	neoplastic (AGS, KatoIII, NCI-N87, SNU-1, SNU-5, SNU-16) and non-neoplastic (HFE-145)	1.89	SILAC	23161554
RPL27	gastric cancer	neoplastic (AGS, KatoIII, NCI-N87, SNU-1, SNU-5, SNU-16) and non-neoplastic (HFE-145)	2.36	SILAC	23161554
RPL27A	gastric cancer	neoplastic (AGS, KatoIII, NCI-N87, SNU-1, SNU-5, SNU-16) and non-neoplastic (HFE-145)	1.97	SILAC	23161554
RPL28	gastric cancer	neoplastic (AGS, KatoIII, NCI-N87, SNU-1, SNU-5, SNU-16) and non-neoplastic (HFE-145)	2.18	SILAC	23161554
RPL3	gastric cancer	neoplastic (AGS, KatoIII, NCI-N87, SNU-1, SNU-5, SNU-16) and non-neoplastic (HFE-145)	1.95	SILAC	23161554
RPL30	gastric cancer	neoplastic (AGS, KatoIII, NCI-N87, SNU-1, SNU-5, SNU-16) and non-neoplastic (HFE-145)	1.56	SILAC	23161554
RPL31	gastric cancer	neoplastic (AGS, KatoIII, NCI-N87, SNU-1, SNU-5, SNU-16) and non-neoplastic (HFE-145)	1.84	SILAC	23161554

RPL32	gastric cancer	neoplastic (AGS, KatoIII, NCI-N87, SNU-1, SNU-5, SNU-16) and non-neoplastic (HFE-145)	2.12	SILAC	23161554
RPL35	gastric cancer	neoplastic (AGS, KatoIII, NCI-N87, SNU-1, SNU-5, SNU-16) and non-neoplastic (HFE-145)	2.78	SILAC	23161554
RPL35A	gastric cancer	neoplastic (AGS, KatoIII, NCI-N87, SNU-1, SNU-5, SNU-16) and non-neoplastic (HFE-145)	2.19	SILAC	23161554
RPL38	gastric cancer	neoplastic (AGS, KatoIII, NCI-N87, SNU-1, SNU-5, SNU-16) and non-neoplastic (HFE-145)	2.40	SILAC	23161554
RPL4	gastric cancer	neoplastic (AGS, KatoIII, NCI-N87, SNU-1, SNU-5, SNU-16) and non-neoplastic (HFE-145)	1.89	SILAC	23161554
RPL5	gastric cancer	neoplastic (AGS, KatoIII, NCI-N87, SNU-1, SNU-5, SNU-16) and non-neoplastic (HFE-145)	1.35	SILAC	23161554
RPL6	gastric cancer	neoplastic (AGS, KatoIII, NCI-N87, SNU-1, SNU-5, SNU-16) and non-neoplastic (HFE-145)	1.93	SILAC	23161554
RPL7	gastric cancer	neoplastic (AGS, KatoIII, NCI-N87, SNU-1, SNU-5, SNU-16) and non-neoplastic (HFE-145)	1.89	SILAC	23161554
RPL7A	gastric cancer	neoplastic (AGS, KatoIII, NCI-N87, SNU-1, SNU-5, SNU-16) and non-neoplastic (HFE-145)	2.48	SILAC	23161554
RPL8	gastric cancer	neoplastic (AGS, KatoIII, NCI-N87, SNU-1, SNU-5, SNU-16) and non-neoplastic (HFE-145)	2.06	SILAC	23161554
RPL9	gastric cancer	neoplastic (AGS, KatoIII, NCI-N87, SNU-1, SNU-5, SNU-16) and non-neoplastic (HFE-145)	2.01	SILAC	23161554
RPLP0	gastric cancer	neoplastic (AGS, KatoIII, NCI-N87, SNU-1, SNU-5, SNU-16) and non-neoplastic (HFE-145)	1.64	SILAC	23161554
RPLP1	gastric cancer	neoplastic (AGS, KatoIII, NCI-N87, SNU-1, SNU-5, SNU-16) and non-neoplastic (HFE-145)	2.02	SILAC	23161554
RPLP2	gastric cancer	neoplastic (AGS, KatoIII, NCI-N87, SNU-1, SNU-5, SNU-16) and non-neoplastic (HFE-145)	1.54	SILAC	23161554
RPP14	gastric cancer	neoplastic (AGS, KatoIII, NCI-N87, SNU-1, SNU-5, SNU-16) and non-neoplastic (HFE-145)	1.52	SILAC	23161554
RPP30	gastric cancer	neoplastic (AGS, KatoIII, NCI-N87, SNU-1, SNU-5, SNU-16) and non-neoplastic (HFE-145)	1.65	SILAC	23161554
RPS10	gastric cancer	neoplastic (AGS, KatoIII, NCI-N87, SNU-1, SNU-5, SNU-16) and non-neoplastic (HFE-145)	1.77	SILAC	23161554
RPS11	gastric cancer	neoplastic (AGS, KatoIII, NCI-N87, SNU-1, SNU-5, SNU-16) and non-neoplastic (HFE-145)	2.23	SILAC	23161554
RPS12	gastric cancer	neoplastic (AGS, KatoIII, NCI-N87, SNU-1, SNU-5, SNU-16) and non-neoplastic (HFE-145)	1.68	SILAC	23161554
RPS13	gastric cancer	neoplastic (AGS, KatoIII, NCI-N87, SNU-1, SNU-5, SNU-16) and non-neoplastic (HFE-145)	1.93	SILAC	23161554
RPS14	gastric cancer	neoplastic (AGS, KatoIII, NCI-N87, SNU-1, SNU-5, SNU-16) and non-neoplastic (HFE-145)	2.01	SILAC	23161554
RPS15	gastric cancer	neoplastic (AGS, KatoIII, NCI-N87, SNU-1, SNU-5, SNU-16) and non-neoplastic (HFE-145)	2.62	SILAC	23161554
RPS15A	gastric cancer	neoplastic (AGS, KatoIII, NCI-N87, SNU-1, SNU-5, SNU-16) and non-neoplastic (HFE-145)	1.86	SILAC	23161554
RPS16	gastric cancer	neoplastic (AGS, KatoIII, NCI-N87, SNU-1, SNU-5, SNU-16) and non-neoplastic (HFE-145)	1.58	SILAC	23161554
RPS18	gastric cancer	neoplastic (AGS, KatoIII, NCI-N87, SNU-1, SNU-5, SNU-16) and non-neoplastic (HFE-145)	1.88	SILAC	23161554
RPS19	gastric cancer	neoplastic (AGS, KatoIII, NCI-N87, SNU-1, SNU-5, SNU-16) and non-neoplastic (HFE-145)	2.52	SILAC	23161554
RPS2	gastric cancer	neoplastic (AGS, KatoIII, NCI-N87, SNU-1, SNU-5, SNU-16) and non-neoplastic (HFE-145)	1.82	SILAC	23161554
RPS20	gastric cancer	neoplastic (AGS, KatoIII, NCI-N87, SNU-1, SNU-5, SNU-16) and non-neoplastic (HFE-145)	1.76	SILAC	23161554
RPS21	gastric cancer	neoplastic (AGS, KatoIII, NCI-N87, SNU-1, SNU-5, SNU-16) and non-neoplastic (HFE-145)	1.21	SILAC	23161554
RPS23	gastric cancer	neoplastic (AGS, KatoIII, NCI-N87, SNU-1, SNU-5, SNU-16) and non-neoplastic (HFE-145)	2.02	SILAC	23161554
RPS24	gastric cancer	neoplastic (AGS, KatoIII, NCI-N87, SNU-1, SNU-5, SNU-16) and non-neoplastic (HFE-145)	1.95	SILAC	23161554
RPS25	gastric cancer	neoplastic (AGS, KatoIII, NCI-N87, SNU-1, SNU-5, SNU-16) and non-neoplastic (HFE-145)	1.68	SILAC	23161554
RPS26	gastric cancer	neoplastic (AGS, KatoIII, NCI-N87, SNU-1, SNU-5, SNU-16) and non-neoplastic (HFE-145)	1.76	SILAC	23161554
RPS27	gastric cancer	neoplastic (AGS, KatoIII, NCI-N87, SNU-1, SNU-5, SNU-16) and non-neoplastic (HFE-145)	2.51	SILAC	23161554
RPS27L	gastric cancer	neoplastic (AGS, KatoIII, NCI-N87, SNU-1, SNU-5, SNU-16) and non-neoplastic (HFE-145)	1.80	SILAC	23161554
RPS28	gastric cancer	neoplastic (AGS, KatoIII, NCI-N87, SNU-1, SNU-5, SNU-16) and non-neoplastic (HFE-145)	1.78	SILAC	23161554
RPS3	gastric cancer	neoplastic (AGS, KatoIII, NCI-N87, SNU-1, SNU-5, SNU-16) and non-neoplastic (HFE-145)	2.06	SILAC	23161554
RPS3A	gastric cancer	neoplastic (AGS, KatoIII, NCI-N87, SNU-1, SNU-5, SNU-16) and non-neoplastic (HFE-145)	2.19	SILAC	23161554
RPS4X	gastric cancer	neoplastic (AGS, KatoIII, NCI-N87, SNU-1, SNU-5, SNU-16) and non-neoplastic (HFE-145)	1.86	SILAC	23161554
RPS5	gastric cancer	neoplastic (AGS, KatoIII, NCI-N87, SNU-1, SNU-5, SNU-16) and non-neoplastic (HFE-145)	1.40	SILAC	23161554
RPS6	gastric cancer	neoplastic (AGS, KatoIII, NCI-N87, SNU-1, SNU-5, SNU-16) and non-neoplastic (HFE-145)	1.04	SILAC	23161554
RPS7	gastric cancer	neoplastic (AGS, KatoIII, NCI-N87, SNU-1, SNU-5, SNU-16) and non-neoplastic (HFE-145)	2.41	SILAC	23161554
RPS8	gastric cancer	neoplastic (AGS, KatoIII, NCI-N87, SNU-1, SNU-5, SNU-16) and non-neoplastic (HFE-145)	1.81	SILAC	23161554

RPS9	gastric cancer	neoplastic (AGS, KatoIII, NCI-N87, SNU-1, SNU-5, SNU-16) and non-neoplastic (HFE-145)	2.33	SILAC	23161554
RPSA	gastric cancer	neoplastic (AGS, KatoIII, NCI-N87, SNU-1, SNU-5, SNU-16) and non-neoplastic (HFE-145)	1.33	SILAC	23161554
RPUSD2	gastric cancer	neoplastic (AGS, KatoIII, NCI-N87, SNU-1, SNU-5, SNU-16) and non-neoplastic (HFE-145)	1.21	SILAC	23161554
RRBP1	gastric cancer	neoplastic (AGS, KatoIII, NCI-N87, SNU-1, SNU-5, SNU-16) and non-neoplastic (HFE-145)	2.13	SILAC	23161554
RTCD1	gastric cancer	neoplastic (AGS, KatoIII, NCI-N87, SNU-1, SNU-5, SNU-16) and non-neoplastic (HFE-145)	1.37	SILAC	23161554
RTN3	gastric cancer	neoplastic (AGS, KatoIII, NCI-N87, SNU-1, SNU-5, SNU-16) and non-neoplastic (HFE-145)	1.42	SILAC	23161554
RTN4	gastric cancer	neoplastic (AGS, KatoIII, NCI-N87, SNU-1, SNU-5, SNU-16) and non-neoplastic (HFE-145)	2.04	SILAC	23161554
RUVBL1	gastric cancer	neoplastic (AGS, KatoIII, NCI-N87, SNU-1, SNU-5, SNU-16) and non-neoplastic (HFE-145)	1.30	SILAC	23161554
RUVBL2	gastric cancer	neoplastic (AGS, KatoIII, NCI-N87, SNU-1, SNU-5, SNU-16) and non-neoplastic (HFE-145)	1.11	SILAC	23161554
S100A11	gastric cancer	neoplastic (AGS, KatoIII, NCI-N87, SNU-1, SNU-5, SNU-16) and non-neoplastic (HFE-145)	3.66	SILAC	23161554
S100A16	gastric cancer	neoplastic (AGS, KatoIII, NCI-N87, SNU-1, SNU-5, SNU-16) and non-neoplastic (HFE-145)	1.57	SILAC	23161554
S100A6	gastric cancer	neoplastic (AGS, KatoIII, NCI-N87, SNU-1, SNU-5, SNU-16) and non-neoplastic (HFE-145)	3.14	SILAC	23161554
SAMD9	gastric cancer	neoplastic (AGS, KatoIII, NCI-N87, SNU-1, SNU-5, SNU-16) and non-neoplastic (HFE-145)	1.49	SILAC	23161554
SARNP	gastric cancer	neoplastic (AGS, KatoIII, NCI-N87, SNU-1, SNU-5, SNU-16) and non-neoplastic (HFE-145)	1.98	SILAC	23161554
SARS	gastric cancer	neoplastic (AGS, KatoIII, NCI-N87, SNU-1, SNU-5, SNU-16) and non-neoplastic (HFE-145)	2.14	SILAC	23161554
SART1	gastric cancer	neoplastic (AGS, KatoIII, NCI-N87, SNU-1, SNU-5, SNU-16) and non-neoplastic (HFE-145)	2.14	SILAC	23161554
SART3	gastric cancer	neoplastic (AGS, KatoIII, NCI-N87, SNU-1, SNU-5, SNU-16) and non-neoplastic (HFE-145)	1.34	SILAC	23161554
SBDS	gastric cancer	neoplastic (AGS, KatoIII, NCI-N87, SNU-1, SNU-5, SNU-16) and non-neoplastic (HFE-145)	1.33	SILAC	23161554
SCPEP1	gastric cancer	neoplastic (AGS, KatoIII, NCI-N87, SNU-1, SNU-5, SNU-16) and non-neoplastic (HFE-145)	1.25	SILAC	23161554
SDC1	gastric cancer	neoplastic (AGS, KatoIII, NCI-N87, SNU-1, SNU-5, SNU-16) and non-neoplastic (HFE-145)	3.21	SILAC	23161554
SDC4	gastric cancer	neoplastic (AGS, KatoIII, NCI-N87, SNU-1, SNU-5, SNU-16) and non-neoplastic (HFE-145)	2.59	SILAC	23161554
SDCBP	gastric cancer	neoplastic (AGS, KatoIII, NCI-N87, SNU-1, SNU-5, SNU-16) and non-neoplastic (HFE-145)	1.41	SILAC	23161554
SDF4	gastric cancer	neoplastic (AGS, KatoIII, NCI-N87, SNU-1, SNU-5, SNU-16) and non-neoplastic (HFE-145)	2.10	SILAC	23161554
SEC23IP	gastric cancer	neoplastic (AGS, KatoIII, NCI-N87, SNU-1, SNU-5, SNU-16) and non-neoplastic (HFE-145)	1.23	SILAC	23161554
SEC31A	gastric cancer	neoplastic (AGS, KatoIII, NCI-N87, SNU-1, SNU-5, SNU-16) and non-neoplastic (HFE-145)	1.36	SILAC	23161554
SEMA7A	gastric cancer	neoplastic (AGS, KatoIII, NCI-N87, SNU-1, SNU-5, SNU-16) and non-neoplastic (HFE-145)	-2.73	SILAC	23161554
SERPINA1	gastric cancer	neoplastic (AGS, KatoIII, NCI-N87, SNU-1, SNU-5, SNU-16) and non-neoplastic (HFE-145)	3.14	SILAC	23161554
SERPINB1	gastric cancer	neoplastic (AGS, KatoIII, NCI-N87, SNU-1, SNU-5, SNU-16) and non-neoplastic (HFE-145)	5.17	SILAC	23161554
SERPINB2	gastric cancer	neoplastic (AGS, KatoIII, NCI-N87, SNU-1, SNU-5, SNU-16) and non-neoplastic (HFE-145)	-1.88	SILAC	23161554
SERPINB6	gastric cancer	neoplastic (AGS, KatoIII, NCI-N87, SNU-1, SNU-5, SNU-16) and non-neoplastic (HFE-145)	1.20	SILAC	23161554
SERPINB9	gastric cancer	neoplastic (AGS, KatoIII, NCI-N87, SNU-1, SNU-5, SNU-16) and non-neoplastic (HFE-145)	1.42	SILAC	23161554
SERPINC1	gastric cancer	neoplastic (AGS, KatoIII, NCI-N87, SNU-1, SNU-5, SNU-16) and non-neoplastic (HFE-145)	3.65	SILAC	23161554
SERPINE1	gastric cancer	neoplastic (AGS, KatoIII, NCI-N87, SNU-1, SNU-5, SNU-16) and non-neoplastic (HFE-145)	-4.64	SILAC	23161554
SERPINE2	gastric cancer	neoplastic (AGS, KatoIII, NCI-N87, SNU-1, SNU-5, SNU-16) and non-neoplastic (HFE-145)	-2.94	SILAC	23161554
SET	gastric cancer	neoplastic (AGS, KatoIII, NCI-N87, SNU-1, SNU-5, SNU-16) and non-neoplastic (HFE-145)	1.12	SILAC	23161554
SF3A1	gastric cancer	neoplastic (AGS, KatoIII, NCI-N87, SNU-1, SNU-5, SNU-16) and non-neoplastic (HFE-145)	1.42	SILAC	23161554
SF3B1	gastric cancer	neoplastic (AGS, KatoIII, NCI-N87, SNU-1, SNU-5, SNU-16) and non-neoplastic (HFE-145)	1.63	SILAC	23161554
SF3B14	gastric cancer	neoplastic (AGS, KatoIII, NCI-N87, SNU-1, SNU-5, SNU-16) and non-neoplastic (HFE-145)	1.33	SILAC	23161554
SF3B3	gastric cancer	neoplastic (AGS, KatoIII, NCI-N87, SNU-1, SNU-5, SNU-16) and non-neoplastic (HFE-145)	1.01	SILAC	23161554
SH3BGRL2	gastric cancer	neoplastic (AGS, KatoIII, NCI-N87, SNU-1, SNU-5, SNU-16) and non-neoplastic (HFE-145)	2.92	SILAC	23161554
SHMT1	gastric cancer	neoplastic (AGS, KatoIII, NCI-N87, SNU-1, SNU-5, SNU-16) and non-neoplastic (HFE-145)	-1.05	SILAC	23161554
SKIV2L	gastric cancer	neoplastic (AGS, KatoIII, NCI-N87, SNU-1, SNU-5, SNU-16) and non-neoplastic (HFE-145)	1.50	SILAC	23161554
SLC1A5	gastric cancer	neoplastic (AGS, KatoIII, NCI-N87, SNU-1, SNU-5, SNU-16) and non-neoplastic (HFE-145)	1.74	SILAC	23161554

SLC3A2	gastric cancer	neoplastic (AGS, KatoIII, NCI-N87, SNU-1, SNU-5, SNU-16) and non-neoplastic (HFE-145)	3.00	SILAC	23161554
SLC9A3R1	gastric cancer	neoplastic (AGS, KatoIII, NCI-N87, SNU-1, SNU-5, SNU-16) and non-neoplastic (HFE-145)	1.81	SILAC	23161554
SMC3	gastric cancer	neoplastic (AGS, KatoIII, NCI-N87, SNU-1, SNU-5, SNU-16) and non-neoplastic (HFE-145)	1.23	SILAC	23161554
SNRNP70	gastric cancer	neoplastic (AGS, KatoIII, NCI-N87, SNU-1, SNU-5, SNU-16) and non-neoplastic (HFE-145)	1.38	SILAC	23161554
SNRPB	gastric cancer	neoplastic (AGS, KatoIII, NCI-N87, SNU-1, SNU-5, SNU-16) and non-neoplastic (HFE-145)	1.91	SILAC	23161554
SNRPD1	gastric cancer	neoplastic (AGS, KatoIII, NCI-N87, SNU-1, SNU-5, SNU-16) and non-neoplastic (HFE-145)	1.50	SILAC	23161554
SNRPD2	gastric cancer	neoplastic (AGS, KatoIII, NCI-N87, SNU-1, SNU-5, SNU-16) and non-neoplastic (HFE-145)	1.29	SILAC	23161554
SNRPD3	gastric cancer	neoplastic (AGS, KatoIII, NCI-N87, SNU-1, SNU-5, SNU-16) and non-neoplastic (HFE-145)	1.42	SILAC	23161554
SNRPE	gastric cancer	neoplastic (AGS, KatoIII, NCI-N87, SNU-1, SNU-5, SNU-16) and non-neoplastic (HFE-145)	1.45	SILAC	23161554
SNRPF	gastric cancer	neoplastic (AGS, KatoIII, NCI-N87, SNU-1, SNU-5, SNU-16) and non-neoplastic (HFE-145)	1.97	SILAC	23161554
SNX3	gastric cancer	neoplastic (AGS, KatoIII, NCI-N87, SNU-1, SNU-5, SNU-16) and non-neoplastic (HFE-145)	1.29	SILAC	23161554
SOD1	gastric cancer	neoplastic (AGS, KatoIII, NCI-N87, SNU-1, SNU-5, SNU-16) and non-neoplastic (HFE-145)	1.10	SILAC	23161554
SOD2	gastric cancer	neoplastic (AGS, KatoIII, NCI-N87, SNU-1, SNU-5, SNU-16) and non-neoplastic (HFE-145)	-1.43	SILAC	23161554
SORD	gastric cancer	neoplastic (AGS, KatoIII, NCI-N87, SNU-1, SNU-5, SNU-16) and non-neoplastic (HFE-145)	2.92	SILAC	23161554
SPARC	gastric cancer	neoplastic (AGS, KatoIII, NCI-N87, SNU-1, SNU-5, SNU-16) and non-neoplastic (HFE-145)	-4.32	SILAC	23161554
SPR	gastric cancer	neoplastic (AGS, KatoIII, NCI-N87, SNU-1, SNU-5, SNU-16) and non-neoplastic (HFE-145)	1.58	SILAC	23161554
SPTAN1	gastric cancer	neoplastic (AGS, KatoIII, NCI-N87, SNU-1, SNU-5, SNU-16) and non-neoplastic (HFE-145)	2.04	SILAC	23161554
SPTBN1	gastric cancer	neoplastic (AGS, KatoIII, NCI-N87, SNU-1, SNU-5, SNU-16) and non-neoplastic (HFE-145)	1.95	SILAC	23161554
SRI	gastric cancer	neoplastic (AGS, KatoIII, NCI-N87, SNU-1, SNU-5, SNU-16) and non-neoplastic (HFE-145)	2.06	SILAC	23161554
SRP14	gastric cancer	neoplastic (AGS, KatoIII, NCI-N87, SNU-1, SNU-5, SNU-16) and non-neoplastic (HFE-145)	1.40	SILAC	23161554
SRSF1	gastric cancer	neoplastic (AGS, KatoIII, NCI-N87, SNU-1, SNU-5, SNU-16) and non-neoplastic (HFE-145)	2.01	SILAC	23161554
SRSF10	gastric cancer	neoplastic (AGS, KatoIII, NCI-N87, SNU-1, SNU-5, SNU-16) and non-neoplastic (HFE-145)	2.48	SILAC	23161554
SRSF2	gastric cancer	neoplastic (AGS, KatoIII, NCI-N87, SNU-1, SNU-5, SNU-16) and non-neoplastic (HFE-145)	2.70	SILAC	23161554
SRSF3	gastric cancer	neoplastic (AGS, KatoIII, NCI-N87, SNU-1, SNU-5, SNU-16) and non-neoplastic (HFE-145)	1.65	SILAC	23161554
SRSF6	gastric cancer	neoplastic (AGS, KatoIII, NCI-N87, SNU-1, SNU-5, SNU-16) and non-neoplastic (HFE-145)	2.80	SILAC	23161554
SRSF7	gastric cancer	neoplastic (AGS, KatoIII, NCI-N87, SNU-1, SNU-5, SNU-16) and non-neoplastic (HFE-145)	2.22	SILAC	23161554
SRSF9	gastric cancer	neoplastic (AGS, KatoIII, NCI-N87, SNU-1, SNU-5, SNU-16) and non-neoplastic (HFE-145)	2.71	SILAC	23161554
SSB	gastric cancer	neoplastic (AGS, KatoIII, NCI-N87, SNU-1, SNU-5, SNU-16) and non-neoplastic (HFE-145)	1.96	SILAC	23161554
SSSCA1	gastric cancer	neoplastic (AGS, KatoIII, NCI-N87, SNU-1, SNU-5, SNU-16) and non-neoplastic (HFE-145)	1.70	SILAC	23161554
SSU72	gastric cancer	neoplastic (AGS, KatoIII, NCI-N87, SNU-1, SNU-5, SNU-16) and non-neoplastic (HFE-145)	1.11	SILAC	23161554
ST13	gastric cancer	neoplastic (AGS, KatoIII, NCI-N87, SNU-1, SNU-5, SNU-16) and non-neoplastic (HFE-145)	1.25	SILAC	23161554
STAT3	gastric cancer	neoplastic (AGS, KatoIII, NCI-N87, SNU-1, SNU-5, SNU-16) and non-neoplastic (HFE-145)	1.95	SILAC	23161554
STX7	gastric cancer	neoplastic (AGS, KatoIII, NCI-N87, SNU-1, SNU-5, SNU-16) and non-neoplastic (HFE-145)	-2.55	SILAC	23161554
SUB1	gastric cancer	neoplastic (AGS, KatoIII, NCI-N87, SNU-1, SNU-5, SNU-16) and non-neoplastic (HFE-145)	1.65	SILAC	23161554
TAF15	gastric cancer	neoplastic (AGS, KatoIII, NCI-N87, SNU-1, SNU-5, SNU-16) and non-neoplastic (HFE-145)	1.94	SILAC	23161554
TAGLN	gastric cancer	neoplastic (AGS, KatoIII, NCI-N87, SNU-1, SNU-5, SNU-16) and non-neoplastic (HFE-145)	-1.12	SILAC	23161554
TARS	gastric cancer	neoplastic (AGS, KatoIII, NCI-N87, SNU-1, SNU-5, SNU-16) and non-neoplastic (HFE-145)	1.01	SILAC	23161554
TAX1BP1	gastric cancer	neoplastic (AGS, KatoIII, NCI-N87, SNU-1, SNU-5, SNU-16) and non-neoplastic (HFE-145)	2.45	SILAC	23161554
TBC1D13	gastric cancer	neoplastic (AGS, KatoIII, NCI-N87, SNU-1, SNU-5, SNU-16) and non-neoplastic (HFE-145)	-1.15	SILAC	23161554
TBC1D9B	gastric cancer	neoplastic (AGS, KatoIII, NCI-N87, SNU-1, SNU-5, SNU-16) and non-neoplastic (HFE-145)	1.07	SILAC	23161554
TBL1XR1	gastric cancer	neoplastic (AGS, KatoIII, NCI-N87, SNU-1, SNU-5, SNU-16) and non-neoplastic (HFE-145)	3.19	SILAC	23161554
TBPL1	gastric cancer	neoplastic (AGS, KatoIII, NCI-N87, SNU-1, SNU-5, SNU-16) and non-neoplastic (HFE-145)	1.69	SILAC	23161554
TFAM	gastric cancer	neoplastic (AGS, KatoIII, NCI-N87, SNU-1, SNU-5, SNU-16) and non-neoplastic (HFE-145)	-2.25	SILAC	23161554

TFRC	gastric cancer	neoplastic (AGS, KatoIII, NCI-N87, SNU-1, SNU-5, SNU-16) and non-neoplastic (HFE-145)	3.82	SILAC	23161554
TGFB1	gastric cancer	neoplastic (AGS, KatoIII, NCI-N87, SNU-1, SNU-5, SNU-16) and non-neoplastic (HFE-145)	-1.39	SILAC	23161554
TGFB2	gastric cancer	neoplastic (AGS, KatoIII, NCI-N87, SNU-1, SNU-5, SNU-16) and non-neoplastic (HFE-145)	3.80	SILAC	23161554
THBS1	gastric cancer	neoplastic (AGS, KatoIII, NCI-N87, SNU-1, SNU-5, SNU-16) and non-neoplastic (HFE-145)	-1.83	SILAC	23161554
THBS2	gastric cancer	neoplastic (AGS, KatoIII, NCI-N87, SNU-1, SNU-5, SNU-16) and non-neoplastic (HFE-145)	-2.05	SILAC	23161554
TIMP1	gastric cancer	neoplastic (AGS, KatoIII, NCI-N87, SNU-1, SNU-5, SNU-16) and non-neoplastic (HFE-145)	2.12	SILAC	23161554
TIMP2	gastric cancer	neoplastic (AGS, KatoIII, NCI-N87, SNU-1, SNU-5, SNU-16) and non-neoplastic (HFE-145)	-2.05	SILAC	23161554
TINAGL1	gastric cancer	neoplastic (AGS, KatoIII, NCI-N87, SNU-1, SNU-5, SNU-16) and non-neoplastic (HFE-145)	2.48	SILAC	23161554
TJP1	gastric cancer	neoplastic (AGS, KatoIII, NCI-N87, SNU-1, SNU-5, SNU-16) and non-neoplastic (HFE-145)	3.26	SILAC	23161554
TKT	gastric cancer	neoplastic (AGS, KatoIII, NCI-N87, SNU-1, SNU-5, SNU-16) and non-neoplastic (HFE-145)	1.13	SILAC	23161554
TMED10	gastric cancer	neoplastic (AGS, KatoIII, NCI-N87, SNU-1, SNU-5, SNU-16) and non-neoplastic (HFE-145)	1.84	SILAC	23161554
TMOD3	gastric cancer	neoplastic (AGS, KatoIII, NCI-N87, SNU-1, SNU-5, SNU-16) and non-neoplastic (HFE-145)	4.84	SILAC	23161554
TMSB4X	gastric cancer	neoplastic (AGS, KatoIII, NCI-N87, SNU-1, SNU-5, SNU-16) and non-neoplastic (HFE-145)	1.93	SILAC	23161554
TNC	gastric cancer	neoplastic (AGS, KatoIII, NCI-N87, SNU-1, SNU-5, SNU-16) and non-neoplastic (HFE-145)	-2.18	SILAC	23161554
TNFRSF12A	gastric cancer	neoplastic (AGS, KatoIII, NCI-N87, SNU-1, SNU-5, SNU-16) and non-neoplastic (HFE-145)	2.06	SILAC	23161554
TOLLIP	gastric cancer	neoplastic (AGS, KatoIII, NCI-N87, SNU-1, SNU-5, SNU-16) and non-neoplastic (HFE-145)	3.47	SILAC	23161554
TOP1MT	gastric cancer	neoplastic (AGS, KatoIII, NCI-N87, SNU-1, SNU-5, SNU-16) and non-neoplastic (HFE-145)	-1.32	SILAC	23161554
TPM3	gastric cancer	neoplastic (AGS, KatoIII, NCI-N87, SNU-1, SNU-5, SNU-16) and non-neoplastic (HFE-145)	1.36	SILAC	23161554
TPMT	gastric cancer	neoplastic (AGS, KatoIII, NCI-N87, SNU-1, SNU-5, SNU-16) and non-neoplastic (HFE-145)	1.84	SILAC	23161554
TPP1	gastric cancer	neoplastic (AGS, KatoIII, NCI-N87, SNU-1, SNU-5, SNU-16) and non-neoplastic (HFE-145)	-1	SILAC	23161554
TPP2	gastric cancer	neoplastic (AGS, KatoIII, NCI-N87, SNU-1, SNU-5, SNU-16) and non-neoplastic (HFE-145)	1.43	SILAC	23161554
TRA2A	gastric cancer	neoplastic (AGS, KatoIII, NCI-N87, SNU-1, SNU-5, SNU-16) and non-neoplastic (HFE-145)	1.90	SILAC	23161554
TRA2B	gastric cancer	neoplastic (AGS, KatoIII, NCI-N87, SNU-1, SNU-5, SNU-16) and non-neoplastic (HFE-145)	1.33	SILAC	23161554
TRHDE	gastric cancer	neoplastic (AGS, KatoIII, NCI-N87, SNU-1, SNU-5, SNU-16) and non-neoplastic (HFE-145)	-3.47	SILAC	23161554
TRIM28	gastric cancer	neoplastic (AGS, KatoIII, NCI-N87, SNU-1, SNU-5, SNU-16) and non-neoplastic (HFE-145)	1.66	SILAC	23161554
TRMT112	gastric cancer	neoplastic (AGS, KatoIII, NCI-N87, SNU-1, SNU-5, SNU-16) and non-neoplastic (HFE-145)	1.11	SILAC	23161554
TROVE2	gastric cancer	neoplastic (AGS, KatoIII, NCI-N87, SNU-1, SNU-5, SNU-16) and non-neoplastic (HFE-145)	1.56	SILAC	23161554
TSN	gastric cancer	neoplastic (AGS, KatoIII, NCI-N87, SNU-1, SNU-5, SNU-16) and non-neoplastic (HFE-145)	1.04	SILAC	23161554
TSTA3	gastric cancer	neoplastic (AGS, KatoIII, NCI-N87, SNU-1, SNU-5, SNU-16) and non-neoplastic (HFE-145)	1.40	SILAC	23161554
TTC27	gastric cancer	neoplastic (AGS, KatoIII, NCI-N87, SNU-1, SNU-5, SNU-16) and non-neoplastic (HFE-145)	1.27	SILAC	23161554
TTC37	gastric cancer	neoplastic (AGS, KatoIII, NCI-N87, SNU-1, SNU-5, SNU-16) and non-neoplastic (HFE-145)	1.87	SILAC	23161554
TTLL12	gastric cancer	neoplastic (AGS, KatoIII, NCI-N87, SNU-1, SNU-5, SNU-16) and non-neoplastic (HFE-145)	3.49	SILAC	23161554
TUBA1A	gastric cancer	neoplastic (AGS, KatoIII, NCI-N87, SNU-1, SNU-5, SNU-16) and non-neoplastic (HFE-145)	-2.32	SILAC	23161554
TUBA1C	gastric cancer	neoplastic (AGS, KatoIII, NCI-N87, SNU-1, SNU-5, SNU-16) and non-neoplastic (HFE-145)	1.35	SILAC	23161554
TUBB2A	gastric cancer	neoplastic (AGS, KatoIII, NCI-N87, SNU-1, SNU-5, SNU-16) and non-neoplastic (HFE-145)	2.09	SILAC	23161554
TUBB2C	gastric cancer	neoplastic (AGS, KatoIII, NCI-N87, SNU-1, SNU-5, SNU-16) and non-neoplastic (HFE-145)	1.37	SILAC	23161554
TUBB3	gastric cancer	neoplastic (AGS, KatoIII, NCI-N87, SNU-1, SNU-5, SNU-16) and non-neoplastic (HFE-145)	1	SILAC	23161554
TWSG1	gastric cancer	neoplastic (AGS, KatoIII, NCI-N87, SNU-1, SNU-5, SNU-16) and non-neoplastic (HFE-145)	2.83	SILAC	23161554
TXN	gastric cancer	neoplastic (AGS, KatoIII, NCI-N87, SNU-1, SNU-5, SNU-16) and non-neoplastic (HFE-145)	1.36	SILAC	23161554
TXNDC12	gastric cancer	neoplastic (AGS, KatoIII, NCI-N87, SNU-1, SNU-5, SNU-16) and non-neoplastic (HFE-145)	2.16	SILAC	23161554
TXNDC5	gastric cancer	neoplastic (AGS, KatoIII, NCI-N87, SNU-1, SNU-5, SNU-16) and non-neoplastic (HFE-145)	1.89	SILAC	23161554
UAP1	gastric cancer	neoplastic (AGS, KatoIII, NCI-N87, SNU-1, SNU-5, SNU-16) and non-neoplastic (HFE-145)	-2.47	SILAC	23161554
UBE2C	gastric cancer	neoplastic (AGS, KatoIII, NCI-N87, SNU-1, SNU-5, SNU-16) and non-neoplastic (HFE-145)	1.13	SILAC	23161554

UBE3A	gastric cancer	neoplastic (AGS, KatoIII, NCI-N87, SNU-1, SNU-5, SNU-16) and non-neoplastic (HFE-145)	1.16	SILAC	23161554
UBE4A	gastric cancer	neoplastic (AGS, KatoIII, NCI-N87, SNU-1, SNU-5, SNU-16) and non-neoplastic (HFE-145)	1.31	SILAC	23161554
UCHL1	gastric cancer	neoplastic (AGS, KatoIII, NCI-N87, SNU-1, SNU-5, SNU-16) and non-neoplastic (HFE-145)	-3.83	SILAC	23161554
UGDH	gastric cancer	neoplastic (AGS, KatoIII, NCI-N87, SNU-1, SNU-5, SNU-16) and non-neoplastic (HFE-145)	1.92	SILAC	23161554
UGP2	gastric cancer	neoplastic (AGS, KatoIII, NCI-N87, SNU-1, SNU-5, SNU-16) and non-neoplastic (HFE-145)	1.13	SILAC	23161554
ULBP2	gastric cancer	neoplastic (AGS, KatoIII, NCI-N87, SNU-1, SNU-5, SNU-16) and non-neoplastic (HFE-145)	2.12	SILAC	23161554
UPF1	gastric cancer	neoplastic (AGS, KatoIII, NCI-N87, SNU-1, SNU-5, SNU-16) and non-neoplastic (HFE-145)	2.06	SILAC	23161554
UPP1	gastric cancer	neoplastic (AGS, KatoIII, NCI-N87, SNU-1, SNU-5, SNU-16) and non-neoplastic (HFE-145)	1.78	SILAC	23161554
USP39	gastric cancer	neoplastic (AGS, KatoIII, NCI-N87, SNU-1, SNU-5, SNU-16) and non-neoplastic (HFE-145)	2.05	SILAC	23161554
USP9X	gastric cancer	neoplastic (AGS, KatoIII, NCI-N87, SNU-1, SNU-5, SNU-16) and non-neoplastic (HFE-145)	1.59	SILAC	23161554
VARS	gastric cancer	neoplastic (AGS, KatoIII, NCI-N87, SNU-1, SNU-5, SNU-16) and non-neoplastic (HFE-145)	1.18	SILAC	23161554
VCP	gastric cancer	neoplastic (AGS, KatoIII, NCI-N87, SNU-1, SNU-5, SNU-16) and non-neoplastic (HFE-145)	1.06	SILAC	23161554
VIL1	gastric cancer	neoplastic (AGS, KatoIII, NCI-N87, SNU-1, SNU-5, SNU-16) and non-neoplastic (HFE-145)	2.63	SILAC	23161554
VIM	gastric cancer	neoplastic (AGS, KatoIII, NCI-N87, SNU-1, SNU-5, SNU-16) and non-neoplastic (HFE-145)	-4.05	SILAC	23161554
VPS26A	gastric cancer	neoplastic (AGS, KatoIII, NCI-N87, SNU-1, SNU-5, SNU-16) and non-neoplastic (HFE-145)	1.00	SILAC	23161554
VPS28	gastric cancer	neoplastic (AGS, KatoIII, NCI-N87, SNU-1, SNU-5, SNU-16) and non-neoplastic (HFE-145)	2.46	SILAC	23161554
VPS29	gastric cancer	neoplastic (AGS, KatoIII, NCI-N87, SNU-1, SNU-5, SNU-16) and non-neoplastic (HFE-145)	1.02	SILAC	23161554
VPS35	gastric cancer	neoplastic (AGS, KatoIII, NCI-N87, SNU-1, SNU-5, SNU-16) and non-neoplastic (HFE-145)	1.23	SILAC	23161554
WARS	gastric cancer	neoplastic (AGS, KatoIII, NCI-N87, SNU-1, SNU-5, SNU-16) and non-neoplastic (HFE-145)	1.74	SILAC	23161554
WDR11	gastric cancer	neoplastic (AGS, KatoIII, NCI-N87, SNU-1, SNU-5, SNU-16) and non-neoplastic (HFE-145)	2.58	SILAC	23161554
WDR12	gastric cancer	neoplastic (AGS, KatoIII, NCI-N87, SNU-1, SNU-5, SNU-16) and non-neoplastic (HFE-145)	1.61	SILAC	23161554
WDR13	gastric cancer	neoplastic (AGS, KatoIII, NCI-N87, SNU-1, SNU-5, SNU-16) and non-neoplastic (HFE-145)	1.41	SILAC	23161554
WDR61	gastric cancer	neoplastic (AGS, KatoIII, NCI-N87, SNU-1, SNU-5, SNU-16) and non-neoplastic (HFE-145)	1.82	SILAC	23161554
WDR77	gastric cancer	neoplastic (AGS, KatoIII, NCI-N87, SNU-1, SNU-5, SNU-16) and non-neoplastic (HFE-145)	2.23	SILAC	23161554
XPNPEP1	gastric cancer	neoplastic (AGS, KatoIII, NCI-N87, SNU-1, SNU-5, SNU-16) and non-neoplastic (HFE-145)	1.43	SILAC	23161554
XPO5	gastric cancer	neoplastic (AGS, KatoIII, NCI-N87, SNU-1, SNU-5, SNU-16) and non-neoplastic (HFE-145)	1.37	SILAC	23161554
XRCC5	gastric cancer	neoplastic (AGS, KatoIII, NCI-N87, SNU-1, SNU-5, SNU-16) and non-neoplastic (HFE-145)	1.28	SILAC	23161554
XRCC6	gastric cancer	neoplastic (AGS, KatoIII, NCI-N87, SNU-1, SNU-5, SNU-16) and non-neoplastic (HFE-145)	1.02	SILAC	23161554
YARS	gastric cancer	neoplastic (AGS, KatoIII, NCI-N87, SNU-1, SNU-5, SNU-16) and non-neoplastic (HFE-145)	1.22	SILAC	23161554
YWHAB	gastric cancer	neoplastic (AGS, KatoIII, NCI-N87, SNU-1, SNU-5, SNU-16) and non-neoplastic (HFE-145)	2.09	SILAC	23161554
YWHAG	gastric cancer	neoplastic (AGS, KatoIII, NCI-N87, SNU-1, SNU-5, SNU-16) and non-neoplastic (HFE-145)	1.36	SILAC	23161554
YWHAH	gastric cancer	neoplastic (AGS, KatoIII, NCI-N87, SNU-1, SNU-5, SNU-16) and non-neoplastic (HFE-145)	1.27	SILAC	23161554
YWHAQ	gastric cancer	neoplastic (AGS, KatoIII, NCI-N87, SNU-1, SNU-5, SNU-16) and non-neoplastic (HFE-145)	1.56	SILAC	23161554
YWHAZ	gastric cancer	neoplastic (AGS, KatoIII, NCI-N87, SNU-1, SNU-5, SNU-16) and non-neoplastic (HFE-145)	2.15	SILAC	23161554
ZBTB22	gastric cancer	neoplastic (AGS, KatoIII, NCI-N87, SNU-1, SNU-5, SNU-16) and non-neoplastic (HFE-145)	2.23	SILAC	23161554
ZNF207	gastric cancer	neoplastic (AGS, KatoIII, NCI-N87, SNU-1, SNU-5, SNU-16) and non-neoplastic (HFE-145)	1.57	SILAC	23161554
ZNF426	gastric cancer	neoplastic (AGS, KatoIII, NCI-N87, SNU-1, SNU-5, SNU-16) and non-neoplastic (HFE-145)	1.76	SILAC	23161554
ZRANB2	gastric cancer	neoplastic (AGS, KatoIII, NCI-N87, SNU-1, SNU-5, SNU-16) and non-neoplastic (HFE-145)	1.57	SILAC	23161554
ZW10	gastric cancer	neoplastic (AGS, KatoIII, NCI-N87, SNU-1, SNU-5, SNU-16) and non-neoplastic (HFE-145)	1.13	SILAC	23161554
ACTR2	colorectal cancer metastasis	neoplastic (KM12SM, KM12C)	-1	SILAC	23443137
AGRN	colorectal cancer metastasis	neoplastic (KM12SM, KM12C)	-1.59	SILAC	23443137
ARPC4	colorectal cancer metastasis	neoplastic (KM12SM, KM12C)	-1	SILAC	23443137
ATP1B1	colorectal cancer metastasis	neoplastic (KM12SM, KM12C)	-1	SILAC	23443137

ATP5A1	colorectal cancer metastasis	neoplastic (KM12SM, KM12C)	-1.51	SILAC	23443137
ATP5D	colorectal cancer metastasis	neoplastic (KM12SM, KM12C)	-1.18	SILAC	23443137
C19orf10	colorectal cancer metastasis	neoplastic (KM12SM, KM12C)	-2.18	SILAC	23443137
CD137L/TNFS	colorectal cancer metastasis	neoplastic (KM12SM, KM12C)	4.79	SILAC	23443137
CD81	colorectal cancer metastasis	neoplastic (KM12SM, KM12C)	-2.55	SILAC	23443137
CEACAM5	colorectal cancer metastasis	neoplastic (KM12SM, KM12C)	3.06	SILAC	23443137
CHID1	colorectal cancer metastasis	neoplastic (KM12SM, KM12C)	-2.47	SILAC	23443137
COX4I1	colorectal cancer metastasis	neoplastic (KM12SM, KM12C)	-1.21	SILAC	23443137
COX5A	colorectal cancer metastasis	neoplastic (KM12SM, KM12C)	-1.02	SILAC	23443137
CTSD	colorectal cancer metastasis	neoplastic (KM12SM, KM12C)	-1.64	SILAC	23443137
CTSS	colorectal cancer metastasis	neoplastic (KM12SM, KM12C)	3.92	SILAC	23443137
DDT	colorectal cancer metastasis	neoplastic (KM12SM, KM12C)	-1	SILAC	23443137
EFNA3	colorectal cancer metastasis	neoplastic (KM12SM, KM12C)	-3.05	SILAC	23443137
EPHA4	colorectal cancer metastasis	neoplastic (KM12SM, KM12C)	-1.43	SILAC	23443137
ETFA	colorectal cancer metastasis	neoplastic (KM12SM, KM12C)	-1.25	SILAC	23443137
ETHE1	colorectal cancer metastasis	neoplastic (KM12SM, KM12C)	1.15	SILAC	23443137
FSCN1	colorectal cancer metastasis	neoplastic (KM12SM, KM12C)	1.54	SILAC	23443137
GANAB	colorectal cancer metastasis	neoplastic (KM12SM, KM12C)	-1.18	SILAC	23443137
GDF15	colorectal cancer metastasis	neoplastic (KM12SM, KM12C)	2.63	SILAC	23443137
GNG12	colorectal cancer metastasis	neoplastic (KM12SM, KM12C)	-1.05	SILAC	23443137
GSTP1	colorectal cancer metastasis	neoplastic (KM12SM, KM12C)	2.79	SILAC	23443137
GTF2F1	colorectal cancer metastasis	neoplastic (KM12SM, KM12C)	2.29	SILAC	23443137
GTF2I	colorectal cancer metastasis	neoplastic (KM12SM, KM12C)	1.59	SILAC	23443137
HADHB	colorectal cancer metastasis	neoplastic (KM12SM, KM12C)	-1.51	SILAC	23443137
HIBADH	colorectal cancer metastasis	neoplastic (KM12SM, KM12C)	-1.12	SILAC	23443137
HMGCS1	colorectal cancer metastasis	neoplastic (KM12SM, KM12C)	-1.25	SILAC	23443137
HSPA5	colorectal cancer metastasis	neoplastic (KM12SM, KM12C)	-1.28	SILAC	23443137
HSPE1	colorectal cancer metastasis	neoplastic (KM12SM, KM12C)	-1	SILAC	23443137
IGFBP2	colorectal cancer metastasis	neoplastic (KM12SM, KM12C)	-2.05	SILAC	23443137
ITGA6	colorectal cancer metastasis	neoplastic (KM12SM, KM12C)	-2.55	SILAC	23443137
ITGB4	colorectal cancer metastasis	neoplastic (KM12SM, KM12C)	-2.12	SILAC	23443137
KLK10	colorectal cancer metastasis	neoplastic (KM12SM, KM12C)	-3.32	SILAC	23443137
KLK6	colorectal cancer metastasis	neoplastic (KM12SM, KM12C)	-2.18	SILAC	23443137
LAMA5	colorectal cancer metastasis	neoplastic (KM12SM, KM12C)	-1.05	SILAC	23443137
LAMC1	colorectal cancer metastasis	neoplastic (KM12SM, KM12C)	-2.18	SILAC	23443137
LFNG	colorectal cancer metastasis	neoplastic (KM12SM, KM12C)	-2.05	SILAC	23443137
LUM	colorectal cancer metastasis	neoplastic (KM12SM, KM12C)	-2.32	SILAC	23443137
MAN1B1	colorectal cancer metastasis	neoplastic (KM12SM, KM12C)	-1.18	SILAC	23443137
MANF	colorectal cancer metastasis	neoplastic (KM12SM, KM12C)	-2.64	SILAC	23443137
MARCKSL1	colorectal cancer metastasis	neoplastic (KM12SM, KM12C)	-1	SILAC	23443137
MDK	colorectal cancer metastasis	neoplastic (KM12SM, KM12C)	3.65	SILAC	23443137
MFGE8	colorectal cancer metastasis	neoplastic (KM12SM, KM12C)	-1.94	SILAC	23443137
MGAL1	colorectal cancer metastasis	neoplastic (KM12SM, KM12C)	1.37	SILAC	23443137

MMP1	colorectal cancer metastasis	neoplastic (KM12SM, KM12C)	-2.83	SILAC	23443137
NEO1	colorectal cancer metastasis	neoplastic (KM12SM, KM12C)	-4.64	SILAC	23443137
NPNT	colorectal cancer metastasis	neoplastic (KM12SM, KM12C)	-1.25	SILAC	23443137
PGAM5	colorectal cancer metastasis	neoplastic (KM12SM, KM12C)	-1.08	SILAC	23443137
PGP	colorectal cancer metastasis	neoplastic (KM12SM, KM12C)	-1	SILAC	23443137
PHB2	colorectal cancer metastasis	neoplastic (KM12SM, KM12C)	-1.12	SILAC	23443137
PODXL	colorectal cancer metastasis	neoplastic (KM12SM, KM12C)	3.44	SILAC	23443137
PPIC	colorectal cancer metastasis	neoplastic (KM12SM, KM12C)	-1.73	SILAC	23443137
PROCR	colorectal cancer metastasis	neoplastic (KM12SM, KM12C)	1.49	SILAC	23443137
PRSS22	colorectal cancer metastasis	neoplastic (KM12SM, KM12C)	2.10	SILAC	23443137
PSMB8	colorectal cancer metastasis	neoplastic (KM12SM, KM12C)	1.15	SILAC	23443137
PSMB9	colorectal cancer metastasis	neoplastic (KM12SM, KM12C)	1.65	SILAC	23443137
RAB25	colorectal cancer metastasis	neoplastic (KM12SM, KM12C)	-1.12	SILAC	23443137
RALY	colorectal cancer metastasis	neoplastic (KM12SM, KM12C)	-1.18	SILAC	23443137
S100A8	colorectal cancer metastasis	neoplastic (KM12SM, KM12C)	4.90	SILAC	23443137
SDC1	colorectal cancer metastasis	neoplastic (KM12SM, KM12C)	-1.05	SILAC	23443137
SDF2L1	colorectal cancer metastasis	neoplastic (KM12SM, KM12C)	-1.73	SILAC	23443137
SDF4	colorectal cancer metastasis	neoplastic (KM12SM, KM12C)	-1.68	SILAC	23443137
SELENBP1	colorectal cancer metastasis	neoplastic (KM12SM, KM12C)	1.88	SILAC	23443137
SERPINI1	colorectal cancer metastasis	neoplastic (KM12SM, KM12C)	-2.73	SILAC	23443137
SH3KBP1	colorectal cancer metastasis	neoplastic (KM12SM, KM12C)	1.31	SILAC	23443137
SIL1	colorectal cancer metastasis	neoplastic (KM12SM, KM12C)	-1.15	SILAC	23443137
SLC12A2	colorectal cancer metastasis	neoplastic (KM12SM, KM12C)	-1.02	SILAC	23443137
SOSTDC1	colorectal cancer metastasis	neoplastic (KM12SM, KM12C)	1.75	SILAC	23443137
SSR4	colorectal cancer metastasis	neoplastic (KM12SM, KM12C)	-1.02	SILAC	23443137
ST6GALNAC1	colorectal cancer metastasis	neoplastic (KM12SM, KM12C)	-1.21	SILAC	23443137
STOML2	colorectal cancer metastasis	neoplastic (KM12SM, KM12C)	-1.05	SILAC	23443137
SUB1	colorectal cancer metastasis	neoplastic (KM12SM, KM12C)	-1.02	SILAC	23443137
SYNGR2	colorectal cancer metastasis	neoplastic (KM12SM, KM12C)	-1.08	SILAC	23443137
TAX1BP3	colorectal cancer metastasis	neoplastic (KM12SM, KM12C)	-1.18	SILAC	23443137
TMED4	colorectal cancer metastasis	neoplastic (KM12SM, KM12C)	-1	SILAC	23443137
TMED7	colorectal cancer metastasis	neoplastic (KM12SM, KM12C)	-1.18	SILAC	23443137
TMED9	colorectal cancer metastasis	neoplastic (KM12SM, KM12C)	-1.25	SILAC	23443137
TRIM28	colorectal cancer metastasis	neoplastic (KM12SM, KM12C)	-1.21	SILAC	23443137
TSPAN8	colorectal cancer metastasis	neoplastic (KM12SM, KM12C)	-1	SILAC	23443137
UQCRC2	colorectal cancer metastasis	neoplastic (KM12SM, KM12C)	-1.02	SILAC	23443137
UQCRFS1	colorectal cancer metastasis	neoplastic (KM12SM, KM12C)	-1.12	SILAC	23443137
VDAC1	colorectal cancer metastasis	neoplastic (KM12SM, KM12C)	-1.28	SILAC	23443137
VGF	colorectal cancer metastasis	neoplastic (KM12SM, KM12C)	3.27	SILAC	23443137
ZG16B	colorectal cancer metastasis	neoplastic (KM12SM, KM12C)	1.77	SILAC	23443137
A1BG	head and neck cancer	neoplastic (JHU-O28, JHU-O22, JHU-O11, JHU-O29, OFK6/TERT1, FaDu, CAL 27)	1.51	iTRAQ	23665456
A2M	head and neck cancer	neoplastic (JHU-O28, JHU-O22, JHU-O11, JHU-O29, OFK6/TERT1, FaDu, CAL 27)	2.63	iTRAQ	23665456
AAK1	head and neck cancer	neoplastic (JHU-O28, JHU-O22, JHU-O11, JHU-O29, OFK6/TERT1, FaDu, CAL 27)	-1.15	iTRAQ	23665456

ABCA4	head and neck cancer	neoplastic (JHU-O28, JHU-O22, JHU-O11, JHU-O29, OFK6/TERT1, FaDu, CAL 27)	5.76	iTRAQ	23665456
ABCF1	head and neck cancer	neoplastic (JHU-O28, JHU-O22, JHU-O11, JHU-O29, OFK6/TERT1, FaDu, CAL 27)	2.56	iTRAQ	23665456
ABHD10	head and neck cancer	neoplastic (JHU-O28, JHU-O22, JHU-O11, JHU-O29, OFK6/TERT1, FaDu, CAL 27)	-1.39	iTRAQ	23665456
ABHD11	head and neck cancer	neoplastic (JHU-O28, JHU-O22, JHU-O11, JHU-O29, OFK6/TERT1, FaDu, CAL 27)	-2.05	iTRAQ	23665456
ABHD14B	head and neck cancer	neoplastic (JHU-O28, JHU-O22, JHU-O11, JHU-O29, OFK6/TERT1, FaDu, CAL 27)	-1.51	iTRAQ	23665456
ACAT2	head and neck cancer	neoplastic (JHU-O28, JHU-O22, JHU-O11, JHU-O29, OFK6/TERT1, FaDu, CAL 27)	-2.18	iTRAQ	23665456
ACLY	head and neck cancer	neoplastic (JHU-O28, JHU-O22, JHU-O11, JHU-O29, OFK6/TERT1, FaDu, CAL 27)	-3.32	iTRAQ	23665456
ACOT1	head and neck cancer	neoplastic (JHU-O28, JHU-O22, JHU-O11, JHU-O29, OFK6/TERT1, FaDu, CAL 27)	1.04	iTRAQ	23665456
ACP5	head and neck cancer	neoplastic (JHU-O28, JHU-O22, JHU-O11, JHU-O29, OFK6/TERT1, FaDu, CAL 27)	1.43	iTRAQ	23665456
ACSF3	head and neck cancer	neoplastic (JHU-O28, JHU-O22, JHU-O11, JHU-O29, OFK6/TERT1, FaDu, CAL 27)	-1.78	iTRAQ	23665456
ACSS2	head and neck cancer	neoplastic (JHU-O28, JHU-O22, JHU-O11, JHU-O29, OFK6/TERT1, FaDu, CAL 27)	-1.55	iTRAQ	23665456
ACY1	head and neck cancer	neoplastic (JHU-O28, JHU-O22, JHU-O11, JHU-O29, OFK6/TERT1, FaDu, CAL 27)	-2.39	iTRAQ	23665456
ADA	head and neck cancer	neoplastic (JHU-O28, JHU-O22, JHU-O11, JHU-O29, OFK6/TERT1, FaDu, CAL 27)	-1.43	iTRAQ	23665456
ADI1	head and neck cancer	neoplastic (JHU-O28, JHU-O22, JHU-O11, JHU-O29, OFK6/TERT1, FaDu, CAL 27)	-1.05	iTRAQ	23665456
ADSL	head and neck cancer	neoplastic (JHU-O28, JHU-O22, JHU-O11, JHU-O29, OFK6/TERT1, FaDu, CAL 27)	1.05	iTRAQ	23665456
ADSS	head and neck cancer	neoplastic (JHU-O28, JHU-O22, JHU-O11, JHU-O29, OFK6/TERT1, FaDu, CAL 27)	-1.18	iTRAQ	23665456
AFAP1L2	head and neck cancer	neoplastic (JHU-O28, JHU-O22, JHU-O11, JHU-O29, OFK6/TERT1, FaDu, CAL 27)	1.09	iTRAQ	23665456
AFF4	head and neck cancer	neoplastic (JHU-O28, JHU-O22, JHU-O11, JHU-O29, OFK6/TERT1, FaDu, CAL 27)	5.23	iTRAQ	23665456
AFP	head and neck cancer	neoplastic (JHU-O28, JHU-O22, JHU-O11, JHU-O29, OFK6/TERT1, FaDu, CAL 27)	3.30	iTRAQ	23665456
AGFG1	head and neck cancer	neoplastic (JHU-O28, JHU-O22, JHU-O11, JHU-O29, OFK6/TERT1, FaDu, CAL 27)	-1.73	iTRAQ	23665456
AGRN	head and neck cancer	neoplastic (JHU-O28, JHU-O22, JHU-O11, JHU-O29, OFK6/TERT1, FaDu, CAL 27)	1.70	iTRAQ	23665456
AHCTF1	head and neck cancer	neoplastic (JHU-O28, JHU-O22, JHU-O11, JHU-O29, OFK6/TERT1, FaDu, CAL 27)	-1.18	iTRAQ	23665456
AHNAK	head and neck cancer	neoplastic (JHU-O28, JHU-O22, JHU-O11, JHU-O29, OFK6/TERT1, FaDu, CAL 27)	-1.64	iTRAQ	23665456
AHNAK2	head and neck cancer	neoplastic (JHU-O28, JHU-O22, JHU-O11, JHU-O29, OFK6/TERT1, FaDu, CAL 27)	-1.43	iTRAQ	23665456
AHSG	head and neck cancer	neoplastic (JHU-O28, JHU-O22, JHU-O11, JHU-O29, OFK6/TERT1, FaDu, CAL 27)	2.63	iTRAQ	23665456
AIMP2	head and neck cancer	neoplastic (JHU-O28, JHU-O22, JHU-O11, JHU-O29, OFK6/TERT1, FaDu, CAL 27)	1.78	iTRAQ	23665456
AK2	head and neck cancer	neoplastic (JHU-O28, JHU-O22, JHU-O11, JHU-O29, OFK6/TERT1, FaDu, CAL 27)	-2.25	iTRAQ	23665456
AKAP12	head and neck cancer	neoplastic (JHU-O28, JHU-O22, JHU-O11, JHU-O29, OFK6/TERT1, FaDu, CAL 27)	1.28	iTRAQ	23665456
AKR1A1	head and neck cancer	neoplastic (JHU-O28, JHU-O22, JHU-O11, JHU-O29, OFK6/TERT1, FaDu, CAL 27)	-1.15	iTRAQ	23665456
AKR1C2	head and neck cancer	neoplastic (JHU-O28, JHU-O22, JHU-O11, JHU-O29, OFK6/TERT1, FaDu, CAL 27)	-1.12	iTRAQ	23665456
AKR1C3	head and neck cancer	neoplastic (JHU-O28, JHU-O22, JHU-O11, JHU-O29, OFK6/TERT1, FaDu, CAL 27)	-1.43	iTRAQ	23665456
AKR1D1	head and neck cancer	neoplastic (JHU-O28, JHU-O22, JHU-O11, JHU-O29, OFK6/TERT1, FaDu, CAL 27)	-1.39	iTRAQ	23665456
ALB	head and neck cancer	neoplastic (JHU-O28, JHU-O22, JHU-O11, JHU-O29, OFK6/TERT1, FaDu, CAL 27)	-1.64	iTRAQ	23665456
ALB	head and neck cancer	neoplastic (JHU-O28, JHU-O22, JHU-O11, JHU-O29, OFK6/TERT1, FaDu, CAL 27)	4.62	iTRAQ	23665456
ALDH7A1	head and neck cancer	neoplastic (JHU-O28, JHU-O22, JHU-O11, JHU-O29, OFK6/TERT1, FaDu, CAL 27)	-1.68	iTRAQ	23665456
ALDOA	head and neck cancer	neoplastic (JHU-O28, JHU-O22, JHU-O11, JHU-O29, OFK6/TERT1, FaDu, CAL 27)	1.76	iTRAQ	23665456
ALDOC	head and neck cancer	neoplastic (JHU-O28, JHU-O22, JHU-O11, JHU-O29, OFK6/TERT1, FaDu, CAL 27)	-1.39	iTRAQ	23665456
AMIGO2	head and neck cancer	neoplastic (JHU-O28, JHU-O22, JHU-O11, JHU-O29, OFK6/TERT1, FaDu, CAL 27)	-1.02	iTRAQ	23665456
AMY2B	head and neck cancer	neoplastic (JHU-O28, JHU-O22, JHU-O11, JHU-O29, OFK6/TERT1, FaDu, CAL 27)	1.62	iTRAQ	23665456
ANGPTL4	head and neck cancer	neoplastic (JHU-O28, JHU-O22, JHU-O11, JHU-O29, OFK6/TERT1, FaDu, CAL 27)	1.25	iTRAQ	23665456
ANP32B	head and neck cancer	neoplastic (JHU-O28, JHU-O22, JHU-O11, JHU-O29, OFK6/TERT1, FaDu, CAL 27)	2.12	iTRAQ	23665456
ANPEP	head and neck cancer	neoplastic (JHU-O28, JHU-O22, JHU-O11, JHU-O29, OFK6/TERT1, FaDu, CAL 27)	-2.18	iTRAQ	23665456
ANTXR2	head and neck cancer	neoplastic (JHU-O28, JHU-O22, JHU-O11, JHU-O29, OFK6/TERT1, FaDu, CAL 27)	-3.05	iTRAQ	23665456

ANXA1	head and neck cancer	neoplastic (JHU-O28, JHU-O22, JHU-O11, JHU-O29, OFK6/TERT1, FaDu, CAL 27)	2.36	iTRAQ	23665456
ANXA2	head and neck cancer	neoplastic (JHU-O28, JHU-O22, JHU-O11, JHU-O29, OFK6/TERT1, FaDu, CAL 27)	2.74	iTRAQ	23665456
ANXA2	head and neck cancer	neoplastic (JHU-O28, JHU-O22, JHU-O11, JHU-O29, OFK6/TERT1, FaDu, CAL 27)	-3.32	iTRAQ	23665456
ANXA3	head and neck cancer	neoplastic (JHU-O28, JHU-O22, JHU-O11, JHU-O29, OFK6/TERT1, FaDu, CAL 27)	1.73	iTRAQ	23665456
ANXA5	head and neck cancer	neoplastic (JHU-O28, JHU-O22, JHU-O11, JHU-O29, OFK6/TERT1, FaDu, CAL 27)	2.42	iTRAQ	23665456
ANXA6	head and neck cancer	neoplastic (JHU-O28, JHU-O22, JHU-O11, JHU-O29, OFK6/TERT1, FaDu, CAL 27)	1.28	iTRAQ	23665456
ANXA7	head and neck cancer	neoplastic (JHU-O28, JHU-O22, JHU-O11, JHU-O29, OFK6/TERT1, FaDu, CAL 27)	2.16	iTRAQ	23665456
ANXA8L2	head and neck cancer	neoplastic (JHU-O28, JHU-O22, JHU-O11, JHU-O29, OFK6/TERT1, FaDu, CAL 27)	1.63	iTRAQ	23665456
AP1S1	head and neck cancer	neoplastic (JHU-O28, JHU-O22, JHU-O11, JHU-O29, OFK6/TERT1, FaDu, CAL 27)	1.21	iTRAQ	23665456
AP2A1	head and neck cancer	neoplastic (JHU-O28, JHU-O22, JHU-O11, JHU-O29, OFK6/TERT1, FaDu, CAL 27)	-1.32	iTRAQ	23665456
AP2S1	head and neck cancer	neoplastic (JHU-O28, JHU-O22, JHU-O11, JHU-O29, OFK6/TERT1, FaDu, CAL 27)	1.52	iTRAQ	23665456
AP3D1	head and neck cancer	neoplastic (JHU-O28, JHU-O22, JHU-O11, JHU-O29, OFK6/TERT1, FaDu, CAL 27)	1	iTRAQ	23665456
APEH	head and neck cancer	neoplastic (JHU-O28, JHU-O22, JHU-O11, JHU-O29, OFK6/TERT1, FaDu, CAL 27)	2.53	iTRAQ	23665456
APEX1	head and neck cancer	neoplastic (JHU-O28, JHU-O22, JHU-O11, JHU-O29, OFK6/TERT1, FaDu, CAL 27)	2.76	iTRAQ	23665456
APLP2	head and neck cancer	neoplastic (JHU-O28, JHU-O22, JHU-O11, JHU-O29, OFK6/TERT1, FaDu, CAL 27)	2.28	iTRAQ	23665456
APOA1	head and neck cancer	neoplastic (JHU-O28, JHU-O22, JHU-O11, JHU-O29, OFK6/TERT1, FaDu, CAL 27)	1.79	iTRAQ	23665456
APOA1BP	head and neck cancer	neoplastic (JHU-O28, JHU-O22, JHU-O11, JHU-O29, OFK6/TERT1, FaDu, CAL 27)	-1.25	iTRAQ	23665456
APOA1BP	head and neck cancer	neoplastic (JHU-O28, JHU-O22, JHU-O11, JHU-O29, OFK6/TERT1, FaDu, CAL 27)	-1.32	iTRAQ	23665456
APOC3	head and neck cancer	neoplastic (JHU-O28, JHU-O22, JHU-O11, JHU-O29, OFK6/TERT1, FaDu, CAL 27)	2.19	iTRAQ	23665456
APOH	head and neck cancer	neoplastic (JHU-O28, JHU-O22, JHU-O11, JHU-O29, OFK6/TERT1, FaDu, CAL 27)	2.14	iTRAQ	23665456
APP	head and neck cancer	neoplastic (JHU-O28, JHU-O22, JHU-O11, JHU-O29, OFK6/TERT1, FaDu, CAL 27)	2.53	iTRAQ	23665456
AREGB	head and neck cancer	neoplastic (JHU-O28, JHU-O22, JHU-O11, JHU-O29, OFK6/TERT1, FaDu, CAL 27)	-2.18	iTRAQ	23665456
ARFIP1	head and neck cancer	neoplastic (JHU-O28, JHU-O22, JHU-O11, JHU-O29, OFK6/TERT1, FaDu, CAL 27)	-1.05	iTRAQ	23665456
ARHGAP1	head and neck cancer	neoplastic (JHU-O28, JHU-O22, JHU-O11, JHU-O29, OFK6/TERT1, FaDu, CAL 27)	-1.64	iTRAQ	23665456
ARHGDIA	head and neck cancer	neoplastic (JHU-O28, JHU-O22, JHU-O11, JHU-O29, OFK6/TERT1, FaDu, CAL 27)	-1.15	iTRAQ	23665456
ARHGDIB	head and neck cancer	neoplastic (JHU-O28, JHU-O22, JHU-O11, JHU-O29, OFK6/TERT1, FaDu, CAL 27)	-2	iTRAQ	23665456
ARHGEF18	head and neck cancer	neoplastic (JHU-O28, JHU-O22, JHU-O11, JHU-O29, OFK6/TERT1, FaDu, CAL 27)	6.92	iTRAQ	23665456
ARHGEF5	head and neck cancer	neoplastic (JHU-O28, JHU-O22, JHU-O11, JHU-O29, OFK6/TERT1, FaDu, CAL 27)	-1.02	iTRAQ	23665456
ARL3	head and neck cancer	neoplastic (JHU-O28, JHU-O22, JHU-O11, JHU-O29, OFK6/TERT1, FaDu, CAL 27)	-1	iTRAQ	23665456
ARMC10	head and neck cancer	neoplastic (JHU-O28, JHU-O22, JHU-O11, JHU-O29, OFK6/TERT1, FaDu, CAL 27)	-2.05	iTRAQ	23665456
ARPP19	head and neck cancer	neoplastic (JHU-O28, JHU-O22, JHU-O11, JHU-O29, OFK6/TERT1, FaDu, CAL 27)	-1.12	iTRAQ	23665456
ASAP2	head and neck cancer	neoplastic (JHU-O28, JHU-O22, JHU-O11, JHU-O29, OFK6/TERT1, FaDu, CAL 27)	1.33	iTRAQ	23665456
ASNS	head and neck cancer	neoplastic (JHU-O28, JHU-O22, JHU-O11, JHU-O29, OFK6/TERT1, FaDu, CAL 27)	2.40	iTRAQ	23665456
ATOX1	head and neck cancer	neoplastic (JHU-O28, JHU-O22, JHU-O11, JHU-O29, OFK6/TERT1, FaDu, CAL 27)	-1.59	iTRAQ	23665456
ATP1A1	head and neck cancer	neoplastic (JHU-O28, JHU-O22, JHU-O11, JHU-O29, OFK6/TERT1, FaDu, CAL 27)	1.62	iTRAQ	23665456
AVP	head and neck cancer	neoplastic (JHU-O28, JHU-O22, JHU-O11, JHU-O29, OFK6/TERT1, FaDu, CAL 27)	-1.35	iTRAQ	23665456
B4GALT5	head and neck cancer	neoplastic (JHU-O28, JHU-O22, JHU-O11, JHU-O29, OFK6/TERT1, FaDu, CAL 27)	1.69	iTRAQ	23665456
BAG2	head and neck cancer	neoplastic (JHU-O28, JHU-O22, JHU-O11, JHU-O29, OFK6/TERT1, FaDu, CAL 27)	2.85	iTRAQ	23665456
BAIAP2L1	head and neck cancer	neoplastic (JHU-O28, JHU-O22, JHU-O11, JHU-O29, OFK6/TERT1, FaDu, CAL 27)	-1.25	iTRAQ	23665456
BANF1	head and neck cancer	neoplastic (JHU-O28, JHU-O22, JHU-O11, JHU-O29, OFK6/TERT1, FaDu, CAL 27)	2	iTRAQ	23665456
BASP1	head and neck cancer	neoplastic (JHU-O28, JHU-O22, JHU-O11, JHU-O29, OFK6/TERT1, FaDu, CAL 27)	-1.25	iTRAQ	23665456
BCAR1	head and neck cancer	neoplastic (JHU-O28, JHU-O22, JHU-O11, JHU-O29, OFK6/TERT1, FaDu, CAL 27)	-1.43	iTRAQ	23665456
BCAT2	head and neck cancer	neoplastic (JHU-O28, JHU-O22, JHU-O11, JHU-O29, OFK6/TERT1, FaDu, CAL 27)	-1.35	iTRAQ	23665456

BCCIP	head and neck cancer	neoplastic (JHU-O28, JHU-O22, JHU-O11, JHU-O29, OFK6/TERT1, FaDu, CAL 27)	1.26	iTRAQ	23665456
BCLAF1	head and neck cancer	neoplastic (JHU-O28, JHU-O22, JHU-O11, JHU-O29, OFK6/TERT1, FaDu, CAL 27)	2.79	iTRAQ	23665456
BIRC6	head and neck cancer	neoplastic (JHU-O28, JHU-O22, JHU-O11, JHU-O29, OFK6/TERT1, FaDu, CAL 27)	1.65	iTRAQ	23665456
BMP1	head and neck cancer	neoplastic (JHU-O28, JHU-O22, JHU-O11, JHU-O29, OFK6/TERT1, FaDu, CAL 27)	1.02	iTRAQ	23665456
BPGM	head and neck cancer	neoplastic (JHU-O28, JHU-O22, JHU-O11, JHU-O29, OFK6/TERT1, FaDu, CAL 27)	-1.05	iTRAQ	23665456
BPIL2	head and neck cancer	neoplastic (JHU-O28, JHU-O22, JHU-O11, JHU-O29, OFK6/TERT1, FaDu, CAL 27)	-2.32	iTRAQ	23665456
BPNT1	head and neck cancer	neoplastic (JHU-O28, JHU-O22, JHU-O11, JHU-O29, OFK6/TERT1, FaDu, CAL 27)	-1.73	iTRAQ	23665456
BROX	head and neck cancer	neoplastic (JHU-O28, JHU-O22, JHU-O11, JHU-O29, OFK6/TERT1, FaDu, CAL 27)	-1.02	iTRAQ	23665456
BTF3	head and neck cancer	neoplastic (JHU-O28, JHU-O22, JHU-O11, JHU-O29, OFK6/TERT1, FaDu, CAL 27)	2.27	iTRAQ	23665456
BUB3	head and neck cancer	neoplastic (JHU-O28, JHU-O22, JHU-O11, JHU-O29, OFK6/TERT1, FaDu, CAL 27)	1.33	iTRAQ	23665456
C10orf116	head and neck cancer	neoplastic (JHU-O28, JHU-O22, JHU-O11, JHU-O29, OFK6/TERT1, FaDu, CAL 27)	-1.15	iTRAQ	23665456
C11orf67	head and neck cancer	neoplastic (JHU-O28, JHU-O22, JHU-O11, JHU-O29, OFK6/TERT1, FaDu, CAL 27)	-1.05	iTRAQ	23665456
C11orf68	head and neck cancer	neoplastic (JHU-O28, JHU-O22, JHU-O11, JHU-O29, OFK6/TERT1, FaDu, CAL 27)	-1	iTRAQ	23665456
C12orf10	head and neck cancer	neoplastic (JHU-O28, JHU-O22, JHU-O11, JHU-O29, OFK6/TERT1, FaDu, CAL 27)	-1.12	iTRAQ	23665456
C12orf5	head and neck cancer	neoplastic (JHU-O28, JHU-O22, JHU-O11, JHU-O29, OFK6/TERT1, FaDu, CAL 27)	-2.18	iTRAQ	23665456
C13orf33	head and neck cancer	neoplastic (JHU-O28, JHU-O22, JHU-O11, JHU-O29, OFK6/TERT1, FaDu, CAL 27)	2.13	iTRAQ	23665456
C14orf135	head and neck cancer	neoplastic (JHU-O28, JHU-O22, JHU-O11, JHU-O29, OFK6/TERT1, FaDu, CAL 27)	-2.05	iTRAQ	23665456
C17orf49	head and neck cancer	neoplastic (JHU-O28, JHU-O22, JHU-O11, JHU-O29, OFK6/TERT1, FaDu, CAL 27)	-1.35	iTRAQ	23665456
C19orf10	head and neck cancer	neoplastic (JHU-O28, JHU-O22, JHU-O11, JHU-O29, OFK6/TERT1, FaDu, CAL 27)	-1	iTRAQ	23665456
C19orf10	head and neck cancer	neoplastic (JHU-O28, JHU-O22, JHU-O11, JHU-O29, OFK6/TERT1, FaDu, CAL 27)	-1.32	iTRAQ	23665456
C1orf116	head and neck cancer	neoplastic (JHU-O28, JHU-O22, JHU-O11, JHU-O29, OFK6/TERT1, FaDu, CAL 27)	-1.32	iTRAQ	23665456
C1orf50	head and neck cancer	neoplastic (JHU-O28, JHU-O22, JHU-O11, JHU-O29, OFK6/TERT1, FaDu, CAL 27)	-1.43	iTRAQ	23665456
C1QBP	head and neck cancer	neoplastic (JHU-O28, JHU-O22, JHU-O11, JHU-O29, OFK6/TERT1, FaDu, CAL 27)	-1.73	iTRAQ	23665456
C1QBP	head and neck cancer	neoplastic (JHU-O28, JHU-O22, JHU-O11, JHU-O29, OFK6/TERT1, FaDu, CAL 27)	2.47	iTRAQ	23665456
C1QTNF3	head and neck cancer	neoplastic (JHU-O28, JHU-O22, JHU-O11, JHU-O29, OFK6/TERT1, FaDu, CAL 27)	1.75	iTRAQ	23665456
C1R	head and neck cancer	neoplastic (JHU-O28, JHU-O22, JHU-O11, JHU-O29, OFK6/TERT1, FaDu, CAL 27)	1.72	iTRAQ	23665456
C1S	head and neck cancer	neoplastic (JHU-O28, JHU-O22, JHU-O11, JHU-O29, OFK6/TERT1, FaDu, CAL 27)	2.49	iTRAQ	23665456
C21orf33	head and neck cancer	neoplastic (JHU-O28, JHU-O22, JHU-O11, JHU-O29, OFK6/TERT1, FaDu, CAL 27)	-1	iTRAQ	23665456
C3orf64	head and neck cancer	neoplastic (JHU-O28, JHU-O22, JHU-O11, JHU-O29, OFK6/TERT1, FaDu, CAL 27)	-1.64	iTRAQ	23665456
C5orf42	head and neck cancer	neoplastic (JHU-O28, JHU-O22, JHU-O11, JHU-O29, OFK6/TERT1, FaDu, CAL 27)	-4.32	iTRAQ	23665456
C6orf115	head and neck cancer	neoplastic (JHU-O28, JHU-O22, JHU-O11, JHU-O29, OFK6/TERT1, FaDu, CAL 27)	-2.39	iTRAQ	23665456
C6orf211	head and neck cancer	neoplastic (JHU-O28, JHU-O22, JHU-O11, JHU-O29, OFK6/TERT1, FaDu, CAL 27)	-1.43	iTRAQ	23665456
C9orf117	head and neck cancer	neoplastic (JHU-O28, JHU-O22, JHU-O11, JHU-O29, OFK6/TERT1, FaDu, CAL 27)	-1.68	iTRAQ	23665456
CA12	head and neck cancer	neoplastic (JHU-O28, JHU-O22, JHU-O11, JHU-O29, OFK6/TERT1, FaDu, CAL 27)	-2.05	iTRAQ	23665456
CA2	head and neck cancer	neoplastic (JHU-O28, JHU-O22, JHU-O11, JHU-O29, OFK6/TERT1, FaDu, CAL 27)	-1	iTRAQ	23665456
CAB39	head and neck cancer	neoplastic (JHU-O28, JHU-O22, JHU-O11, JHU-O29, OFK6/TERT1, FaDu, CAL 27)	-1.73	iTRAQ	23665456
CACYBP	head and neck cancer	neoplastic (JHU-O28, JHU-O22, JHU-O11, JHU-O29, OFK6/TERT1, FaDu, CAL 27)	2.02	iTRAQ	23665456
CAD	head and neck cancer	neoplastic (JHU-O28, JHU-O22, JHU-O11, JHU-O29, OFK6/TERT1, FaDu, CAL 27)	1.42	iTRAQ	23665456
CALR	head and neck cancer	neoplastic (JHU-O28, JHU-O22, JHU-O11, JHU-O29, OFK6/TERT1, FaDu, CAL 27)	2.54	iTRAQ	23665456
CALR	head and neck cancer	neoplastic (JHU-O28, JHU-O22, JHU-O11, JHU-O29, OFK6/TERT1, FaDu, CAL 27)	2.78	iTRAQ	23665456
CALU	head and neck cancer	neoplastic (JHU-O28, JHU-O22, JHU-O11, JHU-O29, OFK6/TERT1, FaDu, CAL 27)	-1.83	iTRAQ	23665456
CAND1	head and neck cancer	neoplastic (JHU-O28, JHU-O22, JHU-O11, JHU-O29, OFK6/TERT1, FaDu, CAL 27)	1.73	iTRAQ	23665456
CANX	head and neck cancer	neoplastic (JHU-O28, JHU-O22, JHU-O11, JHU-O29, OFK6/TERT1, FaDu, CAL 27)	1.65	iTRAQ	23665456

CAP1	head and neck cancer	neoplastic (JHU-O28, JHU-O22, JHU-O11, JHU-O29, OFK6/TERT1, FaDu, CAL 27)	-1.32	iTRAQ	23665456
CAPG	head and neck cancer	neoplastic (JHU-O28, JHU-O22, JHU-O11, JHU-O29, OFK6/TERT1, FaDu, CAL 27)	-2.64	iTRAQ	23665456
CAPRIN1	head and neck cancer	neoplastic (JHU-O28, JHU-O22, JHU-O11, JHU-O29, OFK6/TERT1, FaDu, CAL 27)	1.68	iTRAQ	23665456
CASP1	head and neck cancer	neoplastic (JHU-O28, JHU-O22, JHU-O11, JHU-O29, OFK6/TERT1, FaDu, CAL 27)	-1.21	iTRAQ	23665456
CBR1	head and neck cancer	neoplastic (JHU-O28, JHU-O22, JHU-O11, JHU-O29, OFK6/TERT1, FaDu, CAL 27)	-2.25	iTRAQ	23665456
CBX3	head and neck cancer	neoplastic (JHU-O28, JHU-O22, JHU-O11, JHU-O29, OFK6/TERT1, FaDu, CAL 27)	1.63	iTRAQ	23665456
CCBL1	head and neck cancer	neoplastic (JHU-O28, JHU-O22, JHU-O11, JHU-O29, OFK6/TERT1, FaDu, CAL 27)	-1.47	iTRAQ	23665456
CCBL2	head and neck cancer	neoplastic (JHU-O28, JHU-O22, JHU-O11, JHU-O29, OFK6/TERT1, FaDu, CAL 27)	-1.55	iTRAQ	23665456
CCDC124	head and neck cancer	neoplastic (JHU-O28, JHU-O22, JHU-O11, JHU-O29, OFK6/TERT1, FaDu, CAL 27)	-1.12	iTRAQ	23665456
CCDC58	head and neck cancer	neoplastic (JHU-O28, JHU-O22, JHU-O11, JHU-O29, OFK6/TERT1, FaDu, CAL 27)	-1.47	iTRAQ	23665456
CCDC80	head and neck cancer	neoplastic (JHU-O28, JHU-O22, JHU-O11, JHU-O29, OFK6/TERT1, FaDu, CAL 27)	1.59	iTRAQ	23665456
CCL2	head and neck cancer	neoplastic (JHU-O28, JHU-O22, JHU-O11, JHU-O29, OFK6/TERT1, FaDu, CAL 27)	3.17	iTRAQ	23665456
CCT2	head and neck cancer	neoplastic (JHU-O28, JHU-O22, JHU-O11, JHU-O29, OFK6/TERT1, FaDu, CAL 27)	1.14	iTRAQ	23665456
CCT4	head and neck cancer	neoplastic (JHU-O28, JHU-O22, JHU-O11, JHU-O29, OFK6/TERT1, FaDu, CAL 27)	1.24	iTRAQ	23665456
CCT4	head and neck cancer	neoplastic (JHU-O28, JHU-O22, JHU-O11, JHU-O29, OFK6/TERT1, FaDu, CAL 27)	-3.32	iTRAQ	23665456
CCT6A	head and neck cancer	neoplastic (JHU-O28, JHU-O22, JHU-O11, JHU-O29, OFK6/TERT1, FaDu, CAL 27)	1.09	iTRAQ	23665456
CCT6A	head and neck cancer	neoplastic (JHU-O28, JHU-O22, JHU-O11, JHU-O29, OFK6/TERT1, FaDu, CAL 27)	1.32	iTRAQ	23665456
CCT8	head and neck cancer	neoplastic (JHU-O28, JHU-O22, JHU-O11, JHU-O29, OFK6/TERT1, FaDu, CAL 27)	2.03	iTRAQ	23665456
CD109	head and neck cancer	neoplastic (JHU-O28, JHU-O22, JHU-O11, JHU-O29, OFK6/TERT1, FaDu, CAL 27)	-1.35	iTRAQ	23665456
CD14	head and neck cancer	neoplastic (JHU-O28, JHU-O22, JHU-O11, JHU-O29, OFK6/TERT1, FaDu, CAL 27)	-1.73	iTRAQ	23665456
CD151	head and neck cancer	neoplastic (JHU-O28, JHU-O22, JHU-O11, JHU-O29, OFK6/TERT1, FaDu, CAL 27)	1.98	iTRAQ	23665456
CD47	head and neck cancer	neoplastic (JHU-O28, JHU-O22, JHU-O11, JHU-O29, OFK6/TERT1, FaDu, CAL 27)	1.43	iTRAQ	23665456
CD82	head and neck cancer	neoplastic (JHU-O28, JHU-O22, JHU-O11, JHU-O29, OFK6/TERT1, FaDu, CAL 27)	2.31	iTRAQ	23665456
CD9	head and neck cancer	neoplastic (JHU-O28, JHU-O22, JHU-O11, JHU-O29, OFK6/TERT1, FaDu, CAL 27)	1.70	iTRAQ	23665456
CDA	head and neck cancer	neoplastic (JHU-O28, JHU-O22, JHU-O11, JHU-O29, OFK6/TERT1, FaDu, CAL 27)	-1.43	iTRAQ	23665456
CDC42EP4	head and neck cancer	neoplastic (JHU-O28, JHU-O22, JHU-O11, JHU-O29, OFK6/TERT1, FaDu, CAL 27)	-1.88	iTRAQ	23665456
CDH1	head and neck cancer	neoplastic (JHU-O28, JHU-O22, JHU-O11, JHU-O29, OFK6/TERT1, FaDu, CAL 27)	-1.08	iTRAQ	23665456
CDH11	head and neck cancer	neoplastic (JHU-O28, JHU-O22, JHU-O11, JHU-O29, OFK6/TERT1, FaDu, CAL 27)	-1.02	iTRAQ	23665456
CDH2	head and neck cancer	neoplastic (JHU-O28, JHU-O22, JHU-O11, JHU-O29, OFK6/TERT1, FaDu, CAL 27)	2.52	iTRAQ	23665456
CDH3	head and neck cancer	neoplastic (JHU-O28, JHU-O22, JHU-O11, JHU-O29, OFK6/TERT1, FaDu, CAL 27)	-1.47	iTRAQ	23665456
CDH4	head and neck cancer	neoplastic (JHU-O28, JHU-O22, JHU-O11, JHU-O29, OFK6/TERT1, FaDu, CAL 27)	1.59	iTRAQ	23665456
CEP170	head and neck cancer	neoplastic (JHU-O28, JHU-O22, JHU-O11, JHU-O29, OFK6/TERT1, FaDu, CAL 27)	-1.43	iTRAQ	23665456
CES2	head and neck cancer	neoplastic (JHU-O28, JHU-O22, JHU-O11, JHU-O29, OFK6/TERT1, FaDu, CAL 27)	-1.59	iTRAQ	23665456
CFB	head and neck cancer	neoplastic (JHU-O28, JHU-O22, JHU-O11, JHU-O29, OFK6/TERT1, FaDu, CAL 27)	1.20	iTRAQ	23665456
CFL1	head and neck cancer	neoplastic (JHU-O28, JHU-O22, JHU-O11, JHU-O29, OFK6/TERT1, FaDu, CAL 27)	2.72	iTRAQ	23665456
CGRRF1	head and neck cancer	neoplastic (JHU-O28, JHU-O22, JHU-O11, JHU-O29, OFK6/TERT1, FaDu, CAL 27)	2.32	iTRAQ	23665456
CHID1	head and neck cancer	neoplastic (JHU-O28, JHU-O22, JHU-O11, JHU-O29, OFK6/TERT1, FaDu, CAL 27)	1.86	iTRAQ	23665456
CHM	head and neck cancer	neoplastic (JHU-O28, JHU-O22, JHU-O11, JHU-O29, OFK6/TERT1, FaDu, CAL 27)	6.24	iTRAQ	23665456
CIAPIN1	head and neck cancer	neoplastic (JHU-O28, JHU-O22, JHU-O11, JHU-O29, OFK6/TERT1, FaDu, CAL 27)	-1.47	iTRAQ	23665456
CKMT1A	head and neck cancer	neoplastic (JHU-O28, JHU-O22, JHU-O11, JHU-O29, OFK6/TERT1, FaDu, CAL 27)	-2.39	iTRAQ	23665456
CLCA2	head and neck cancer	neoplastic (JHU-O28, JHU-O22, JHU-O11, JHU-O29, OFK6/TERT1, FaDu, CAL 27)	-2.25	iTRAQ	23665456
CLCC1	head and neck cancer	neoplastic (JHU-O28, JHU-O22, JHU-O11, JHU-O29, OFK6/TERT1, FaDu, CAL 27)	-1.68	iTRAQ	23665456
CLDN1	head and neck cancer	neoplastic (JHU-O28, JHU-O22, JHU-O11, JHU-O29, OFK6/TERT1, FaDu, CAL 27)	1.46	iTRAQ	23665456

CLIC1	head and neck cancer	neoplastic (JHU-O28, JHU-O22, JHU-O11, JHU-O29, OFK6/TERT1, FaDu, CAL 27)	-1.32	iTRAQ	23665456
CLIC4	head and neck cancer	neoplastic (JHU-O28, JHU-O22, JHU-O11, JHU-O29, OFK6/TERT1, FaDu, CAL 27)	-1.64	iTRAQ	23665456
CLINT1	head and neck cancer	neoplastic (JHU-O28, JHU-O22, JHU-O11, JHU-O29, OFK6/TERT1, FaDu, CAL 27)	-1.47	iTRAQ	23665456
CLIP1	head and neck cancer	neoplastic (JHU-O28, JHU-O22, JHU-O11, JHU-O29, OFK6/TERT1, FaDu, CAL 27)	3.97	iTRAQ	23665456
CLNS1A	head and neck cancer	neoplastic (JHU-O28, JHU-O22, JHU-O11, JHU-O29, OFK6/TERT1, FaDu, CAL 27)	1.00	iTRAQ	23665456
CLSTN1	head and neck cancer	neoplastic (JHU-O28, JHU-O22, JHU-O11, JHU-O29, OFK6/TERT1, FaDu, CAL 27)	1.02	iTRAQ	23665456
CLTB	head and neck cancer	neoplastic (JHU-O28, JHU-O22, JHU-O11, JHU-O29, OFK6/TERT1, FaDu, CAL 27)	-1	iTRAQ	23665456
CLTC	head and neck cancer	neoplastic (JHU-O28, JHU-O22, JHU-O11, JHU-O29, OFK6/TERT1, FaDu, CAL 27)	1.65	iTRAQ	23665456
CLU	head and neck cancer	neoplastic (JHU-O28, JHU-O22, JHU-O11, JHU-O29, OFK6/TERT1, FaDu, CAL 27)	1.23	iTRAQ	23665456
CNBP	head and neck cancer	neoplastic (JHU-O28, JHU-O22, JHU-O11, JHU-O29, OFK6/TERT1, FaDu, CAL 27)	-1	iTRAQ	23665456
CNDP2	head and neck cancer	neoplastic (JHU-O28, JHU-O22, JHU-O11, JHU-O29, OFK6/TERT1, FaDu, CAL 27)	-1.12	iTRAQ	23665456
CNN1	head and neck cancer	neoplastic (JHU-O28, JHU-O22, JHU-O11, JHU-O29, OFK6/TERT1, FaDu, CAL 27)	-2	iTRAQ	23665456
CNTN1	head and neck cancer	neoplastic (JHU-O28, JHU-O22, JHU-O11, JHU-O29, OFK6/TERT1, FaDu, CAL 27)	-1.47	iTRAQ	23665456
COL12A1	head and neck cancer	neoplastic (JHU-O28, JHU-O22, JHU-O11, JHU-O29, OFK6/TERT1, FaDu, CAL 27)	1.85	iTRAQ	23665456
COL18A1	head and neck cancer	neoplastic (JHU-O28, JHU-O22, JHU-O11, JHU-O29, OFK6/TERT1, FaDu, CAL 27)	2.54	iTRAQ	23665456
COL1A1	head and neck cancer	neoplastic (JHU-O28, JHU-O22, JHU-O11, JHU-O29, OFK6/TERT1, FaDu, CAL 27)	1.15	iTRAQ	23665456
COL4A2	head and neck cancer	neoplastic (JHU-O28, JHU-O22, JHU-O11, JHU-O29, OFK6/TERT1, FaDu, CAL 27)	1.58	iTRAQ	23665456
COL5A1	head and neck cancer	neoplastic (JHU-O28, JHU-O22, JHU-O11, JHU-O29, OFK6/TERT1, FaDu, CAL 27)	2.52	iTRAQ	23665456
COL6A2	head and neck cancer	neoplastic (JHU-O28, JHU-O22, JHU-O11, JHU-O29, OFK6/TERT1, FaDu, CAL 27)	1.10	iTRAQ	23665456
COPA	head and neck cancer	neoplastic (JHU-O28, JHU-O22, JHU-O11, JHU-O29, OFK6/TERT1, FaDu, CAL 27)	1.73	iTRAQ	23665456
COPG	head and neck cancer	neoplastic (JHU-O28, JHU-O22, JHU-O11, JHU-O29, OFK6/TERT1, FaDu, CAL 27)	1.20	iTRAQ	23665456
COPS2	head and neck cancer	neoplastic (JHU-O28, JHU-O22, JHU-O11, JHU-O29, OFK6/TERT1, FaDu, CAL 27)	1.11	iTRAQ	23665456
COPS8	head and neck cancer	neoplastic (JHU-O28, JHU-O22, JHU-O11, JHU-O29, OFK6/TERT1, FaDu, CAL 27)	1.70	iTRAQ	23665456
COPZ1	head and neck cancer	neoplastic (JHU-O28, JHU-O22, JHU-O11, JHU-O29, OFK6/TERT1, FaDu, CAL 27)	-1.05	iTRAQ	23665456
CORO1B	head and neck cancer	neoplastic (JHU-O28, JHU-O22, JHU-O11, JHU-O29, OFK6/TERT1, FaDu, CAL 27)	1.08	iTRAQ	23665456
CORO1C	head and neck cancer	neoplastic (JHU-O28, JHU-O22, JHU-O11, JHU-O29, OFK6/TERT1, FaDu, CAL 27)	1.17	iTRAQ	23665456
COX4I1	head and neck cancer	neoplastic (JHU-O28, JHU-O22, JHU-O11, JHU-O29, OFK6/TERT1, FaDu, CAL 27)	-1.43	iTRAQ	23665456
COX5B	head and neck cancer	neoplastic (JHU-O28, JHU-O22, JHU-O11, JHU-O29, OFK6/TERT1, FaDu, CAL 27)	-1.88	iTRAQ	23665456
CP	head and neck cancer	neoplastic (JHU-O28, JHU-O22, JHU-O11, JHU-O29, OFK6/TERT1, FaDu, CAL 27)	1.72	iTRAQ	23665456
CPD	head and neck cancer	neoplastic (JHU-O28, JHU-O22, JHU-O11, JHU-O29, OFK6/TERT1, FaDu, CAL 27)	-1.28	iTRAQ	23665456
CPNE1	head and neck cancer	neoplastic (JHU-O28, JHU-O22, JHU-O11, JHU-O29, OFK6/TERT1, FaDu, CAL 27)	1.58	iTRAQ	23665456
CPOX	head and neck cancer	neoplastic (JHU-O28, JHU-O22, JHU-O11, JHU-O29, OFK6/TERT1, FaDu, CAL 27)	-1.35	iTRAQ	23665456
CPSF1	head and neck cancer	neoplastic (JHU-O28, JHU-O22, JHU-O11, JHU-O29, OFK6/TERT1, FaDu, CAL 27)	1.13	iTRAQ	23665456
CPVL	head and neck cancer	neoplastic (JHU-O28, JHU-O22, JHU-O11, JHU-O29, OFK6/TERT1, FaDu, CAL 27)	2.59	iTRAQ	23665456
CRABP2	head and neck cancer	neoplastic (JHU-O28, JHU-O22, JHU-O11, JHU-O29, OFK6/TERT1, FaDu, CAL 27)	-1.02	iTRAQ	23665456
CRIM1	head and neck cancer	neoplastic (JHU-O28, JHU-O22, JHU-O11, JHU-O29, OFK6/TERT1, FaDu, CAL 27)	-1	iTRAQ	23665456
CRIP2	head and neck cancer	neoplastic (JHU-O28, JHU-O22, JHU-O11, JHU-O29, OFK6/TERT1, FaDu, CAL 27)	-1.51	iTRAQ	23665456
CST3	head and neck cancer	neoplastic (JHU-O28, JHU-O22, JHU-O11, JHU-O29, OFK6/TERT1, FaDu, CAL 27)	7.24	iTRAQ	23665456
CSTB	head and neck cancer	neoplastic (JHU-O28, JHU-O22, JHU-O11, JHU-O29, OFK6/TERT1, FaDu, CAL 27)	-1.55	iTRAQ	23665456
CTNNA1	head and neck cancer	neoplastic (JHU-O28, JHU-O22, JHU-O11, JHU-O29, OFK6/TERT1, FaDu, CAL 27)	2.50	iTRAQ	23665456
CTNNA2	head and neck cancer	neoplastic (JHU-O28, JHU-O22, JHU-O11, JHU-O29, OFK6/TERT1, FaDu, CAL 27)	1.33	iTRAQ	23665456
CTNNB1	head and neck cancer	neoplastic (JHU-O28, JHU-O22, JHU-O11, JHU-O29, OFK6/TERT1, FaDu, CAL 27)	1.72	iTRAQ	23665456
CTSA	head and neck cancer	neoplastic (JHU-O28, JHU-O22, JHU-O11, JHU-O29, OFK6/TERT1, FaDu, CAL 27)	1.09	iTRAQ	23665456

CTSD	head and neck cancer	neoplastic (JHU-O28, JHU-O22, JHU-O11, JHU-O29, OFK6/TERT1, FaDu, CAL 27)	2.96	iTRAQ	23665456
CTSL1	head and neck cancer	neoplastic (JHU-O28, JHU-O22, JHU-O11, JHU-O29, OFK6/TERT1, FaDu, CAL 27)	1.26	iTRAQ	23665456
CTSL2	head and neck cancer	neoplastic (JHU-O28, JHU-O22, JHU-O11, JHU-O29, OFK6/TERT1, FaDu, CAL 27)	2.35	iTRAQ	23665456
CTSS	head and neck cancer	neoplastic (JHU-O28, JHU-O22, JHU-O11, JHU-O29, OFK6/TERT1, FaDu, CAL 27)	1.55	iTRAQ	23665456
CUL1	head and neck cancer	neoplastic (JHU-O28, JHU-O22, JHU-O11, JHU-O29, OFK6/TERT1, FaDu, CAL 27)	1.30	iTRAQ	23665456
CUTC	head and neck cancer	neoplastic (JHU-O28, JHU-O22, JHU-O11, JHU-O29, OFK6/TERT1, FaDu, CAL 27)	1.29	iTRAQ	23665456
CXCL1	head and neck cancer	neoplastic (JHU-O28, JHU-O22, JHU-O11, JHU-O29, OFK6/TERT1, FaDu, CAL 27)	2.01	iTRAQ	23665456
CYB5R2	head and neck cancer	neoplastic (JHU-O28, JHU-O22, JHU-O11, JHU-O29, OFK6/TERT1, FaDu, CAL 27)	-2.47	iTRAQ	23665456
CYCS	head and neck cancer	neoplastic (JHU-O28, JHU-O22, JHU-O11, JHU-O29, OFK6/TERT1, FaDu, CAL 27)	-1.73	iTRAQ	23665456
DARS	head and neck cancer	neoplastic (JHU-O28, JHU-O22, JHU-O11, JHU-O29, OFK6/TERT1, FaDu, CAL 27)	1.07	iTRAQ	23665456
DBI	head and neck cancer	neoplastic (JHU-O28, JHU-O22, JHU-O11, JHU-O29, OFK6/TERT1, FaDu, CAL 27)	-1.39	iTRAQ	23665456
DBN1	head and neck cancer	neoplastic (JHU-O28, JHU-O22, JHU-O11, JHU-O29, OFK6/TERT1, FaDu, CAL 27)	-2	iTRAQ	23665456
DCBLD2	head and neck cancer	neoplastic (JHU-O28, JHU-O22, JHU-O11, JHU-O29, OFK6/TERT1, FaDu, CAL 27)	-1.15	iTRAQ	23665456
DCPS	head and neck cancer	neoplastic (JHU-O28, JHU-O22, JHU-O11, JHU-O29, OFK6/TERT1, FaDu, CAL 27)	-1.51	iTRAQ	23665456
DCTD	head and neck cancer	neoplastic (JHU-O28, JHU-O22, JHU-O11, JHU-O29, OFK6/TERT1, FaDu, CAL 27)	-1.64	iTRAQ	23665456
DCUN1D1	head and neck cancer	neoplastic (JHU-O28, JHU-O22, JHU-O11, JHU-O29, OFK6/TERT1, FaDu, CAL 27)	-1.15	iTRAQ	23665456
DDR1	head and neck cancer	neoplastic (JHU-O28, JHU-O22, JHU-O11, JHU-O29, OFK6/TERT1, FaDu, CAL 27)	-1.51	iTRAQ	23665456
DDT	head and neck cancer	neoplastic (JHU-O28, JHU-O22, JHU-O11, JHU-O29, OFK6/TERT1, FaDu, CAL 27)	-1.15	iTRAQ	23665456
DDX17	head and neck cancer	neoplastic (JHU-O28, JHU-O22, JHU-O11, JHU-O29, OFK6/TERT1, FaDu, CAL 27)	1.22	iTRAQ	23665456
DDX21	head and neck cancer	neoplastic (JHU-O28, JHU-O22, JHU-O11, JHU-O29, OFK6/TERT1, FaDu, CAL 27)	1.32	iTRAQ	23665456
DDX24	head and neck cancer	neoplastic (JHU-O28, JHU-O22, JHU-O11, JHU-O29, OFK6/TERT1, FaDu, CAL 27)	1.73	iTRAQ	23665456
DDX3X	head and neck cancer	neoplastic (JHU-O28, JHU-O22, JHU-O11, JHU-O29, OFK6/TERT1, FaDu, CAL 27)	1.59	iTRAQ	23665456
DDX42	head and neck cancer	neoplastic (JHU-O28, JHU-O22, JHU-O11, JHU-O29, OFK6/TERT1, FaDu, CAL 27)	2.03	iTRAQ	23665456
DDX6	head and neck cancer	neoplastic (JHU-O28, JHU-O22, JHU-O11, JHU-O29, OFK6/TERT1, FaDu, CAL 27)	1.35	iTRAQ	23665456
DEK	head and neck cancer	neoplastic (JHU-O28, JHU-O22, JHU-O11, JHU-O29, OFK6/TERT1, FaDu, CAL 27)	2.37	iTRAQ	23665456
DERA	head and neck cancer	neoplastic (JHU-O28, JHU-O22, JHU-O11, JHU-O29, OFK6/TERT1, FaDu, CAL 27)	1.85	iTRAQ	23665456
DHX9	head and neck cancer	neoplastic (JHU-O28, JHU-O22, JHU-O11, JHU-O29, OFK6/TERT1, FaDu, CAL 27)	1.07	iTRAQ	23665456
DKK3	head and neck cancer	neoplastic (JHU-O28, JHU-O22, JHU-O11, JHU-O29, OFK6/TERT1, FaDu, CAL 27)	-1.51	iTRAQ	23665456
DLGAP4	head and neck cancer	neoplastic (JHU-O28, JHU-O22, JHU-O11, JHU-O29, OFK6/TERT1, FaDu, CAL 27)	-2.12	iTRAQ	23665456
DMKN	head and neck cancer	neoplastic (JHU-O28, JHU-O22, JHU-O11, JHU-O29, OFK6/TERT1, FaDu, CAL 27)	-2.18	iTRAQ	23665456
DNAH17	head and neck cancer	neoplastic (JHU-O28, JHU-O22, JHU-O11, JHU-O29, OFK6/TERT1, FaDu, CAL 27)	-2.94	iTRAQ	23665456
DNAJB11	head and neck cancer	neoplastic (JHU-O28, JHU-O22, JHU-O11, JHU-O29, OFK6/TERT1, FaDu, CAL 27)	1.31	iTRAQ	23665456
DPP3	head and neck cancer	neoplastic (JHU-O28, JHU-O22, JHU-O11, JHU-O29, OFK6/TERT1, FaDu, CAL 27)	-1.12	iTRAQ	23665456
DPY30	head and neck cancer	neoplastic (JHU-O28, JHU-O22, JHU-O11, JHU-O29, OFK6/TERT1, FaDu, CAL 27)	-1.12	iTRAQ	23665456
DPYSL2	head and neck cancer	neoplastic (JHU-O28, JHU-O22, JHU-O11, JHU-O29, OFK6/TERT1, FaDu, CAL 27)	-1.68	iTRAQ	23665456
DSG2	head and neck cancer	neoplastic (JHU-O28, JHU-O22, JHU-O11, JHU-O29, OFK6/TERT1, FaDu, CAL 27)	4.79	iTRAQ	23665456
DSTN	head and neck cancer	neoplastic (JHU-O28, JHU-O22, JHU-O11, JHU-O29, OFK6/TERT1, FaDu, CAL 27)	-1.21	iTRAQ	23665456
DYNC1H1	head and neck cancer	neoplastic (JHU-O28, JHU-O22, JHU-O11, JHU-O29, OFK6/TERT1, FaDu, CAL 27)	1.59	iTRAQ	23665456
ECM1	head and neck cancer	neoplastic (JHU-O28, JHU-O22, JHU-O11, JHU-O29, OFK6/TERT1, FaDu, CAL 27)	-1.51	iTRAQ	23665456
EEF1A1	head and neck cancer	neoplastic (JHU-O28, JHU-O22, JHU-O11, JHU-O29, OFK6/TERT1, FaDu, CAL 27)	1.04	iTRAQ	23665456
EEF1B2	head and neck cancer	neoplastic (JHU-O28, JHU-O22, JHU-O11, JHU-O29, OFK6/TERT1, FaDu, CAL 27)	1.89	iTRAQ	23665456
EEF1D	head and neck cancer	neoplastic (JHU-O28, JHU-O22, JHU-O11, JHU-O29, OFK6/TERT1, FaDu, CAL 27)	1.98	iTRAQ	23665456
EEF1D	head and neck cancer	neoplastic (JHU-O28, JHU-O22, JHU-O11, JHU-O29, OFK6/TERT1, FaDu, CAL 27)	2.25	iTRAQ	23665456

EEF1G	head and neck cancer	neoplastic (JHU-O28, JHU-O22, JHU-O11, JHU-O29, OFK6/TERT1, FaDu, CAL 27)	1.20	iTRAQ	23665456
EFNB1	head and neck cancer	neoplastic (JHU-O28, JHU-O22, JHU-O11, JHU-O29, OFK6/TERT1, FaDu, CAL 27)	-2	iTRAQ	23665456
EFNB2	head and neck cancer	neoplastic (JHU-O28, JHU-O22, JHU-O11, JHU-O29, OFK6/TERT1, FaDu, CAL 27)	-1.78	iTRAQ	23665456
EFTUD2	head and neck cancer	neoplastic (JHU-O28, JHU-O22, JHU-O11, JHU-O29, OFK6/TERT1, FaDu, CAL 27)	1.64	iTRAQ	23665456
EIF1AD	head and neck cancer	neoplastic (JHU-O28, JHU-O22, JHU-O11, JHU-O29, OFK6/TERT1, FaDu, CAL 27)	1.02	iTRAQ	23665456
EIF1AY	head and neck cancer	neoplastic (JHU-O28, JHU-O22, JHU-O11, JHU-O29, OFK6/TERT1, FaDu, CAL 27)	1.48	iTRAQ	23665456
EIF1B	head and neck cancer	neoplastic (JHU-O28, JHU-O22, JHU-O11, JHU-O29, OFK6/TERT1, FaDu, CAL 27)	-1.18	iTRAQ	23665456
EIF2S1	head and neck cancer	neoplastic (JHU-O28, JHU-O22, JHU-O11, JHU-O29, OFK6/TERT1, FaDu, CAL 27)	1.63	iTRAQ	23665456
EIF2S2	head and neck cancer	neoplastic (JHU-O28, JHU-O22, JHU-O11, JHU-O29, OFK6/TERT1, FaDu, CAL 27)	2.01	iTRAQ	23665456
EIF3A	head and neck cancer	neoplastic (JHU-O28, JHU-O22, JHU-O11, JHU-O29, OFK6/TERT1, FaDu, CAL 27)	1.50	iTRAQ	23665456
EIF3B	head and neck cancer	neoplastic (JHU-O28, JHU-O22, JHU-O11, JHU-O29, OFK6/TERT1, FaDu, CAL 27)	1.91	iTRAQ	23665456
EIF3C	head and neck cancer	neoplastic (JHU-O28, JHU-O22, JHU-O11, JHU-O29, OFK6/TERT1, FaDu, CAL 27)	1.60	iTRAQ	23665456
EIF3D	head and neck cancer	neoplastic (JHU-O28, JHU-O22, JHU-O11, JHU-O29, OFK6/TERT1, FaDu, CAL 27)	1.78	iTRAQ	23665456
EIF3E	head and neck cancer	neoplastic (JHU-O28, JHU-O22, JHU-O11, JHU-O29, OFK6/TERT1, FaDu, CAL 27)	1.38	iTRAQ	23665456
EIF3I	head and neck cancer	neoplastic (JHU-O28, JHU-O22, JHU-O11, JHU-O29, OFK6/TERT1, FaDu, CAL 27)	1.20	iTRAQ	23665456
EIF3J	head and neck cancer	neoplastic (JHU-O28, JHU-O22, JHU-O11, JHU-O29, OFK6/TERT1, FaDu, CAL 27)	1.13	iTRAQ	23665456
EIF3K	head and neck cancer	neoplastic (JHU-O28, JHU-O22, JHU-O11, JHU-O29, OFK6/TERT1, FaDu, CAL 27)	1.07	iTRAQ	23665456
EIF3L	head and neck cancer	neoplastic (JHU-O28, JHU-O22, JHU-O11, JHU-O29, OFK6/TERT1, FaDu, CAL 27)	1.70	iTRAQ	23665456
EIF3M	head and neck cancer	neoplastic (JHU-O28, JHU-O22, JHU-O11, JHU-O29, OFK6/TERT1, FaDu, CAL 27)	1.61	iTRAQ	23665456
EIF4A1	head and neck cancer	neoplastic (JHU-O28, JHU-O22, JHU-O11, JHU-O29, OFK6/TERT1, FaDu, CAL 27)	1.21	iTRAQ	23665456
EIF4A3	head and neck cancer	neoplastic (JHU-O28, JHU-O22, JHU-O11, JHU-O29, OFK6/TERT1, FaDu, CAL 27)	2.27	iTRAQ	23665456
EIF4E	head and neck cancer	neoplastic (JHU-O28, JHU-O22, JHU-O11, JHU-O29, OFK6/TERT1, FaDu, CAL 27)	-1.02	iTRAQ	23665456
EIF4G3	head and neck cancer	neoplastic (JHU-O28, JHU-O22, JHU-O11, JHU-O29, OFK6/TERT1, FaDu, CAL 27)	-1.12	iTRAQ	23665456
EIF5	head and neck cancer	neoplastic (JHU-O28, JHU-O22, JHU-O11, JHU-O29, OFK6/TERT1, FaDu, CAL 27)	3.12	iTRAQ	23665456
EIF5B	head and neck cancer	neoplastic (JHU-O28, JHU-O22, JHU-O11, JHU-O29, OFK6/TERT1, FaDu, CAL 27)	2.84	iTRAQ	23665456
EMD	head and neck cancer	neoplastic (JHU-O28, JHU-O22, JHU-O11, JHU-O29, OFK6/TERT1, FaDu, CAL 27)	-1.08	iTRAQ	23665456
EML2	head and neck cancer	neoplastic (JHU-O28, JHU-O22, JHU-O11, JHU-O29, OFK6/TERT1, FaDu, CAL 27)	-1.59	iTRAQ	23665456
ENO1	head and neck cancer	neoplastic (JHU-O28, JHU-O22, JHU-O11, JHU-O29, OFK6/TERT1, FaDu, CAL 27)	1.20	iTRAQ	23665456
ENO1	head and neck cancer	neoplastic (JHU-O28, JHU-O22, JHU-O11, JHU-O29, OFK6/TERT1, FaDu, CAL 27)	-1.47	iTRAQ	23665456
ENO2	head and neck cancer	neoplastic (JHU-O28, JHU-O22, JHU-O11, JHU-O29, OFK6/TERT1, FaDu, CAL 27)	-1.28	iTRAQ	23665456
ENOPH1	head and neck cancer	neoplastic (JHU-O28, JHU-O22, JHU-O11, JHU-O29, OFK6/TERT1, FaDu, CAL 27)	-1.05	iTRAQ	23665456
EP400	head and neck cancer	neoplastic (JHU-O28, JHU-O22, JHU-O11, JHU-O29, OFK6/TERT1, FaDu, CAL 27)	5.19	iTRAQ	23665456
EPB41L1	head and neck cancer	neoplastic (JHU-O28, JHU-O22, JHU-O11, JHU-O29, OFK6/TERT1, FaDu, CAL 27)	3.56	iTRAQ	23665456
EPHA5	head and neck cancer	neoplastic (JHU-O28, JHU-O22, JHU-O11, JHU-O29, OFK6/TERT1, FaDu, CAL 27)	2.42	iTRAQ	23665456
EPHB4	head and neck cancer	neoplastic (JHU-O28, JHU-O22, JHU-O11, JHU-O29, OFK6/TERT1, FaDu, CAL 27)	-1.55	iTRAQ	23665456
EPN3	head and neck cancer	neoplastic (JHU-O28, JHU-O22, JHU-O11, JHU-O29, OFK6/TERT1, FaDu, CAL 27)	-1.47	iTRAQ	23665456
ERAP2	head and neck cancer	neoplastic (JHU-O28, JHU-O22, JHU-O11, JHU-O29, OFK6/TERT1, FaDu, CAL 27)	-1.35	iTRAQ	23665456
ERO1L	head and neck cancer	neoplastic (JHU-O28, JHU-O22, JHU-O11, JHU-O29, OFK6/TERT1, FaDu, CAL 27)	-1.43	iTRAQ	23665456
ERP44	head and neck cancer	neoplastic (JHU-O28, JHU-O22, JHU-O11, JHU-O29, OFK6/TERT1, FaDu, CAL 27)	-1.28	iTRAQ	23665456
ESYT1	head and neck cancer	neoplastic (JHU-O28, JHU-O22, JHU-O11, JHU-O29, OFK6/TERT1, FaDu, CAL 27)	-1.78	iTRAQ	23665456
ETHE1	head and neck cancer	neoplastic (JHU-O28, JHU-O22, JHU-O11, JHU-O29, OFK6/TERT1, FaDu, CAL 27)	-2.25	iTRAQ	23665456
EVPL	head and neck cancer	neoplastic (JHU-O28, JHU-O22, JHU-O11, JHU-O29, OFK6/TERT1, FaDu, CAL 27)	-2.55	iTRAQ	23665456
EZR	head and neck cancer	neoplastic (JHU-O28, JHU-O22, JHU-O11, JHU-O29, OFK6/TERT1, FaDu, CAL 27)	1.32	iTRAQ	23665456

F3	head and neck cancer	neoplastic (JHU-O28, JHU-O22, JHU-O11, JHU-O29, OFK6/TERT1, FaDu, CAL 27)	2.52	iTRAQ	23665456
FABP5	head and neck cancer	neoplastic (JHU-O28, JHU-O22, JHU-O11, JHU-O29, OFK6/TERT1, FaDu, CAL 27)	-1.64	iTRAQ	23665456
FAH	head and neck cancer	neoplastic (JHU-O28, JHU-O22, JHU-O11, JHU-O29, OFK6/TERT1, FaDu, CAL 27)	-1.73	iTRAQ	23665456
FAHD2A	head and neck cancer	neoplastic (JHU-O28, JHU-O22, JHU-O11, JHU-O29, OFK6/TERT1, FaDu, CAL 27)	-1.12	iTRAQ	23665456
FAM120A	head and neck cancer	neoplastic (JHU-O28, JHU-O22, JHU-O11, JHU-O29, OFK6/TERT1, FaDu, CAL 27)	1.13	iTRAQ	23665456
FAM20C	head and neck cancer	neoplastic (JHU-O28, JHU-O22, JHU-O11, JHU-O29, OFK6/TERT1, FaDu, CAL 27)	1.35	iTRAQ	23665456
FAM45A	head and neck cancer	neoplastic (JHU-O28, JHU-O22, JHU-O11, JHU-O29, OFK6/TERT1, FaDu, CAL 27)	-1.18	iTRAQ	23665456
FAM50B	head and neck cancer	neoplastic (JHU-O28, JHU-O22, JHU-O11, JHU-O29, OFK6/TERT1, FaDu, CAL 27)	1.74	iTRAQ	23665456
FAM82A2	head and neck cancer	neoplastic (JHU-O28, JHU-O22, JHU-O11, JHU-O29, OFK6/TERT1, FaDu, CAL 27)	1.20	iTRAQ	23665456
FAM82B	head and neck cancer	neoplastic (JHU-O28, JHU-O22, JHU-O11, JHU-O29, OFK6/TERT1, FaDu, CAL 27)	-1.25	iTRAQ	23665456
FAM96B	head and neck cancer	neoplastic (JHU-O28, JHU-O22, JHU-O11, JHU-O29, OFK6/TERT1, FaDu, CAL 27)	-3.32	iTRAQ	23665456
FARSB	head and neck cancer	neoplastic (JHU-O28, JHU-O22, JHU-O11, JHU-O29, OFK6/TERT1, FaDu, CAL 27)	1.22	iTRAQ	23665456
FAS	head and neck cancer	neoplastic (JHU-O28, JHU-O22, JHU-O11, JHU-O29, OFK6/TERT1, FaDu, CAL 27)	-1.51	iTRAQ	23665456
FBL	head and neck cancer	neoplastic (JHU-O28, JHU-O22, JHU-O11, JHU-O29, OFK6/TERT1, FaDu, CAL 27)	1.12	iTRAQ	23665456
FBLN1	head and neck cancer	neoplastic (JHU-O28, JHU-O22, JHU-O11, JHU-O29, OFK6/TERT1, FaDu, CAL 27)	2.49	iTRAQ	23665456
FBLN1	head and neck cancer	neoplastic (JHU-O28, JHU-O22, JHU-O11, JHU-O29, OFK6/TERT1, FaDu, CAL 27)	2.67	iTRAQ	23665456
FDPS	head and neck cancer	neoplastic (JHU-O28, JHU-O22, JHU-O11, JHU-O29, OFK6/TERT1, FaDu, CAL 27)	-1.73	iTRAQ	23665456
FDPS	head and neck cancer	neoplastic (JHU-O28, JHU-O22, JHU-O11, JHU-O29, OFK6/TERT1, FaDu, CAL 27)	-1.88	iTRAQ	23665456
FH	head and neck cancer	neoplastic (JHU-O28, JHU-O22, JHU-O11, JHU-O29, OFK6/TERT1, FaDu, CAL 27)	-2.05	iTRAQ	23665456
FHIT	head and neck cancer	neoplastic (JHU-O28, JHU-O22, JHU-O11, JHU-O29, OFK6/TERT1, FaDu, CAL 27)	-2.64	iTRAQ	23665456
FHL1	head and neck cancer	neoplastic (JHU-O28, JHU-O22, JHU-O11, JHU-O29, OFK6/TERT1, FaDu, CAL 27)	-1.39	iTRAQ	23665456
FKBP1A	head and neck cancer	neoplastic (JHU-O28, JHU-O22, JHU-O11, JHU-O29, OFK6/TERT1, FaDu, CAL 27)	-1.32	iTRAQ	23665456
FKBP1B	head and neck cancer	neoplastic (JHU-O28, JHU-O22, JHU-O11, JHU-O29, OFK6/TERT1, FaDu, CAL 27)	-2.12	iTRAQ	23665456
FKBP2	head and neck cancer	neoplastic (JHU-O28, JHU-O22, JHU-O11, JHU-O29, OFK6/TERT1, FaDu, CAL 27)	-1.43	iTRAQ	23665456
FLJ43806	head and neck cancer	neoplastic (JHU-O28, JHU-O22, JHU-O11, JHU-O29, OFK6/TERT1, FaDu, CAL 27)	-1	iTRAQ	23665456
FLNA	head and neck cancer	neoplastic (JHU-O28, JHU-O22, JHU-O11, JHU-O29, OFK6/TERT1, FaDu, CAL 27)	1.76	iTRAQ	23665456
FLNB	head and neck cancer	neoplastic (JHU-O28, JHU-O22, JHU-O11, JHU-O29, OFK6/TERT1, FaDu, CAL 27)	-1.05	iTRAQ	23665456
FLRT2	head and neck cancer	neoplastic (JHU-O28, JHU-O22, JHU-O11, JHU-O29, OFK6/TERT1, FaDu, CAL 27)	-1.12	iTRAQ	23665456
FLRT3	head and neck cancer	neoplastic (JHU-O28, JHU-O22, JHU-O11, JHU-O29, OFK6/TERT1, FaDu, CAL 27)	-1.64	iTRAQ	23665456
FLYWCH2	head and neck cancer	neoplastic (JHU-O28, JHU-O22, JHU-O11, JHU-O29, OFK6/TERT1, FaDu, CAL 27)	-1.05	iTRAQ	23665456
FN1	head and neck cancer	neoplastic (JHU-O28, JHU-O22, JHU-O11, JHU-O29, OFK6/TERT1, FaDu, CAL 27)	-1.08	iTRAQ	23665456
FRMPD1	head and neck cancer	neoplastic (JHU-O28, JHU-O22, JHU-O11, JHU-O29, OFK6/TERT1, FaDu, CAL 27)	3.33	iTRAQ	23665456
FSCN1	head and neck cancer	neoplastic (JHU-O28, JHU-O22, JHU-O11, JHU-O29, OFK6/TERT1, FaDu, CAL 27)	-1.05	iTRAQ	23665456
FST	head and neck cancer	neoplastic (JHU-O28, JHU-O22, JHU-O11, JHU-O29, OFK6/TERT1, FaDu, CAL 27)	2.01	iTRAQ	23665456
FSTL1	head and neck cancer	neoplastic (JHU-O28, JHU-O22, JHU-O11, JHU-O29, OFK6/TERT1, FaDu, CAL 27)	1.73	iTRAQ	23665456
FTH1	head and neck cancer	neoplastic (JHU-O28, JHU-O22, JHU-O11, JHU-O29, OFK6/TERT1, FaDu, CAL 27)	-1.73	iTRAQ	23665456
FUCA2	head and neck cancer	neoplastic (JHU-O28, JHU-O22, JHU-O11, JHU-O29, OFK6/TERT1, FaDu, CAL 27)	1.74	iTRAQ	23665456
FUT11	head and neck cancer	neoplastic (JHU-O28, JHU-O22, JHU-O11, JHU-O29, OFK6/TERT1, FaDu, CAL 27)	-2.64	iTRAQ	23665456
G3BP2	head and neck cancer	neoplastic (JHU-O28, JHU-O22, JHU-O11, JHU-O29, OFK6/TERT1, FaDu, CAL 27)	1.35	iTRAQ	23665456
GABARAP	head and neck cancer	neoplastic (JHU-O28, JHU-O22, JHU-O11, JHU-O29, OFK6/TERT1, FaDu, CAL 27)	-1.05	iTRAQ	23665456
GALE	head and neck cancer	neoplastic (JHU-O28, JHU-O22, JHU-O11, JHU-O29, OFK6/TERT1, FaDu, CAL 27)	-1.51	iTRAQ	23665456
GALNS	head and neck cancer	neoplastic (JHU-O28, JHU-O22, JHU-O11, JHU-O29, OFK6/TERT1, FaDu, CAL 27)	1.85	iTRAQ	23665456
GALNT5	head and neck cancer	neoplastic (JHU-O28, JHU-O22, JHU-O11, JHU-O29, OFK6/TERT1, FaDu, CAL 27)	-1.68	iTRAQ	23665456

GALT	head and neck cancer	neoplastic (JHU-O28, JHU-O22, JHU-O11, JHU-O29, OFK6/TERT1, FaDu, CAL 27)	-1.21	iTRAQ	23665456
GAMT	head and neck cancer	neoplastic (JHU-O28, JHU-O22, JHU-O11, JHU-O29, OFK6/TERT1, FaDu, CAL 27)	-1.73	iTRAQ	23665456
GAPDH	head and neck cancer	neoplastic (JHU-O28, JHU-O22, JHU-O11, JHU-O29, OFK6/TERT1, FaDu, CAL 27)	1.76	iTRAQ	23665456
GAPDH	head and neck cancer	neoplastic (JHU-O28, JHU-O22, JHU-O11, JHU-O29, OFK6/TERT1, FaDu, CAL 27)	2.51	iTRAQ	23665456
GAS6	head and neck cancer	neoplastic (JHU-O28, JHU-O22, JHU-O11, JHU-O29, OFK6/TERT1, FaDu, CAL 27)	1.60	iTRAQ	23665456
GC	head and neck cancer	neoplastic (JHU-O28, JHU-O22, JHU-O11, JHU-O29, OFK6/TERT1, FaDu, CAL 27)	1.58	iTRAQ	23665456
GCLC	head and neck cancer	neoplastic (JHU-O28, JHU-O22, JHU-O11, JHU-O29, OFK6/TERT1, FaDu, CAL 27)	-1.35	iTRAQ	23665456
GDI2	head and neck cancer	neoplastic (JHU-O28, JHU-O22, JHU-O11, JHU-O29, OFK6/TERT1, FaDu, CAL 27)	1.84	iTRAQ	23665456
GGH	head and neck cancer	neoplastic (JHU-O28, JHU-O22, JHU-O11, JHU-O29, OFK6/TERT1, FaDu, CAL 27)	1.65	iTRAQ	23665456
GLO1	head and neck cancer	neoplastic (JHU-O28, JHU-O22, JHU-O11, JHU-O29, OFK6/TERT1, FaDu, CAL 27)	-1.55	iTRAQ	23665456
GLOD4	head and neck cancer	neoplastic (JHU-O28, JHU-O22, JHU-O11, JHU-O29, OFK6/TERT1, FaDu, CAL 27)	-1.39	iTRAQ	23665456
GLRX	head and neck cancer	neoplastic (JHU-O28, JHU-O22, JHU-O11, JHU-O29, OFK6/TERT1, FaDu, CAL 27)	-1.64	iTRAQ	23665456
GLRX5	head and neck cancer	neoplastic (JHU-O28, JHU-O22, JHU-O11, JHU-O29, OFK6/TERT1, FaDu, CAL 27)	-1.59	iTRAQ	23665456
GLT25D1	head and neck cancer	neoplastic (JHU-O28, JHU-O22, JHU-O11, JHU-O29, OFK6/TERT1, FaDu, CAL 27)	-1.05	iTRAQ	23665456
GLUD1	head and neck cancer	neoplastic (JHU-O28, JHU-O22, JHU-O11, JHU-O29, OFK6/TERT1, FaDu, CAL 27)	1.59	iTRAQ	23665456
GNA13	head and neck cancer	neoplastic (JHU-O28, JHU-O22, JHU-O11, JHU-O29, OFK6/TERT1, FaDu, CAL 27)	1.69	iTRAQ	23665456
GOLM1	head and neck cancer	neoplastic (JHU-O28, JHU-O22, JHU-O11, JHU-O29, OFK6/TERT1, FaDu, CAL 27)	1.58	iTRAQ	23665456
GOT2	head and neck cancer	neoplastic (JHU-O28, JHU-O22, JHU-O11, JHU-O29, OFK6/TERT1, FaDu, CAL 27)	-1.21	iTRAQ	23665456
GOT2	head and neck cancer	neoplastic (JHU-O28, JHU-O22, JHU-O11, JHU-O29, OFK6/TERT1, FaDu, CAL 27)	-1.32	iTRAQ	23665456
GPC1	head and neck cancer	neoplastic (JHU-O28, JHU-O22, JHU-O11, JHU-O29, OFK6/TERT1, FaDu, CAL 27)	1.14	iTRAQ	23665456
GPC1	head and neck cancer	neoplastic (JHU-O28, JHU-O22, JHU-O11, JHU-O29, OFK6/TERT1, FaDu, CAL 27)	4.67	iTRAQ	23665456
GPI	head and neck cancer	neoplastic (JHU-O28, JHU-O22, JHU-O11, JHU-O29, OFK6/TERT1, FaDu, CAL 27)	-1	iTRAQ	23665456
GPR125	head and neck cancer	neoplastic (JHU-O28, JHU-O22, JHU-O11, JHU-O29, OFK6/TERT1, FaDu, CAL 27)	1.05	iTRAQ	23665456
GSPT1	head and neck cancer	neoplastic (JHU-O28, JHU-O22, JHU-O11, JHU-O29, OFK6/TERT1, FaDu, CAL 27)	1.55	iTRAQ	23665456
GSTM1	head and neck cancer	neoplastic (JHU-O28, JHU-O22, JHU-O11, JHU-O29, OFK6/TERT1, FaDu, CAL 27)	-2.83	iTRAQ	23665456
GSTO1	head and neck cancer	neoplastic (JHU-O28, JHU-O22, JHU-O11, JHU-O29, OFK6/TERT1, FaDu, CAL 27)	-1	iTRAQ	23665456
GSTP1	head and neck cancer	neoplastic (JHU-O28, JHU-O22, JHU-O11, JHU-O29, OFK6/TERT1, FaDu, CAL 27)	1.92	iTRAQ	23665456
H2AFV	head and neck cancer	neoplastic (JHU-O28, JHU-O22, JHU-O11, JHU-O29, OFK6/TERT1, FaDu, CAL 27)	1.31	iTRAQ	23665456
H2AFX	head and neck cancer	neoplastic (JHU-O28, JHU-O22, JHU-O11, JHU-O29, OFK6/TERT1, FaDu, CAL 27)	-2.32	iTRAQ	23665456
H6PD	head and neck cancer	neoplastic (JHU-O28, JHU-O22, JHU-O11, JHU-O29, OFK6/TERT1, FaDu, CAL 27)	-1.43	iTRAQ	23665456
HADH	head and neck cancer	neoplastic (JHU-O28, JHU-O22, JHU-O11, JHU-O29, OFK6/TERT1, FaDu, CAL 27)	-1.47	iTRAQ	23665456
HARS	head and neck cancer	neoplastic (JHU-O28, JHU-O22, JHU-O11, JHU-O29, OFK6/TERT1, FaDu, CAL 27)	-1.32	iTRAQ	23665456
HAVCR1	head and neck cancer	neoplastic (JHU-O28, JHU-O22, JHU-O11, JHU-O29, OFK6/TERT1, FaDu, CAL 27)	2.11	iTRAQ	23665456
HBA2	head and neck cancer	neoplastic (JHU-O28, JHU-O22, JHU-O11, JHU-O29, OFK6/TERT1, FaDu, CAL 27)	-1.78	iTRAQ	23665456
HBB	head and neck cancer	neoplastic (JHU-O28, JHU-O22, JHU-O11, JHU-O29, OFK6/TERT1, FaDu, CAL 27)	1.53	iTRAQ	23665456
HBE1	head and neck cancer	neoplastic (JHU-O28, JHU-O22, JHU-O11, JHU-O29, OFK6/TERT1, FaDu, CAL 27)	2.43	iTRAQ	23665456
HBG1	head and neck cancer	neoplastic (JHU-O28, JHU-O22, JHU-O11, JHU-O29, OFK6/TERT1, FaDu, CAL 27)	-5.64	iTRAQ	23665456
HCLS1	head and neck cancer	neoplastic (JHU-O28, JHU-O22, JHU-O11, JHU-O29, OFK6/TERT1, FaDu, CAL 27)	-1.73	iTRAQ	23665456
HDAC1	head and neck cancer	neoplastic (JHU-O28, JHU-O22, JHU-O11, JHU-O29, OFK6/TERT1, FaDu, CAL 27)	1.62	iTRAQ	23665456
HDAC11	head and neck cancer	neoplastic (JHU-O28, JHU-O22, JHU-O11, JHU-O29, OFK6/TERT1, FaDu, CAL 27)	-1.39	iTRAQ	23665456
HDGF	head and neck cancer	neoplastic (JHU-O28, JHU-O22, JHU-O11, JHU-O29, OFK6/TERT1, FaDu, CAL 27)	2.15	iTRAQ	23665456
HDGF	head and neck cancer	neoplastic (JHU-O28, JHU-O22, JHU-O11, JHU-O29, OFK6/TERT1, FaDu, CAL 27)	4.68	iTRAQ	23665456
HDHD1	head and neck cancer	neoplastic (JHU-O28, JHU-O22, JHU-O11, JHU-O29, OFK6/TERT1, FaDu, CAL 27)	-2.47	iTRAQ	23665456

HEBP2	head and neck cancer	neoplastic (JHU-O28, JHU-O22, JHU-O11, JHU-O29, OFK6/TERT1, FaDu, CAL 27)	-1.94	iTRAQ	23665456
HGFAC	head and neck cancer	neoplastic (JHU-O28, JHU-O22, JHU-O11, JHU-O29, OFK6/TERT1, FaDu, CAL 27)	2.26	iTRAQ	23665456
HGS	head and neck cancer	neoplastic (JHU-O28, JHU-O22, JHU-O11, JHU-O29, OFK6/TERT1, FaDu, CAL 27)	-1.25	iTRAQ	23665456
HIBADH	head and neck cancer	neoplastic (JHU-O28, JHU-O22, JHU-O11, JHU-O29, OFK6/TERT1, FaDu, CAL 27)	-1.08	iTRAQ	23665456
HINT2	head and neck cancer	neoplastic (JHU-O28, JHU-O22, JHU-O11, JHU-O29, OFK6/TERT1, FaDu, CAL 27)	-1.18	iTRAQ	23665456
HIST1H1C	head and neck cancer	neoplastic (JHU-O28, JHU-O22, JHU-O11, JHU-O29, OFK6/TERT1, FaDu, CAL 27)	1.92	iTRAQ	23665456
HIST1H2BB	head and neck cancer	neoplastic (JHU-O28, JHU-O22, JHU-O11, JHU-O29, OFK6/TERT1, FaDu, CAL 27)	2.84	iTRAQ	23665456
HIST1H2BD	head and neck cancer	neoplastic (JHU-O28, JHU-O22, JHU-O11, JHU-O29, OFK6/TERT1, FaDu, CAL 27)	1.77	iTRAQ	23665456
HIST1H2BM	head and neck cancer	neoplastic (JHU-O28, JHU-O22, JHU-O11, JHU-O29, OFK6/TERT1, FaDu, CAL 27)	-1.73	iTRAQ	23665456
HIST1H4I	head and neck cancer	neoplastic (JHU-O28, JHU-O22, JHU-O11, JHU-O29, OFK6/TERT1, FaDu, CAL 27)	1.78	iTRAQ	23665456
HIST2H3C	head and neck cancer	neoplastic (JHU-O28, JHU-O22, JHU-O11, JHU-O29, OFK6/TERT1, FaDu, CAL 27)	1.19	iTRAQ	23665456
HK1	head and neck cancer	neoplastic (JHU-O28, JHU-O22, JHU-O11, JHU-O29, OFK6/TERT1, FaDu, CAL 27)	1.38	iTRAQ	23665456
HMGA1	head and neck cancer	neoplastic (JHU-O28, JHU-O22, JHU-O11, JHU-O29, OFK6/TERT1, FaDu, CAL 27)	2.22	iTRAQ	23665456
HMGB2	head and neck cancer	neoplastic (JHU-O28, JHU-O22, JHU-O11, JHU-O29, OFK6/TERT1, FaDu, CAL 27)	1.19	iTRAQ	23665456
HMGCS1	head and neck cancer	neoplastic (JHU-O28, JHU-O22, JHU-O11, JHU-O29, OFK6/TERT1, FaDu, CAL 27)	-1.25	iTRAQ	23665456
HMGN3	head and neck cancer	neoplastic (JHU-O28, JHU-O22, JHU-O11, JHU-O29, OFK6/TERT1, FaDu, CAL 27)	1.11	iTRAQ	23665456
HNRNPA3	head and neck cancer	neoplastic (JHU-O28, JHU-O22, JHU-O11, JHU-O29, OFK6/TERT1, FaDu, CAL 27)	1.18	iTRAQ	23665456
HNRNPAB	head and neck cancer	neoplastic (JHU-O28, JHU-O22, JHU-O11, JHU-O29, OFK6/TERT1, FaDu, CAL 27)	-1.32	iTRAQ	23665456
HNRNPC	head and neck cancer	neoplastic (JHU-O28, JHU-O22, JHU-O11, JHU-O29, OFK6/TERT1, FaDu, CAL 27)	1.65	iTRAQ	23665456
HNRNPR	head and neck cancer	neoplastic (JHU-O28, JHU-O22, JHU-O11, JHU-O29, OFK6/TERT1, FaDu, CAL 27)	1.41	iTRAQ	23665456
HNRNPU	head and neck cancer	neoplastic (JHU-O28, JHU-O22, JHU-O11, JHU-O29, OFK6/TERT1, FaDu, CAL 27)	1.86	iTRAQ	23665456
HNRNPUL2	head and neck cancer	neoplastic (JHU-O28, JHU-O22, JHU-O11, JHU-O29, OFK6/TERT1, FaDu, CAL 27)	1.51	iTRAQ	23665456
HP	head and neck cancer	neoplastic (JHU-O28, JHU-O22, JHU-O11, JHU-O29, OFK6/TERT1, FaDu, CAL 27)	-1.55	iTRAQ	23665456
HPX	head and neck cancer	neoplastic (JHU-O28, JHU-O22, JHU-O11, JHU-O29, OFK6/TERT1, FaDu, CAL 27)	-2.47	iTRAQ	23665456
HSBP1	head and neck cancer	neoplastic (JHU-O28, JHU-O22, JHU-O11, JHU-O29, OFK6/TERT1, FaDu, CAL 27)	-1.83	iTRAQ	23665456
HSP90AA1	head and neck cancer	neoplastic (JHU-O28, JHU-O22, JHU-O11, JHU-O29, OFK6/TERT1, FaDu, CAL 27)	1.96	iTRAQ	23665456
HSP90AA1	head and neck cancer	neoplastic (JHU-O28, JHU-O22, JHU-O11, JHU-O29, OFK6/TERT1, FaDu, CAL 27)	2.58	iTRAQ	23665456
HSP90AB1	head and neck cancer	neoplastic (JHU-O28, JHU-O22, JHU-O11, JHU-O29, OFK6/TERT1, FaDu, CAL 27)	2.48	iTRAQ	23665456
HSP90B1	head and neck cancer	neoplastic (JHU-O28, JHU-O22, JHU-O11, JHU-O29, OFK6/TERT1, FaDu, CAL 27)	1.67	iTRAQ	23665456
HSPA1A	head and neck cancer	neoplastic (JHU-O28, JHU-O22, JHU-O11, JHU-O29, OFK6/TERT1, FaDu, CAL 27)	4.27	iTRAQ	23665456
HSPA4	head and neck cancer	neoplastic (JHU-O28, JHU-O22, JHU-O11, JHU-O29, OFK6/TERT1, FaDu, CAL 27)	1.26	iTRAQ	23665456
HSPA5	head and neck cancer	neoplastic (JHU-O28, JHU-O22, JHU-O11, JHU-O29, OFK6/TERT1, FaDu, CAL 27)	1.37	iTRAQ	23665456
HSPD1	head and neck cancer	neoplastic (JHU-O28, JHU-O22, JHU-O11, JHU-O29, OFK6/TERT1, FaDu, CAL 27)	1.53	iTRAQ	23665456
HSPD1	head and neck cancer	neoplastic (JHU-O28, JHU-O22, JHU-O11, JHU-O29, OFK6/TERT1, FaDu, CAL 27)	-2.32	iTRAQ	23665456
HSPE1	head and neck cancer	neoplastic (JHU-O28, JHU-O22, JHU-O11, JHU-O29, OFK6/TERT1, FaDu, CAL 27)	-1.02	iTRAQ	23665456
HSPG2	head and neck cancer	neoplastic (JHU-O28, JHU-O22, JHU-O11, JHU-O29, OFK6/TERT1, FaDu, CAL 27)	1.09	iTRAQ	23665456
HSPH1	head and neck cancer	neoplastic (JHU-O28, JHU-O22, JHU-O11, JHU-O29, OFK6/TERT1, FaDu, CAL 27)	1.15	iTRAQ	23665456
HTRA2	head and neck cancer	neoplastic (JHU-O28, JHU-O22, JHU-O11, JHU-O29, OFK6/TERT1, FaDu, CAL 27)	-1.32	iTRAQ	23665456
HYI	head and neck cancer	neoplastic (JHU-O28, JHU-O22, JHU-O11, JHU-O29, OFK6/TERT1, FaDu, CAL 27)	-1.83	iTRAQ	23665456
IAH1	head and neck cancer	neoplastic (JHU-O28, JHU-O22, JHU-O11, JHU-O29, OFK6/TERT1, FaDu, CAL 27)	-1.64	iTRAQ	23665456
IARS	head and neck cancer	neoplastic (JHU-O28, JHU-O22, JHU-O11, JHU-O29, OFK6/TERT1, FaDu, CAL 27)	1.11	iTRAQ	23665456
ICAM5	head and neck cancer	neoplastic (JHU-O28, JHU-O22, JHU-O11, JHU-O29, OFK6/TERT1, FaDu, CAL 27)	-1.25	iTRAQ	23665456
IDH1	head and neck cancer	neoplastic (JHU-O28, JHU-O22, JHU-O11, JHU-O29, OFK6/TERT1, FaDu, CAL 27)	1.26	iTRAQ	23665456

IDI1	head and neck cancer	neoplastic (JHU-O28, JHU-O22, JHU-O11, JHU-O29, OFK6/TERT1, FaDu, CAL 27)	-1.47	iTRAQ	23665456
IFIT1B	head and neck cancer	neoplastic (JHU-O28, JHU-O22, JHU-O11, JHU-O29, OFK6/TERT1, FaDu, CAL 27)	1.61	iTRAQ	23665456
IGFBP2	head and neck cancer	neoplastic (JHU-O28, JHU-O22, JHU-O11, JHU-O29, OFK6/TERT1, FaDu, CAL 27)	1.44	iTRAQ	23665456
IGFBP3	head and neck cancer	neoplastic (JHU-O28, JHU-O22, JHU-O11, JHU-O29, OFK6/TERT1, FaDu, CAL 27)	3.53	iTRAQ	23665456
IGFBP4	head and neck cancer	neoplastic (JHU-O28, JHU-O22, JHU-O11, JHU-O29, OFK6/TERT1, FaDu, CAL 27)	1.25	iTRAQ	23665456
IGFBP7	head and neck cancer	neoplastic (JHU-O28, JHU-O22, JHU-O11, JHU-O29, OFK6/TERT1, FaDu, CAL 27)	-1.73	iTRAQ	23665456
IGFBP7	head and neck cancer	neoplastic (JHU-O28, JHU-O22, JHU-O11, JHU-O29, OFK6/TERT1, FaDu, CAL 27)	1.84	iTRAQ	23665456
IL1A	head and neck cancer	neoplastic (JHU-O28, JHU-O22, JHU-O11, JHU-O29, OFK6/TERT1, FaDu, CAL 27)	-2.12	iTRAQ	23665456
IL1F9	head and neck cancer	neoplastic (JHU-O28, JHU-O22, JHU-O11, JHU-O29, OFK6/TERT1, FaDu, CAL 27)	-1.25	iTRAQ	23665456
IL1RN	head and neck cancer	neoplastic (JHU-O28, JHU-O22, JHU-O11, JHU-O29, OFK6/TERT1, FaDu, CAL 27)	-2.05	iTRAQ	23665456
IL6	head and neck cancer	neoplastic (JHU-O28, JHU-O22, JHU-O11, JHU-O29, OFK6/TERT1, FaDu, CAL 27)	3.09	iTRAQ	23665456
IL8	head and neck cancer	neoplastic (JHU-O28, JHU-O22, JHU-O11, JHU-O29, OFK6/TERT1, FaDu, CAL 27)	1.35	iTRAQ	23665456
ILF3	head and neck cancer	neoplastic (JHU-O28, JHU-O22, JHU-O11, JHU-O29, OFK6/TERT1, FaDu, CAL 27)	1.26	iTRAQ	23665456
IMMT	head and neck cancer	neoplastic (JHU-O28, JHU-O22, JHU-O11, JHU-O29, OFK6/TERT1, FaDu, CAL 27)	-1.32	iTRAQ	23665456
INPP1	head and neck cancer	neoplastic (JHU-O28, JHU-O22, JHU-O11, JHU-O29, OFK6/TERT1, FaDu, CAL 27)	-1.83	iTRAQ	23665456
INS	head and neck cancer	neoplastic (JHU-O28, JHU-O22, JHU-O11, JHU-O29, OFK6/TERT1, FaDu, CAL 27)	-2.39	iTRAQ	23665456
IPO7	head and neck cancer	neoplastic (JHU-O28, JHU-O22, JHU-O11, JHU-O29, OFK6/TERT1, FaDu, CAL 27)	1.81	iTRAQ	23665456
ISCU	head and neck cancer	neoplastic (JHU-O28, JHU-O22, JHU-O11, JHU-O29, OFK6/TERT1, FaDu, CAL 27)	-1.05	iTRAQ	23665456
ISOC2	head and neck cancer	neoplastic (JHU-O28, JHU-O22, JHU-O11, JHU-O29, OFK6/TERT1, FaDu, CAL 27)	-2.32	iTRAQ	23665456
ITGA3	head and neck cancer	neoplastic (JHU-O28, JHU-O22, JHU-O11, JHU-O29, OFK6/TERT1, FaDu, CAL 27)	1.59	iTRAQ	23665456
ITGB1	head and neck cancer	neoplastic (JHU-O28, JHU-O22, JHU-O11, JHU-O29, OFK6/TERT1, FaDu, CAL 27)	1.29	iTRAQ	23665456
ITIH2	head and neck cancer	neoplastic (JHU-O28, JHU-O22, JHU-O11, JHU-O29, OFK6/TERT1, FaDu, CAL 27)	2.72	iTRAQ	23665456
ITIH3	head and neck cancer	neoplastic (JHU-O28, JHU-O22, JHU-O11, JHU-O29, OFK6/TERT1, FaDu, CAL 27)	1.24	iTRAQ	23665456
JAKMIP2	head and neck cancer	neoplastic (JHU-O28, JHU-O22, JHU-O11, JHU-O29, OFK6/TERT1, FaDu, CAL 27)	2.01	iTRAQ	23665456
KDELR2	head and neck cancer	neoplastic (JHU-O28, JHU-O22, JHU-O11, JHU-O29, OFK6/TERT1, FaDu, CAL 27)	2.35	iTRAQ	23665456
KIAA1033	head and neck cancer	neoplastic (JHU-O28, JHU-O22, JHU-O11, JHU-O29, OFK6/TERT1, FaDu, CAL 27)	-1.83	iTRAQ	23665456
KIAA1949	head and neck cancer	neoplastic (JHU-O28, JHU-O22, JHU-O11, JHU-O29, OFK6/TERT1, FaDu, CAL 27)	-1.78	iTRAQ	23665456
KLK10	head and neck cancer	neoplastic (JHU-O28, JHU-O22, JHU-O11, JHU-O29, OFK6/TERT1, FaDu, CAL 27)	1.95	iTRAQ	23665456
KLK9	head and neck cancer	neoplastic (JHU-O28, JHU-O22, JHU-O11, JHU-O29, OFK6/TERT1, FaDu, CAL 27)	-1.55	iTRAQ	23665456
KNG1	head and neck cancer	neoplastic (JHU-O28, JHU-O22, JHU-O11, JHU-O29, OFK6/TERT1, FaDu, CAL 27)	1.39	iTRAQ	23665456
KPNA3	head and neck cancer	neoplastic (JHU-O28, JHU-O22, JHU-O11, JHU-O29, OFK6/TERT1, FaDu, CAL 27)	1.42	iTRAQ	23665456
KPNB1	head and neck cancer	neoplastic (JHU-O28, JHU-O22, JHU-O11, JHU-O29, OFK6/TERT1, FaDu, CAL 27)	1.13	iTRAQ	23665456
KPNB1	head and neck cancer	neoplastic (JHU-O28, JHU-O22, JHU-O11, JHU-O29, OFK6/TERT1, FaDu, CAL 27)	1.52	iTRAQ	23665456
KRI1	head and neck cancer	neoplastic (JHU-O28, JHU-O22, JHU-O11, JHU-O29, OFK6/TERT1, FaDu, CAL 27)	1.22	iTRAQ	23665456
KRT1	head and neck cancer	neoplastic (JHU-O28, JHU-O22, JHU-O11, JHU-O29, OFK6/TERT1, FaDu, CAL 27)	3.51	iTRAQ	23665456
KRT10	head and neck cancer	neoplastic (JHU-O28, JHU-O22, JHU-O11, JHU-O29, OFK6/TERT1, FaDu, CAL 27)	4.24	iTRAQ	23665456
KRT16	head and neck cancer	neoplastic (JHU-O28, JHU-O22, JHU-O11, JHU-O29, OFK6/TERT1, FaDu, CAL 27)	1.07	iTRAQ	23665456
KRT6B	head and neck cancer	neoplastic (JHU-O28, JHU-O22, JHU-O11, JHU-O29, OFK6/TERT1, FaDu, CAL 27)	3.68	iTRAQ	23665456
KRT8	head and neck cancer	neoplastic (JHU-O28, JHU-O22, JHU-O11, JHU-O29, OFK6/TERT1, FaDu, CAL 27)	1.15	iTRAQ	23665456
KRT9	head and neck cancer	neoplastic (JHU-O28, JHU-O22, JHU-O11, JHU-O29, OFK6/TERT1, FaDu, CAL 27)	5.02	iTRAQ	23665456
KYNU	head and neck cancer	neoplastic (JHU-O28, JHU-O22, JHU-O11, JHU-O29, OFK6/TERT1, FaDu, CAL 27)	2.12	iTRAQ	23665456
LAD1	head and neck cancer	neoplastic (JHU-O28, JHU-O22, JHU-O11, JHU-O29, OFK6/TERT1, FaDu, CAL 27)	-1.35	iTRAQ	23665456
LAGE3	head and neck cancer	neoplastic (JHU-O28, JHU-O22, JHU-O11, JHU-O29, OFK6/TERT1, FaDu, CAL 27)	1.12	iTRAQ	23665456

LAMA3	head and neck cancer	neoplastic (JHU-O28, JHU-O22, JHU-O11, JHU-O29, OFK6/TERT1, FaDu, CAL 27)	-1.21	iTRAQ	23665456
LAMA5	head and neck cancer	neoplastic (JHU-O28, JHU-O22, JHU-O11, JHU-O29, OFK6/TERT1, FaDu, CAL 27)	1.77	iTRAQ	23665456
LAMA5	head and neck cancer	neoplastic (JHU-O28, JHU-O22, JHU-O11, JHU-O29, OFK6/TERT1, FaDu, CAL 27)	2.40	iTRAQ	23665456
LAMB4	head and neck cancer	neoplastic (JHU-O28, JHU-O22, JHU-O11, JHU-O29, OFK6/TERT1, FaDu, CAL 27)	8.22	iTRAQ	23665456
LAMC1	head and neck cancer	neoplastic (JHU-O28, JHU-O22, JHU-O11, JHU-O29, OFK6/TERT1, FaDu, CAL 27)	1.51	iTRAQ	23665456
LAMC2	head and neck cancer	neoplastic (JHU-O28, JHU-O22, JHU-O11, JHU-O29, OFK6/TERT1, FaDu, CAL 27)	-1.47	iTRAQ	23665456
LAMC2	head and neck cancer	neoplastic (JHU-O28, JHU-O22, JHU-O11, JHU-O29, OFK6/TERT1, FaDu, CAL 27)	4.30	iTRAQ	23665456
LANCL1	head and neck cancer	neoplastic (JHU-O28, JHU-O22, JHU-O11, JHU-O29, OFK6/TERT1, FaDu, CAL 27)	-1.02	iTRAQ	23665456
LAP3	head and neck cancer	neoplastic (JHU-O28, JHU-O22, JHU-O11, JHU-O29, OFK6/TERT1, FaDu, CAL 27)	-1.32	iTRAQ	23665456
LAP3	head and neck cancer	neoplastic (JHU-O28, JHU-O22, JHU-O11, JHU-O29, OFK6/TERT1, FaDu, CAL 27)	-1.35	iTRAQ	23665456
LARP1	head and neck cancer	neoplastic (JHU-O28, JHU-O22, JHU-O11, JHU-O29, OFK6/TERT1, FaDu, CAL 27)	-1.05	iTRAQ	23665456
LARP4B	head and neck cancer	neoplastic (JHU-O28, JHU-O22, JHU-O11, JHU-O29, OFK6/TERT1, FaDu, CAL 27)	-1.39	iTRAQ	23665456
LCN2	head and neck cancer	neoplastic (JHU-O28, JHU-O22, JHU-O11, JHU-O29, OFK6/TERT1, FaDu, CAL 27)	2.69	iTRAQ	23665456
LDHAL6B	head and neck cancer	neoplastic (JHU-O28, JHU-O22, JHU-O11, JHU-O29, OFK6/TERT1, FaDu, CAL 27)	2	iTRAQ	23665456
LDLR	head and neck cancer	neoplastic (JHU-O28, JHU-O22, JHU-O11, JHU-O29, OFK6/TERT1, FaDu, CAL 27)	-1.88	iTRAQ	23665456
LEMD2	head and neck cancer	neoplastic (JHU-O28, JHU-O22, JHU-O11, JHU-O29, OFK6/TERT1, FaDu, CAL 27)	-1	iTRAQ	23665456
LETM1	head and neck cancer	neoplastic (JHU-O28, JHU-O22, JHU-O11, JHU-O29, OFK6/TERT1, FaDu, CAL 27)	-1.43	iTRAQ	23665456
LGALS1	head and neck cancer	neoplastic (JHU-O28, JHU-O22, JHU-O11, JHU-O29, OFK6/TERT1, FaDu, CAL 27)	-2.05	iTRAQ	23665456
LGALS3	head and neck cancer	neoplastic (JHU-O28, JHU-O22, JHU-O11, JHU-O29, OFK6/TERT1, FaDu, CAL 27)	-2	iTRAQ	23665456
LGALS3BP	head and neck cancer	neoplastic (JHU-O28, JHU-O22, JHU-O11, JHU-O29, OFK6/TERT1, FaDu, CAL 27)	3.21	iTRAQ	23665456
LGMN	head and neck cancer	neoplastic (JHU-O28, JHU-O22, JHU-O11, JHU-O29, OFK6/TERT1, FaDu, CAL 27)	1.94	iTRAQ	23665456
LIF	head and neck cancer	neoplastic (JHU-O28, JHU-O22, JHU-O11, JHU-O29, OFK6/TERT1, FaDu, CAL 27)	2.72	iTRAQ	23665456
LMAN1	head and neck cancer	neoplastic (JHU-O28, JHU-O22, JHU-O11, JHU-O29, OFK6/TERT1, FaDu, CAL 27)	-1.05	iTRAQ	23665456
LMNA	head and neck cancer	neoplastic (JHU-O28, JHU-O22, JHU-O11, JHU-O29, OFK6/TERT1, FaDu, CAL 27)	1.54	iTRAQ	23665456
LMNB2	head and neck cancer	neoplastic (JHU-O28, JHU-O22, JHU-O11, JHU-O29, OFK6/TERT1, FaDu, CAL 27)	-1.12	iTRAQ	23665456
LNPEP	head and neck cancer	neoplastic (JHU-O28, JHU-O22, JHU-O11, JHU-O29, OFK6/TERT1, FaDu, CAL 27)	-1.21	iTRAQ	23665456
LOC10050769	head and neck cancer	neoplastic (JHU-O28, JHU-O22, JHU-O11, JHU-O29, OFK6/TERT1, FaDu, CAL 27)	1.09	iTRAQ	23665456
LOC650157	head and neck cancer	neoplastic (JHU-O28, JHU-O22, JHU-O11, JHU-O29, OFK6/TERT1, FaDu, CAL 27)	-2.47	iTRAQ	23665456
LOC728763	head and neck cancer	neoplastic (JHU-O28, JHU-O22, JHU-O11, JHU-O29, OFK6/TERT1, FaDu, CAL 27)	-1.83	iTRAQ	23665456
LOX	head and neck cancer	neoplastic (JHU-O28, JHU-O22, JHU-O11, JHU-O29, OFK6/TERT1, FaDu, CAL 27)	1.22	iTRAQ	23665456
LOXL2	head and neck cancer	neoplastic (JHU-O28, JHU-O22, JHU-O11, JHU-O29, OFK6/TERT1, FaDu, CAL 27)	1.99	iTRAQ	23665456
LPHN1	head and neck cancer	neoplastic (JHU-O28, JHU-O22, JHU-O11, JHU-O29, OFK6/TERT1, FaDu, CAL 27)	-1.02	iTRAQ	23665456
LPP	head and neck cancer	neoplastic (JHU-O28, JHU-O22, JHU-O11, JHU-O29, OFK6/TERT1, FaDu, CAL 27)	-1.05	iTRAQ	23665456
LRRIQ1	head and neck cancer	neoplastic (JHU-O28, JHU-O22, JHU-O11, JHU-O29, OFK6/TERT1, FaDu, CAL 27)	5.07	iTRAQ	23665456
LRSAM1	head and neck cancer	neoplastic (JHU-O28, JHU-O22, JHU-O11, JHU-O29, OFK6/TERT1, FaDu, CAL 27)	-1	iTRAQ	23665456
LSM1	head and neck cancer	neoplastic (JHU-O28, JHU-O22, JHU-O11, JHU-O29, OFK6/TERT1, FaDu, CAL 27)	-1.02	iTRAQ	23665456
LSM5	head and neck cancer	neoplastic (JHU-O28, JHU-O22, JHU-O11, JHU-O29, OFK6/TERT1, FaDu, CAL 27)	-1.39	iTRAQ	23665456
LSM7	head and neck cancer	neoplastic (JHU-O28, JHU-O22, JHU-O11, JHU-O29, OFK6/TERT1, FaDu, CAL 27)	-1.12	iTRAQ	23665456
LTA4H	head and neck cancer	neoplastic (JHU-O28, JHU-O22, JHU-O11, JHU-O29, OFK6/TERT1, FaDu, CAL 27)	-1.35	iTRAQ	23665456
LTBP3	head and neck cancer	neoplastic (JHU-O28, JHU-O22, JHU-O11, JHU-O29, OFK6/TERT1, FaDu, CAL 27)	1.44	iTRAQ	23665456
LTF	head and neck cancer	neoplastic (JHU-O28, JHU-O22, JHU-O11, JHU-O29, OFK6/TERT1, FaDu, CAL 27)	2.12	iTRAQ	23665456
LUC7L2	head and neck cancer	neoplastic (JHU-O28, JHU-O22, JHU-O11, JHU-O29, OFK6/TERT1, FaDu, CAL 27)	1.17	iTRAQ	23665456
LUZP1	head and neck cancer	neoplastic (JHU-O28, JHU-O22, JHU-O11, JHU-O29, OFK6/TERT1, FaDu, CAL 27)	-1.02	iTRAQ	23665456

LYPD3	head and neck cancer	neoplastic (JHU-O28, JHU-O22, JHU-O11, JHU-O29, OFK6/TERT1, FaDu, CAL 27)	-1.32	iTRAQ	23665456
LZIC	head and neck cancer	neoplastic (JHU-O28, JHU-O22, JHU-O11, JHU-O29, OFK6/TERT1, FaDu, CAL 27)	1.62	iTRAQ	23665456
MAGEA4	head and neck cancer	neoplastic (JHU-O28, JHU-O22, JHU-O11, JHU-O29, OFK6/TERT1, FaDu, CAL 27)	2.67	iTRAQ	23665456
MAGOH	head and neck cancer	neoplastic (JHU-O28, JHU-O22, JHU-O11, JHU-O29, OFK6/TERT1, FaDu, CAL 27)	2.41	iTRAQ	23665456
MAP2	head and neck cancer	neoplastic (JHU-O28, JHU-O22, JHU-O11, JHU-O29, OFK6/TERT1, FaDu, CAL 27)	-2.39	iTRAQ	23665456
MAP4	head and neck cancer	neoplastic (JHU-O28, JHU-O22, JHU-O11, JHU-O29, OFK6/TERT1, FaDu, CAL 27)	-1.18	iTRAQ	23665456
MAST4	head and neck cancer	neoplastic (JHU-O28, JHU-O22, JHU-O11, JHU-O29, OFK6/TERT1, FaDu, CAL 27)	-1.15	iTRAQ	23665456
MAVS	head and neck cancer	neoplastic (JHU-O28, JHU-O22, JHU-O11, JHU-O29, OFK6/TERT1, FaDu, CAL 27)	-2	iTRAQ	23665456
MCAM	head and neck cancer	neoplastic (JHU-O28, JHU-O22, JHU-O11, JHU-O29, OFK6/TERT1, FaDu, CAL 27)	-1.73	iTRAQ	23665456
MCM2	head and neck cancer	neoplastic (JHU-O28, JHU-O22, JHU-O11, JHU-O29, OFK6/TERT1, FaDu, CAL 27)	1.53	iTRAQ	23665456
MCM4	head and neck cancer	neoplastic (JHU-O28, JHU-O22, JHU-O11, JHU-O29, OFK6/TERT1, FaDu, CAL 27)	1.06	iTRAQ	23665456
MCM7	head and neck cancer	neoplastic (JHU-O28, JHU-O22, JHU-O11, JHU-O29, OFK6/TERT1, FaDu, CAL 27)	2.33	iTRAQ	23665456
MDH1	head and neck cancer	neoplastic (JHU-O28, JHU-O22, JHU-O11, JHU-O29, OFK6/TERT1, FaDu, CAL 27)	-1.21	iTRAQ	23665456
MDH2	head and neck cancer	neoplastic (JHU-O28, JHU-O22, JHU-O11, JHU-O29, OFK6/TERT1, FaDu, CAL 27)	-1.51	iTRAQ	23665456
MDK	head and neck cancer	neoplastic (JHU-O28, JHU-O22, JHU-O11, JHU-O29, OFK6/TERT1, FaDu, CAL 27)	1.99	iTRAQ	23665456
MED15	head and neck cancer	neoplastic (JHU-O28, JHU-O22, JHU-O11, JHU-O29, OFK6/TERT1, FaDu, CAL 27)	-1.73	iTRAQ	23665456
METAP2	head and neck cancer	neoplastic (JHU-O28, JHU-O22, JHU-O11, JHU-O29, OFK6/TERT1, FaDu, CAL 27)	-1.73	iTRAQ	23665456
METAP2	head and neck cancer	neoplastic (JHU-O28, JHU-O22, JHU-O11, JHU-O29, OFK6/TERT1, FaDu, CAL 27)	4.48	iTRAQ	23665456
MIF	head and neck cancer	neoplastic (JHU-O28, JHU-O22, JHU-O11, JHU-O29, OFK6/TERT1, FaDu, CAL 27)	-3.18	iTRAQ	23665456
MKI67	head and neck cancer	neoplastic (JHU-O28, JHU-O22, JHU-O11, JHU-O29, OFK6/TERT1, FaDu, CAL 27)	1.37	iTRAQ	23665456
MMAB	head and neck cancer	neoplastic (JHU-O28, JHU-O22, JHU-O11, JHU-O29, OFK6/TERT1, FaDu, CAL 27)	-1.35	iTRAQ	23665456
MMP1	head and neck cancer	neoplastic (JHU-O28, JHU-O22, JHU-O11, JHU-O29, OFK6/TERT1, FaDu, CAL 27)	3.09	iTRAQ	23665456
MMP1	head and neck cancer	neoplastic (JHU-O28, JHU-O22, JHU-O11, JHU-O29, OFK6/TERT1, FaDu, CAL 27)	4.57	iTRAQ	23665456
MMP10	head and neck cancer	neoplastic (JHU-O28, JHU-O22, JHU-O11, JHU-O29, OFK6/TERT1, FaDu, CAL 27)	1.20	iTRAQ	23665456
MMP10	head and neck cancer	neoplastic (JHU-O28, JHU-O22, JHU-O11, JHU-O29, OFK6/TERT1, FaDu, CAL 27)	2.48	iTRAQ	23665456
MMP12	head and neck cancer	neoplastic (JHU-O28, JHU-O22, JHU-O11, JHU-O29, OFK6/TERT1, FaDu, CAL 27)	1.69	iTRAQ	23665456
MMP13	head and neck cancer	neoplastic (JHU-O28, JHU-O22, JHU-O11, JHU-O29, OFK6/TERT1, FaDu, CAL 27)	2.45	iTRAQ	23665456
MMP17	head and neck cancer	neoplastic (JHU-O28, JHU-O22, JHU-O11, JHU-O29, OFK6/TERT1, FaDu, CAL 27)	-1.32	iTRAQ	23665456
MMP2	head and neck cancer	neoplastic (JHU-O28, JHU-O22, JHU-O11, JHU-O29, OFK6/TERT1, FaDu, CAL 27)	1.32	iTRAQ	23665456
MMP7	head and neck cancer	neoplastic (JHU-O28, JHU-O22, JHU-O11, JHU-O29, OFK6/TERT1, FaDu, CAL 27)	2.34	iTRAQ	23665456
MMP9	head and neck cancer	neoplastic (JHU-O28, JHU-O22, JHU-O11, JHU-O29, OFK6/TERT1, FaDu, CAL 27)	-1.59	iTRAQ	23665456
MOCS2	head and neck cancer	neoplastic (JHU-O28, JHU-O22, JHU-O11, JHU-O29, OFK6/TERT1, FaDu, CAL 27)	-1.05	iTRAQ	23665456
MPZL1	head and neck cancer	neoplastic (JHU-O28, JHU-O22, JHU-O11, JHU-O29, OFK6/TERT1, FaDu, CAL 27)	-1.28	iTRAQ	23665456
MRC2	head and neck cancer	neoplastic (JHU-O28, JHU-O22, JHU-O11, JHU-O29, OFK6/TERT1, FaDu, CAL 27)	-1.12	iTRAQ	23665456
MRPL12	head and neck cancer	neoplastic (JHU-O28, JHU-O22, JHU-O11, JHU-O29, OFK6/TERT1, FaDu, CAL 27)	-1.47	iTRAQ	23665456
MRPS23	head and neck cancer	neoplastic (JHU-O28, JHU-O22, JHU-O11, JHU-O29, OFK6/TERT1, FaDu, CAL 27)	-1.94	iTRAQ	23665456
MSRA	head and neck cancer	neoplastic (JHU-O28, JHU-O22, JHU-O11, JHU-O29, OFK6/TERT1, FaDu, CAL 27)	-1.55	iTRAQ	23665456
MST1	head and neck cancer	neoplastic (JHU-O28, JHU-O22, JHU-O11, JHU-O29, OFK6/TERT1, FaDu, CAL 27)	2.15	iTRAQ	23665456
MT1G	head and neck cancer	neoplastic (JHU-O28, JHU-O22, JHU-O11, JHU-O29, OFK6/TERT1, FaDu, CAL 27)	-2.05	iTRAQ	23665456
MTPN	head and neck cancer	neoplastic (JHU-O28, JHU-O22, JHU-O11, JHU-O29, OFK6/TERT1, FaDu, CAL 27)	-1.35	iTRAQ	23665456
MVD	head and neck cancer	neoplastic (JHU-O28, JHU-O22, JHU-O11, JHU-O29, OFK6/TERT1, FaDu, CAL 27)	-1.39	iTRAQ	23665456
MVP	head and neck cancer	neoplastic (JHU-O28, JHU-O22, JHU-O11, JHU-O29, OFK6/TERT1, FaDu, CAL 27)	1.59	iTRAQ	23665456
MYD88	head and neck cancer	neoplastic (JHU-O28, JHU-O22, JHU-O11, JHU-O29, OFK6/TERT1, FaDu, CAL 27)	-2	iTRAQ	23665456

MYH9	head and neck cancer	neoplastic (JHU-O28, JHU-O22, JHU-O11, JHU-O29, OFK6/TERT1, FaDu, CAL 27)	2	iTRAQ	23665456
MYH9	head and neck cancer	neoplastic (JHU-O28, JHU-O22, JHU-O11, JHU-O29, OFK6/TERT1, FaDu, CAL 27)	1.03	iTRAQ	23665456
MYL12A	head and neck cancer	neoplastic (JHU-O28, JHU-O22, JHU-O11, JHU-O29, OFK6/TERT1, FaDu, CAL 27)	1.41	iTRAQ	23665456
MYO6	head and neck cancer	neoplastic (JHU-O28, JHU-O22, JHU-O11, JHU-O29, OFK6/TERT1, FaDu, CAL 27)	1.33	iTRAQ	23665456
NACA	head and neck cancer	neoplastic (JHU-O28, JHU-O22, JHU-O11, JHU-O29, OFK6/TERT1, FaDu, CAL 27)	2.20	iTRAQ	23665456
NACA2	head and neck cancer	neoplastic (JHU-O28, JHU-O22, JHU-O11, JHU-O29, OFK6/TERT1, FaDu, CAL 27)	1.83	iTRAQ	23665456
NAGK	head and neck cancer	neoplastic (JHU-O28, JHU-O22, JHU-O11, JHU-O29, OFK6/TERT1, FaDu, CAL 27)	-1.15	iTRAQ	23665456
NAMPT	head and neck cancer	neoplastic (JHU-O28, JHU-O22, JHU-O11, JHU-O29, OFK6/TERT1, FaDu, CAL 27)	2.13	iTRAQ	23665456
NAP1L1	head and neck cancer	neoplastic (JHU-O28, JHU-O22, JHU-O11, JHU-O29, OFK6/TERT1, FaDu, CAL 27)	2.30	iTRAQ	23665456
NAP1L4	head and neck cancer	neoplastic (JHU-O28, JHU-O22, JHU-O11, JHU-O29, OFK6/TERT1, FaDu, CAL 27)	2.02	iTRAQ	23665456
NASP	head and neck cancer	neoplastic (JHU-O28, JHU-O22, JHU-O11, JHU-O29, OFK6/TERT1, FaDu, CAL 27)	1.21	iTRAQ	23665456
NAT10	head and neck cancer	neoplastic (JHU-O28, JHU-O22, JHU-O11, JHU-O29, OFK6/TERT1, FaDu, CAL 27)	1.26	iTRAQ	23665456
NBPF11	head and neck cancer	neoplastic (JHU-O28, JHU-O22, JHU-O11, JHU-O29, OFK6/TERT1, FaDu, CAL 27)	-2.32	iTRAQ	23665456
NCAM1	head and neck cancer	neoplastic (JHU-O28, JHU-O22, JHU-O11, JHU-O29, OFK6/TERT1, FaDu, CAL 27)	-1.43	iTRAQ	23665456
NCK1	head and neck cancer	neoplastic (JHU-O28, JHU-O22, JHU-O11, JHU-O29, OFK6/TERT1, FaDu, CAL 27)	-1.25	iTRAQ	23665456
NCL	head and neck cancer	neoplastic (JHU-O28, JHU-O22, JHU-O11, JHU-O29, OFK6/TERT1, FaDu, CAL 27)	2.17	iTRAQ	23665456
NCSTN	head and neck cancer	neoplastic (JHU-O28, JHU-O22, JHU-O11, JHU-O29, OFK6/TERT1, FaDu, CAL 27)	1.11	iTRAQ	23665456
NDRG3	head and neck cancer	neoplastic (JHU-O28, JHU-O22, JHU-O11, JHU-O29, OFK6/TERT1, FaDu, CAL 27)	-1.08	iTRAQ	23665456
NDUFAB1	head and neck cancer	neoplastic (JHU-O28, JHU-O22, JHU-O11, JHU-O29, OFK6/TERT1, FaDu, CAL 27)	-1.88	iTRAQ	23665456
NEDD8	head and neck cancer	neoplastic (JHU-O28, JHU-O22, JHU-O11, JHU-O29, OFK6/TERT1, FaDu, CAL 27)	-2	iTRAQ	23665456
NFKB2	head and neck cancer	neoplastic (JHU-O28, JHU-O22, JHU-O11, JHU-O29, OFK6/TERT1, FaDu, CAL 27)	1.62	iTRAQ	23665456
NISCH	head and neck cancer	neoplastic (JHU-O28, JHU-O22, JHU-O11, JHU-O29, OFK6/TERT1, FaDu, CAL 27)	1.07	iTRAQ	23665456
NIT2	head and neck cancer	neoplastic (JHU-O28, JHU-O22, JHU-O11, JHU-O29, OFK6/TERT1, FaDu, CAL 27)	-1	iTRAQ	23665456
NLE1	head and neck cancer	neoplastic (JHU-O28, JHU-O22, JHU-O11, JHU-O29, OFK6/TERT1, FaDu, CAL 27)	2.31	iTRAQ	23665456
NME1-NME2	head and neck cancer	neoplastic (JHU-O28, JHU-O22, JHU-O11, JHU-O29, OFK6/TERT1, FaDu, CAL 27)	-1	iTRAQ	23665456
NME2	head and neck cancer	neoplastic (JHU-O28, JHU-O22, JHU-O11, JHU-O29, OFK6/TERT1, FaDu, CAL 27)	-1	iTRAQ	23665456
NOL4	head and neck cancer	neoplastic (JHU-O28, JHU-O22, JHU-O11, JHU-O29, OFK6/TERT1, FaDu, CAL 27)	3.95	iTRAQ	23665456
NP	head and neck cancer	neoplastic (JHU-O28, JHU-O22, JHU-O11, JHU-O29, OFK6/TERT1, FaDu, CAL 27)	-1	iTRAQ	23665456
NPM1	head and neck cancer	neoplastic (JHU-O28, JHU-O22, JHU-O11, JHU-O29, OFK6/TERT1, FaDu, CAL 27)	2.19	iTRAQ	23665456
NRCAM	head and neck cancer	neoplastic (JHU-O28, JHU-O22, JHU-O11, JHU-O29, OFK6/TERT1, FaDu, CAL 27)	-1.78	iTRAQ	23665456
NRD1	head and neck cancer	neoplastic (JHU-O28, JHU-O22, JHU-O11, JHU-O29, OFK6/TERT1, FaDu, CAL 27)	2.12	iTRAQ	23665456
NRP1	head and neck cancer	neoplastic (JHU-O28, JHU-O22, JHU-O11, JHU-O29, OFK6/TERT1, FaDu, CAL 27)	1.04	iTRAQ	23665456
NSUN2	head and neck cancer	neoplastic (JHU-O28, JHU-O22, JHU-O11, JHU-O29, OFK6/TERT1, FaDu, CAL 27)	1.92	iTRAQ	23665456
NT5C2	head and neck cancer	neoplastic (JHU-O28, JHU-O22, JHU-O11, JHU-O29, OFK6/TERT1, FaDu, CAL 27)	1.58	iTRAQ	23665456
NTN4	head and neck cancer	neoplastic (JHU-O28, JHU-O22, JHU-O11, JHU-O29, OFK6/TERT1, FaDu, CAL 27)	1.63	iTRAQ	23665456
NUDC	head and neck cancer	neoplastic (JHU-O28, JHU-O22, JHU-O11, JHU-O29, OFK6/TERT1, FaDu, CAL 27)	1.12	iTRAQ	23665456
NUDCD1	head and neck cancer	neoplastic (JHU-O28, JHU-O22, JHU-O11, JHU-O29, OFK6/TERT1, FaDu, CAL 27)	1.40	iTRAQ	23665456
NUDT2	head and neck cancer	neoplastic (JHU-O28, JHU-O22, JHU-O11, JHU-O29, OFK6/TERT1, FaDu, CAL 27)	-1.59	iTRAQ	23665456
NUDT9	head and neck cancer	neoplastic (JHU-O28, JHU-O22, JHU-O11, JHU-O29, OFK6/TERT1, FaDu, CAL 27)	-1.32	iTRAQ	23665456
NUTF2	head and neck cancer	neoplastic (JHU-O28, JHU-O22, JHU-O11, JHU-O29, OFK6/TERT1, FaDu, CAL 27)	-1.12	iTRAQ	23665456
ODZ2	head and neck cancer	neoplastic (JHU-O28, JHU-O22, JHU-O11, JHU-O29, OFK6/TERT1, FaDu, CAL 27)	-1.73	iTRAQ	23665456
OGFR	head and neck cancer	neoplastic (JHU-O28, JHU-O22, JHU-O11, JHU-O29, OFK6/TERT1, FaDu, CAL 27)	3.44	iTRAQ	23665456
OLFM4	head and neck cancer	neoplastic (JHU-O28, JHU-O22, JHU-O11, JHU-O29, OFK6/TERT1, FaDu, CAL 27)	3.58	iTRAQ	23665456

OLFML2A	head and neck cancer	neoplastic (JHU-O28, JHU-O22, JHU-O11, JHU-O29, OFK6/TERT1, FaDu, CAL 27)	3.04	iTRAQ	23665456
OLR1	head and neck cancer	neoplastic (JHU-O28, JHU-O22, JHU-O11, JHU-O29, OFK6/TERT1, FaDu, CAL 27)	2.02	iTRAQ	23665456
OSBP	head and neck cancer	neoplastic (JHU-O28, JHU-O22, JHU-O11, JHU-O29, OFK6/TERT1, FaDu, CAL 27)	1.32	iTRAQ	23665456
OTOP1	head and neck cancer	neoplastic (JHU-O28, JHU-O22, JHU-O11, JHU-O29, OFK6/TERT1, FaDu, CAL 27)	1.72	iTRAQ	23665456
OXCT1	head and neck cancer	neoplastic (JHU-O28, JHU-O22, JHU-O11, JHU-O29, OFK6/TERT1, FaDu, CAL 27)	-1.35	iTRAQ	23665456
P4HA2	head and neck cancer	neoplastic (JHU-O28, JHU-O22, JHU-O11, JHU-O29, OFK6/TERT1, FaDu, CAL 27)	1.35	iTRAQ	23665456
P4HB	head and neck cancer	neoplastic (JHU-O28, JHU-O22, JHU-O11, JHU-O29, OFK6/TERT1, FaDu, CAL 27)	1.32	iTRAQ	23665456
PABPC4	head and neck cancer	neoplastic (JHU-O28, JHU-O22, JHU-O11, JHU-O29, OFK6/TERT1, FaDu, CAL 27)	1.15	iTRAQ	23665456
PACSIN2	head and neck cancer	neoplastic (JHU-O28, JHU-O22, JHU-O11, JHU-O29, OFK6/TERT1, FaDu, CAL 27)	1.04	iTRAQ	23665456
PADI3	head and neck cancer	neoplastic (JHU-O28, JHU-O22, JHU-O11, JHU-O29, OFK6/TERT1, FaDu, CAL 27)	1.33	iTRAQ	23665456
PAFAH1B1	head and neck cancer	neoplastic (JHU-O28, JHU-O22, JHU-O11, JHU-O29, OFK6/TERT1, FaDu, CAL 27)	1.52	iTRAQ	23665456
PAFAH1B2	head and neck cancer	neoplastic (JHU-O28, JHU-O22, JHU-O11, JHU-O29, OFK6/TERT1, FaDu, CAL 27)	-1.12	iTRAQ	23665456
PAFAH1B3	head and neck cancer	neoplastic (JHU-O28, JHU-O22, JHU-O11, JHU-O29, OFK6/TERT1, FaDu, CAL 27)	-1.02	iTRAQ	23665456
PALM2-AKAP	head and neck cancer	neoplastic (JHU-O28, JHU-O22, JHU-O11, JHU-O29, OFK6/TERT1, FaDu, CAL 27)	-1.21	iTRAQ	23665456
PARVA	head and neck cancer	neoplastic (JHU-O28, JHU-O22, JHU-O11, JHU-O29, OFK6/TERT1, FaDu, CAL 27)	-1.39	iTRAQ	23665456
PCDH15	head and neck cancer	neoplastic (JHU-O28, JHU-O22, JHU-O11, JHU-O29, OFK6/TERT1, FaDu, CAL 27)	-1.73	iTRAQ	23665456
PCDH19	head and neck cancer	neoplastic (JHU-O28, JHU-O22, JHU-O11, JHU-O29, OFK6/TERT1, FaDu, CAL 27)	-1.73	iTRAQ	23665456
PCDH7	head and neck cancer	neoplastic (JHU-O28, JHU-O22, JHU-O11, JHU-O29, OFK6/TERT1, FaDu, CAL 27)	1.18	iTRAQ	23665456
PCDHGC3	head and neck cancer	neoplastic (JHU-O28, JHU-O22, JHU-O11, JHU-O29, OFK6/TERT1, FaDu, CAL 27)	-1.12	iTRAQ	23665456
PCK2	head and neck cancer	neoplastic (JHU-O28, JHU-O22, JHU-O11, JHU-O29, OFK6/TERT1, FaDu, CAL 27)	-1.35	iTRAQ	23665456
PCMT1	head and neck cancer	neoplastic (JHU-O28, JHU-O22, JHU-O11, JHU-O29, OFK6/TERT1, FaDu, CAL 27)	-1.28	iTRAQ	23665456
PCSK9	head and neck cancer	neoplastic (JHU-O28, JHU-O22, JHU-O11, JHU-O29, OFK6/TERT1, FaDu, CAL 27)	1.11	iTRAQ	23665456
PDGFA	head and neck cancer	neoplastic (JHU-O28, JHU-O22, JHU-O11, JHU-O29, OFK6/TERT1, FaDu, CAL 27)	1.50	iTRAQ	23665456
PDGFD	head and neck cancer	neoplastic (JHU-O28, JHU-O22, JHU-O11, JHU-O29, OFK6/TERT1, FaDu, CAL 27)	2.20	iTRAQ	23665456
PDIA3	head and neck cancer	neoplastic (JHU-O28, JHU-O22, JHU-O11, JHU-O29, OFK6/TERT1, FaDu, CAL 27)	1.13	iTRAQ	23665456
PDIA3	head and neck cancer	neoplastic (JHU-O28, JHU-O22, JHU-O11, JHU-O29, OFK6/TERT1, FaDu, CAL 27)	1.16	iTRAQ	23665456
PDIA4	head and neck cancer	neoplastic (JHU-O28, JHU-O22, JHU-O11, JHU-O29, OFK6/TERT1, FaDu, CAL 27)	2.25	iTRAQ	23665456
PDIA6	head and neck cancer	neoplastic (JHU-O28, JHU-O22, JHU-O11, JHU-O29, OFK6/TERT1, FaDu, CAL 27)	1.61	iTRAQ	23665456
PDLIM3	head and neck cancer	neoplastic (JHU-O28, JHU-O22, JHU-O11, JHU-O29, OFK6/TERT1, FaDu, CAL 27)	-1.94	iTRAQ	23665456
PDLIM5	head and neck cancer	neoplastic (JHU-O28, JHU-O22, JHU-O11, JHU-O29, OFK6/TERT1, FaDu, CAL 27)	-1.35	iTRAQ	23665456
PDXK	head and neck cancer	neoplastic (JHU-O28, JHU-O22, JHU-O11, JHU-O29, OFK6/TERT1, FaDu, CAL 27)	-1.55	iTRAQ	23665456
PEX19	head and neck cancer	neoplastic (JHU-O28, JHU-O22, JHU-O11, JHU-O29, OFK6/TERT1, FaDu, CAL 27)	-1.47	iTRAQ	23665456
PFDN4	head and neck cancer	neoplastic (JHU-O28, JHU-O22, JHU-O11, JHU-O29, OFK6/TERT1, FaDu, CAL 27)	1.57	iTRAQ	23665456
PFKM	head and neck cancer	neoplastic (JHU-O28, JHU-O22, JHU-O11, JHU-O29, OFK6/TERT1, FaDu, CAL 27)	6.44	iTRAQ	23665456
PFN1	head and neck cancer	neoplastic (JHU-O28, JHU-O22, JHU-O11, JHU-O29, OFK6/TERT1, FaDu, CAL 27)	-1.15	iTRAQ	23665456
PFN2	head and neck cancer	neoplastic (JHU-O28, JHU-O22, JHU-O11, JHU-O29, OFK6/TERT1, FaDu, CAL 27)	-1.73	iTRAQ	23665456
PFN2	head and neck cancer	neoplastic (JHU-O28, JHU-O22, JHU-O11, JHU-O29, OFK6/TERT1, FaDu, CAL 27)	-1.94	iTRAQ	23665456
PFN2	head and neck cancer	neoplastic (JHU-O28, JHU-O22, JHU-O11, JHU-O29, OFK6/TERT1, FaDu, CAL 27)	-2.83	iTRAQ	23665456
PGCP	head and neck cancer	neoplastic (JHU-O28, JHU-O22, JHU-O11, JHU-O29, OFK6/TERT1, FaDu, CAL 27)	-1.73	iTRAQ	23665456
PGD	head and neck cancer	neoplastic (JHU-O28, JHU-O22, JHU-O11, JHU-O29, OFK6/TERT1, FaDu, CAL 27)	1	iTRAQ	23665456
PGLS	head and neck cancer	neoplastic (JHU-O28, JHU-O22, JHU-O11, JHU-O29, OFK6/TERT1, FaDu, CAL 27)	-1.43	iTRAQ	23665456
PGM2	head and neck cancer	neoplastic (JHU-O28, JHU-O22, JHU-O11, JHU-O29, OFK6/TERT1, FaDu, CAL 27)	-1.15	iTRAQ	23665456
PGP	head and neck cancer	neoplastic (JHU-O28, JHU-O22, JHU-O11, JHU-O29, OFK6/TERT1, FaDu, CAL 27)	-1.02	iTRAQ	23665456

PHLDB2	head and neck cancer	neoplastic (JHU-O28, JHU-O22, JHU-O11, JHU-O29, OFK6/TERT1, FaDu, CAL 27)	-1.05	iTRAQ	23665456
PITPNB	head and neck cancer	neoplastic (JHU-O28, JHU-O22, JHU-O11, JHU-O29, OFK6/TERT1, FaDu, CAL 27)	-1.43	iTRAQ	23665456
PIWIL2	head and neck cancer	neoplastic (JHU-O28, JHU-O22, JHU-O11, JHU-O29, OFK6/TERT1, FaDu, CAL 27)	3.39	iTRAQ	23665456
PKM2	head and neck cancer	neoplastic (JHU-O28, JHU-O22, JHU-O11, JHU-O29, OFK6/TERT1, FaDu, CAL 27)	3.73	iTRAQ	23665456
PLAT	head and neck cancer	neoplastic (JHU-O28, JHU-O22, JHU-O11, JHU-O29, OFK6/TERT1, FaDu, CAL 27)	2.05	iTRAQ	23665456
PLAU	head and neck cancer	neoplastic (JHU-O28, JHU-O22, JHU-O11, JHU-O29, OFK6/TERT1, FaDu, CAL 27)	1.59	iTRAQ	23665456
PLD3	head and neck cancer	neoplastic (JHU-O28, JHU-O22, JHU-O11, JHU-O29, OFK6/TERT1, FaDu, CAL 27)	-1.55	iTRAQ	23665456
PLEKHA5	head and neck cancer	neoplastic (JHU-O28, JHU-O22, JHU-O11, JHU-O29, OFK6/TERT1, FaDu, CAL 27)	-1.68	iTRAQ	23665456
PLEKHF2	head and neck cancer	neoplastic (JHU-O28, JHU-O22, JHU-O11, JHU-O29, OFK6/TERT1, FaDu, CAL 27)	2.78	iTRAQ	23665456
PLIN3	head and neck cancer	neoplastic (JHU-O28, JHU-O22, JHU-O11, JHU-O29, OFK6/TERT1, FaDu, CAL 27)	-1.55	iTRAQ	23665456
PLK1S1	head and neck cancer	neoplastic (JHU-O28, JHU-O22, JHU-O11, JHU-O29, OFK6/TERT1, FaDu, CAL 27)	-2	iTRAQ	23665456
PLOD1	head and neck cancer	neoplastic (JHU-O28, JHU-O22, JHU-O11, JHU-O29, OFK6/TERT1, FaDu, CAL 27)	1.20	iTRAQ	23665456
PLS3	head and neck cancer	neoplastic (JHU-O28, JHU-O22, JHU-O11, JHU-O29, OFK6/TERT1, FaDu, CAL 27)	-1.25	iTRAQ	23665456
PLS3	head and neck cancer	neoplastic (JHU-O28, JHU-O22, JHU-O11, JHU-O29, OFK6/TERT1, FaDu, CAL 27)	1.37	iTRAQ	23665456
PLTP	head and neck cancer	neoplastic (JHU-O28, JHU-O22, JHU-O11, JHU-O29, OFK6/TERT1, FaDu, CAL 27)	-2.12	iTRAQ	23665456
PLXDC2	head and neck cancer	neoplastic (JHU-O28, JHU-O22, JHU-O11, JHU-O29, OFK6/TERT1, FaDu, CAL 27)	-1.21	iTRAQ	23665456
PLXNA2	head and neck cancer	neoplastic (JHU-O28, JHU-O22, JHU-O11, JHU-O29, OFK6/TERT1, FaDu, CAL 27)	-1.59	iTRAQ	23665456
PNP	head and neck cancer	neoplastic (JHU-O28, JHU-O22, JHU-O11, JHU-O29, OFK6/TERT1, FaDu, CAL 27)	-1	iTRAQ	23665456
PNP	head and neck cancer	neoplastic (JHU-O28, JHU-O22, JHU-O11, JHU-O29, OFK6/TERT1, FaDu, CAL 27)	-1.39	iTRAQ	23665456
PNPO	head and neck cancer	neoplastic (JHU-O28, JHU-O22, JHU-O11, JHU-O29, OFK6/TERT1, FaDu, CAL 27)	-1.08	iTRAQ	23665456
POF1B	head and neck cancer	neoplastic (JHU-O28, JHU-O22, JHU-O11, JHU-O29, OFK6/TERT1, FaDu, CAL 27)	-1.25	iTRAQ	23665456
POFUT2	head and neck cancer	neoplastic (JHU-O28, JHU-O22, JHU-O11, JHU-O29, OFK6/TERT1, FaDu, CAL 27)	-1.05	iTRAQ	23665456
POSTN	head and neck cancer	neoplastic (JHU-O28, JHU-O22, JHU-O11, JHU-O29, OFK6/TERT1, FaDu, CAL 27)	1.85	iTRAQ	23665456
PPA2	head and neck cancer	neoplastic (JHU-O28, JHU-O22, JHU-O11, JHU-O29, OFK6/TERT1, FaDu, CAL 27)	-1.88	iTRAQ	23665456
PPIA	head and neck cancer	neoplastic (JHU-O28, JHU-O22, JHU-O11, JHU-O29, OFK6/TERT1, FaDu, CAL 27)	-1.64	iTRAQ	23665456
PPIC	head and neck cancer	neoplastic (JHU-O28, JHU-O22, JHU-O11, JHU-O29, OFK6/TERT1, FaDu, CAL 27)	-1.32	iTRAQ	23665456
PPIF	head and neck cancer	neoplastic (JHU-O28, JHU-O22, JHU-O11, JHU-O29, OFK6/TERT1, FaDu, CAL 27)	-1.05	iTRAQ	23665456
PPIL1	head and neck cancer	neoplastic (JHU-O28, JHU-O22, JHU-O11, JHU-O29, OFK6/TERT1, FaDu, CAL 27)	2.20	iTRAQ	23665456
PPM1G	head and neck cancer	neoplastic (JHU-O28, JHU-O22, JHU-O11, JHU-O29, OFK6/TERT1, FaDu, CAL 27)	2.57	iTRAQ	23665456
PPME1	head and neck cancer	neoplastic (JHU-O28, JHU-O22, JHU-O11, JHU-O29, OFK6/TERT1, FaDu, CAL 27)	1.16	iTRAQ	23665456
PPP2R4	head and neck cancer	neoplastic (JHU-O28, JHU-O22, JHU-O11, JHU-O29, OFK6/TERT1, FaDu, CAL 27)	-1.35	iTRAQ	23665456
PPP3CA	head and neck cancer	neoplastic (JHU-O28, JHU-O22, JHU-O11, JHU-O29, OFK6/TERT1, FaDu, CAL 27)	-1.47	iTRAQ	23665456
PPP6R1	head and neck cancer	neoplastic (JHU-O28, JHU-O22, JHU-O11, JHU-O29, OFK6/TERT1, FaDu, CAL 27)	-1.39	iTRAQ	23665456
PPT1	head and neck cancer	neoplastic (JHU-O28, JHU-O22, JHU-O11, JHU-O29, OFK6/TERT1, FaDu, CAL 27)	1.30	iTRAQ	23665456
PRCP	head and neck cancer	neoplastic (JHU-O28, JHU-O22, JHU-O11, JHU-O29, OFK6/TERT1, FaDu, CAL 27)	1.82	iTRAQ	23665456
PRDX2	head and neck cancer	neoplastic (JHU-O28, JHU-O22, JHU-O11, JHU-O29, OFK6/TERT1, FaDu, CAL 27)	-1.51	iTRAQ	23665456
PRDX3	head and neck cancer	neoplastic (JHU-O28, JHU-O22, JHU-O11, JHU-O29, OFK6/TERT1, FaDu, CAL 27)	-1.39	iTRAQ	23665456
PRDX3	head and neck cancer	neoplastic (JHU-O28, JHU-O22, JHU-O11, JHU-O29, OFK6/TERT1, FaDu, CAL 27)	-1.73	iTRAQ	23665456
PRDX4	head and neck cancer	neoplastic (JHU-O28, JHU-O22, JHU-O11, JHU-O29, OFK6/TERT1, FaDu, CAL 27)	1.15	iTRAQ	23665456
PRDX5	head and neck cancer	neoplastic (JHU-O28, JHU-O22, JHU-O11, JHU-O29, OFK6/TERT1, FaDu, CAL 27)	-1.59	iTRAQ	23665456
PRDX6	head and neck cancer	neoplastic (JHU-O28, JHU-O22, JHU-O11, JHU-O29, OFK6/TERT1, FaDu, CAL 27)	-1.83	iTRAQ	23665456
PRKCSH	head and neck cancer	neoplastic (JHU-O28, JHU-O22, JHU-O11, JHU-O29, OFK6/TERT1, FaDu, CAL 27)	2.27	iTRAQ	23665456
PRKDC	head and neck cancer	neoplastic (JHU-O28, JHU-O22, JHU-O11, JHU-O29, OFK6/TERT1, FaDu, CAL 27)	1.02	iTRAQ	23665456

PRNP	head and neck cancer	neoplastic (JHU-O28, JHU-O22, JHU-O11, JHU-O29, OFK6/TERT1, FaDu, CAL 27)	-1.28	iTRAQ	23665456
PROCR	head and neck cancer	neoplastic (JHU-O28, JHU-O22, JHU-O11, JHU-O29, OFK6/TERT1, FaDu, CAL 27)	-1.73	iTRAQ	23665456
PROS1	head and neck cancer	neoplastic (JHU-O28, JHU-O22, JHU-O11, JHU-O29, OFK6/TERT1, FaDu, CAL 27)	1.72	iTRAQ	23665456
PROSC	head and neck cancer	neoplastic (JHU-O28, JHU-O22, JHU-O11, JHU-O29, OFK6/TERT1, FaDu, CAL 27)	-1.83	iTRAQ	23665456
PRPF19	head and neck cancer	neoplastic (JHU-O28, JHU-O22, JHU-O11, JHU-O29, OFK6/TERT1, FaDu, CAL 27)	1.41	iTRAQ	23665456
PRPF40B	head and neck cancer	neoplastic (JHU-O28, JHU-O22, JHU-O11, JHU-O29, OFK6/TERT1, FaDu, CAL 27)	-1.78	iTRAQ	23665456
PRRC1	head and neck cancer	neoplastic (JHU-O28, JHU-O22, JHU-O11, JHU-O29, OFK6/TERT1, FaDu, CAL 27)	-2.25	iTRAQ	23665456
PRSS22	head and neck cancer	neoplastic (JHU-O28, JHU-O22, JHU-O11, JHU-O29, OFK6/TERT1, FaDu, CAL 27)	1.35	iTRAQ	23665456
PRSS23	head and neck cancer	neoplastic (JHU-O28, JHU-O22, JHU-O11, JHU-O29, OFK6/TERT1, FaDu, CAL 27)	1.07	iTRAQ	23665456
PSAT1	head and neck cancer	neoplastic (JHU-O28, JHU-O22, JHU-O11, JHU-O29, OFK6/TERT1, FaDu, CAL 27)	-1.32	iTRAQ	23665456
PSMA1	head and neck cancer	neoplastic (JHU-O28, JHU-O22, JHU-O11, JHU-O29, OFK6/TERT1, FaDu, CAL 27)	2.54	iTRAQ	23665456
PSMA1	head and neck cancer	neoplastic (JHU-O28, JHU-O22, JHU-O11, JHU-O29, OFK6/TERT1, FaDu, CAL 27)	3.05	iTRAQ	23665456
PSMA2	head and neck cancer	neoplastic (JHU-O28, JHU-O22, JHU-O11, JHU-O29, OFK6/TERT1, FaDu, CAL 27)	2.09	iTRAQ	23665456
PSMA3	head and neck cancer	neoplastic (JHU-O28, JHU-O22, JHU-O11, JHU-O29, OFK6/TERT1, FaDu, CAL 27)	1.75	iTRAQ	23665456
PSMA4	head and neck cancer	neoplastic (JHU-O28, JHU-O22, JHU-O11, JHU-O29, OFK6/TERT1, FaDu, CAL 27)	1.42	iTRAQ	23665456
PSMA5	head and neck cancer	neoplastic (JHU-O28, JHU-O22, JHU-O11, JHU-O29, OFK6/TERT1, FaDu, CAL 27)	1.30	iTRAQ	23665456
PSMA6	head and neck cancer	neoplastic (JHU-O28, JHU-O22, JHU-O11, JHU-O29, OFK6/TERT1, FaDu, CAL 27)	-1.73	iTRAQ	23665456
PSMA6	head and neck cancer	neoplastic (JHU-O28, JHU-O22, JHU-O11, JHU-O29, OFK6/TERT1, FaDu, CAL 27)	2.24	iTRAQ	23665456
PSMB1	head and neck cancer	neoplastic (JHU-O28, JHU-O22, JHU-O11, JHU-O29, OFK6/TERT1, FaDu, CAL 27)	1.76	iTRAQ	23665456
PSMB2	head and neck cancer	neoplastic (JHU-O28, JHU-O22, JHU-O11, JHU-O29, OFK6/TERT1, FaDu, CAL 27)	1.70	iTRAQ	23665456
PSMB3	head and neck cancer	neoplastic (JHU-O28, JHU-O22, JHU-O11, JHU-O29, OFK6/TERT1, FaDu, CAL 27)	1.64	iTRAQ	23665456
PSMB4	head and neck cancer	neoplastic (JHU-O28, JHU-O22, JHU-O11, JHU-O29, OFK6/TERT1, FaDu, CAL 27)	1.67	iTRAQ	23665456
PSMB5	head and neck cancer	neoplastic (JHU-O28, JHU-O22, JHU-O11, JHU-O29, OFK6/TERT1, FaDu, CAL 27)	1.54	iTRAQ	23665456
PSMB5	head and neck cancer	neoplastic (JHU-O28, JHU-O22, JHU-O11, JHU-O29, OFK6/TERT1, FaDu, CAL 27)	2.58	iTRAQ	23665456
PSMB7	head and neck cancer	neoplastic (JHU-O28, JHU-O22, JHU-O11, JHU-O29, OFK6/TERT1, FaDu, CAL 27)	1.81	iTRAQ	23665456
PSMB8	head and neck cancer	neoplastic (JHU-O28, JHU-O22, JHU-O11, JHU-O29, OFK6/TERT1, FaDu, CAL 27)	1.59	iTRAQ	23665456
PSMB9	head and neck cancer	neoplastic (JHU-O28, JHU-O22, JHU-O11, JHU-O29, OFK6/TERT1, FaDu, CAL 27)	1.18	iTRAQ	23665456
PSMC1	head and neck cancer	neoplastic (JHU-O28, JHU-O22, JHU-O11, JHU-O29, OFK6/TERT1, FaDu, CAL 27)	1.50	iTRAQ	23665456
PSMC2	head and neck cancer	neoplastic (JHU-O28, JHU-O22, JHU-O11, JHU-O29, OFK6/TERT1, FaDu, CAL 27)	1.67	iTRAQ	23665456
PSMC4	head and neck cancer	neoplastic (JHU-O28, JHU-O22, JHU-O11, JHU-O29, OFK6/TERT1, FaDu, CAL 27)	1.16	iTRAQ	23665456
PSMC5	head and neck cancer	neoplastic (JHU-O28, JHU-O22, JHU-O11, JHU-O29, OFK6/TERT1, FaDu, CAL 27)	1.19	iTRAQ	23665456
PSMD11	head and neck cancer	neoplastic (JHU-O28, JHU-O22, JHU-O11, JHU-O29, OFK6/TERT1, FaDu, CAL 27)	1.11	iTRAQ	23665456
PSMD2	head and neck cancer	neoplastic (JHU-O28, JHU-O22, JHU-O11, JHU-O29, OFK6/TERT1, FaDu, CAL 27)	1.65	iTRAQ	23665456
PSMD3	head and neck cancer	neoplastic (JHU-O28, JHU-O22, JHU-O11, JHU-O29, OFK6/TERT1, FaDu, CAL 27)	1.53	iTRAQ	23665456
PSMD6	head and neck cancer	neoplastic (JHU-O28, JHU-O22, JHU-O11, JHU-O29, OFK6/TERT1, FaDu, CAL 27)	1.65	iTRAQ	23665456
PSMD7	head and neck cancer	neoplastic (JHU-O28, JHU-O22, JHU-O11, JHU-O29, OFK6/TERT1, FaDu, CAL 27)	1.43	iTRAQ	23665456
PSME1	head and neck cancer	neoplastic (JHU-O28, JHU-O22, JHU-O11, JHU-O29, OFK6/TERT1, FaDu, CAL 27)	-1.15	iTRAQ	23665456
PSME3	head and neck cancer	neoplastic (JHU-O28, JHU-O22, JHU-O11, JHU-O29, OFK6/TERT1, FaDu, CAL 27)	1.16	iTRAQ	23665456
PSMF1	head and neck cancer	neoplastic (JHU-O28, JHU-O22, JHU-O11, JHU-O29, OFK6/TERT1, FaDu, CAL 27)	-1.15	iTRAQ	23665456
PSMG4	head and neck cancer	neoplastic (JHU-O28, JHU-O22, JHU-O11, JHU-O29, OFK6/TERT1, FaDu, CAL 27)	1.37	iTRAQ	23665456
PTBP2	head and neck cancer	neoplastic (JHU-O28, JHU-O22, JHU-O11, JHU-O29, OFK6/TERT1, FaDu, CAL 27)	-2.12	iTRAQ	23665456
PTGES3	head and neck cancer	neoplastic (JHU-O28, JHU-O22, JHU-O11, JHU-O29, OFK6/TERT1, FaDu, CAL 27)	2.71	iTRAQ	23665456
PTGFRN	head and neck cancer	neoplastic (JHU-O28, JHU-O22, JHU-O11, JHU-O29, OFK6/TERT1, FaDu, CAL 27)	1.19	iTRAQ	23665456

PTGR1	head and neck cancer	neoplastic (JHU-O28, JHU-O22, JHU-O11, JHU-O29, OFK6/TERT1, FaDu, CAL 27)	-1	iTRAQ	23665456
PTK7	head and neck cancer	neoplastic (JHU-O28, JHU-O22, JHU-O11, JHU-O29, OFK6/TERT1, FaDu, CAL 27)	-1.21	iTRAQ	23665456
PTMA	head and neck cancer	neoplastic (JHU-O28, JHU-O22, JHU-O11, JHU-O29, OFK6/TERT1, FaDu, CAL 27)	2.25	iTRAQ	23665456
PTPLAD1	head and neck cancer	neoplastic (JHU-O28, JHU-O22, JHU-O11, JHU-O29, OFK6/TERT1, FaDu, CAL 27)	2.10	iTRAQ	23665456
PTPN12	head and neck cancer	neoplastic (JHU-O28, JHU-O22, JHU-O11, JHU-O29, OFK6/TERT1, FaDu, CAL 27)	-1.68	iTRAQ	23665456
PTPRG	head and neck cancer	neoplastic (JHU-O28, JHU-O22, JHU-O11, JHU-O29, OFK6/TERT1, FaDu, CAL 27)	-1.68	iTRAQ	23665456
PTPRJ	head and neck cancer	neoplastic (JHU-O28, JHU-O22, JHU-O11, JHU-O29, OFK6/TERT1, FaDu, CAL 27)	2.54	iTRAQ	23665456
PTPRK	head and neck cancer	neoplastic (JHU-O28, JHU-O22, JHU-O11, JHU-O29, OFK6/TERT1, FaDu, CAL 27)	-1.15	iTRAQ	23665456
PTPRS	head and neck cancer	neoplastic (JHU-O28, JHU-O22, JHU-O11, JHU-O29, OFK6/TERT1, FaDu, CAL 27)	1.11	iTRAQ	23665456
PTRF	head and neck cancer	neoplastic (JHU-O28, JHU-O22, JHU-O11, JHU-O29, OFK6/TERT1, FaDu, CAL 27)	1.99	iTRAQ	23665456
PTX3	head and neck cancer	neoplastic (JHU-O28, JHU-O22, JHU-O11, JHU-O29, OFK6/TERT1, FaDu, CAL 27)	1.29	iTRAQ	23665456
PVRL1	head and neck cancer	neoplastic (JHU-O28, JHU-O22, JHU-O11, JHU-O29, OFK6/TERT1, FaDu, CAL 27)	-1.68	iTRAQ	23665456
PWP1	head and neck cancer	neoplastic (JHU-O28, JHU-O22, JHU-O11, JHU-O29, OFK6/TERT1, FaDu, CAL 27)	2.26	iTRAQ	23665456
PXN	head and neck cancer	neoplastic (JHU-O28, JHU-O22, JHU-O11, JHU-O29, OFK6/TERT1, FaDu, CAL 27)	-1.73	iTRAQ	23665456
PYCARD	head and neck cancer	neoplastic (JHU-O28, JHU-O22, JHU-O11, JHU-O29, OFK6/TERT1, FaDu, CAL 27)	-2.64	iTRAQ	23665456
PYGB	head and neck cancer	neoplastic (JHU-O28, JHU-O22, JHU-O11, JHU-O29, OFK6/TERT1, FaDu, CAL 27)	-1	iTRAQ	23665456
PYGL	head and neck cancer	neoplastic (JHU-O28, JHU-O22, JHU-O11, JHU-O29, OFK6/TERT1, FaDu, CAL 27)	-1.32	iTRAQ	23665456
PYGL	head and neck cancer	neoplastic (JHU-O28, JHU-O22, JHU-O11, JHU-O29, OFK6/TERT1, FaDu, CAL 27)	-1.55	iTRAQ	23665456
PYGM	head and neck cancer	neoplastic (JHU-O28, JHU-O22, JHU-O11, JHU-O29, OFK6/TERT1, FaDu, CAL 27)	-1.73	iTRAQ	23665456
PZP	head and neck cancer	neoplastic (JHU-O28, JHU-O22, JHU-O11, JHU-O29, OFK6/TERT1, FaDu, CAL 27)	1.02	iTRAQ	23665456
QPCT	head and neck cancer	neoplastic (JHU-O28, JHU-O22, JHU-O11, JHU-O29, OFK6/TERT1, FaDu, CAL 27)	1.20	iTRAQ	23665456
RAB10	head and neck cancer	neoplastic (JHU-O28, JHU-O22, JHU-O11, JHU-O29, OFK6/TERT1, FaDu, CAL 27)	1.31	iTRAQ	23665456
RAB11B	head and neck cancer	neoplastic (JHU-O28, JHU-O22, JHU-O11, JHU-O29, OFK6/TERT1, FaDu, CAL 27)	-2.32	iTRAQ	23665456
RAB1B	head and neck cancer	neoplastic (JHU-O28, JHU-O22, JHU-O11, JHU-O29, OFK6/TERT1, FaDu, CAL 27)	-1.35	iTRAQ	23665456
RAB5C	head and neck cancer	neoplastic (JHU-O28, JHU-O22, JHU-O11, JHU-O29, OFK6/TERT1, FaDu, CAL 27)	1.18	iTRAQ	23665456
RAD21L1	head and neck cancer	neoplastic (JHU-O28, JHU-O22, JHU-O11, JHU-O29, OFK6/TERT1, FaDu, CAL 27)	1.44	iTRAQ	23665456
RALY	head and neck cancer	neoplastic (JHU-O28, JHU-O22, JHU-O11, JHU-O29, OFK6/TERT1, FaDu, CAL 27)	1.91	iTRAQ	23665456
RANBP6	head and neck cancer	neoplastic (JHU-O28, JHU-O22, JHU-O11, JHU-O29, OFK6/TERT1, FaDu, CAL 27)	1.16	iTRAQ	23665456
RANGAP1	head and neck cancer	neoplastic (JHU-O28, JHU-O22, JHU-O11, JHU-O29, OFK6/TERT1, FaDu, CAL 27)	1.21	iTRAQ	23665456
RASA3	head and neck cancer	neoplastic (JHU-O28, JHU-O22, JHU-O11, JHU-O29, OFK6/TERT1, FaDu, CAL 27)	-1	iTRAQ	23665456
RBBP7	head and neck cancer	neoplastic (JHU-O28, JHU-O22, JHU-O11, JHU-O29, OFK6/TERT1, FaDu, CAL 27)	1.37	iTRAQ	23665456
RBBP9	head and neck cancer	neoplastic (JHU-O28, JHU-O22, JHU-O11, JHU-O29, OFK6/TERT1, FaDu, CAL 27)	-1.59	iTRAQ	23665456
RBM8A	head and neck cancer	neoplastic (JHU-O28, JHU-O22, JHU-O11, JHU-O29, OFK6/TERT1, FaDu, CAL 27)	1.83	iTRAQ	23665456
RCC1	head and neck cancer	neoplastic (JHU-O28, JHU-O22, JHU-O11, JHU-O29, OFK6/TERT1, FaDu, CAL 27)	1.20	iTRAQ	23665456
RCN1	head and neck cancer	neoplastic (JHU-O28, JHU-O22, JHU-O11, JHU-O29, OFK6/TERT1, FaDu, CAL 27)	-1.12	iTRAQ	23665456
RCN3	head and neck cancer	neoplastic (JHU-O28, JHU-O22, JHU-O11, JHU-O29, OFK6/TERT1, FaDu, CAL 27)	-2.25	iTRAQ	23665456
RECQL	head and neck cancer	neoplastic (JHU-O28, JHU-O22, JHU-O11, JHU-O29, OFK6/TERT1, FaDu, CAL 27)	1.34	iTRAQ	23665456
REXO2	head and neck cancer	neoplastic (JHU-O28, JHU-O22, JHU-O11, JHU-O29, OFK6/TERT1, FaDu, CAL 27)	-1.39	iTRAQ	23665456
RGS22	head and neck cancer	neoplastic (JHU-O28, JHU-O22, JHU-O11, JHU-O29, OFK6/TERT1, FaDu, CAL 27)	-1.43	iTRAQ	23665456
RHOA	head and neck cancer	neoplastic (JHU-O28, JHU-O22, JHU-O11, JHU-O29, OFK6/TERT1, FaDu, CAL 27)	-1.02	iTRAQ	23665456
RNASE4	head and neck cancer	neoplastic (JHU-O28, JHU-O22, JHU-O11, JHU-O29, OFK6/TERT1, FaDu, CAL 27)	1.15	iTRAQ	23665456
RNH1	head and neck cancer	neoplastic (JHU-O28, JHU-O22, JHU-O11, JHU-O29, OFK6/TERT1, FaDu, CAL 27)	-1.28	iTRAQ	23665456
RNPEP	head and neck cancer	neoplastic (JHU-O28, JHU-O22, JHU-O11, JHU-O29, OFK6/TERT1, FaDu, CAL 27)	-1.32	iTRAQ	23665456

RPAP3	head and neck cancer	neoplastic (JHU-O28, JHU-O22, JHU-O11, JHU-O29, OFK6/TERT1, FaDu, CAL 27)	-1.39	iTRAQ	23665456
RPE	head and neck cancer	neoplastic (JHU-O28, JHU-O22, JHU-O11, JHU-O29, OFK6/TERT1, FaDu, CAL 27)	-1.25	iTRAQ	23665456
RPL10	head and neck cancer	neoplastic (JHU-O28, JHU-O22, JHU-O11, JHU-O29, OFK6/TERT1, FaDu, CAL 27)	1.04	iTRAQ	23665456
RPL10A	head and neck cancer	neoplastic (JHU-O28, JHU-O22, JHU-O11, JHU-O29, OFK6/TERT1, FaDu, CAL 27)	2.67	iTRAQ	23665456
RPL12	head and neck cancer	neoplastic (JHU-O28, JHU-O22, JHU-O11, JHU-O29, OFK6/TERT1, FaDu, CAL 27)	1.36	iTRAQ	23665456
RPL17	head and neck cancer	neoplastic (JHU-O28, JHU-O22, JHU-O11, JHU-O29, OFK6/TERT1, FaDu, CAL 27)	1.63	iTRAQ	23665456
RPL24	head and neck cancer	neoplastic (JHU-O28, JHU-O22, JHU-O11, JHU-O29, OFK6/TERT1, FaDu, CAL 27)	1.07	iTRAQ	23665456
RPL26	head and neck cancer	neoplastic (JHU-O28, JHU-O22, JHU-O11, JHU-O29, OFK6/TERT1, FaDu, CAL 27)	1.74	iTRAQ	23665456
RPL28	head and neck cancer	neoplastic (JHU-O28, JHU-O22, JHU-O11, JHU-O29, OFK6/TERT1, FaDu, CAL 27)	2.38	iTRAQ	23665456
RPL29	head and neck cancer	neoplastic (JHU-O28, JHU-O22, JHU-O11, JHU-O29, OFK6/TERT1, FaDu, CAL 27)	4.13	iTRAQ	23665456
RPL5	head and neck cancer	neoplastic (JHU-O28, JHU-O22, JHU-O11, JHU-O29, OFK6/TERT1, FaDu, CAL 27)	1.07	iTRAQ	23665456
RPL6	head and neck cancer	neoplastic (JHU-O28, JHU-O22, JHU-O11, JHU-O29, OFK6/TERT1, FaDu, CAL 27)	2.01	iTRAQ	23665456
RPL7A	head and neck cancer	neoplastic (JHU-O28, JHU-O22, JHU-O11, JHU-O29, OFK6/TERT1, FaDu, CAL 27)	1.30	iTRAQ	23665456
RPLP0	head and neck cancer	neoplastic (JHU-O28, JHU-O22, JHU-O11, JHU-O29, OFK6/TERT1, FaDu, CAL 27)	1.09	iTRAQ	23665456
RPLP1	head and neck cancer	neoplastic (JHU-O28, JHU-O22, JHU-O11, JHU-O29, OFK6/TERT1, FaDu, CAL 27)	1.80	iTRAQ	23665456
RPLP1	head and neck cancer	neoplastic (JHU-O28, JHU-O22, JHU-O11, JHU-O29, OFK6/TERT1, FaDu, CAL 27)	1.92	iTRAQ	23665456
RPLP2	head and neck cancer	neoplastic (JHU-O28, JHU-O22, JHU-O11, JHU-O29, OFK6/TERT1, FaDu, CAL 27)	1.16	iTRAQ	23665456
RPN2	head and neck cancer	neoplastic (JHU-O28, JHU-O22, JHU-O11, JHU-O29, OFK6/TERT1, FaDu, CAL 27)	1.62	iTRAQ	23665456
RPRD1B	head and neck cancer	neoplastic (JHU-O28, JHU-O22, JHU-O11, JHU-O29, OFK6/TERT1, FaDu, CAL 27)	3.21	iTRAQ	23665456
RPS13	head and neck cancer	neoplastic (JHU-O28, JHU-O22, JHU-O11, JHU-O29, OFK6/TERT1, FaDu, CAL 27)	1.73	iTRAQ	23665456
RPS15	head and neck cancer	neoplastic (JHU-O28, JHU-O22, JHU-O11, JHU-O29, OFK6/TERT1, FaDu, CAL 27)	1.58	iTRAQ	23665456
RPS15A	head and neck cancer	neoplastic (JHU-O28, JHU-O22, JHU-O11, JHU-O29, OFK6/TERT1, FaDu, CAL 27)	1.23	iTRAQ	23665456
RPS24	head and neck cancer	neoplastic (JHU-O28, JHU-O22, JHU-O11, JHU-O29, OFK6/TERT1, FaDu, CAL 27)	1.11	iTRAQ	23665456
RPS27	head and neck cancer	neoplastic (JHU-O28, JHU-O22, JHU-O11, JHU-O29, OFK6/TERT1, FaDu, CAL 27)	1.75	iTRAQ	23665456
RPS28	head and neck cancer	neoplastic (JHU-O28, JHU-O22, JHU-O11, JHU-O29, OFK6/TERT1, FaDu, CAL 27)	-1.28	iTRAQ	23665456
RPS6	head and neck cancer	neoplastic (JHU-O28, JHU-O22, JHU-O11, JHU-O29, OFK6/TERT1, FaDu, CAL 27)	2.77	iTRAQ	23665456
RPS8	head and neck cancer	neoplastic (JHU-O28, JHU-O22, JHU-O11, JHU-O29, OFK6/TERT1, FaDu, CAL 27)	1.59	iTRAQ	23665456
RPS9	head and neck cancer	neoplastic (JHU-O28, JHU-O22, JHU-O11, JHU-O29, OFK6/TERT1, FaDu, CAL 27)	1.05	iTRAQ	23665456
RSL1D1	head and neck cancer	neoplastic (JHU-O28, JHU-O22, JHU-O11, JHU-O29, OFK6/TERT1, FaDu, CAL 27)	3.31	iTRAQ	23665456
RUFY1	head and neck cancer	neoplastic (JHU-O28, JHU-O22, JHU-O11, JHU-O29, OFK6/TERT1, FaDu, CAL 27)	-1.25	iTRAQ	23665456
RUVBL1	head and neck cancer	neoplastic (JHU-O28, JHU-O22, JHU-O11, JHU-O29, OFK6/TERT1, FaDu, CAL 27)	1.08	iTRAQ	23665456
RUVBL2	head and neck cancer	neoplastic (JHU-O28, JHU-O22, JHU-O11, JHU-O29, OFK6/TERT1, FaDu, CAL 27)	1.44	iTRAQ	23665456
RWDD4	head and neck cancer	neoplastic (JHU-O28, JHU-O22, JHU-O11, JHU-O29, OFK6/TERT1, FaDu, CAL 27)	-1.05	iTRAQ	23665456
S100A11	head and neck cancer	neoplastic (JHU-O28, JHU-O22, JHU-O11, JHU-O29, OFK6/TERT1, FaDu, CAL 27)	-2.55	iTRAQ	23665456
S100A14	head and neck cancer	neoplastic (JHU-O28, JHU-O22, JHU-O11, JHU-O29, OFK6/TERT1, FaDu, CAL 27)	-2.25	iTRAQ	23665456
S100A4	head and neck cancer	neoplastic (JHU-O28, JHU-O22, JHU-O11, JHU-O29, OFK6/TERT1, FaDu, CAL 27)	-1.21	iTRAQ	23665456
SAP18	head and neck cancer	neoplastic (JHU-O28, JHU-O22, JHU-O11, JHU-O29, OFK6/TERT1, FaDu, CAL 27)	1.18	iTRAQ	23665456
SCAMP1	head and neck cancer	neoplastic (JHU-O28, JHU-O22, JHU-O11, JHU-O29, OFK6/TERT1, FaDu, CAL 27)	-1.59	iTRAQ	23665456
SCG2	head and neck cancer	neoplastic (JHU-O28, JHU-O22, JHU-O11, JHU-O29, OFK6/TERT1, FaDu, CAL 27)	-2.25	iTRAQ	23665456
SCG3	head and neck cancer	neoplastic (JHU-O28, JHU-O22, JHU-O11, JHU-O29, OFK6/TERT1, FaDu, CAL 27)	-1.15	iTRAQ	23665456
SCRN2	head and neck cancer	neoplastic (JHU-O28, JHU-O22, JHU-O11, JHU-O29, OFK6/TERT1, FaDu, CAL 27)	-1.39	iTRAQ	23665456
SDC4	head and neck cancer	neoplastic (JHU-O28, JHU-O22, JHU-O11, JHU-O29, OFK6/TERT1, FaDu, CAL 27)	1.70	iTRAQ	23665456
SDC4	head and neck cancer	neoplastic (JHU-O28, JHU-O22, JHU-O11, JHU-O29, OFK6/TERT1, FaDu, CAL 27)	3.44	iTRAQ	23665456

SDCBP	head and neck cancer	neoplastic (JHU-O28, JHU-O22, JHU-O11, JHU-O29, OFK6/TERT1, FaDu, CAL 27)	1.66	iTRAQ	23665456
SDHAF2	head and neck cancer	neoplastic (JHU-O28, JHU-O22, JHU-O11, JHU-O29, OFK6/TERT1, FaDu, CAL 27)	-2.18	iTRAQ	23665456
SEC16A	head and neck cancer	neoplastic (JHU-O28, JHU-O22, JHU-O11, JHU-O29, OFK6/TERT1, FaDu, CAL 27)	-1.32	iTRAQ	23665456
SECTM1	head and neck cancer	neoplastic (JHU-O28, JHU-O22, JHU-O11, JHU-O29, OFK6/TERT1, FaDu, CAL 27)	1.80	iTRAQ	23665456
SEPT10	head and neck cancer	neoplastic (JHU-O28, JHU-O22, JHU-O11, JHU-O29, OFK6/TERT1, FaDu, CAL 27)	1.35	iTRAQ	23665456
SEPT11	head and neck cancer	neoplastic (JHU-O28, JHU-O22, JHU-O11, JHU-O29, OFK6/TERT1, FaDu, CAL 27)	1.47	iTRAQ	23665456
SEPT2	head and neck cancer	neoplastic (JHU-O28, JHU-O22, JHU-O11, JHU-O29, OFK6/TERT1, FaDu, CAL 27)	1.55	iTRAQ	23665456
SEPT7	head and neck cancer	neoplastic (JHU-O28, JHU-O22, JHU-O11, JHU-O29, OFK6/TERT1, FaDu, CAL 27)	1.83	iTRAQ	23665456
SEPT9	head and neck cancer	neoplastic (JHU-O28, JHU-O22, JHU-O11, JHU-O29, OFK6/TERT1, FaDu, CAL 27)	1.66	iTRAQ	23665456
SEPW1	head and neck cancer	neoplastic (JHU-O28, JHU-O22, JHU-O11, JHU-O29, OFK6/TERT1, FaDu, CAL 27)	-1.55	iTRAQ	23665456
SERPINA1	head and neck cancer	neoplastic (JHU-O28, JHU-O22, JHU-O11, JHU-O29, OFK6/TERT1, FaDu, CAL 27)	-2.39	iTRAQ	23665456
SERPINA12	head and neck cancer	neoplastic (JHU-O28, JHU-O22, JHU-O11, JHU-O29, OFK6/TERT1, FaDu, CAL 27)	9.07	iTRAQ	23665456
SERPINA3	head and neck cancer	neoplastic (JHU-O28, JHU-O22, JHU-O11, JHU-O29, OFK6/TERT1, FaDu, CAL 27)	1.25	iTRAQ	23665456
SERPINA7	head and neck cancer	neoplastic (JHU-O28, JHU-O22, JHU-O11, JHU-O29, OFK6/TERT1, FaDu, CAL 27)	1.53	iTRAQ	23665456
SERPINB1	head and neck cancer	neoplastic (JHU-O28, JHU-O22, JHU-O11, JHU-O29, OFK6/TERT1, FaDu, CAL 27)	-1.12	iTRAQ	23665456
SERPINB1	head and neck cancer	neoplastic (JHU-O28, JHU-O22, JHU-O11, JHU-O29, OFK6/TERT1, FaDu, CAL 27)	2.63	iTRAQ	23665456
SERPINB2	head and neck cancer	neoplastic (JHU-O28, JHU-O22, JHU-O11, JHU-O29, OFK6/TERT1, FaDu, CAL 27)	-2.05	iTRAQ	23665456
SERPINB4	head and neck cancer	neoplastic (JHU-O28, JHU-O22, JHU-O11, JHU-O29, OFK6/TERT1, FaDu, CAL 27)	1.07	iTRAQ	23665456
SERPINB6	head and neck cancer	neoplastic (JHU-O28, JHU-O22, JHU-O11, JHU-O29, OFK6/TERT1, FaDu, CAL 27)	-1.43	iTRAQ	23665456
SERPINB7	head and neck cancer	neoplastic (JHU-O28, JHU-O22, JHU-O11, JHU-O29, OFK6/TERT1, FaDu, CAL 27)	-1.68	iTRAQ	23665456
SERPINC1	head and neck cancer	neoplastic (JHU-O28, JHU-O22, JHU-O11, JHU-O29, OFK6/TERT1, FaDu, CAL 27)	2.57	iTRAQ	23665456
SERPINC1	head and neck cancer	neoplastic (JHU-O28, JHU-O22, JHU-O11, JHU-O29, OFK6/TERT1, FaDu, CAL 27)	3.36	iTRAQ	23665456
SERPINE1	head and neck cancer	neoplastic (JHU-O28, JHU-O22, JHU-O11, JHU-O29, OFK6/TERT1, FaDu, CAL 27)	-1.39	iTRAQ	23665456
SERPINE1	head and neck cancer	neoplastic (JHU-O28, JHU-O22, JHU-O11, JHU-O29, OFK6/TERT1, FaDu, CAL 27)	2.23	iTRAQ	23665456
SERPINF2	head and neck cancer	neoplastic (JHU-O28, JHU-O22, JHU-O11, JHU-O29, OFK6/TERT1, FaDu, CAL 27)	1.31	iTRAQ	23665456
SERPINH1	head and neck cancer	neoplastic (JHU-O28, JHU-O22, JHU-O11, JHU-O29, OFK6/TERT1, FaDu, CAL 27)	1.09	iTRAQ	23665456
SET	head and neck cancer	neoplastic (JHU-O28, JHU-O22, JHU-O11, JHU-O29, OFK6/TERT1, FaDu, CAL 27)	2.85	iTRAQ	23665456
SF3A2	head and neck cancer	neoplastic (JHU-O28, JHU-O22, JHU-O11, JHU-O29, OFK6/TERT1, FaDu, CAL 27)	2.89	iTRAQ	23665456
SF3A3	head and neck cancer	neoplastic (JHU-O28, JHU-O22, JHU-O11, JHU-O29, OFK6/TERT1, FaDu, CAL 27)	2.62	iTRAQ	23665456
SF3B3	head and neck cancer	neoplastic (JHU-O28, JHU-O22, JHU-O11, JHU-O29, OFK6/TERT1, FaDu, CAL 27)	2.02	iTRAQ	23665456
SF3B5	head and neck cancer	neoplastic (JHU-O28, JHU-O22, JHU-O11, JHU-O29, OFK6/TERT1, FaDu, CAL 27)	1.60	iTRAQ	23665456
SFN	head and neck cancer	neoplastic (JHU-O28, JHU-O22, JHU-O11, JHU-O29, OFK6/TERT1, FaDu, CAL 27)	-2	iTRAQ	23665456
SH3BGRL	head and neck cancer	neoplastic (JHU-O28, JHU-O22, JHU-O11, JHU-O29, OFK6/TERT1, FaDu, CAL 27)	-1.64	iTRAQ	23665456
SH3KBP1	head and neck cancer	neoplastic (JHU-O28, JHU-O22, JHU-O11, JHU-O29, OFK6/TERT1, FaDu, CAL 27)	-1.64	iTRAQ	23665456
SHANK1	head and neck cancer	neoplastic (JHU-O28, JHU-O22, JHU-O11, JHU-O29, OFK6/TERT1, FaDu, CAL 27)	1.87	iTRAQ	23665456
SHROOM3	head and neck cancer	neoplastic (JHU-O28, JHU-O22, JHU-O11, JHU-O29, OFK6/TERT1, FaDu, CAL 27)	3.00	iTRAQ	23665456
SIL1	head and neck cancer	neoplastic (JHU-O28, JHU-O22, JHU-O11, JHU-O29, OFK6/TERT1, FaDu, CAL 27)	-1.25	iTRAQ	23665456
SKIV2L2	head and neck cancer	neoplastic (JHU-O28, JHU-O22, JHU-O11, JHU-O29, OFK6/TERT1, FaDu, CAL 27)	1.63	iTRAQ	23665456
SLC16A3	head and neck cancer	neoplastic (JHU-O28, JHU-O22, JHU-O11, JHU-O29, OFK6/TERT1, FaDu, CAL 27)	-1.68	iTRAQ	23665456
SLC1A5	head and neck cancer	neoplastic (JHU-O28, JHU-O22, JHU-O11, JHU-O29, OFK6/TERT1, FaDu, CAL 27)	1.09	iTRAQ	23665456
SLC2A1	head and neck cancer	neoplastic (JHU-O28, JHU-O22, JHU-O11, JHU-O29, OFK6/TERT1, FaDu, CAL 27)	1.89	iTRAQ	23665456
SLC34A3	head and neck cancer	neoplastic (JHU-O28, JHU-O22, JHU-O11, JHU-O29, OFK6/TERT1, FaDu, CAL 27)	8.23	iTRAQ	23665456
SLC44A2	head and neck cancer	neoplastic (JHU-O28, JHU-O22, JHU-O11, JHU-O29, OFK6/TERT1, FaDu, CAL 27)	2.80	iTRAQ	23665456

SLC7A5	head and neck cancer	neoplastic (JHU-O28, JHU-O22, JHU-O11, JHU-O29, OFK6/TERT1, FaDu, CAL 27)	2.22	iTRAQ	23665456
SLPI	head and neck cancer	neoplastic (JHU-O28, JHU-O22, JHU-O11, JHU-O29, OFK6/TERT1, FaDu, CAL 27)	3.00	iTRAQ	23665456
SLTM	head and neck cancer	neoplastic (JHU-O28, JHU-O22, JHU-O11, JHU-O29, OFK6/TERT1, FaDu, CAL 27)	2.45	iTRAQ	23665456
SMARCC1	head and neck cancer	neoplastic (JHU-O28, JHU-O22, JHU-O11, JHU-O29, OFK6/TERT1, FaDu, CAL 27)	2.90	iTRAQ	23665456
SMC1A	head and neck cancer	neoplastic (JHU-O28, JHU-O22, JHU-O11, JHU-O29, OFK6/TERT1, FaDu, CAL 27)	-2.64	iTRAQ	23665456
SMC3	head and neck cancer	neoplastic (JHU-O28, JHU-O22, JHU-O11, JHU-O29, OFK6/TERT1, FaDu, CAL 27)	1.38	iTRAQ	23665456
SNCA	head and neck cancer	neoplastic (JHU-O28, JHU-O22, JHU-O11, JHU-O29, OFK6/TERT1, FaDu, CAL 27)	-2.55	iTRAQ	23665456
SNRNP70	head and neck cancer	neoplastic (JHU-O28, JHU-O22, JHU-O11, JHU-O29, OFK6/TERT1, FaDu, CAL 27)	1.38	iTRAQ	23665456
SNRPB	head and neck cancer	neoplastic (JHU-O28, JHU-O22, JHU-O11, JHU-O29, OFK6/TERT1, FaDu, CAL 27)	2.54	iTRAQ	23665456
SNRPD1	head and neck cancer	neoplastic (JHU-O28, JHU-O22, JHU-O11, JHU-O29, OFK6/TERT1, FaDu, CAL 27)	1.50	iTRAQ	23665456
SNRPD2	head and neck cancer	neoplastic (JHU-O28, JHU-O22, JHU-O11, JHU-O29, OFK6/TERT1, FaDu, CAL 27)	1.70	iTRAQ	23665456
SNRPD3	head and neck cancer	neoplastic (JHU-O28, JHU-O22, JHU-O11, JHU-O29, OFK6/TERT1, FaDu, CAL 27)	3.06	iTRAQ	23665456
SNRPE	head and neck cancer	neoplastic (JHU-O28, JHU-O22, JHU-O11, JHU-O29, OFK6/TERT1, FaDu, CAL 27)	2.94	iTRAQ	23665456
SNRPF	head and neck cancer	neoplastic (JHU-O28, JHU-O22, JHU-O11, JHU-O29, OFK6/TERT1, FaDu, CAL 27)	2.02	iTRAQ	23665456
SNW1	head and neck cancer	neoplastic (JHU-O28, JHU-O22, JHU-O11, JHU-O29, OFK6/TERT1, FaDu, CAL 27)	-1.28	iTRAQ	23665456
SNX18	head and neck cancer	neoplastic (JHU-O28, JHU-O22, JHU-O11, JHU-O29, OFK6/TERT1, FaDu, CAL 27)	-1.15	iTRAQ	23665456
SOD2	head and neck cancer	neoplastic (JHU-O28, JHU-O22, JHU-O11, JHU-O29, OFK6/TERT1, FaDu, CAL 27)	-1.02	iTRAQ	23665456
SOD2	head and neck cancer	neoplastic (JHU-O28, JHU-O22, JHU-O11, JHU-O29, OFK6/TERT1, FaDu, CAL 27)	-1.55	iTRAQ	23665456
SORT1	head and neck cancer	neoplastic (JHU-O28, JHU-O22, JHU-O11, JHU-O29, OFK6/TERT1, FaDu, CAL 27)	1.99	iTRAQ	23665456
SPG20	head and neck cancer	neoplastic (JHU-O28, JHU-O22, JHU-O11, JHU-O29, OFK6/TERT1, FaDu, CAL 27)	-1.18	iTRAQ	23665456
SPINT1	head and neck cancer	neoplastic (JHU-O28, JHU-O22, JHU-O11, JHU-O29, OFK6/TERT1, FaDu, CAL 27)	-1.73	iTRAQ	23665456
SPOCK1	head and neck cancer	neoplastic (JHU-O28, JHU-O22, JHU-O11, JHU-O29, OFK6/TERT1, FaDu, CAL 27)	1.83	iTRAQ	23665456
SPON2	head and neck cancer	neoplastic (JHU-O28, JHU-O22, JHU-O11, JHU-O29, OFK6/TERT1, FaDu, CAL 27)	2.56	iTRAQ	23665456
SPP1	head and neck cancer	neoplastic (JHU-O28, JHU-O22, JHU-O11, JHU-O29, OFK6/TERT1, FaDu, CAL 27)	2.78	iTRAQ	23665456
SPTAN1	head and neck cancer	neoplastic (JHU-O28, JHU-O22, JHU-O11, JHU-O29, OFK6/TERT1, FaDu, CAL 27)	1.32	iTRAQ	23665456
SPTBN1	head and neck cancer	neoplastic (JHU-O28, JHU-O22, JHU-O11, JHU-O29, OFK6/TERT1, FaDu, CAL 27)	2.65	iTRAQ	23665456
SRA1	head and neck cancer	neoplastic (JHU-O28, JHU-O22, JHU-O11, JHU-O29, OFK6/TERT1, FaDu, CAL 27)	-1.55	iTRAQ	23665456
SRI	head and neck cancer	neoplastic (JHU-O28, JHU-O22, JHU-O11, JHU-O29, OFK6/TERT1, FaDu, CAL 27)	-1.83	iTRAQ	23665456
SRM	head and neck cancer	neoplastic (JHU-O28, JHU-O22, JHU-O11, JHU-O29, OFK6/TERT1, FaDu, CAL 27)	-1.18	iTRAQ	23665456
SRRM1	head and neck cancer	neoplastic (JHU-O28, JHU-O22, JHU-O11, JHU-O29, OFK6/TERT1, FaDu, CAL 27)	1.01	iTRAQ	23665456
SRRT	head and neck cancer	neoplastic (JHU-O28, JHU-O22, JHU-O11, JHU-O29, OFK6/TERT1, FaDu, CAL 27)	1.09	iTRAQ	23665456
SRSF1	head and neck cancer	neoplastic (JHU-O28, JHU-O22, JHU-O11, JHU-O29, OFK6/TERT1, FaDu, CAL 27)	2.03	iTRAQ	23665456
SRSF10	head and neck cancer	neoplastic (JHU-O28, JHU-O22, JHU-O11, JHU-O29, OFK6/TERT1, FaDu, CAL 27)	1.36	iTRAQ	23665456
SRSF2	head and neck cancer	neoplastic (JHU-O28, JHU-O22, JHU-O11, JHU-O29, OFK6/TERT1, FaDu, CAL 27)	2.34	iTRAQ	23665456
SRSF3	head and neck cancer	neoplastic (JHU-O28, JHU-O22, JHU-O11, JHU-O29, OFK6/TERT1, FaDu, CAL 27)	1.38	iTRAQ	23665456
SRSF7	head and neck cancer	neoplastic (JHU-O28, JHU-O22, JHU-O11, JHU-O29, OFK6/TERT1, FaDu, CAL 27)	1.74	iTRAQ	23665456
SRSF9	head and neck cancer	neoplastic (JHU-O28, JHU-O22, JHU-O11, JHU-O29, OFK6/TERT1, FaDu, CAL 27)	1.32	iTRAQ	23665456
SSB	head and neck cancer	neoplastic (JHU-O28, JHU-O22, JHU-O11, JHU-O29, OFK6/TERT1, FaDu, CAL 27)	1.51	iTRAQ	23665456
SSRP1	head and neck cancer	neoplastic (JHU-O28, JHU-O22, JHU-O11, JHU-O29, OFK6/TERT1, FaDu, CAL 27)	1.49	iTRAQ	23665456
ST13	head and neck cancer	neoplastic (JHU-O28, JHU-O22, JHU-O11, JHU-O29, OFK6/TERT1, FaDu, CAL 27)	1.94	iTRAQ	23665456
STAT3	head and neck cancer	neoplastic (JHU-O28, JHU-O22, JHU-O11, JHU-O29, OFK6/TERT1, FaDu, CAL 27)	1.21	iTRAQ	23665456
STC2	head and neck cancer	neoplastic (JHU-O28, JHU-O22, JHU-O11, JHU-O29, OFK6/TERT1, FaDu, CAL 27)	1.49	iTRAQ	23665456
STIM1	head and neck cancer	neoplastic (JHU-O28, JHU-O22, JHU-O11, JHU-O29, OFK6/TERT1, FaDu, CAL 27)	-1.47	iTRAQ	23665456

STK10	head and neck cancer	neoplastic (JHU-O28, JHU-O22, JHU-O11, JHU-O29, OFK6/TERT1, FaDu, CAL 27)	-1.64	iTRAQ	23665456
STMN1	head and neck cancer	neoplastic (JHU-O28, JHU-O22, JHU-O11, JHU-O29, OFK6/TERT1, FaDu, CAL 27)	-1.08	iTRAQ	23665456
STMN2	head and neck cancer	neoplastic (JHU-O28, JHU-O22, JHU-O11, JHU-O29, OFK6/TERT1, FaDu, CAL 27)	-1.51	iTRAQ	23665456
STON1	head and neck cancer	neoplastic (JHU-O28, JHU-O22, JHU-O11, JHU-O29, OFK6/TERT1, FaDu, CAL 27)	4.32	iTRAQ	23665456
STX6	head and neck cancer	neoplastic (JHU-O28, JHU-O22, JHU-O11, JHU-O29, OFK6/TERT1, FaDu, CAL 27)	-1.21	iTRAQ	23665456
STX7	head and neck cancer	neoplastic (JHU-O28, JHU-O22, JHU-O11, JHU-O29, OFK6/TERT1, FaDu, CAL 27)	-1	iTRAQ	23665456
SULF2	head and neck cancer	neoplastic (JHU-O28, JHU-O22, JHU-O11, JHU-O29, OFK6/TERT1, FaDu, CAL 27)	1.59	iTRAQ	23665456
SUPT16H	head and neck cancer	neoplastic (JHU-O28, JHU-O22, JHU-O11, JHU-O29, OFK6/TERT1, FaDu, CAL 27)	1.36	iTRAQ	23665456
SYNGR2	head and neck cancer	neoplastic (JHU-O28, JHU-O22, JHU-O11, JHU-O29, OFK6/TERT1, FaDu, CAL 27)	1.15	iTRAQ	23665456
TAB1	head and neck cancer	neoplastic (JHU-O28, JHU-O22, JHU-O11, JHU-O29, OFK6/TERT1, FaDu, CAL 27)	-1.25	iTRAQ	23665456
TAGLN	head and neck cancer	neoplastic (JHU-O28, JHU-O22, JHU-O11, JHU-O29, OFK6/TERT1, FaDu, CAL 27)	-1.18	iTRAQ	23665456
TAGLN2	head and neck cancer	neoplastic (JHU-O28, JHU-O22, JHU-O11, JHU-O29, OFK6/TERT1, FaDu, CAL 27)	-1.88	iTRAQ	23665456
TAGLN3	head and neck cancer	neoplastic (JHU-O28, JHU-O22, JHU-O11, JHU-O29, OFK6/TERT1, FaDu, CAL 27)	-1.32	iTRAQ	23665456
TARBP1	head and neck cancer	neoplastic (JHU-O28, JHU-O22, JHU-O11, JHU-O29, OFK6/TERT1, FaDu, CAL 27)	-1.18	iTRAQ	23665456
TARS	head and neck cancer	neoplastic (JHU-O28, JHU-O22, JHU-O11, JHU-O29, OFK6/TERT1, FaDu, CAL 27)	1.30	iTRAQ	23665456
TAX1BP3	head and neck cancer	neoplastic (JHU-O28, JHU-O22, JHU-O11, JHU-O29, OFK6/TERT1, FaDu, CAL 27)	-1.08	iTRAQ	23665456
TBC1D10B	head and neck cancer	neoplastic (JHU-O28, JHU-O22, JHU-O11, JHU-O29, OFK6/TERT1, FaDu, CAL 27)	-1.12	iTRAQ	23665456
TBC1D23	head and neck cancer	neoplastic (JHU-O28, JHU-O22, JHU-O11, JHU-O29, OFK6/TERT1, FaDu, CAL 27)	-1.32	iTRAQ	23665456
TBCC	head and neck cancer	neoplastic (JHU-O28, JHU-O22, JHU-O11, JHU-O29, OFK6/TERT1, FaDu, CAL 27)	-1.78	iTRAQ	23665456
TCEB2	head and neck cancer	neoplastic (JHU-O28, JHU-O22, JHU-O11, JHU-O29, OFK6/TERT1, FaDu, CAL 27)	-1.08	iTRAQ	23665456
TCOF1	head and neck cancer	neoplastic (JHU-O28, JHU-O22, JHU-O11, JHU-O29, OFK6/TERT1, FaDu, CAL 27)	1.06	iTRAQ	23665456
TCP1	head and neck cancer	neoplastic (JHU-O28, JHU-O22, JHU-O11, JHU-O29, OFK6/TERT1, FaDu, CAL 27)	1.01	iTRAQ	23665456
TCP1	head and neck cancer	neoplastic (JHU-O28, JHU-O22, JHU-O11, JHU-O29, OFK6/TERT1, FaDu, CAL 27)	1.58	iTRAQ	23665456
TES	head and neck cancer	neoplastic (JHU-O28, JHU-O22, JHU-O11, JHU-O29, OFK6/TERT1, FaDu, CAL 27)	-1.02	iTRAQ	23665456
TF	head and neck cancer	neoplastic (JHU-O28, JHU-O22, JHU-O11, JHU-O29, OFK6/TERT1, FaDu, CAL 27)	-2.73	iTRAQ	23665456
TF	head and neck cancer	neoplastic (JHU-O28, JHU-O22, JHU-O11, JHU-O29, OFK6/TERT1, FaDu, CAL 27)	3.90	iTRAQ	23665456
TFPI	head and neck cancer	neoplastic (JHU-O28, JHU-O22, JHU-O11, JHU-O29, OFK6/TERT1, FaDu, CAL 27)	1.41	iTRAQ	23665456
TFRC	head and neck cancer	neoplastic (JHU-O28, JHU-O22, JHU-O11, JHU-O29, OFK6/TERT1, FaDu, CAL 27)	1.56	iTRAQ	23665456
TFRC	head and neck cancer	neoplastic (JHU-O28, JHU-O22, JHU-O11, JHU-O29, OFK6/TERT1, FaDu, CAL 27)	2.10	iTRAQ	23665456
TGFB1	head and neck cancer	neoplastic (JHU-O28, JHU-O22, JHU-O11, JHU-O29, OFK6/TERT1, FaDu, CAL 27)	1.53	iTRAQ	23665456
TGFBI	head and neck cancer	neoplastic (JHU-O28, JHU-O22, JHU-O11, JHU-O29, OFK6/TERT1, FaDu, CAL 27)	3.23	iTRAQ	23665456
TGM2	head and neck cancer	neoplastic (JHU-O28, JHU-O22, JHU-O11, JHU-O29, OFK6/TERT1, FaDu, CAL 27)	2.03	iTRAQ	23665456
TGOLN2	head and neck cancer	neoplastic (JHU-O28, JHU-O22, JHU-O11, JHU-O29, OFK6/TERT1, FaDu, CAL 27)	1.43	iTRAQ	23665456
THBS1	head and neck cancer	neoplastic (JHU-O28, JHU-O22, JHU-O11, JHU-O29, OFK6/TERT1, FaDu, CAL 27)	1.53	iTRAQ	23665456
THBS1	head and neck cancer	neoplastic (JHU-O28, JHU-O22, JHU-O11, JHU-O29, OFK6/TERT1, FaDu, CAL 27)	1.87	iTRAQ	23665456
THRAP3	head and neck cancer	neoplastic (JHU-O28, JHU-O22, JHU-O11, JHU-O29, OFK6/TERT1, FaDu, CAL 27)	1.15	iTRAQ	23665456
THYN1	head and neck cancer	neoplastic (JHU-O28, JHU-O22, JHU-O11, JHU-O29, OFK6/TERT1, FaDu, CAL 27)	-1.25	iTRAQ	23665456
TIAM1	head and neck cancer	neoplastic (JHU-O28, JHU-O22, JHU-O11, JHU-O29, OFK6/TERT1, FaDu, CAL 27)	4.68	iTRAQ	23665456
TIMP1	head and neck cancer	neoplastic (JHU-O28, JHU-O22, JHU-O11, JHU-O29, OFK6/TERT1, FaDu, CAL 27)	-1.21	iTRAQ	23665456
TIMP1	head and neck cancer	neoplastic (JHU-O28, JHU-O22, JHU-O11, JHU-O29, OFK6/TERT1, FaDu, CAL 27)	4.98	iTRAQ	23665456
TIMP2	head and neck cancer	neoplastic (JHU-O28, JHU-O22, JHU-O11, JHU-O29, OFK6/TERT1, FaDu, CAL 27)	1.04	iTRAQ	23665456
TINAGL1	head and neck cancer	neoplastic (JHU-O28, JHU-O22, JHU-O11, JHU-O29, OFK6/TERT1, FaDu, CAL 27)	1.62	iTRAQ	23665456
TK1	head and neck cancer	neoplastic (JHU-O28, JHU-O22, JHU-O11, JHU-O29, OFK6/TERT1, FaDu, CAL 27)	1.23	iTRAQ	23665456

TKT	head and neck cancer	neoplastic (JHU-O28, JHU-O22, JHU-O11, JHU-O29, OFK6/TERT1, FaDu, CAL 27)	-1.43	iTRAQ	23665456
TMSB15B	head and neck cancer	neoplastic (JHU-O28, JHU-O22, JHU-O11, JHU-O29, OFK6/TERT1, FaDu, CAL 27)	-2.47	iTRAQ	23665456
TNC	head and neck cancer	neoplastic (JHU-O28, JHU-O22, JHU-O11, JHU-O29, OFK6/TERT1, FaDu, CAL 27)	1.53	iTRAQ	23665456
TNFAIP2	head and neck cancer	neoplastic (JHU-O28, JHU-O22, JHU-O11, JHU-O29, OFK6/TERT1, FaDu, CAL 27)	2.98	iTRAQ	23665456
TNFRSF10B	head and neck cancer	neoplastic (JHU-O28, JHU-O22, JHU-O11, JHU-O29, OFK6/TERT1, FaDu, CAL 27)	-1.02	iTRAQ	23665456
TNKS1BP1	head and neck cancer	neoplastic (JHU-O28, JHU-O22, JHU-O11, JHU-O29, OFK6/TERT1, FaDu, CAL 27)	-1	iTRAQ	23665456
TNPO3	head and neck cancer	neoplastic (JHU-O28, JHU-O22, JHU-O11, JHU-O29, OFK6/TERT1, FaDu, CAL 27)	1.21	iTRAQ	23665456
TNS3	head and neck cancer	neoplastic (JHU-O28, JHU-O22, JHU-O11, JHU-O29, OFK6/TERT1, FaDu, CAL 27)	-1.08	iTRAQ	23665456
TOM1L1	head and neck cancer	neoplastic (JHU-O28, JHU-O22, JHU-O11, JHU-O29, OFK6/TERT1, FaDu, CAL 27)	-1.83	iTRAQ	23665456
TOP3B	head and neck cancer	neoplastic (JHU-O28, JHU-O22, JHU-O11, JHU-O29, OFK6/TERT1, FaDu, CAL 27)	3	iTRAQ	23665456
TPI1	head and neck cancer	neoplastic (JHU-O28, JHU-O22, JHU-O11, JHU-O29, OFK6/TERT1, FaDu, CAL 27)	-1.25	iTRAQ	23665456
TPM1	head and neck cancer	neoplastic (JHU-O28, JHU-O22, JHU-O11, JHU-O29, OFK6/TERT1, FaDu, CAL 27)	1.76	iTRAQ	23665456
TPM3	head and neck cancer	neoplastic (JHU-O28, JHU-O22, JHU-O11, JHU-O29, OFK6/TERT1, FaDu, CAL 27)	1.33	iTRAQ	23665456
TPP2	head and neck cancer	neoplastic (JHU-O28, JHU-O22, JHU-O11, JHU-O29, OFK6/TERT1, FaDu, CAL 27)	1.15	iTRAQ	23665456
TPR	head and neck cancer	neoplastic (JHU-O28, JHU-O22, JHU-O11, JHU-O29, OFK6/TERT1, FaDu, CAL 27)	1.24	iTRAQ	23665456
TPT1	head and neck cancer	neoplastic (JHU-O28, JHU-O22, JHU-O11, JHU-O29, OFK6/TERT1, FaDu, CAL 27)	3.48	iTRAQ	23665456
TRA2B	head and neck cancer	neoplastic (JHU-O28, JHU-O22, JHU-O11, JHU-O29, OFK6/TERT1, FaDu, CAL 27)	1.52	iTRAQ	23665456
TRMT6	head and neck cancer	neoplastic (JHU-O28, JHU-O22, JHU-O11, JHU-O29, OFK6/TERT1, FaDu, CAL 27)	1.20	iTRAQ	23665456
TROVE2	head and neck cancer	neoplastic (JHU-O28, JHU-O22, JHU-O11, JHU-O29, OFK6/TERT1, FaDu, CAL 27)	1.02	iTRAQ	23665456
TRPM7	head and neck cancer	neoplastic (JHU-O28, JHU-O22, JHU-O11, JHU-O29, OFK6/TERT1, FaDu, CAL 27)	-1.73	iTRAQ	23665456
TSNAXIP1	head and neck cancer	neoplastic (JHU-O28, JHU-O22, JHU-O11, JHU-O29, OFK6/TERT1, FaDu, CAL 27)	-5.64	iTRAQ	23665456
TSTA3	head and neck cancer	neoplastic (JHU-O28, JHU-O22, JHU-O11, JHU-O29, OFK6/TERT1, FaDu, CAL 27)	-1.51	iTRAQ	23665456
TSTD1	head and neck cancer	neoplastic (JHU-O28, JHU-O22, JHU-O11, JHU-O29, OFK6/TERT1, FaDu, CAL 27)	-1.25	iTRAQ	23665456
TTC38	head and neck cancer	neoplastic (JHU-O28, JHU-O22, JHU-O11, JHU-O29, OFK6/TERT1, FaDu, CAL 27)	-1.18	iTRAQ	23665456
TTLL9	head and neck cancer	neoplastic (JHU-O28, JHU-O22, JHU-O11, JHU-O29, OFK6/TERT1, FaDu, CAL 27)	4.28	iTRAQ	23665456
TUBA1C	head and neck cancer	neoplastic (JHU-O28, JHU-O22, JHU-O11, JHU-O29, OFK6/TERT1, FaDu, CAL 27)	1.75	iTRAQ	23665456
TUBA4A	head and neck cancer	neoplastic (JHU-O28, JHU-O22, JHU-O11, JHU-O29, OFK6/TERT1, FaDu, CAL 27)	1.79	iTRAQ	23665456
TUBB	head and neck cancer	neoplastic (JHU-O28, JHU-O22, JHU-O11, JHU-O29, OFK6/TERT1, FaDu, CAL 27)	2.44	iTRAQ	23665456
TUBB2C	head and neck cancer	neoplastic (JHU-O28, JHU-O22, JHU-O11, JHU-O29, OFK6/TERT1, FaDu, CAL 27)	1.95	iTRAQ	23665456
TUBB3	head and neck cancer	neoplastic (JHU-O28, JHU-O22, JHU-O11, JHU-O29, OFK6/TERT1, FaDu, CAL 27)	2.96	iTRAQ	23665456
TUBB6	head and neck cancer	neoplastic (JHU-O28, JHU-O22, JHU-O11, JHU-O29, OFK6/TERT1, FaDu, CAL 27)	1.08	iTRAQ	23665456
TWF2	head and neck cancer	neoplastic (JHU-O28, JHU-O22, JHU-O11, JHU-O29, OFK6/TERT1, FaDu, CAL 27)	-1.68	iTRAQ	23665456
TXNDC17	head and neck cancer	neoplastic (JHU-O28, JHU-O22, JHU-O11, JHU-O29, OFK6/TERT1, FaDu, CAL 27)	-1.55	iTRAQ	23665456
TXNDC5	head and neck cancer	neoplastic (JHU-O28, JHU-O22, JHU-O11, JHU-O29, OFK6/TERT1, FaDu, CAL 27)	-1.15	iTRAQ	23665456
TXNRD1	head and neck cancer	neoplastic (JHU-O28, JHU-O22, JHU-O11, JHU-O29, OFK6/TERT1, FaDu, CAL 27)	1.79	iTRAQ	23665456
TXNRD1	head and neck cancer	neoplastic (JHU-O28, JHU-O22, JHU-O11, JHU-O29, OFK6/TERT1, FaDu, CAL 27)	-1.88	iTRAQ	23665456
U2AF1	head and neck cancer	neoplastic (JHU-O28, JHU-O22, JHU-O11, JHU-O29, OFK6/TERT1, FaDu, CAL 27)	2.37	iTRAQ	23665456
UBA1	head and neck cancer	neoplastic (JHU-O28, JHU-O22, JHU-O11, JHU-O29, OFK6/TERT1, FaDu, CAL 27)	-1.25	iTRAQ	23665456
UBA7	head and neck cancer	neoplastic (JHU-O28, JHU-O22, JHU-O11, JHU-O29, OFK6/TERT1, FaDu, CAL 27)	-1.15	iTRAQ	23665456
UBE2E2	head and neck cancer	neoplastic (JHU-O28, JHU-O22, JHU-O11, JHU-O29, OFK6/TERT1, FaDu, CAL 27)	-1.94	iTRAQ	23665456
UBE2E3	head and neck cancer	neoplastic (JHU-O28, JHU-O22, JHU-O11, JHU-O29, OFK6/TERT1, FaDu, CAL 27)	-1.21	iTRAQ	23665456
UBE2G1	head and neck cancer	neoplastic (JHU-O28, JHU-O22, JHU-O11, JHU-O29, OFK6/TERT1, FaDu, CAL 27)	-1.18	iTRAQ	23665456
UBE2K	head and neck cancer	neoplastic (JHU-O28, JHU-O22, JHU-O11, JHU-O29, OFK6/TERT1, FaDu, CAL 27)	-1.25	iTRAQ	23665456

UBE2N	head and neck cancer	neoplastic (JHU-O28, JHU-O22, JHU-O11, JHU-O29, OFK6/TERT1, FaDu, CAL 27)	-1.25	iTRAQ	23665456
ACTN4	lung cancer metastasis	neoplastic (SPC-A-1 sci, SPC-A-1)	-2.25	SWATH	24667143
ALPP	lung cancer metastasis	neoplastic (SPC-A-1 sci, SPC-A-1)	2.28	SWATH	24667143
ARSA	lung cancer metastasis	neoplastic (SPC-A-1 sci, SPC-A-1)	1.40	SWATH	24667143
ASS1	lung cancer metastasis	neoplastic (SPC-A-1 sci, SPC-A-1)	1.49	SWATH	24667143
ASS1	lung cancer metastasis	neoplastic (SPC-A-1 sci, SPC-A-1)	1.70	i TRAQ	24667143
B3GNT1	lung cancer metastasis	neoplastic (SPC-A-1 sci, SPC-A-1)	1.32	SWATH	24667143
B4GALT1	lung cancer metastasis	neoplastic (SPC-A-1 sci, SPC-A-1)	-2.12	SWATH	24667143
C1R	lung cancer metastasis	neoplastic (SPC-A-1 sci, SPC-A-1)	1.17	SWATH	24667143
C1S	lung cancer metastasis	neoplastic (SPC-A-1 sci, SPC-A-1)	1.36	i TRAQ	24667143
CCDC80	lung cancer metastasis	neoplastic (SPC-A-1 sci, SPC-A-1)	2.32	i TRAQ	24667143
CCT4	lung cancer metastasis	neoplastic (SPC-A-1 sci, SPC-A-1)	-1.25	SWATH	24667143
CCT8	lung cancer metastasis	neoplastic (SPC-A-1 sci, SPC-A-1)	1.18	i TRAQ	24667143
CD109	lung cancer metastasis	neoplastic (SPC-A-1 sci, SPC-A-1)	1.23	i TRAQ	24667143
CD109	lung cancer metastasis	neoplastic (SPC-A-1 sci, SPC-A-1)	-1.59	SWATH	24667143
CFD	lung cancer metastasis	neoplastic (SPC-A-1 sci, SPC-A-1)	1.46	i TRAQ	24667143
CHST14	lung cancer metastasis	neoplastic (SPC-A-1 sci, SPC-A-1)	1.52	i TRAQ	24667143
CHST14	lung cancer metastasis	neoplastic (SPC-A-1 sci, SPC-A-1)	1.61	SWATH	24667143
CLSTN1	lung cancer metastasis	neoplastic (SPC-A-1 sci, SPC-A-1)	1.05	i TRAQ	24667143
CLSTN1	lung cancer metastasis	neoplastic (SPC-A-1 sci, SPC-A-1)	1.24	SWATH	24667143
COL12A1	lung cancer metastasis	neoplastic (SPC-A-1 sci, SPC-A-1)	1.05	i TRAQ	24667143
COL5A1	lung cancer metastasis	neoplastic (SPC-A-1 sci, SPC-A-1)	1.49	i TRAQ	24667143
COL5A1	lung cancer metastasis	neoplastic (SPC-A-1 sci, SPC-A-1)	1.65	SWATH	24667143
COPB1	lung cancer metastasis	neoplastic (SPC-A-1 sci, SPC-A-1)	1.36	SWATH	24667143
CPA4	lung cancer metastasis	neoplastic (SPC-A-1 sci, SPC-A-1)	1.38	i TRAQ	24667143
CPA4	lung cancer metastasis	neoplastic (SPC-A-1 sci, SPC-A-1)	2.00	SWATH	24667143
CTSB	lung cancer metastasis	neoplastic (SPC-A-1 sci, SPC-A-1)	1.61	SWATH	24667143
CTSB	lung cancer metastasis	neoplastic (SPC-A-1 sci, SPC-A-1)	2.13	i TRAQ	24667143
CTSC	lung cancer metastasis	neoplastic (SPC-A-1 sci, SPC-A-1)	1.10	SWATH	24667143
CTSC	lung cancer metastasis	neoplastic (SPC-A-1 sci, SPC-A-1)	1.37	i TRAQ	24667143
CTSL1	lung cancer metastasis	neoplastic (SPC-A-1 sci, SPC-A-1)	1.21	SWATH	24667143
EEF2	lung cancer metastasis	neoplastic (SPC-A-1 sci, SPC-A-1)	-1.68	SWATH	24667143
FABP3	lung cancer metastasis	neoplastic (SPC-A-1 sci, SPC-A-1)	1.22	SWATH	24667143
FABP3	lung cancer metastasis	neoplastic (SPC-A-1 sci, SPC-A-1)	2.07	i TRAQ	24667143
FASN	lung cancer metastasis	neoplastic (SPC-A-1 sci, SPC-A-1)	1.45	i TRAQ	24667143
FBLN1	lung cancer metastasis	neoplastic (SPC-A-1 sci, SPC-A-1)	1.25	SWATH	24667143
FBLN1	lung cancer metastasis	neoplastic (SPC-A-1 sci, SPC-A-1)	1.41	SWATH	24667143
FBLN1	lung cancer metastasis	neoplastic (SPC-A-1 sci, SPC-A-1)	2.01	i TRAQ	24667143
FGFBP1	lung cancer metastasis	neoplastic (SPC-A-1 sci, SPC-A-1)	1.71	i TRAQ	24667143
FH	lung cancer metastasis	neoplastic (SPC-A-1 sci, SPC-A-1)	1.23	i TRAQ	24667143
FUCA1	lung cancer metastasis	neoplastic (SPC-A-1 sci, SPC-A-1)	1.11	SWATH	24667143
FUCA2	lung cancer metastasis	neoplastic (SPC-A-1 sci, SPC-A-1)	-1.25	SWATH	24667143
GGH	lung cancer metastasis	neoplastic (SPC-A-1 sci, SPC-A-1)	1.36	SWATH	24667143

GGH	lung cancer metastasis	neoplastic (SPC-A-1 sci, SPC-A-1)	1.44	i TRAQ	24667143
GOLM1	lung cancer metastasis	neoplastic (SPC-A-1 sci, SPC-A-1)	1.87	SWATH	24667143
GPI	lung cancer metastasis	neoplastic (SPC-A-1 sci, SPC-A-1)	1.74	i TRAQ	24667143
GRN	lung cancer metastasis	neoplastic (SPC-A-1 sci, SPC-A-1)	1.77	SWATH	24667143
GSTP1	lung cancer metastasis	neoplastic (SPC-A-1 sci, SPC-A-1)	-1.59	SWATH	24667143
HSP90AB1	lung cancer metastasis	neoplastic (SPC-A-1 sci, SPC-A-1)	-1.78	SWATH	24667143
HSPA1A	lung cancer metastasis	neoplastic (SPC-A-1 sci, SPC-A-1)	-2.73	SWATH	24667143
HSPA8	lung cancer metastasis	neoplastic (SPC-A-1 sci, SPC-A-1)	-1.51	SWATH	24667143
HSPG2	lung cancer metastasis	neoplastic (SPC-A-1 sci, SPC-A-1)	-1.51	SWATH	24667143
IFI30	lung cancer metastasis	neoplastic (SPC-A-1 sci, SPC-A-1)	1.21	i TRAQ	24667143
IGFBP1	lung cancer metastasis	neoplastic (SPC-A-1 sci, SPC-A-1)	1.51	SWATH	24667143
IGFBP2	lung cancer metastasis	neoplastic (SPC-A-1 sci, SPC-A-1)	1.50	i TRAQ	24667143
IGFBP2	lung cancer metastasis	neoplastic (SPC-A-1 sci, SPC-A-1)	1.91	SWATH	24667143
IGFBP3	lung cancer metastasis	neoplastic (SPC-A-1 sci, SPC-A-1)	1.04	SWATH	24667143
LDHB	lung cancer metastasis	neoplastic (SPC-A-1 sci, SPC-A-1)	1.35	i TRAQ	24667143
LDHB	lung cancer metastasis	neoplastic (SPC-A-1 sci, SPC-A-1)	-1.68	SWATH	24667143
LMNA	lung cancer metastasis	neoplastic (SPC-A-1 sci, SPC-A-1)	-1.51	SWATH	24667143
MMP1	lung cancer metastasis	neoplastic (SPC-A-1 sci, SPC-A-1)	2.18	SWATH	24667143
MMP1	lung cancer metastasis	neoplastic (SPC-A-1 sci, SPC-A-1)	2.50	i TRAQ	24667143
MSLN	lung cancer metastasis	neoplastic (SPC-A-1 sci, SPC-A-1)	1.35	i TRAQ	24667143
MSLN	lung cancer metastasis	neoplastic (SPC-A-1 sci, SPC-A-1)	2.02	SWATH	24667143
NID2	lung cancer metastasis	neoplastic (SPC-A-1 sci, SPC-A-1)	2.07	i TRAQ	24667143
NID2	lung cancer metastasis	neoplastic (SPC-A-1 sci, SPC-A-1)	2.43	SWATH	24667143
NPC2	lung cancer metastasis	neoplastic (SPC-A-1 sci, SPC-A-1)	1.35	i TRAQ	24667143
NPC2	lung cancer metastasis	neoplastic (SPC-A-1 sci, SPC-A-1)	1.54	SWATH	24667143
NPM1	lung cancer metastasis	neoplastic (SPC-A-1 sci, SPC-A-1)	-1.21	SWATH	24667143
PCOLCE	lung cancer metastasis	neoplastic (SPC-A-1 sci, SPC-A-1)	1.46	i TRAQ	24667143
PCOLCE	lung cancer metastasis	neoplastic (SPC-A-1 sci, SPC-A-1)	2.98	SWATH	24667143
PCSK9	lung cancer metastasis	neoplastic (SPC-A-1 sci, SPC-A-1)	-1.15	SWATH	24667143
PLAU	lung cancer metastasis	neoplastic (SPC-A-1 sci, SPC-A-1)	2.09	i TRAQ	24667143
PLAU	lung cancer metastasis	neoplastic (SPC-A-1 sci, SPC-A-1)	2.47	SWATH	24667143
PPIA	lung cancer metastasis	neoplastic (SPC-A-1 sci, SPC-A-1)	1.44	i TRAQ	24667143
PPIA	lung cancer metastasis	neoplastic (SPC-A-1 sci, SPC-A-1)	-1.55	SWATH	24667143
PPIB	lung cancer metastasis	neoplastic (SPC-A-1 sci, SPC-A-1)	1.88	i TRAQ	24667143
PRDX6	lung cancer metastasis	neoplastic (SPC-A-1 sci, SPC-A-1)	1.45	i TRAQ	24667143
PSMA5	lung cancer metastasis	neoplastic (SPC-A-1 sci, SPC-A-1)	-1.05	SWATH	24667143
PTGDS	lung cancer metastasis	neoplastic (SPC-A-1 sci, SPC-A-1)	2.19	SWATH	24667143
PYGB	lung cancer metastasis	neoplastic (SPC-A-1 sci, SPC-A-1)	1.16	i TRAQ	24667143
PYGB	lung cancer metastasis	neoplastic (SPC-A-1 sci, SPC-A-1)	1.33	SWATH	24667143
QPRT	lung cancer metastasis	neoplastic (SPC-A-1 sci, SPC-A-1)	2.06	SWATH	24667143
RBP4	lung cancer metastasis	neoplastic (SPC-A-1 sci, SPC-A-1)	1.79	SWATH	24667143
S100A4	lung cancer metastasis	neoplastic (SPC-A-1 sci, SPC-A-1)	1.07	i TRAQ	24667143
S100A4	lung cancer metastasis	neoplastic (SPC-A-1 sci, SPC-A-1)	-1.55	SWATH	24667143

S100A6	lung cancer metastasis	neoplastic (SPC-A-1 sci, SPC-A-1)	1.19	SWATH	24667143
SFN	lung cancer metastasis	neoplastic (SPC-A-1 sci, SPC-A-1)	-2.05	SWATH	24667143
SPARC	lung cancer metastasis	neoplastic (SPC-A-1 sci, SPC-A-1)	2.42	i TRAQ	24667143
SPARC	lung cancer metastasis	neoplastic (SPC-A-1 sci, SPC-A-1)	3.19	SWATH	24667143
SPP1	lung cancer metastasis	neoplastic (SPC-A-1 sci, SPC-A-1)	1.96	i TRAQ	24667143
SPP1	lung cancer metastasis	neoplastic (SPC-A-1 sci, SPC-A-1)	3.16	SWATH	24667143
TAGLN2	lung cancer metastasis	neoplastic (SPC-A-1 sci, SPC-A-1)	-2.39	SWATH	24667143
TARS	lung cancer metastasis	neoplastic (SPC-A-1 sci, SPC-A-1)	-1.32	SWATH	24667143
THBS1	lung cancer metastasis	neoplastic (SPC-A-1 sci, SPC-A-1)	1.83	i TRAQ	24667143
THBS1	lung cancer metastasis	neoplastic (SPC-A-1 sci, SPC-A-1)	2.10	SWATH	24667143
TNFRSF12A	lung cancer metastasis	neoplastic (SPC-A-1 sci, SPC-A-1)	1.49	SWATH	24667143
UBA1	lung cancer metastasis	neoplastic (SPC-A-1 sci, SPC-A-1)	-1.12	SWATH	24667143
VCAN	lung cancer metastasis	neoplastic (SPC-A-1 sci, SPC-A-1)	2.14	i TRAQ	24667143
VCAN	lung cancer metastasis	neoplastic (SPC-A-1 sci, SPC-A-1)	2.79	SWATH	24667143
XYLT2	lung cancer metastasis	neoplastic (SPC-A-1 sci, SPC-A-1)	1.13	SWATH	24667143
YWHAE	lung cancer metastasis	neoplastic (SPC-A-1 sci, SPC-A-1)	1.16	i TRAQ	24667143
