# Supplementary material for: Cellular Interactions in the Tumor Microenvironment: The Role of Secretome
Source: J Cancer. 2019 Aug 7;10(19):4574–87. doi: 10.7150/jca.21780 (PMC6746126; doi:10.7150/jca.21780)
Supplement: Supplementary file 2 — Table S2. [file jcav10p4574s2.pdf]

Table S2. Genes coding for proteins identified after a literature search in PubMed database. MSC=mesenchymal stem cells; CAF=carcinoma-associated stromal fibroblasts; NF=normal fibroblasts; NHOst=human osteoblast progenitor.

Gene symbol	Focus of study	Cell type/ Cell line	Method/	Reference PMID
DKK3	c-Myc and cancer	non-neoplastic (hT-RPE/MycER, hT-RPE)	c-ICAT	23542208, 22011035
IGFBP6	c-Myc and cancer	non-neoplastic (hT-RPE/MycER, hT-RPE)	c-ICAT	23542208, 22011035
IGFBP7	c-Myc and cancer	non-neoplastic (hT-RPE/MycER, hT-RPE)	c-ICAT	23542208, 22011035
SFRP1	c-Myc and cancer	non-neoplastic (hT-RPE/MycER, hT-RPE)	c-ICAT	23542208, 22011035
TGFB2	c-Myc and cancer	non-neoplastic (hT-RPE/MycER, hT-RPE)	c-ICAT	23542208, 22011035
IGF2	mesenchymal stem cells	non-neoplastic (MSC)	cytokine array	23542208, 21809383
IGFBP2	mesenchymal stem cells	non-neoplastic (MSC)	cytokine array	23542208, 21809383
IGFBP3	mesenchymal stem cells	non-neoplastic (MSC)	cytokine array	23542208, 21809383
IGFBP4	mesenchymal stem cells	non-neoplastic (MSC)	cytokine array	23542208, 21809383
IGFBP6	mesenchymal stem cells	non-neoplastic (MSC)	cytokine array	23542208, 21809383
TGFB1	mesenchymal stem cells	non-neoplastic (MSC)	cytokine array	23542208, 21809383
TGFB2	mesenchymal stem cells	non-neoplastic (MSC)	cytokine array	23542208, 21809383
VEGFA	mesenchymal stem cells	non-neoplastic (MSC)	cytokine array	23542208, 21809383
ANXA5	nasopharyngeal cancer	non-neoplastic (CAF, NF)	non label-based method	23542208, 22969981
CST3	nasopharyngeal cancer	non-neoplastic (CAF, NF)	non label-based method	23542208, 22969981
CTSL	nasopharyngeal cancer	non-neoplastic (CAF, NF)	non label-based method	23542208, 22969981
HGF	breast cancer	non-neoplastic (CAF, NF)	cytokine array	23542208, 21249190
IL11	breast cancer	neoplastic (MDA-MB-231)	ELISA	23542208, 12842083
CCL2	breast cancer	non-neoplastic (MSC, NHOst)	chemokine array	23542208, 19003962
IL6	breast cancer	non-neoplastic (MSC, NHOst)	chemokine array	23542208, 19003962
CXCL8	breast cancer	non-neoplastic (MSC, NHOst)	chemokine array	23542208, 19003962
FGF1	oral cancer	non-neoplastic (CAF, NF)	multiplex ELISA	23598279
FGF7	oral cancer	non-neoplastic (CAF, NF)	multiplex ELISA	23598279
HGF	oral cancer	non-neoplastic (CAF, NF)	multiplex ELISA	23598279
IL1A	oral cancer	non-neoplastic (CAF, NF)	multiplex ELISA	23598279
MMP3	oral cancer	non-neoplastic (CAF, NF)	multiplex ELISA	23598279
TGFB1	oral cancer	non-neoplastic (CAF, NF)	multiplex ELISA	23598279
TNF	oral cancer	non-neoplastic (CAF, NF)	multiplex ELISA	23598279
IL6	prostate cancer	non-neoplastic (CAF, NF)	chemokine array	29883428, 19671672
TNF	prostate cancer	non-neoplastic (CAF, NF)	chemokine array	29883428, 19671672
IL1B	prostate cancer	non-neoplastic BH101, BH150, BH153, BPH, PN156	immunoradiometric/ELISA	29883428, 8900430
IL6	prostate cancer	non-neoplastic BH101, BH150, BH153, BPH, PN156	immunoradiometric/ELISA	29883428, 8900430
CXCL8	prostate cancer	non-neoplastic BH101, BH150, BH153, BPH, PN156	immunoradiometric/ELISA	29883428, 8900430
TGFB1	prostate cancer	non-neoplastic BH101, BH150, BH153, BPH, PN156	immunoradiometric/ELISA	29883428, 8900430
ACTB	oral cancer	neoplastic (BICR66F, BICR73F, BICR59F, H357F, H314F, BICR18F, BICR3F) and non-neoplastic (CAF, NHOF1, NHOF2, NHOF6)	non label-based method	29883428, 25117810

HSPA5	oral cancer	neoplastic (BICR66F, BICR73F, BICR59F, H357F, H314F, BICR18F, BICR3F) and non-neoplastic (CAF, NHOF1, NHOF2, NHOF6)	non label-based method	29883428, 25117810
MMP1	oral cancer	neoplastic (BICR66F, BICR73F, BICR59F, H357F, H314F, BICR18F, BICR3F) and non-neoplastic (CAF, NHOF1, NHOF2, NHOF6)	non label-based method	29883428, 25117810
MMP2	oral cancer	neoplastic (BICR66F, BICR73F, BICR59F, H357F, H314F, BICR18F, BICR3F) and non-neoplastic (CAF, NHOF1, NHOF2, NHOF6)	non label-based method	29883428, 25117810
SPARC	oral cancer	neoplastic (BICR66F, BICR73F, BICR59F, H357F, H314F, BICR18F, BICR3F) and non-neoplastic (CAF, NHOF1, NHOF2, NHOF6)	non label-based method	29883428, 25117810
TIMP1	oral cancer	neoplastic (BICR66F, BICR73F, BICR59F, H357F, H314F, BICR18F, BICR3F) and non-neoplastic (CAF, NHOF1, NHOF2, NHOF6)	non label-based method	29883428, 25117810
CXCL1	senescent cells	non-neoplastic (HEF, HSF)	antibody array	21321098, 20169192
IL6	senescent cells	non-neoplastic (HEF, HSF)	antibody array	21321098, 20169192
CXCL8	senescent cells	non-neoplastic (HEF, HSF)	antibody array	21321098, 20169192
CSF2	senescent cells	non-neoplastic (WI-38, IMR-90, BJ, HCA2, hBF184, PrEC, BPH1, RWPE1, PC3)	antibody array	21321098, 19053174
CXCL1	senescent cells	non-neoplastic (WI-38, IMR-90, BJ, HCA2, hBF184, PrEC, BPH1, RWPE1, PC3)	antibody array	21321098, 19053174
CXCL2	senescent cells	non-neoplastic (WI-38, IMR-90, BJ, HCA2, hBF184, PrEC, BPH1, RWPE1, PC3)	antibody array	21321098, 19053174
CXCL5	senescent cells	non-neoplastic (WI-38, IMR-90, BJ, HCA2, hBF184, PrEC, BPH1, RWPE1, PC3)	antibody array	21321098, 19053174
IL6	senescent cells	non-neoplastic (WI-38, IMR-90, BJ, HCA2, hBF184, PrEC, BPH1, RWPE1, PC3)	antibody array	21321098, 19053174
CXCL8	senescent cells	non-neoplastic (WI-38, IMR-90, BJ, HCA2, hBF184, PrEC, BPH1, RWPE1, PC3)	antibody array	21321098, 19053174
PLAUR	senescent cells	non-neoplastic (WI-38, IMR-90, BJ, HCA2, hBF184, PrEC, BPH1, RWPE1, PC3)	antibody array	21321098, 19053174
TIMP2	senescent cells	non-neoplastic (WI-38, IMR-90, BJ, HCA2, hBF184, PrEC, BPH1, RWPE1, PC3)	antibody array	21321098, 19053174
